# Cabozantinib plus atezolizumab in previously untreated advanced hepatocellular carcinoma and previously treated gastric cancer and gastroesophageal junction adenocarcinoma: results from two expansion cohorts of a multicentre, open-label, phase 1b trial (COSMIC-021)

**DOI:** 10.1016/j.eclinm.2023.102376

**Published:** 2023-12-21

**Authors:** Daneng Li, Yohann Loriot, Adam M. Burgoyne, James M. Cleary, Armando Santoro, Daniel Lin, Santiago Ponce Aix, Ignacio Garrido-Laguna, Ramu Sudhagoni, Xiang Guo, Svetlana Andrianova, Scott Paulson

**Affiliations:** a City of Hope Comprehensive Cancer Center, Duarte, CA, USA; b Department of Cancer Medicine, Gustave Roussy Institute, INSERM 981, University Paris-Saclay, Villejuif, France; c University of California San Diego, San Diego, CA, USA; d Dana-Farber Cancer Institute, Harvard Medical School, Boston, MA, USA; e IRCCS Humanitas Research Hospital, Humanitas Cancer Center, Rozzano, Italy; f Humanitas University, Pieve Emanuele, Italy; g Sidney Kimmel Cancer Center, Thomas Jefferson University, Philadelphia, PA, USA; h Hospital Universitario 12 de Octubre, H12O-CNIO Lung Cancer Clinical Research Unit, Universidad Complutense and Ciberonc, Madrid, Spain; i Huntsman Cancer Institute, University of Utah, Salt Lake City, UT, USA; j Exelixis, Inc., Alameda, CA, USA; k Texas Oncology-Baylor Charles A. Sammons Cancer Center, Dallas, TX, USA

**Keywords:** Hepatocellular carcinoma, Gastric cancer, Gastroesophageal junction adenocarcinoma, Cabozantinib, Atezolizumab

## Abstract

**Background:**

Cabozantinib is approved for previously treated advanced hepatocellular carcinoma (aHCC) and has been investigated in gastric cancer (GC) and gastroesophageal junction adenocarcinoma (GEJ). Atezolizumab plus bevacizumab is approved for unresectable or metastatic HCC untreated with prior systemic therapy. We evaluated efficacy and safety of cabozantinib plus atezolizumab in aHCC previously untreated with systemic anticancer therapy or previously treated GC/GEJ.

**Methods:**

COSMIC-021 (ClinicalTrials.gov, NCT03170960) is an open-label, phase 1b study in solid tumours with a dose-escalation stage followed by tumour-specific expansion cohorts, including aHCC (cohort 14) and GC/GEJ (cohort 15). Eligible patients were aged ≥18 years with measurable locally advanced, metastatic, or recurrent disease per RECIST version 1.1. Patients received oral cabozantinib 40 mg daily and intravenous atezolizumab 1200 mg once every 3 weeks until progressive disease or unacceptable toxicity. The primary endpoint was investigator-assessed objective response rate per RECIST version 1.1.

**Findings:**

Patients were screened between February 14, 2019, and May 7, 2020, and 61 (30 aHCC, 31 GC/GEJ) were enrolled and received at least one dose of study treatment. Median duration of follow-up was 31.2 months (IQR 28.5–32.7) for aHCC and 30.4 months (28.7–31.9) for GC/GEJ. Objective response rate was 13% (4/30, 95% CI 4–31) for aHCC and 0% (95% CI 0–11) for GC/GEJ. Six (20%) aHCC patients and three (10%) GC/GEJ patients had treatment-related adverse events resulting in discontinuation of either study drug.

**Interpretation:**

Cabozantinib plus atezolizumab had clinical activity with a manageable safety profile in aHCC previously untreated with systemic anticancer therapy. Clinical activity of cabozantinib plus atezolizumab was minimal in previously treated GC/GEJ.

**Funding:**

10.13039/100010544Exelixis, Inc., Alameda, CA, USA.

**Research in context**

**Evidence before this study**

We searched PubMed for articles published between January 1, 2013, and April 12, 2023, using the terms “tyrosine kinase inhibitor” OR “axitinib” OR “sorafenib” OR “lenvatinib” OR “cabozantinib” OR “regorafenib” AND “immune checkpoint inhibitor” OR “avelumab” OR “pembrolizumab” OR “durvalumab” OR “nivolumab” OR “atezolizumab” OR “tislelizumab” AND “hepatocellular carcinoma” OR “gastric cancer” OR “gastroesophageal junction adenocarcinoma.” Results were not limited to trials published in English. We identified 990 citations related to advanced hepatocellular carcinoma; of these, 12 reported clinical trial results on tyrosine kinase/vascular endothelial growth factor (VEGF) inhibition plus programmed cell death protein 1/programmed cell death ligand 1 (PD-1/PD-L1) inhibition combination in previously untreated patients (four phase 1b, one phase 1/2, six phase 3, one not indicated). Five of these clinical trials were for atezolizumab plus bevacizumab and three were for cabozantinib in combination with either nivolumab (two) or atezolizumab. We identified 1276 citations related to gastric cancer/gastroesophageal junction adenocarcinoma; of these, nine reported clinical trial results on tyrosine kinase/VEGF inhibition plus PD-1/PD-L1 inhibition combination in previously treated patients (two phase 1a/1b, two phase 1b, one phase 1/2, four phase 2). This included two reports for ramucirumab plus durvalumab and one report for cabozantinib plus durvalumab. The existing evidence suggests that the role of combination tyrosine kinase/VEGF inhibition plus immune checkpoint inhibition for patients with advanced hepatocellular carcinoma (aHCC) previously untreated with systemic anticancer therapy and patients with previously treated gastric cancer/gastroesophageal junction adenocarcinoma has not yet been fully investigated.

**Added value of this study**

Results from the cohort of patients with aHCC previously untreated with systemic anticancer therapy from COSMIC-021 established preliminary efficacy of cabozantinib plus atezolizumab and provided a rationale for investigating the combination in aHCC in the phase 3 COSMIC-312 study (NCT03755791). We report the longest follow-up for patients with aHCC receiving this regimen. To our knowledge, this is the first study to investigate cabozantinib plus atezolizumab in patients with previously treated gastric cancer or gastroesophageal junction adenocarcinoma. The safety profile of the combination treatment in each population was similar to previously reported safety profiles of each individual agent.

**Implications of all the available evidence**

The results of this study show tolerable safety and potential clinical activity of cabozantinib plus atezolizumab among untreated patients with advanced liver cancer and minimal activity in previously treated gastric cancer or gastroesophageal junction adenocarcinoma. The use of a tyrosine kinase inhibitor in combination with a PD-1 or PD-L1 immune checkpoint inhibitor in these patient populations requires additional investigation.

## Introduction

Advanced gastrointestinal cancers are associated with poor prognoses, with 5-year survival rates of 4%, 7%, and 6% for metastatic liver, stomach, and esophageal cancers, respectively.^1^, ^2^, ^3^ These grim statistics suggest that despite the current arsenal of systemic treatment options,^4^^,^^5^ additional therapies are needed.

The small-molecule inhibitor cabozantinib inhibits the activity of multiple receptor tyrosine kinases, including MET and vascular endothelial growth factor receptor (VEGFR), resulting in the inhibition of tumour angiogenesis and metastasis.^6^ Cabozantinib is approved as a single-agent therapy in patients with advanced hepatocellular carcinoma (aHCC) who previously received sorafenib.^7^ Furthermore, in a phase 2 randomised discontinuation trial of patients with solid tumours, cabozantinib demonstrated a 5% objective response rate and a 33% disease control rate in the cohort of patients with advanced gastric cancer (GC) or gastroesophageal junction adenocarcinoma (GEJ).^8^

Antibodies against programmed cell death protein 1 (PD-1)/PD ligand 1 (PD-L1) prevent PD-1/PD-L1 interactions between tumour and immune cells, which renders the tumour susceptible to immune cell–mediated attack. Various clinical studies have shown efficacy with anti–PD-1/PD-L1 antibodies in patients with aHCC previously untreated with systemic anticancer therapy.^9^, ^10^, ^11^, ^12^, ^13^ The anti–PD-L1 monoclonal antibody atezolizumab in combination with VEGF inhibitor bevacizumab provided superior outcomes for these patients *vs* sorafenib and was more effective than atezolizumab alone, leading to the approval of this combination as frontline therapy for patients with aHCC.^9^^,^^10^ The combination of the anti–PD-L1 monoclonal antibody durvalumab with the anti-CTLA-4 monoclonal antibody tremelimumab also received approval as frontline therapy based on the significant improvements in overall survival compared with sorafenib alone.^11^ Additionally, single-agent anti–PD-1 monoclonal antibodies pembrolizumab and nivolumab also showed activity in this patient population.^12^^,^^13^ Among patients with advanced GC/GEJ, the PD-1 inhibitor pembrolizumab initially received accelerated approval as a third-line treatment based on clinical activity.^14^ However, this approval has since been withdrawn after the phase 3 KEYNOTE-061 trial did not meet its primary endpoints of improved progression-free survival and overall survival^15^ suggesting that single-agent PD-1/PD-L1 therapy may not be sufficient in this patient population. Treatments of durvalumab plus the anti-VEGFR2 monoclonal antibody ramucirumab and pembrolizumab plus ramucirumab achieved objective responses in patients with advanced GC/GEJ in phase 1a/b studies showing promise for the anti-PD-L1/PD-1 plus VEGF inhibitor combination in these patients.^16^^,^^17^

Inhibition of VEGFR, MET, and other tyrosine kinases including the TAM family of kinases (TYRO3, AXL, and MER) by cabozantinib reduces the potential for tumour growth and creates an immune-permissive tumour microenvironment^6^^,^^18^; PD-1/PD-L1 inhibition by atezolizumab has been shown to reverse T-cell suppression.^19^ Therefore, combining these two mechanisms of action may lead to a synergistic tumour response. In addition, potential synergistic efficacy between PD-1/PD-L1 and VEGF pathway inhibition has been demonstrated in multiple cancers including hepatocellular cancer, endometrial cancer, and renal cancer.^20^, ^21^, ^22^, ^23^ Promising clinical activity of cabozantinib plus immune checkpoint inhibitors for several solid tumour indications has been demonstrated, including improved progression-free survival and overall survival with cabozantinib in combination with nivolumab, an anti–PD-1 monoclonal antibody, *vs* sunitinib as first-line treatment for renal cell carcinoma in the phase 3 CheckMate 9ER study.^20^^,^^21^ The phase 3 COSMIC-312 study evaluated cabozantinib plus atezolizumab *vs* sorafenib for first-line treatment of aHCC and showed that of the primary endpoints, the combination significantly improved progression-free survival but not overall survival.^22^ Limited data are available on the potential of tyrosine kinase inhibitor/immune checkpoint inhibitor combinations for the treatment of GC/GEJ.

In the phase 1b COSMIC-021 trial, we evaluated the combination of cabozantinib plus atezolizumab in patients with advanced solid tumours. Reported here are results for expansion cohort 14, which enrolled patients with aHCC previously untreated with systemic anticancer therapy, and expansion cohort 15, which enrolled patients with previously treated GC/GEJ. These two GI tumour cohorts with a small total number of patients were analyzed using the same methods; thus, reported here together in this manuscript.

## Methods

### Study design

COSMIC-021 was a multicentre, open-label, phase 1b study consisting of a dose-escalation stage in solid tumours followed by a tumour-specific cohort expansion stage. Patients were enrolled in expansion cohort 14 at nine sites in Italy and the United States. Patients were enrolled in expansion cohort 15 at 14 sites in France, Italy, Spain, and the United States. The study adhered to the principles in the Guideline for Good Clinical Practice and the Declaration of Helsinki. The study protocol was reviewed and approved by the institutional review boards at participating sites. All patients gave written, informed consent. The protocol is included in the Appendix.

### Registration and protocol

The study is registered with ClinicalTrials.gov, NCT03170960. The scoping review conducted by our research team and protocol is registered at DOI: 10.17605/OSF.IO/JWTGE.

### Patients

All eligible patients were aged ≥18 years with measurable disease per Response Evaluation Criteria in Solid Tumours version 1.1 (RECIST v1.1) and had inoperable locally advanced, metastatic, or recurrent disease. Eligible patients for cohort 14 had aHCC previously untreated with systemic anticancer therapy, an Eastern Cooperative Oncology Group (ECOG) score ≤1, and a Child-Pugh A classification. Prior local-regional treatment was allowed. Eligible patients for cohort 15 had GC, GEJ, or lower one-third esophageal adenocarcinoma, an ECOG score ≤1, radiographic progression on or after platinum- or fluoropyrimidine-containing chemotherapy, and ≤2 prior lines of therapy (HER2/neu-directed therapy was allowed).

### Procedures

In the dose-escalation stage, patients received oral cabozantinib either 40 mg or 60 mg once daily (QD) in combination with intravenous atezolizumab 1200 mg once every 3 weeks. No dose-limiting toxicities were observed at either cabozantinib dose, and the cabozantinib 40-mg QD dose was selected as the recommended dose for use in the combination, based on clinical activity and tolerability.^21^^,^^24^ In the aHCC and GC/GEJ expansion cohorts, patients received oral cabozantinib 40 mg QD and intravenous atezolizumab 1200 mg once every 3 weeks. Cabozantinib could be dose-reduced from 40 mg QD to 20 mg QD and from 20 mg QD to 20 mg every other day to manage adverse events. Atezolizumab infusions could be delayed for adverse event management; dose reductions were not allowed for atezolizumab. Patients received study treatment until radiographic or clinical disease progression or unacceptable toxicity. Treatment beyond radiographic progression was allowed at the discretion of the investigator in patients who experienced clinical benefit. Patients were followed every 12 weeks (±14 days) for survival until death, withdrawal of consent, or decision to no longer collect study data. Tumour assessments were performed using computed tomography or magnetic resonance imaging at screening, every 6 weeks for the first 12 months, and every 12 weeks thereafter. Tumour response was assessed by investigators using RECIST v1.1. The PD-L1 expression in the archival or recently biopsied tumour samples were assessed using Ventana SP263 assay (Roche Diagnostics, Florham Park, NJ) and characterised according to the combined positive score (CPS, total number of tumour and immune cells stained with PD-L1 divided by the number of all viable tumour cells, then multiplied by 100). The PD-L1 expression levels were categorised as CPS ≥5% or <5%.

### Outcomes

The primary endpoint was investigator-assessed objective response rate per RECIST v1.1. Safety was the secondary endpoint, and assessments included adverse events, treatment-related adverse events, serious adverse events, and adverse events of special interest, including immune-related adverse events for atezolizumab. Exploratory endpoints included investigator-assessed duration of response, progression-free survival per RECIST v1.1, overall survival, and biomarker analyses (samples for biomarker analyses were not required for enrollment). Disease control rate was defined as the percentage of patients with a complete response, partial response, or stable disease.

### Statistical analysis

We estimated 30 patients per cohort for the primary endpoint of objective response rate to ensure the lower bound of the two-sided 80% Blyth-Still-Casella confidence interval (CI) extended ≤12 percentage points from the point estimate.^25^ Each cohort may be expanded by approximately 50 additional patients upon approval by the Study Oversight Committee; the decision regarding additional enrollment was to be based on the clinical significance of the achieved ORR (a minimum observed ORR of around 20% or higher). No formal statistical comparisons to historical controls or between cohorts were planned. Categorical and continuous variables were summarised with descriptive statistics. For time-to-event endpoints (progression-free survival, overall survival, duration of response), medians and 95% CIs were estimated using the Kaplan–Meier method. All enrolled patients received at least one dose of any study treatment and were included in all efficacy and safety analyses. Statistical analyses were performed with SAS® software, version 9.4 (Cary, NC).

### Role of the funding source

The funder was involved in the study design and provided cabozantinib. Roche provided atezolizumab. The funder participated in the data collection, analysis, interpretation, and the decision to submit for publication in collaboration with the authors.

## Results

**Fig. 1:**
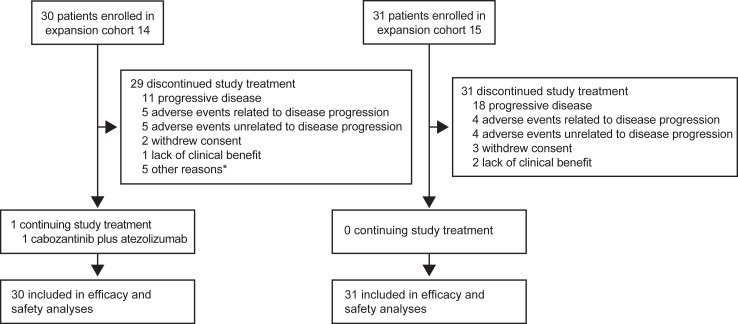
**Trial profile for aHCC (cohort 14) and GC/GEJ (cohort 15) cohorts**. ∗Other reasons for discontinuation in cohort 14 were due to noncompliance (n = 1), robotic left hepatectomy (n = 1), and investigator decision (n = 3).

Between February 14, 2019, and May 7, 2020, we screened 43 and 44 patients for aHCC and GC/GEJ respectively and enrolled 30 for aHCC and 31 for GC/GEJ (22 patients with GEJ, eight patients with GC, one patient with duodenal bulb as primary tumour) (Fig. 1). Eighteen (60%) of the patients with aHCC and 18 (58%) of the patients with GC/GEJ had an ECOG score of 1 (Table 1). Among patients with aHCC, six (20%) had hepatitis B virus (HBV), 11 (37%) had hepatitis C virus (HCV), and 13 (43%) had a nonviral aetiology; two patients (7%) had macrovascular invasion, ten (33%) had portal vein invasion, and 13 (43%) had extrahepatic invasion. Among patients with GC/GEJ, 16 (52%), 14 (45%), and one (3%) had received one, two, and three prior lines of therapy, respectively. Common sites of disease were liver (28 [93%] aHCC, 13 [42%] GC/GEJ), lung (8 [27%] aHCC, 7 [23%] GC/GEJ), and lymph node (7 [23%] aHCC, 17 [55%] GC/GEJ).

**Table 1:** Baseline demographics and clinical characteristics.

	Cohort 14, aHCC (N = 30)	Cohort 15, GC/GEJ (N = 31)
**Age, median (IQR), years**	71 (63–77)	61 (50–68)
**Race**		
White	22 (73)	21 (68)
Asian	6 (20)	0
Other or not reported	2 (7)	10 (32)
**ECOG performance status**		
0	12 (40)	11 (35)
1	18 (60)	18 (58)
2	0	2 (6)
**Disease aetiology**		
HBV	6 (20)	–
HCV	11 (37)	–
Nonviral	13 (43)	–
**Extent of disease**^a^		
Extrahepatic invasion, yes	13 (43)	–
Extrahepatic invasion, no	16 (53)	–
Macrovascular invasion, yes	2 (7)	–
Macrovascular invasion, no	20 (67)	–
Portal vein invasion, yes	10 (33)	–
Portal vein invasion, no	13 (43)	–
**Child-Pugh A**	30 (100)	–
**Primary tumour**		
GC	–	8 (26)^b^
GEJ	–	22 (71)
Other	–	1 (3)^c^
**Prior lines of therapy**		
1	–	16 (52)
2	–	14 (45)
3	–	1 (3)
**Tumour sites per investigator**		
Liver	28 (93)	13 (42)
Lung	8 (27)	7 (23)
Lymph node	7 (23)	17 (55)
Bone	2 (7)	4 (13)
Adrenal	2 (7)	1 (3)
Kidney	0	1 (3)
Other	0	6 (19)

Data are n (%), unless stated otherwise.

aHCC = advanced hepatocellular carcinoma. ECOG = Eastern Cooperative Oncology Group. GC = gastric cancer. GEJ = gastroesophageal junction adenocarcinoma. HBV = hepatitis B virus. HCV = hepatitis C virus. IQR = interquartile range.

a Presence of macrovascular invasion and portal vein invasion were collected separately; extrahepatic, macrovascular, and portal vein invasion were unknown in one, eight, and seven patients, respectively.

b Four cardia, two noncardia, and two unspecified stomach.

c Patient had duodenal bulb as the primary tumour.

The median duration of follow-up was 31.2 months (interquartile range [IQR] 28.5–32.7) for patients with aHCC and 30.4 months (IQR 28.7–31.9) for patients with GC/GEJ. One patient with aHCC remained on study treatment. All patients were included in efficacy and safety analyses.

**Fig. 2:**
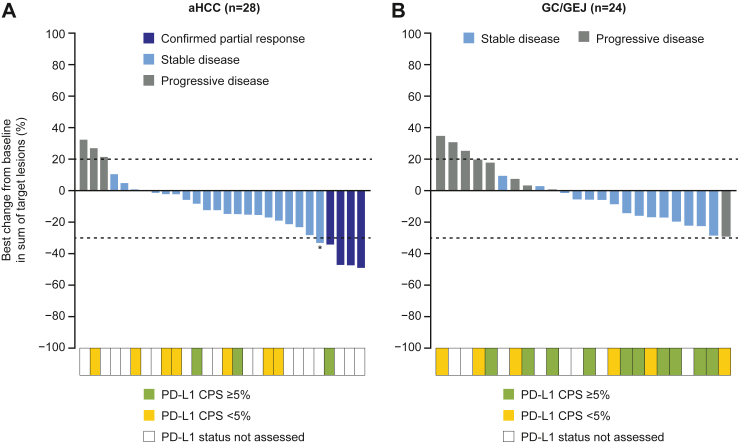
**Waterfall plot of best change from baseline in sum of tumour lesions for individual patients per investigator**. (A) aHCC. (B) GC/GEJ. Waterfall plots show the maximum percentage reduction or minimum percentage increase from baseline in sum of diameters of target lesions. Only patients with measurable disease and at least one postbaseline assessment are shown (n = 28 for aHCC; n = 24 for GC/GEJ). One patient in the GC/GEJ cohort had progressive disease on Week 7 due to a new measurable lesion and was not included in the plot. PD-L1 status categorised as CPS ≥5% or <5%. ∗The patient had an unconfirmed partial response and was therefore categorized as having stable disease. aHCC = advanced hepatocellular carcinoma. CPS = combined positive score. GC = gastric cancer. GEJ = gastroesophageal junction adenocarcinoma. PD-L1 = programmed cell death ligand 1.

Among patients with aHCC, the objective response rate was 13% (95% CI 4–31), with four confirmed partial responses (Table 2). The disease control rate was 83% (95% CI 65–94), with 21 cases of stable disease (70%); median duration of objective response was 22 months (IQR 6.5–22.1), and median time to objective response was 11 months (IQR 4.8–19.6). Among patients with GC/GEJ, the objective response rate was 0% (95% CI 0–11); disease control rate was 48% (95% CI 30–67), with 15 (48%) cases of stable disease. Waterfall plots of the best change from baseline in sum of target lesions per investigator by RECIST v1.1 are shown in Fig. 2. No clear relationship between the level of change in sum of target lesions and PD-L1 status was observed for either cohort.

**Table 2:** Tumour response per investigator per RECIST version 1.1.

	Cohort 14, aHCC (N = 30)	Cohort 15, GC/GEJ (N = 31)
**Objective response rate, n (%)**	4 (13)	0
95% CI, %	4–31	0–11
**Best overall response, n (%)**		
Confirmed complete response	0	0
Confirmed partial response	4 (13)	0
Stable disease	21 (70)	15 (48)
Progressive disease	3 (10)	10 (32)
Missing/not evaluable^a^	2 (7)	6 (19)
**Disease control rate, n (%)**^b^	25 (83)	15 (48)
95% CI, %	65–94	30–67
**Duration of objective response, median (IQR), months**	22.1 (6.5–22.1)	NA
**Time to objective response, median (IQR), months**	11.0 (4.8–19.6)	NA

Data are n (%), unless stated otherwise.

aHCC = advanced hepatocellular carcinoma. CI = confidence interval. GC = gastric cancer. GEJ = gastroesophageal junction adenocarcinoma. IQR = interquartile range. NA = not applicable. RECIST = Response Evaluation Criteria in Solid Tumours.

a In Cohort 14, one patient died prior to the first post-baseline assessment, and the other patient did not meet the criteria for evaluation of response. In Cohort 15, four patients died, one patient withdrew from the study prior to their first post-baseline assessment, and post-baseline assessments were missing for one patient. Deaths in both were not related to the study treatment.

b Disease control rate = complete response + partial response + stable disease.

**Fig. 3:**
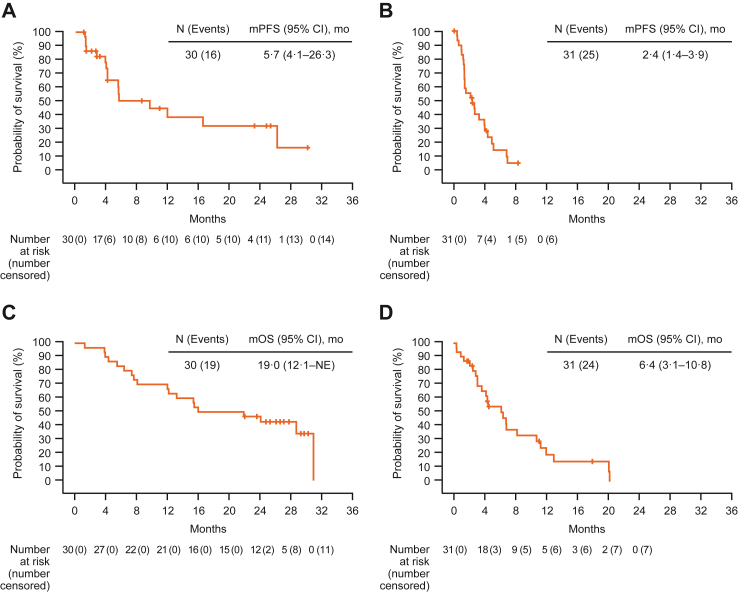
**Kaplan–Meier estimates of progression-free survival per investigator by RECIST v1.1 and overall survival**. (A) aHCC progression-free survival. (B) GC/GEJ progression-free survival. (C) aHCC overall survival. (D) GC/GEJ overall survival. aHCC = advanced hepatocellular carcinoma. CI = confidence interval. GC = gastric cancer. GEJ = gastroesophageal junction adenocarcinoma. mo = months. mOS = median overall survival. mPFS = median progression-free survival. NE = not estimable. RECIST = Response Evaluation Criteria in Solid Tumours.

Among patients with aHCC, median progression-free survival was 5.7 months (95% CI 4.1–26.3), and median overall survival was 19.0 months (95% CI 12.1–not estimable [NE]) (Fig. 3). Responses were observed irrespective of whether patients with aHCC had extrahepatic spread, macrovascular invasion, HBV, or HCV (Appendix p 3). Additionally, responses were observed in patients whose Child-Pugh score was five but not in those with a score of six (Appendix p 3). Similarly, patients with albumin-bilirubin (ALBI) score of one had responses but no responses were observed with those with a score of two (Appendix p 3). The median progression-free survival and overall survival were longer in patients with a Child-Pugh score of five vs six and those with an ALBI score of one vs two (Appendix p 3). Among patients with GC/GEJ, median progression-free survival was 2.4 months (95% CI 1.4–3.9), and median overall survival was 6.4 months (95% CI 3.1–10.8).

Median duration of treatment was 5.8 months (IQR 2.8–12.2) for patients with aHCC and 2.7 months (IQR 1.8–4.2) for patients with GC/GEJ (Appendix pp 4, 8). Twenty-six (87%) patients with aHCC and 16 (52%) patients with GC/GEJ experienced adverse events leading to cabozantinib dose delays (Appendix p 4), and 16 (53%) and seven (23%), respectively, experienced adverse events leading to cabozantinib dose reductions. Fifteen (50%) and 11 (35%) patients with aHCC and GC/GEJ, respectively, experienced adverse events leading to atezolizumab dose delays. Six (20%) patients with aHCC and three (10%) patients with GC/GEJ experienced treatment-related adverse events leading to discontinuation of cabozantinib and/or atezolizumab.

Twenty-eight (93%) patients with aHCC and 25 (81%) patients with GC/GEJ experienced any-grade treatment-related adverse events; 12 (40%) and 11 (35%) experienced grade 3/4 treatment-related adverse events (Table 3). Common treatment-related adverse events included palmar-plantar erythrodysaesthesia, diarrhoea, fatigue, and increased aspartate aminotransferase. No grade 5 treatment-related adverse events occurred in either cohort. Fourteen (47%) patients with aHCC and 16 (52%) patients with GC/GEJ experienced grade 3/4 treatment-emergent adverse events (Appendix pp 5–6). Grade 5 treatment-emergent adverse events occurred in four patients with aHCC (disease progression, hepatic failure, multiple organ dysfunction syndrome, upper gastrointestinal haemorrhage) and seven patients with GC/GEJ (disease progression [6], sepsis).

**Table 3:** Treatment-related adverse events.^a^^,^^b^^,^^c^

	Cohort 14, aHCC (N = 30)			Cohort 15, GC/GEJ (N = 31)		
	Grade 1–2	Grade 3	Grade 4	Grade 1–2	Grade 3	Grade 4
**Any treatment-related adverse event**	16 (53)	11 (37)	1 (3)	14 (45)	10 (32)	1 (3)
Palmar-plantar erythrodysaesthesia	12 (40)	2 (7)	0	4 (13)	0	0
Diarrhoea	8 (27)	3 (10)	0	6 (19)	2 (6)	0
Fatigue	7 (23)	0	0	7 (23)	0	0
Aspartate aminotransferase increased	6 (20)	4 (13)	0	3 (10)	1 (3)	0
Nausea	6 (20)	0	0	4 (13)	0	0
Alanine aminotransferase increased	5 (17)	1 (3)	0	1 (3)	0	0
Decreased appetite	5 (17)	0	0	3 (10)	0	0
Hypertension	5 (17)	1 (3)	0	2 (6)	1 (3)	0
Hypothyroidism	5 (17)	0	0	2 (6)	0	0
Rash	5 (17)	0	0	0	1 (3)	0
Thrombocytopaenia	5 (17)	0	0	1 (3)	2 (6)	0
Asthaenia	3 (10)	0	0	4 (13)	0	0
Dry mouth	3 (10)	0	0	0	0	0
Leukopaenia	3 (10)	0	0	0	0	0
Mucosal inflammation	3 (10)	0	0	0	0	0
Proteinuria	3 (10)	0	0	2 (6)	0	0
Vomiting	3 (10)	0	0	2 (6)	0	0
Blood bilirubin increased	2 (7)	1 (3)	0	1 (3)	0	0
Stomatitis	2 (7)	1 (3)	0	2 (6)	0	0
Transaminases increased	2 (7)	1 (3)	1 (3)	0	0	0
Abdominal pain	1 (3)	1 (3)	0	1 (3)	0	0
Amylase increased	1 (3)	0	0	1 (3)	1 (3)	0
Hyponatraemia	1 (3)	1 (3)	0	0	0	0
Hypophosphataemia	1 (3)	1 (3)	0	2 (6)	1 (3)	0
Infusion-related reaction	1 (3)	0	0	0	0	1 (3)
Neutropaenia	1 (3)	0	0	2 (6)	1 (3)	0
Anaemia	0	0	0	4 (13)	0	0
Hyperbilirubinaemia	0	1 (3)	0	0	0	0
Muscular weakness	0	1 (3)	0	0	0	0
Myocarditis	0	0	0	0	1 (3)	0
White blood cell count decreased	0	0	0	4 (13)	1 (3)	0

Data are n (%).

Adverse events are summarised by decreasing frequency of grade 1–2 for aHCC cohort.

aHCC = advanced hepatocellular carcinoma. GC = gastric cancer. GEJ = gastroesophageal junction adenocarcinoma.

a Grade 1–2 events that occurred in at least 10% of patients and all grade 3–5 events.

b There were no grade 5 treatment-related adverse events in either cohort.

c More than one treatment-related adverse event may have occurred for an individual patient.

Twenty-nine (97%) patients with aHCC and 17 (55%) patients with GC/GEJ experienced adverse event of special interest of any grade; 11 (37%) and seven (23%) patients, respectively, experienced a grade 3/4 adverse event of special interest (Appendix p 7). Common adverse events of special interest included hepatitis (diagnosis and laboratory abnormalities), rash, pancreatitis, and hypothyroidism.

## Discussion

In this cohort analysis of the phase 1b COSMIC 021 study, we report the efficacy and safety of cabozantinib plus atezolizumab treatment for patients with aHCC previously untreated with a systemic anticancer therapy and patients with previously treated GC/GEJ. Notably, this is the longest follow-up reported for this combination in an aHCC cohort, and this is the first study to evaluate cabozantinib plus atezolizumab in patients with previously treated GC/GEJ.

Among patients with aHCC, the current first-line standard-of-care treatments are atezolizumab plus bevacizumab and durvalumab plus tremelimumab.^10^^,^^11^ Encouragingly, the progression-free survival and overall survival for cabozantinib plus atezolizumab in COSMIC-021 were similar to those reported in the phase 3 IMbrave150 study of atezolizumab plus bevacizumab and phase 3 HIMALAYA trial of durvalumab plus tremelimumab.^10^^,^^11^^,^^26^ In the aHCC cohort of the COSMIC-021 study, the progression-free survival was 5.7 months, median overall survival was 19.0 months, and the ORR was 13%, while in the IMbrave150 study, the median progression-free survival was 6.9 months, median overall survival was 19.2 months, and the ORR was 30% and in the HIMALAYA study the median progression-free survival of 3.8 months, median overall survival of 16.4 months and the ORR was 20%.^11^^,^^26^

During further evaluation of cabozantinib plus atezolizumab in previously untreated aHCC in the COSMIC-312 phase 3 study, the median progression-free survival (one of the dual primary endpoints) showed an improvement with cabozantinib plus atezolizumab *vs* sorafenib (6.8 *vs* 4.2 months), but the study did not meet the other primary endpoint for improved interim median overall survival (15.4 *vs* 15.5 months).^22^ Similar to COSMIC-312, the recent phase 3 LEAP-002 trial of lenvatinib plus pembrolizumab *vs* lenvatinib as a first-line treatment in patients with unresectable aHCC reported that its primary endpoint of overall survival was not met.^27^ However, a phase 3 trial of PD-1 inhibition with camrelizumab plus tyrosine kinase inhibition with rivoceranib *vs* sorafenib as a first-line treatment for patients with unresectable aHCC met its primary endpoints of improved progression-free survival and overall survival, although the patient population differed from other aHCC phase 3 trials because the majority of patients (>80%) were recruited at centres in Asia and had a hepatitis B etiology (>72%).^28^ The safety and toxicity profile of cabozantinib plus atezolizumab in the aHCC cohort of COSMIC-021 was comparable to that observed in the larger population of patients with aHCC in COSMIC-312, and of that reported with atezolizumab plus bevacizumab, a current first-line standard of care for aHCC.^10^^,^^22^^,^^26^ Subgroup analyses of the COSMIC-021 aHCC cohort suggested improved clinical outcomes in patients with better hepatic function at baseline based on Child-Pugh score as well as ALBI grade, but the sample size of the cohort and the lack of a control arm limit interpretation of these data. A recent post hoc analysis of IMbrave150 indicated that better liver function at baseline (defined by ALBI grade) was associated with improved outcomes with atezolizumab plus bevacizumab and may have been predictive of outcomes.^29^ Taken together, these data suggest that the role of combination tyrosine kinase inhibition plus PD-1/PD-L1 immune checkpoint inhibition requires additional investigation in patients with aHCC, with a focus on strategies to identify patient subgroups that may benefit from the combination.

Patients with GC/GEJ in COSMIC-021 did not demonstrate a response to cabozantinib plus atezolizumab. In contrast, the phase 1 JVDJ study showed that six of 29 patients with previously treated GC/GEJ who received ramucirumab plus durvalumab had a partial response (21% objective response rate).^16^ It is important to note patients in the COSMIC-021 study had received more prior lines of therapy *vs* JVDJ study (≥2 prior lines, 48% *vs* 28%). Disease control rate (55%), incidence of grade ≥3 treatment-related adverse events (37.9%), and median progression-free survival (2.6 months) from JVDJ study were similar to those reported in COSMIC-021 (disease control rate 48%; grade ≥3 treatment-related adverse events 35%; median progression-free survival 2.4 months). Interestingly, five of the six partial responders in the JVDJ study had high PD-L1–expressing tumours (CPS ≥25%), suggesting that high PD-L1 expression may influence tumour response to combination VEGF inhibition plus PD-1/PD-L1 inhibition in this population. In addition, a phase 1a/1b study of ramucirumab plus pembrolizumab in patients with previously treated GC/GEJ^17^ and a phase 2 study of lenvatinib plus pembrolizumab in patients with GC/GEJ^30^ reported increased response rates and longer median progression-free survival for patients with PD-L1 levels in tumours by CPS ≥1% compared with CPS <1%. Furthermore, the phase 1b CAMILLA study of cabozantinib plus durvalumab in patients with various gastrointestinal malignancies including 10 patients with advanced GC/GEJ also reported that patients with PD-L1 levels in tumours by CPS >5% *vs* overall population had improved objective response rate, disease control rate, median progression-free survival, and median overall survival.^31^ The COSMIC-021 trial did not find a clear relationship between individual patient target lesion change and PD-L1 status for either cohort but was limited by the low number of patients with known PD-L1 status (aHCC: n = 10; GC/GEJ: n = 16). As differences in assays and expression cutoffs make comparisons across studies challenging, further research to better define optimal cutoff of this potential biomarker of response to combination treatment in this patient population is needed.

Limitations of COSMIC-021 include the small sample size of each cohort and the single-arm design. Given a lack of response to treatment in the GC/GEJ cohort, we were unable to correlate potential biomarkers with tumour response in this population. Extended biomarker analyses, including mechanism of resistance to the combination regimen, could not be assessed due to the scarcity of samples with biomarker or molecular data.

In these expansion cohorts of COSMIC-021, we found that cabozantinib plus atezolizumab had clinical activity with a manageable safety profile in patients with aHCC previously untreated with systemic anticancer therapy. Clinical activity of cabozantinib plus atezolizumab was minimal in patients with previously treated GC/GEJ.

## Contributors

RS, XG, and SA contributed to the study design. All authors contributed to the data collection and interpretation. RS, XG, and SA contributed to the data analysis. All authors had full access to and verified all of the data in the study. All authors (DLi, YL, AMB, JMC, AS, DLin, SPA, IG-L, RS, XG, SA, SP) contributed to the drafting of the manuscript. All authors were involved in the review and editing of the manuscript, agree to be accountable for all aspects of the work, and accept responsibility for the decision to submit for publication.

## Data sharing statement

Individual participant data will not be made available.

## Declaration of interests

Daneng Li: Advisory/Consultancy: Adagene, AstraZeneca, Delcath, Eisai, Exelixis, Genentech, Ipsen, Merck, QED, TerSera Therapeutics; Honoraria: Coherus, Eisai, Exelixis, Ipsen, Servier, TerSera Therapeutics; Research Grant/Funding (institution): AstraZeneca, Brooklyn Immunotherapeutics; Travel Accommodations/Expenses: Genentech.

Yohann Loriot: Honoraria: Astellas Pharma, AstraZeneca, Bristol Myers Squibb, Gilead, Janssen, Merck KGaA, MSD (Merck Sharp & Dohme), Pfizer; Travel Accommodation/Expenses: Astellas Pharma, Bristol Myers Squibb, Janssen, Merck KGaA, MSD (Merck Sharp & Dohme), Pfizer, Roche.

Adam Burgoyne: Advisory/Consultancy: Deciphera, Kuur Therapeutics; Research Funding (institution): AstraZeneca, Deciphera, Exelixis, Genentech, Hengrui, Merck, Natera; Speaker Bureau/Expert Testimony: AstraZeneca, Deciphera; Participation on Advisory Boards: Deciphera, Eisai, Exelixis, Genentech.

James M Cleary: Honoraria: Syros Pharmaceuticals, Incyte, Blueprint Medicines; Research Grant/Funding (institution): Merus, Roche, Bristol Myers Squibb; Research Grant/Funding (self): Merck, AstraZeneca, Esperas Pharma, Bayer, Tesaro, Arcus Biosciences, Apexigen; Travel Accommodation/Expenses: Incyte.

Armando Santoro: Advisory/Consultancy: Sanofi, Incyte; Honoraria: Takeda, BMS (Bristol-Myers-Squibb), Roche, AbbVie, Amgen, Celgene, Servier, Gilead, AstraZeneca, Pfizer, Arqule, Eli Lilly, Sandoz, Eisai, Novartis, Bayer, MSD (Merck Sharp & Dohme); Participation on a Data Safety Monitoring Board or Advisory Board: BMS (Bristol-Myers-Squibb), Servier, Gilead, Pfizer, Eisai, Bayer, MSD (Merck Sharp & Dohme).

Daniel Lin: Advisory/Consultancy: Exelixis; Stock Ownership: Bionano Genomics.

Santiago Ponce Aix: Honoraria: AstraZeneca; Patents, Royalties, or Other Intellectual Property: Pharmamar.

Ignacio Garrido-Laguna: Advisory/Consultancy: Kanaph, Jazz Pharmaceuticals, OncoXerna; Participation on Data Safety Monitoring Board: SOTIO.

Ramu Sudhagoni: employed by and have stock ownership in Exelixis.

Svetlana Andrianova: Employment: Exelixis; Spouse Employment: InhibRx; Stock Ownership: Exelixis; Stock Ownership (spouse): InhibRx.

Xiang Guo: employed by Exelixis; Patents Filed: Methods for determining responsiveness to TYK2 inhibitor; Stock Ownership: AbbVie, AstraZeneca, Exelixis, Pfizer.

Scott Paulson: Advisory/Consultancy: Aadi, Advanced Accelerator Applications, Amgen, Astellas Pharma, AstraZeneca, Bristol Myers Squibb, Eisai, Eli Lilly, EMD Serono, Exelixis, Hutchinson MediPharma, Incyte, Ipsen, Mirati Therapeutics, Pfizer, QED Therapeutics, Servier, Stromatis Pharma; Honoraria: Cardinal Health; Research Grant/Funding (institution): AstraZeneca, Bayer, BioNTech SE, Bristol Myers Squibb, Camurus, Deciphera, Exelixis, Eli Lilly, G1 Therapeutics, Gilead Sciences, Gritstone Bio, Hutchinson MediPharma, Incyte, Innovative Cellular Therapeutics Co, Ipsen, Merck, Nucana, Regenxbio, Relay Therapeutics, Seagen, Sotio, Taiho Pharmaceutical, Tempus, Zentalis; Stock or Stock Options: Actinium Pharmaceuticals, Aptose Biosciences, Alexion Pharmaceuticals, Lynx Health, Stromatis Pharma; Travel Accommodations/Expenses: Camurus, Pfizer.

## Appendix A Supplementary data

Supplementary Tables and Figure

Clinical Study Protocol
