# Supplementary material for: Cabozantinib plus atezolizumab in previously untreated advanced hepatocellular carcinoma and previously treated gastric cancer and gastroesophageal junction adenocarcinoma: results from two expansion cohorts of a multicentre, open-label, phase 1b trial (COSMIC-021)
Source: eClinicalMedicine. 2023 Dec 21;67:102376. doi: 10.1016/j.eclinm.2023.102376 (PMC10776423; doi:10.1016/j.eclinm.2023.102376)
Supplement: Supplementary Tables and Figure [file mmc1.docx]

**SUPPLEMENTARY APPENDIX**

**Cabozantinib plus atezolizumab in previously untreated advanced hepatocellular carcinoma and previously treated gastric cancer and gastroesophageal junction adenocarcinoma: results from two expansion cohorts of a multicentre, open-label, phase 1b trial (COSMIC-021)**

Daneng Li, MD, Yohann Loriot, MD, Adam Burgoyne, MD, James M Cleary, MD, Armando Santoro, MD, Daniel Lin, MD, Santiago Ponce Aix, MD, Ignacio Garrido-Laguna, MD, Ramu Sudhagoni, PhD, Xiang Guo, PhD, Svetlana Andrianova, MD, Scott Paulson, MD

**Table of Contents**

[List of investigators 2](#_Toc150342792)

[Supplemental Table 1: Efficacy outcomes in subgroups of interest for cohort 14 (aHCC) 3](#_Toc150342793)

[Supplemental Table 2: Adverse events overview 4](#_Toc150342794)

[Supplemental Table 3: Treatment-emergent adverse events*^†^ 5](#_Toc150342795)

[Supplemental Table 4: Adverse events of special interest* 7](#_Toc150342796)

[Supplemental Figure 1: Duration of treatment and response in (A) aHCC and (B) GC/GEJ 8](#_Toc150342797)

# List of investigators

**Cohort 14**

- **Italy:** Santoro, Armando
- **United States:** Dreicer, Robert; Goldschmidt, Jerome; Hutson, Thomas; Kelly, William; Kurkjian, Carla; McKay, Rana; Niu, Jiaxin (Jason); Pal, Sumanta

**Cohort 15**

- **France:** Loriot, Yohann
- **Italy:** Santoro, Armando
- **Spain:** Ponce Aix, Santiago
- **United States:** Agarwal, Neeraj; Courtney, Kevin; Dreicer, Robert; Fang, Bruno; Kelly, William; McGregor, Bradley; McKay, Rana; Sen, Shiraj; Spencer, Kristen; Squillante, Christian; Wentzel, Kristopher

# *Supplemental* *Table 1*: Efficacy outcomes in subgroups of interest for cohort 14 (aHCC)

|  | **Extrahepatic spread** | | **Macrovascular invasion** | | | **Aetiology** | | | | **Child-Pugh score‡** | | **ALBI score** | | |
| --- | --- | --- | --- | --- | --- | --- | --- | --- | --- | --- | --- | --- | --- | --- |
|  | **Yes**  **n=13** | **No**  **n=16** | **Yes**  **n=11** | **No**  **n=13** | **Unknown**  **n=6** | **HBV**  **n=6** | **HCV**  **n=11** | **NASH**  **n=3** | **Unspecified**  **n=10** | **5**  **n=23** | **6**  **n=5** | **1**  **n=18** | **2**  **n=12** |  |
| **Objective response rate, n (%)** | 2 (15) | 2 (13) | 1 (9) | 3 (23) | 0 | 1 (17) | 2 (18) | 0 | 1 (10) | 3 (13) | 0 | 4 (22) | 0 |  |
| 95% CI, % | 2–45 | 2–38 | 0–41 | 5–54 | 0–46 | 0–64 | 2–52 | 0–71 | 0–45 | 3–34 | 0–52 | 6–48 | (0–27) |  |
| **Best overall response, n (%)** |  |  |  |  |  |  |  |  |  |  |  |  |  |  |
| Confirmed complete response | 0 | 0 | 0 | 0 | 0 | 0 | 0 | 0 | 0 | 0 | 0 | 0 | 0 |  |
| Confirmed partial response | 2 (15) | 2 (13) | 1 (9) | 3 (23) | 0 | 1 (17) | 2 (18) | 0 | 1 (10) | 3 (13) | 0 | 4 (22) | 0 |  |
| Stable disease | 8 (62) | 13 (81) | 7 (64) | 9 (69) | 5 (83) | 4 (67) | 8 (73) | 3 (100) | 6 (60) | 17 (74) | 4 (80) | 11 (61) | 10 (83) |  |
| Progressive disease | 1 (8) | 1 (6) | 1 (9) | 1 (8) | 1 (17) | 1 (17) | 0 | 0 | 2 (20) | 3 (13) | 0 | 3 (17) | 0 |  |
| Missing/not evaluable | 2 (15) | 0 | 2 (18) | 0 | 0 | 0 | 1 (9) | 0 | 1 (10) | 0 | 1 (20) | 0 | 2 (17) |  |
| **Disease control rate, n (%)*** | 10 (77) | 15 (94) | 8 (73) | 12 (92) | 5 (83) | 5 (83) | 10 (91) | 3 (100) | 7 (70) | 20 (87) | 4 (80) | 15 (83) | 10 (83) |  |
| 95% CI, % | 46–95 | 70–100 | 39–94 | 64–100 | 36–100 | 36–100 | 59–100 | 29–100 | 35–93 | 66–97 | 28–100 | 59–96 | 52–98 |  |
| **Progression-free survival, median (95% CI), months** | NA^†^ | 5·7  (3·9–16·6) | 5·7  (1·3–16·6) | 5·6  (4·1–NE) | 9·7  (1·3–NE) | 4·0  (1·4–NE) | 9·7  (4·1–NE) | 5·6  (3·9–NE) | 16·6  (1·2–NE) | 16·6  (5·6–NE) | 4·0  (1·3–NE) | 12·0  (2·8–NE) | 5·6  (3·9–NE) |  |
| **Overall survival, median (95% CI), months** | 8·2  (5·5–NE) | NE  (12·2–NE) | 15·4  (3·9–NE) | 15·5  (7·7–NE) | NE  (13·3–NE) | NE  (4·0–NE) | NE  (7·7–NE) | 12·1  (3·9–NE) | 20·1  (4·4–NE) | 28·8  (13·3–NE) | 4·0  (1·3–NE) | 31·1  (16·1–NE) | 7·9  (3·9–15·5) |  |

% (95% CI) or n (%), unless specified otherwise.

aHCC=advanced hepatocellular carcinoma. ALBI=albumin-billirubin. CI=confidence interval. HBV=hepatitis B virus. HCV=hepatitis C virus. NASH=non-alcoholic steatohepatitis. NA=not applicable. NE=not estimable.

*Disease control rate = complete response + partial response + stable disease. †Not interpretable due to censoring rules and smaller sample size.‡Child-Pugh scores were determined based on baseline laboratory values and two patients who had a score of 7 were not included in the analysis.

# *Supplemental Table 2*: Adverse events overview

|  | **Cohort 14, aHCC (N=30)** | **Cohort 15, GC/GEJ (N=31)** |
| --- | --- | --- |
| Patients on study treatment | 1 (3) | 0 |
| Duration of exposure, months | 5·8 (2·8–12·2) | 2·7 (1·8–4·2) |
| AEs leading to cabozantinib dose delay | 26 (87) | 16 (52) |
| AEs leading to atezolizumab dose delay | 15 (50) | 11 (35) |
| AEs leading to cabozantinib dose reduction | 16 (53) | 7 (23) |
| TRAE leading to discontinuation | | |
| Cabozantinib and/or atezolizumab | 6 (20) | 3 (10) |
| Cabozantinib | 5 (17) | 2 (6) |
| Atezolizumab | 4 (13) | 2 (6) |
| Both | 3 (10) | 1 (3) |

n (%) or median (IQR).

AE=adverse event. aHCC=advanced hepatocellular carcinoma. GC=gastric cancer. GEJ=gastroesophageal junction adenocarcinoma. IQR=interquartile range. TRAE=treatment-related adverse event.

# *Supplemental Table 3*: Treatment-emergent adverse events*^†^

|  | **Cohort 14, aHCC (N=30)** | | | | **Cohort 15, GC/GEJ (N=31)** | | | |
| --- | --- | --- | --- | --- | --- | --- | --- | --- |
|  | **Grade 1–2** | **Grade 3** | **Grade 4** | **Grade 5** | **Grade 1–2** | **Grade 3** | **Grade 4** | **Grade 5** |
| **Any treatment-emergent adverse event** | 12 (40) | 13 (43) | 1 (3) | 4 (13) | 8 (26) | 13 (42) | 3 (10) | 7 (23) |
| Fatigue | 12 (40) | 0 | 0 | 0 | 8 (26) | 0 | 0 | 0 |
| Palmar-plantar erythrodysaesthesia | 12 (40) | 2 (7) | 0 | 0 | 4 (13) | 0 | 0 | 0 |
| Diarrhoea | 10 (33) | 3 (10) | 0 | 0 | 7 (23) | 2 (6) | 0 | 0 |
| Aspartate aminotransferase increased | 9 (30) | 6 (20) | 0 | 0 | 6 (19) | 1 (3) | 0 | 0 |
| Decreased appetite | 9 (30) | 0 | 0 | 0 | 6 (19) | 1 (3) | 0 | 0 |
| Nausea | 9 (30) | 0 | 0 | 0 | 9 (29) | 0 | 0 | 0 |
| Alanine aminotransferase increased | 8 (27) | 1 (3) | 0 | 0 | 1 (3) | 0 | 0 | 0 |
| Blood bilirubin increased | 7 (23) | 1 (3) | 1 (3) | 0 | 1 (3) | 0 | 0 | 0 |
| Constipation | 7 (23) | 0 | 0 | 0 | 5 (16) | 0 | 0 | 0 |
| Hypertension | 6 (20) | 1 (3) | 0 | 0 | 2 (6) | 1 (3) | 0 | 0 |
| Hypothyroidism | 6 (20) | 0 | 0 | 0 | 2 (6) | 0 | 0 | 0 |
| Rash | 6 (20) | 0 | 0 | 0 | 0 | 1 (3) | 0 | 0 |
| Amylase increased | 5 (17) | 1 (3) | 0 | 0 | 2 (6) | 1 (3) | 0 | 0 |
| Anaemia | 5 (17) | 1 (3) | 0 | 0 | 7 (23) | 2 (6) | 0 | 0 |
| Back pain | 5 (17) | 0 | 0 | 0 | 1 (3) | 0 | 0 | 0 |
| Dry mouth | 5 (17) | 0 | 0 | 0 | 0 | 0 | 0 | 0 |
| Gamma-glutamyltransferase increased | 5 (17) | 0 | 0 | 0 | 1 (3) | 0 | 0 | 0 |
| Leukopaenia | 5 (17) | 0 | 0 | 0 | 0 | 0 | 0 | 0 |
| Neutropaenia | 5 (17) | 0 | 0 | 0 | 2 (6) | 1 (3) | 0 | 0 |
| Oedema peripheral | 5 (17) | 0 | 0 | 0 | 3 (10) | 0 | 0 | 0 |
| Proteinuria | 5 (17) | 0 | 0 | 0 | 3 (10) | 0 | 0 | 0 |
| Thrombocytopaenia | 5 (17) | 0 | 0 | 0 | 1 (3) | 2 (6) | 0 | 0 |
| Abdominal pain | 4 (13) | 2 (7) | 0 | 0 | 3 (10) | 1 (3) | 0 | 0 |
| Dizziness | 4 (13) | 0 | 0 | 0 | 2 (6) | 1 (3) | 0 | 0 |
| Vomiting | 4 (13) | 0 | 0 | 0 | 9 (29) | 0 | 0 | 0 |
| Ascites | 3 (10) | 1 (3) | 0 | 0 | 1 (3) | 0 | 0 | 0 |
| Asthaenia | 3 (10) | 1 (3) | 0 | 0 | 8 (26) | 2 (6) | 0 | 0 |
| Blood alkaline phosphatase increased | 3 (10) | 0 | 0 | 0 | 0 | 0 | 0 | 0 |
| Cough | 3 (10) | 0 | 0 | 0 | 4 (13) | 0 | 0 | 0 |
| Dysgeusia | 3 (10) | 0 | 0 | 0 | 3 (10) | 0 | 0 | 0 |
| Dyspepsia | 3 (10) | 0 | 0 | 0 | 0 | 0 | 0 | 0 |
| Dyspnoea | 3 (10) | 0 | 0 | 0 | 3 (10) | 0 | 0 | 0 |
| Headache | 3 (10) | 0 | 0 | 0 | 1 (3) | 0 | 0 | 0 |
| Hypocalcaemia | 3 (10) | 0 | 0 | 0 | 1 (3) | 0 | 0 | 0 |
| Hypomagnesaemia | 3 (10) | 0 | 0 | 0 | 2 (6) | 0 | 0 | 0 |
| Hyponatraemia | 3 (10) | 3 (10) | 0 | 0 | 1 (3) | 1 (3) | 0 | 0 |
| Mucosal inflammation | 3 (10) | 0 | 0 | 0 | 0 | 0 | 0 | 0 |
| Neutrophil count decreased | 3 (10) | 0 | 0 | 0 | 2 (6) | 1 (3) | 0 | 0 |
| Oral pain | 3 (10) | 0 | 0 | 0 | 1 (3) | 0 | 0 | 0 |
| Pain in extremity | 3 (10) | 0 | 0 | 0 | 1 (3) | 0 | 0 | 0 |
| Paraesthesia | 3 (10) | 0 | 0 | 0 | 0 | 0 | 0 | 0 |
| Platelet count decreased | 3 (10) | 1 (3) | 0 | 0 | 1 (3) | 0 | 0 | 0 |
| Pruritus | 3 (10) | 0 | 0 | 0 | 1 (3) | 0 | 0 | 0 |
| Weight decreased | 3 (10) | 0 | 0 | 0 | 6 (19) | 0 | 0 | 0 |
| Hypophosphataemia | 2 (7) | 1 (3) | 0 | 0 | 2 (6) | 1 (3) | 0 | 0 |
| Lipase increased | 2 (7) | 0 | 0 | 0 | 0 | 0 | 1 (3) | 0 |
| Stomatitis | 2 (7) | 1 (3) | 0 | 0 | 2 (6) | 0 | 0 | 0 |
| Transaminases increased | 2 (7) | 1 (3) | 1 (3) | 0 | 0 | 0 | 0 | 0 |
| Hepatic encephalopathy | 1 (3) | 1 (3) | 0 | 0 | 0 | 0 | 0 | 0 |
| Hypernatraemia | 1 (3) | 1 (3) | 0 | 0 | 0 | 0 | 0 | 0 |
| Hypoalbuminaemia | 1 (3) | 1 (3) | 0 | 0 | 6 (19) | 0 | 0 | 0 |
| Infusion-related reaction | 1 (3) | 0 | 0 | 0 | 0 | 0 | 1 (3) | 0 |
| Pyrexia | 1 (3) | 1 (3) | 0 | 0 | 1 (3) | 0 | 0 | 0 |
| White blood cell count decreased | 1 (3) | 0 | 0 | 0 | 5 (16) | 1 (3) | 0 | 0 |
| Bacteraemia | 0 | 0 | 0 | 0 | 1 (3) | 0 | 1 (3) | 0 |
| Bile duct stenosis | 0 | 0 | 0 | 0 | 0 | 1 (3) | 0 | 0 |
| Bilirubin conjugated increased | 0 | 1 (3) | 0 | 0 | 0 | 0 | 0 | 0 |
| Deep vein thrombosis | 0 | 0 | 0 | 0 | 1 (3) | 1 (3) | 0 | 0 |
| Dehydration | 0 | 0 | 0 | 0 | 4 (13) | 0 | 0 | 0 |
| Depression | 0 | 0 | 0 | 0 | 1 (3) | 1 (3) | 0 | 0 |
| Disease progression | 0 | 0 | 0 | 1 (3) | 0 | 0 | 0 | 4 (13) |
| Encephalopathy | 0 | 0 | 0 | 0 | 0 | 0 | 1 (3) | 0 |
| Gastric cancer | 0 | 0 | 0 | 0 | 0 | 0 | 0 | 1 (3) |
| Gastrointestinal haemorrhage | 0 | 0 | 0 | 0 | 0 | 1 (3) | 0 | 0 |
| Gastrostomy tube–site complication | 0 | 0 | 0 | 0 | 0 | 1 (3) | 0 | 0 |
| Hepatic failure | 0 | 0 | 0 | 1 (3) | 0 | 0 | 0 | 0 |
| Hepatocellular injury | 0 | 0 | 0 | 0 | 0 | 1 (3) | 0 | 0 |
| Hyperbilirubinaemia | 0 | 1 (3) | 0 | 0 | 0 | 0 | 0 | 0 |
| Hypoglycaemia | 0 | 0 | 0 | 0 | 0 | 0 | 1 (3) | 0 |
| Intestinal obstruction | 0 | 0 | 0 | 0 | 0 | 0 | 1 (3) | 0 |
| Lumbar radiculopathy | 0 | 0 | 0 | 0 | 0 | 1 (3) | 0 | 0 |
| Malignant neoplasm progression | 0 | 0 | 0 | 0 | 0 | 0 | 0 | 1 (3) |
| Multiple organ dysfunction syndrome | 0 | 0 | 0 | 1 (3) | 0 | 0 | 0 | 0 |
| Muscular weakness | 0 | 1 (3) | 0 | 0 | 0 | 0 | 0 | 0 |
| Myocarditis | 0 | 0 | 0 | 0 | 0 | 1 (3) | 0 | 0 |
| Oedema genital | 0 | 1 (3) | 0 | 0 | 0 | 0 | 0 | 0 |
| Performance status decreased | 0 | 0 | 0 | 0 | 0 | 0 | 1 (3) | 0 |
| Peritonitis bacterial | 0 | 1 (3) | 0 | 0 | 0 | 0 | 0 | 0 |
| Pneumonia | 0 | 0 | 0 | 0 | 1 (3) | 0 | 1 (3) | 0 |
| Pneumonia aspiration | 0 | 0 | 0 | 0 | 0 | 2 (6) | 0 | 0 |
| Postoperative wound infection | 0 | 0 | 0 | 0 | 0 | 0 | 1 (3) | 0 |
| Pulmonary embolism | 0 | 0 | 0 | 0 | 0 | 3 (10) | 0 | 0 |
| Sepsis | 0 | 0 | 0 | 0 | 0 | 0 | 1 (3) | 1 (3) |
| Staphylococcal skin infection | 0 | 1 (3) | 0 | 0 | 0 | 0 | 0 | 0 |
| Tumour pain | 0 | 0 | 0 | 0 | 2 (6) | 1 (3) | 0 | 0 |
| Upper gastrointestinal haemorrhage | 0 | 0 | 0 | 1 (3) | 0 | 0 | 0 | 0 |

Data are n (%).

Adverse events are summarised by decreasing frequency of grade 1–2 for aHCC cohort.

aHCC=advanced hepatocellular carcinoma. GC=gastric cancer. GEJ=gastroesophageal junction adenocarcinoma.

*Grade 1–2 events that occurred in at least 10% of patients and all grade 3–5 events. ^†^More than one treatment-emergent adverse event may have occurred for an individual patient.

# *Supplemental Table 4*: Adverse events of special interest*

|  | **Cohort 14, aHCC (N=30)** | | | | **Cohort 15, GC/GEJ (N=31)** | | | |
| --- | --- | --- | --- | --- | --- | --- | --- | --- |
|  | **Grade 1–2** | **Grade 3** | **Grade 4** | **Grade 5** | **Grade 1–2** | **Grade 3*** | **Grade 4** | **Grade 5** |
| **Any adverse event of special interest** | 17 (57) | 9 (30) | 2 (7) | 1 (3)^†^ | 10 (32) | 5 (16) | 2 (6) | 0 |
| Hepatitis (diagnosis and laboratory abnormalities) | 19 (63) | 8 (27) | 2 (7) | 1 (3)^†^ | 7 (23) | 2 (6) | 0 | 0 |
| Hepatitis (laboratory abnormalities) | 19 (63) | 8 (27) | 2 (7) | 0 | 7 (23) | 1 (3) | 0 | 0 |
| Rash | 19 (63) | 2 (7) | 0 | 0 | 8 (26) | 1 (3) | 0 | 0 |
| Pancreatitis | 7 (23) | 1 (3) | 0 | 0 | 2 (6) | 2 (6) | 1 (3) | 0 |
| Hypothyroidism | 6 (20) | 0 | 0 | 0 | 2 (6) | 0 | 0 | 0 |
| Pneumonitis | 1 (3) | 0 | 0 | 0 | 1 (3) | 0 | 0 | 0 |
| Infusion-related reactions | 1 (3) | 0 | 0 | 0 | 0 | 0 | 1 (3) | 0 |
| Myocarditis | 0 | 0 | 0 | 0 | 0 | 1 (3) | 0 | 0 |

Data are n (%).

Adverse events of special interest are potential immune-related adverse events for atezolizumab provided by the sponsor and summarised as grouped MedDRA terms irrespective of causality; adverse events are summarised by decreasing frequency of any grade in the aHCC cohort.

aHCC=advanced hepatocellular carcinoma. GC=gastric cancer. GEJ=gastroesophageal junction adenocarcinoma. MedDRA=Medical Dictionary for Regulatory Activities. ^*^More than one adverse event of special interest may have occurred for an individual patient. ^†^The Grade 5 event reported in the ‘hepatitis’ group term is the grade 5 treatment-emergent hepatic failure event.

# *Supplemental Figure 1*: Duration of treatment and response in (A) aHCC and (B) GC/GEJ

#
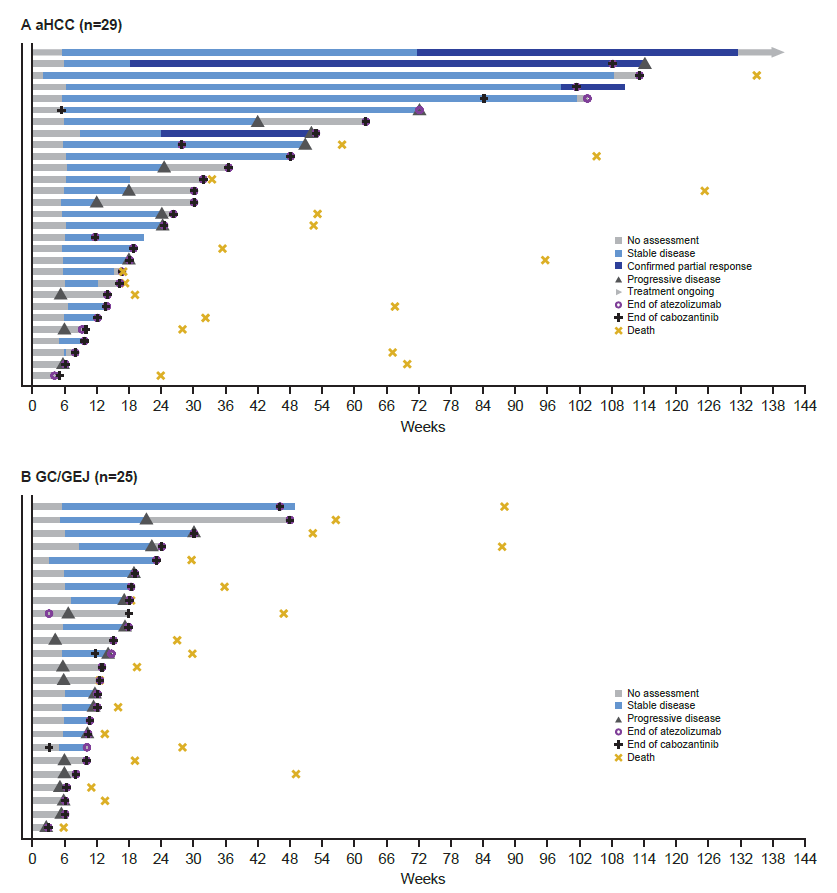


Patients with at least one adequate postbaseline assessment are shown (n=29 for aHCC; n=25 for GC/GEJ).

aHCC=advanced hepatocellular carcinoma. GC=gastric cancer. GEJ=gastroesophageal junction adenocarcinoma.
