# Supplementary material for: Cabozantinib plus atezolizumab in previously untreated advanced hepatocellular carcinoma and previously treated gastric cancer and gastroesophageal junction adenocarcinoma: results from two expansion cohorts of a multicentre, open-label, phase 1b trial (COSMIC-021)
Source: eClinicalMedicine. 2023 Dec 21;67:102376. doi: 10.1016/j.eclinm.2023.102376 (PMC10776423; doi:10.1016/j.eclinm.2023.102376)
Supplement: Clinical Study Protocol [file mmc2.pdf]

CLINICAL STUDY PROTOCOL

A Phase 1b Dose-Escalation Study of Cabozantinib (XL184) Administered Alone or in Combination with Atezolizumab to Subjects with Locally Advanced or Metastatic Solid Tumors

**PROTOCOL NUMBER:**	XL184-021
**STUDY TREATMENT:**	Cabozantinib (XL184) and Atezolizumab
**IND NUMBER:**	72,596
**EudraCT NUMBER:**	2017-001792-24
**SPONSOR:**	Exelixis, Inc.<br>1851 Harbor Bay Parkway<br>Alameda, CA 94502
**MEDICAL MONITOR:**	[redacted]

**FINAL DATE:**	24 March 2017	
**DATE AMENDED:**	20 December 2017	**PROTOCOL AMENDMENT: 1.0**
**DATE AMENDED:**	31 August 2018	**PROTOCOL AMENDMENT: 2.0**
**DATE AMENDED:**	22 February 2019	**PROTOCOL AMENDMENT: 3.0**
**DATE AMENDED:**	02 July 2019	**PROTOCOL AMENDMENT: 4.0**
**DATE AMENDED:**	15 November 2019	**PROTOCOL AMENDMENT: 5.0**
**DATE AMENDED:**	16 April 2020	**PROTOCOL AMENDMENT: 6.0**
**DATE AMENDED:**	11 May 2021	**PROTOCOL AMENDMENT: 7.0**
**DATE AMENDED:**	25 March 2022	**PROTOCOL AMENDMENT: 8.0**

CONFIDENTIAL
This document (and all copies thereof) is the property of Exelixis, Inc. and contains valuable trade secrets and confidential information, including proprietary technical and business information (hereinafter "Confidential Information"). By accepting this document, you agree that the Confidential Information contained herein will not be disclosed to others without prior written authorization from Exelixis, Inc. and shall only be used for the purposes intended by the document.

SYNOPSIS

TITLE

A Phase 1b Dose-Escalation Study of Cabozantinib (XL184) Administered Alone or in Combination with Atezolizumab in Subjects with Locally Advanced or Metastatic Solid Tumors

PROTOCOL NUMBER

XL184-021

CLINICAL PHASE

Phase 1b

RATIONALE

Multi-targeted tyrosine kinase inhibitors (TKIs) and immune checkpoint inhibitors (ICIs) represent two systemic modalities that have been instrumental in the recent advancements of anticancer treatment over the past several years. Both classes of therapies have demonstrated broad clinical effects leading to new approved treatment options across multiple tumor types. The success of these therapy types as single agents with distinct mechanisms of action has naturally led to interest in evaluating combinations of TKIs with ICIs in search of further, possibly synergistic, anticancer clinical effects.

Atezolizumab is a humanized immunoglobulin (Ig) G1 monoclonal antibody that targets programmed death receptor 1 ligand (PD-L1) and inhibits the interaction between PD-L1 and its receptors, programmed death receptor 1 (PD-1) and B7-1 (also known as CD80), both of which function as inhibitory receptors expressed on T cells. Atezolizumab injection for intravenous (IV) use (1200 mg once every 3 weeks [q3w]) has been approved in the US and EU for the treatment of adult patients with advanced urothelial carcinoma (UC) after prior platinum containing chemotherapy or in a subset of patients who are considered cisplatin-ineligible (different patient populations are indicated depending on region; Rosenberg et al 2016, Balar et al 2017). Atezolizumab in combination with bevacizumab, paclitaxel, and carboplatin has been approved in the US for the first-line treatment of adult patients with metastatic non-squamous non-small cell lung cancer (NSCLC) with no epidermal growth factor receptor (EGFR) or anaplastic lymphoma kinase (ALK) genomic tumor aberrations. Atezolizumab has also been approved for adult patients with locally advanced or metastatic NSCLC after prior chemotherapy (Fehrenbacher et al 2016; Tecentriq™ US prescribing information [US PI] and European Medicines Agency Summary of Product Characteristics [EMA SmPC]). Atezolizumab has also been approved for first-line treatment in combination with carboplatin and etoposide in adult patients with extensive-stage small cell lung cancer (ES-SCLC; Horn et al 2018, Tecentriq US PI). Atezolizumab in combination with bevacizumab has been approved for the treatment of patients with unresectable or metastatic HCC who have not received prior systemic therapy (Tecentriq USPI and EMA SmPC). In addition, atezolizumab in combination with cobimetinib and vemurafenib has been approved for the treatment of patients with BRAF V600 mutation-positive unresectable or metastatic melanoma (Tecentriq USPI and EMA SmPC). Treatment with atezolizumab is generally well-tolerated but can be associated with immune-related adverse events (irAEs).

Further, atezolizumab has demonstrated encouraging clinical activity in other tumor treatment settings: monotherapy in treatment-naïve advanced-stage NSCLC (Peters et al 2017), combination with chemotherapy and bevacizumab in treatment-naive advanced-stage NSCLC (Sociniski et al 2018), monotherapy in advanced renal cell carcinoma (RCC) (McDermott et al 2016), monotherapy in metastatic castration-resistant prostate cancer (mCRPC; Kim et al 2018), combination with bevacizumab in treatment-naïve advanced RCC (Motzer et al 2018), monotherapy in

advanced triple-negative breast cancer (TNBC; Schmid et al 2017), monotherapy in advanced ovarian cancer (OC; Infante et al 2016), monotherapy in advanced endometrial cancer (EC; Fleming et al 2017), monotherapy and combination with bevacizumab in treatment-naïve hepatocellular carcinoma (HCC; Stein et al 2018; Roche data on file), monotherapy in advanced gastric cancer (GC; Taieb et al 2018), combination with bevacizumab (± chemotherapy) in advanced colorectal cancer (CRC; Hochster et al 2017), and monotherapy in advanced head and neck (H & N) cancer (Bahleda et al 2017). In addition, atezolizumab is currently being evaluated in combination with bevacizumab or molecular targeted therapies in anaplastic and differentiated thyroid cancer (NCT03181100).

Cabozantinib (XL184) is a potent inhibitor of multiple receptor tyrosine kinases (RTKs) known to play important roles in tumor cell proliferation and/or tumor neovascularization including MET, vascular endothelial growth factor receptor (VEGFR), AXL, and RET. Increased expression of MET and AXL has been implicated in the development of resistance to VEGFR inhibitors in preclinical models of several cancers (Shojaei et al 2010, Zhou et al 2016, Sennino et al 2012, Ciamporcero et al 2015). In addition, targets of cabozantinib are implicated in promoting tumor-immune suppression including TYRO3, MER, and AXL (tumor-assisted macrophage [TAM] family kinases).

Cabozantinib capsules (140 mg) have been approved in the US for the treatment of patients with progressive, metastatic medullary thyroid cancer (MTC) and in the EU for the treatment of patients with progressive, unresectable locally advanced or metastatic MTC (Cometriq® US PI and EMA SmPC). Cabozantinib tablets (60 mg) have been approved in the US, Europe, and other regions for advanced RCC (different patient populations depending on region; Cabometyx® US PI and EMA SmPC). Based on the results from a randomized placebo-controlled Phase 3 study (CELESTIAL) in subjects who had received prior sorafenib, cabozantinib tablets (60 mg) as a single agent have also been approved in the US, EU, and other regions for an HCC indication (Cabometyx US PI and EMA SmPC). Cabozantinib tablets (60 mg) have also been approved for the treatment of patients with radioactive iodine (RAI)-refractory differentiated thyroid cancer (DTC) in the US and are currently under review with EMA and Japan agencies. In addition, cabozantinib tablets (40 mg) have been approved in the United States, EU, and Japan for patients with advanced RCC, as a first-line treatment in combination with nivolumab.

Cabozantinib has also demonstrated encouraging clinical activity in other tumor indications: monotherapy in advanced urothelial carcinoma (Apolo et al [J Clin Oncol] 2016), in combination with ICIs in advanced urothelial carcinoma (Nadal et al 2018, Nadal et al 2017, Apolo et al [Ann Oncol] 2016), monotherapy in CRPC (Smith et al 2013, Smith et al 2014, Basch et al 2015), monotherapy or in combination with erlotinib in advanced NSCLC (Schöffski et al 2017, Neal et al 2016), monotherapy in RET-rearranged NSCLC (Drilon et al 2016), monotherapy in advanced TNBC (Tolaney et al 2017), monotherapy in advanced OC (Matulonis et al 2016, Vergote et al 2017), monotherapy in advanced EC (Dhani et al 2017; Mandilaras et al 2017), monotherapy in advanced GC (Schöffski et al 2017), in combination with panitumumab in CRC (Strickler et al 2016), and monotherapy in radioactive-iodine refractory DTC (Brose et al 2018, Cabanillas et al 2014, Cabanillas et al 2017).

Preclinical studies (Kwilas et al 2014, Song et al 2015, Lu et al 2017) and clinical observations on circulating immune suppressive cells and immune effector cells (Apolo et al 2014) suggest that cabozantinib promotes an immune-permissive environment through inhibition of immune-modulatory targets on immune cells. This might present an opportunity for synergistic effects from combination treatment with ICIs. The combination of cabozantinib with ICIs may also provide a strategy to overcome resistance to ICI therapy. This is based on recent observations in clinical trials where re-treatment with an ICI in combination with cabozantinib or a VEGFR-TKI that has a target profile similar to cabozantinib resulted in reversal of prior ICI resistance in advanced UC and NSCLC patients (Nadal et al 2018, Leal et al 2017). These results suggest that combining ICIs with cabozantinib may result in a tumor microenvironment that is conducive to re-sensitization to ICI therapy after prior progression on an ICI.

In this Phase 1b study, a total of 12 subjects with advanced RCC were enrolled in the Dose-Escalation Stage using a 3 + 3 design. Six (6) subjects were evaluated at both the 40-mg and 60-mg cabozantinib dose levels in combination with the standard dose of atezolizumab. Both dose levels of cabozantinib were generally well tolerated, and no dose-limiting toxicities (DLTs) were observed. After reviewing all available safety and efficacy data of the Dose-Escalation Stage, the Cohort Review Committee determined that cabozantinib 40 mg qd orally in combination with 1200 mg atezolizumab q3w IV is the recommended dose for the Expansion-Stage combination-therapy cohorts. The Cohort Review Committee decision was based on the favorable safety profile of the 40-mg cabozantinib dose level over a prolonged time on study treatment with less frequent dose reductions and encouraging preliminary efficacy, which was deemed to optimize the benefit/risk of the combination for the Expansion Cohorts.

The Expansion Stage is evaluating the efficacy and safety of cabozantinib 40 mg qd in combination with atezolizumab 1200 mg q3w across 20 tumor-specific cohorts of the following tumor types: RCC, UC, CRPC, NSCLC, triple negative breast cancer (TNBC), ovarian cancer (OC), endometrial cancer (EC), hepatocellular cancer (HCC), gastric cancer/gastroesophageal junction cancer/lower esophageal cancer (GC/GEJC/LEC), colorectal cancer (CRC), H&N cancer, and differentiated thyroid cancer (DTC). In order to establish the individual contributions of the components of the combination therapy, the Expansion Stage also includes three exploratory single-agent cabozantinib cohorts (UC, CRPC, and NSCLC) and one single-agent atezolizumab cohort (CRPC). The Study Oversight Committee (SOC) will review the efficacy and safety of the initially enrolled subjects (approximately 30 subjects in each cohort) and may recommend enrollment extension for up to 10 cohorts in which encouraging clinical activity has been demonstrated. The SOC can also recommend additional enrollment at a higher dose of cabozantinib (60 mg qd orally) in combination with 1200 mg atezolizumab q3w IV for tumor cohorts with modest clinical activity at cabozantinib 40 mg in combination with atezolizumab. The single-agent atezolizumab cohort will initially enroll 10 subjects. Enrollment with approximately 30 subjects will depend on the observed efficacy among the first 10 enrolled subjects. The Expansion Stage was initiated on 26 March 2018. At the time of this protocol amendment the SOC has recommended extended enrollment for Cohort 1 (clear cell RCC), Cohort 6 (CRPC), and Cohort 7 (NSCLC, prior ICI therapy) following review of efficacy and safety data of the initially enrolled subjects.

This study provides for subjects enrolling in the cohorts receiving single-agent treatment with either cabozantinib or atezolizumab the opportunity to receive combination treatment with cabozantinib and atezolizumab after Investigator-assessed radiographic disease progression per Response Evaluation Criteria in Solid Tumors (version 1.1) (RECIST 1.1) given these subjects meet the eligibility criteria for receiving combination treatment (Second Agent Add-On Stage).

XL184-021 Protocol Amendment 6.0 introduced considerations and study-related measures necessary due to the COVID-19 pandemic. XL184-021 Protocol Amendment 7.0 expanded the COVID-19-related guidance to include instructions for managing subjects who become infected on study, considerations for administration of COVID-19 vaccines, and confirmation that the COVID-19 accommodations are temporary and will be repealed back to standard study conduct when conditions allow.

OBJECTIVES

[redacted]

[redacted]

[redacted]

[redacted]

Expansion Stage (Combination-Therapy Cohorts):

The primary objective and endpoint is as follows:

- To evaluate preliminary efficacy of the combination therapy by estimating the ORR as assessed by the Investigator per RECIST 1.1

The secondary objective is as follows:

- To assess safety for the combination therapy through the evaluation of incidence and severity of nonserious AEs and SAEs, including irAEs and AESIs.

The exploratory objectives and endpoints are as follows:

- ORR as assessed by the Investigator per immune-related RECIST (irRECIST) for immune response
- Duration of response (DOR) as assessed by the Investigator per RECIST 1.1
- Progression-free survival (PFS) as assessed by the Investigator per RECIST 1.1
- ORR, DOR, and PFS as assessed by a Blinded Independent Radiology Committee (BIRC) per RECIST 1.1 for selected cohorts
- Overall survival (OS)
- Correlation of immune cell, tumor cell, and blood biomarker analyses with clinical outcome
- Changes in tumor infiltration and/or histology or other molecular changes as determined from optional tumor biopsy
- To further evaluate the plasma pharmacokinetics (PK) of daily oral administration of cabozantinib in subjects with solid tumors when given in combination with atezolizumab
- Tumor marker changes from baseline in select tumor indications
- Evaluation of mismatch repair (MMR) and microsatellite instability (MSI) status in relevant tumor indications
- [redacted]

[redacted]

- [redacted]
- [redacted]

[redacted]

- [redacted]
- [redacted]

STUDY DESIGN

This is a multicenter, open-label Phase 1b study to assess safety, tolerability, preliminary efficacy, and PK of cabozantinib alone or taken in combination with atezolizumab in subjects with advanced cancer.

The combination of cabozantinib with atezolizumab will be evaluated first in a Dose-Escalation Stage and subsequently in an Expansion Stage. The Dose-Escalation Stage allows enrollment of subjects with advanced RCC and UC using different dose levels of cabozantinib and a standard dose level of atezolizumab. The Expansion Stage allows enrollment in 20 different tumor cohorts including subjects with advanced RCC, UC, CRPC, NSCLC, TNBC, OC, EC, HCC, GC/GEJC/LEC, CRC, H&N cancer, and DTC to receive the recommended dose of the combination therapy from the Dose-Escalation Stage. In addition, the Expansion Stage includes three single-agent cabozantinib (60 mg) cohorts in UC, CRPC, and NSCLC as well as one single-agent atezolizumab (1200 mg) cohort in CRPC, which will evaluate the individual contributions of the components of the combination therapy.

Special accommodations during the global COVID-19 pandemic are described in Appendix M.

[redacted]

[redacted]

[redacted]

[redacted]

[redacted]

[redacted]

2. **Expansion Stage**: to further assess the efficacy, safety, PK, and pharmacodynamics of cabozantinib in combination with atezolizumab in multiple different tumor types using the recommended dose and schedule as determined by the Cohort Review Committee in the Dose-Escalation Stage. In addition, single-agent cohorts will enroll subjects to receive either cabozantinib only or atezolizumab only to evaluate the individual contributions of the components of the combination therapy.

   After initial enrollment of approximately 30 subjects, cohorts in the Expansion Stage [redacted] may extend enrollment (see Figure 1) based on the available clinical data per the SOC. The extended enrollment will be limited to up to 10 cohorts in the Expansion Stage with a maximum enrollment of 1000 additional subjects. [redacted] The Sponsor can decide to stop enrollment of any cohort at any time (eg, due to slow subject accrual).

   Combination-Therapy Expansion Cohorts:

   All Combination-Therapy Expansion Cohorts will initially enroll approximately 30 subjects with cabozantinib 40 mg in combination with atezolizumab 1200 mg as the assigned starting dose (see Table 1 for a summary of Expansion Cohorts 1-18 and 23-24).

   Because of the high unmet medical need of patients with advanced, incurable cancer, the SOC may decide after periodic review of safety and efficacy data of approximately 30 subjects of an Expansion Cohort to allow for additional enrollment to further assess the clinical activity and safety of the combination therapy. Extended

enrollment of a Combination-Therapy Expansion Cohort will be implemented based on only one of the following two Extended Enrollment Options (see Figure 1 below):

- Extended Enrollment Option 1:
    - Part I: Combination-Therapy Expansion Cohorts may enroll up to approximately 50 additional subjects to receive the same dosing regimen as the initially enrolled approximately 30 subjects (cabozantinib 40 mg + atezolizumab 1200 mg) upon approval by the SOC. Decisions by the SOC regarding additional enrollment will be based on the clinical significance of the achieved ORR in the Expansion Cohorts and will include an evaluation of the lower bound of confidence intervals for ORR in the initially enrolled subjects. A minimum observed ORR of around 20% or more will be used as a target (though not a requirement) for the SOC to consider Part I of Extended Enrollment Option 1 (Section 9.1.2). The magnitude of ORR deemed clinically meaningful by the SOC may vary by cohort. The committee may also consider other factors of clinical benefit (eg, time to response, duration of response, safety/tolerability) in the decision to extend enrollment. [redacted]
    - Part II: Combination-Therapy Expansion Cohorts may further enroll up to approximately 50 additional subjects for a maximum total of approximately 130 subjects in an expansion cohort. These subjects will receive the same dosing regimen as the previously enrolled subjects (cabozantinib 40 mg + atezolizumab 1200 mg) upon approval by the SOC. Decisions by the SOC regarding further extended enrollment in Part II of Extended Enrollment Option 1 will be based on the clinical significance of the achieved ORR in the Expansion Cohorts and will include an evaluation of the lower bound of confidence intervals for ORR in the previously enrolled subjects. A minimum observed ORR of around 35% or more will be used as a target (though not a requirement) for the SOC to consider Part II of the Extended Enrollment Option 1 (Section 9.1.2). Part II extension will only apply to tumor indications with a high-unmet medical need and very encouraging efficacy and safety data observed in Part I.
- Extended Enrollment Option 2: For Combination-Therapy Expansion Cohorts in which the initially enrolled approximately 30 subjects do not meet the criteria for Extended Enrollment Option 1, the SOC may decide to allow each selected Expansion Cohort to enroll up to approximately 30 additional subjects to receive the highest dose level of cabozantinib explored in the Dose-Escalation Stage (60 mg) in combination with atezolizumab 1200 mg to explore whether the higher cabozantinib dose will lead to improved clinical activity and maintain an acceptable safety profile.

Figure 1: Expansion Stage Enrollment Overview (Maximum 1720 Subjects)

Expansion-Stage Cohorts 1-24

Dose level: Combination Therapy: Cabozantinib 40 mg + Atezolizumab 1200 mg

[redacted]

Initial enrollment: n ≈ 30 subjects per cohor[redacted] (total up to 720 subjects)

↓

Study Oversight Committee (SOC) Review:

Safety and Efficacy of initially enrolled subjects in each cohort

↓

The SOC Approves Extended Enrollment for up to 10 Cohorts
in the Expansion Stage[redacted]
(maximum 1000 additional subjects)

Combination-Therapy Cohorts

Option 1-Part I:
Cabozantinib **40 mg** +
Atezolizumab 1200 mg
Enrollment: n ≈ ***50***[redacted]
subjects per cohort
Total ≈ 80 subjects

Option 2:
Cabozantinib **60 mg** +
Atezolizumab 1200 mg
Enrollment: n ≈ ***30***
subjects per cohort
Total ≈ 60 subjects

[redacted]

Option 1-Part II:

Cabozantinib **40 mg** +
Atezolizumab 1200 mg

Enrollment: n ≈ ***50***
subjects per cohort
Total ≈ 130 subjects

[redacted]

[redacted]

[redacted]

[redacted]

[redacted]

[redacted]

Table 1: Summary of All Combination-Therapy [redacted] Cohorts in the Expansion Stage

Cohort	Tumor Type (Histology)	Abbreviated Eligibility Description	Initial Cohort Size (n)
		Combination-Therapy Cohorts	
[redacted]	[redacted]	[redacted]	[redacted]
[redacted]	[redacted]	[redacted]	[redacted]
[redacted]	[redacted]	[redacted]	[redacted]
[redacted]	[redacted]	[redacted]	[redacted]
[redacted]	[redacted]	[redacted]	[redacted]
[redacted]	[redacted]	[redacted]	[redacted]
14	HCC (adeno)	No prior systemic anticancer therapy	30
15	GC/GEJC/LEC (adeno)	Prior platinum- or fluoropyridine-containing therapy	30
[redacted]	[redacted]	[redacted]	[redacted]
[redacted]	[redacted]	[redacted]	[redacted]
[redacted]	[redacted]	[redacted]	[redacted]
[redacted]	[redacted]	[redacted]	[redacted]
[redacted]	[redacted]	[redacted]	[redacted]
		[redacted]	
[redacted]	[redacted]	[redacted]	[redacted]
[redacted]	[redacted]	[redacted]	[redacted]
[redacted]	[redacted]	[redacted]	[redacted]
[redacted]	[redacted]	[redacted]	[redacted]
[redacted]	[redacted]	[redacted]	[redacted]
[redacted]	[redacted]	[redacted]	[redacted]

[redacted]

[redacted]

Treatment Periods:

Each subject's course of treatment will consist of the following periods:

Pre-Treatment Period: Potential subjects will be screened to determine if they meet the required eligibility criteria. Qualifying screening assessments must be performed within 28 days before first dose of study treatment unless otherwise specified.

Treatment Period: Eligible subjects will receive open-label cabozantinib (20, 40, or 60 mg orally administered qd). The date of the first dose of cabozantinib will be defined to be C1D1. Atezolizumab (1200 mg infusion) will be administered once every three weeks (-2 days) on Day 1 of each cycle starting on C1D1 for subjects in combination therapy cohorts [redacted]

Permitted study drug modifications to manage AEs comprise dose reductions (from 60 mg to 40 mg qd, from 40 mg to 20 mg qd, or from 20 mg qd to 20 mg every other day [qod]) or interruptions for cabozantinib and dose delays for atezolizumab.

Subjects will receive study treatment as long as they continue to experience clinical benefit in the opinion of the investigator or until there is unacceptable toxicity, the need for subsequent systemic anticancer treatment, or until any other reasons for treatment discontinuation listed in the protocol. Treatment may continue after radiographic progression as long as the Investigator believes that the subject is still receiving clinical benefit from study treatment and that the potential benefit of continuing study treatment outweighs potential risk. Following Sponsor notification, subjects in combination treatment cohorts may be allowed to discontinue one component of study treatment but

continue to receive the other. For cohorts where the initial dose is 40 mg cabozantinib, intra-subject dose escalation of cabozantinib from 40 mg to 60 mg is allowed after Sponsor approval for subjects who are tolerating the 40 mg cabozantinib dose level well and have been treated on this dose level for at least 4 weeks. [redacted]

Post-Treatment Period: The final safety assessment will occur at the Post-Treatment Follow-Up Visit 30 (+14) days after the date of the decision to discontinue treatment. If a subject is experiencing an ongoing treatment-related AE that led to study treatment discontinuation, SAE, or AESI at the time of that visit, the subject will continue to be followed until the AE has resolved, the AE has improved to Grade 2 or lower, or the Investigator determines that the event has become stable or irreversible.

Maintenance Phase: The purpose of the Maintenance Phase is to continue to provide long-term access to study drug(s) to subjects who are deriving clinical benefit even after evaluation of the study objectives has been completed. When sufficient data have been collected to adequately evaluate all study endpoints, and upon site notification by the Sponsor, subjects remaining on study treatment or who have not completed the Post-Treatment Follow-Up Visit will enter the study Maintenance Phase. In the Maintenance Phase subjects who remain on treatment will continue to receive study treatment until a protocol-defined criterion for discontinuation has been met. Following Sponsor notification, subjects in combination treatment cohorts may be allowed to discontinue one component of study treatment but continue to receive the other.

In the Maintenance Phase, subjects are to undergo periodic safety assessments (including local laboratory tests) and tumor assessments; the nature and frequency of these assessments are to be performed per standard of care if allowed per local regulations. In order to continue to collect important safety information on subjects still enrolled in the study, reporting of SAEs; AEs (including irAEs), whether serious or not, leading to dose modifications or treatment discontinuation; AESIs; and other reportable events (pregnancy and medication errors with sequelae) is to continue per protocol requirements specific to the Maintenance Phase.

Assessments in the Post-Treatment Period (including the Post-Treatment Follow-Up Visit) are not required for subjects who discontinue study treatment in the Maintenance Phase (such subjects are to be followed per standard of care).

Only data collected prior to implementation of Maintenance Phase will be reported in a clinical study report.

Study Completion by Country or by Site: After sufficient data have been collected to adequately evaluate all study endpoints and upon site notification by the Sponsor, the study will be considered complete at sites and in countries that no longer have active subjects.

NUMBER OF SUBJECTS

This study may enroll up to 1732 subjects with advanced solid tumors.

[redacted]

In the Expansion Stage approximately 720 subjects may be treated with either combination therapy or single-agent therapy across 24 tumor-specific cohorts with each initially enrolling approximately 30 subjects [redacted] Enrollment may be further extended in up to 10 Cohorts with up to an additional approximately 100 subjects each in the Combination-Therapy Cohorts [redacted]

[redacted]

TARGET POPULATION

To be eligible for the study the subject must meet all of the inclusion and none of the exclusion criteria. The Sponsor will not grant exceptions to these eligibility criteria.

Of note, in the eligibility criteria described below, maintenance anticancer therapy after the initial anticancer therapy does not count towards the limit of prior systemic therapies, provided there is no tumor progression between the initial anticancer therapy and the start of maintenance anticancer therapy. In addition, radiosensitization chemotherapy and retreatment with the same anticancer agent do not count towards the limit of prior systemic therapies.

[redacted]

Inclusion Criteria

1. Cytologically or histologically and radiologically confirmed solid tumor that is inoperable locally advanced, metastatic, or recurrent:

   [redacted]

   [redacted]

   [redacted]

   Expansion Stage:

   [redacted]

   [redacted]

[redacted]

[redacted]

[redacted]

[redacted]

[redacted] [redacted]

[redacted]

[redacted]

[redacted]

[redacted]

[redacted]

[redacted]

[redacted]

[redacted]

[redacted] [redacted]

[redacted]

[redacted]

[redacted]

[redacted]

p. Expansion Cohort 14: Subjects with advanced HCC who have a Child-Pugh score of A (Appendix L) and have not received prior systemic anticancer therapy for inoperable locally advanced, recurrent, or metastatic disease.

- *Subjects with active hepatitis B virus (HBV) infection (defined by HBsAg positive) must be on standard of care antiviral therapy and have HBV DNA < 500 IU/mL.*
- *Prior local-regional treatment (eg, radiofrequency ablation, transcatheter arterial chemoembolization [TACE]) is allowed.*

q. Expansion Cohort 15: Subjects with gastric cancer, gastroesophageal junction adenocarcinoma, or lower one-third esophageal adenocarcinoma who have radiographically progressed during or following platinum-containing or fluoropyrimidine-containing chemotherapy for inoperable locally advanced, recurrent, or metastatic disease.

- *Allowed are up to 2 lines of prior systemic anticancer therapy for inoperable locally advanced, recurrent, or metastatic disease.*
- *Prior HER-2/neu directed therapy is allowed.*

[redacted]

- [redacted]
- [redacted]
- [redacted]

[redacted]

- [redacted]
- [redacted]
- [redacted]
- [redacted]
- [redacted]

[redacted]

- [redacted]
- [redacted]

[redacted]

- [redacted]
- [redacted]

[redacted]

- [redacted]
- [redacted]
- [redacted]

[redacted]

- [redacted]

  [redacted]

- [redacted]
- [redacted]

  [redacted]

  [redacted]

- [redacted]

  [redacted]

  [redacted]

[redacted]

- [redacted]
- [redacted]

[redacted]

2. Measurable disease per RECIST 1.1 as determined by the Investigator. Measurable disease must be outside the radiation field if prior radiation therapy was administered.
3. Tumor tissue material available (archival or recent tumor biopsy).

[redacted]

4. Recovery to baseline or ≤ Grade 1 CTCAE v4 from toxicities related to any prior treatments, unless AE(s) are clinically nonsignificant and/or stable on supportive therapy.
5. Age eighteen years or older on the day of consent.
6. Eastern Cooperative Oncology Group (ECOG) Performance Status of 0 or 1.
7. Adequate organ and marrow function, based upon meeting all of the following laboratory criteria within 14 days before first dose of study treatment:
   a. Absolute neutrophil count (ANC) ≥ 1500/µL (≥ 1.5 × 10^9/L) without granulocyte colony-stimulating factor support within 2 weeks before screening laboratory sample collection.
   b. White blood cell count ≥ 2500/µL (≥ 2.5 × 10^9/L).
   c. Platelets ≥ 100,000/µL (≥ 100 × 10^9/L) without transfusion within 2 weeks before screening laboratory sample collection. For subjects with HCC ≥ 75,000/µL (≥ 75 × 10^9/L).
   d. Hemoglobin ≥ 9 g/dL (≥ 90 g/L) without transfusion within 2 weeks before screening laboratory sample collection.
   e. Alanine aminotransferase (ALT), aspartate aminotransferase (AST), and alkaline phosphatase (ALP) ≤ 3 × upper limit of normal (ULN). ALP ≤ 5 × ULN with documented bone metastases. For subjects with HCC: ALT, AST, and ALP ≤ 5 × ULN [redacted]

f. Total bilirubin ≤ 1.5 × ULN (for subjects with Gilbert's disease ≤ 3 × ULN). For subjects with HCC ≤ 2 mg/dL (≤ 34.2 μmol/L).

g. Serum creatinine ≤ 1.5 × ULN or calculated creatinine clearance ≥ 40 mL/min (≥ 0.67 mL/sec) using the Cockcroft-Gault equation (see Table 5-2 for Cockcroft-Gault formula).

h. Urine protein/creatinine ratio (UPCR) ≤ 1 mg/mg (≤ 113.2 mg/mmol). [redacted]

8. Capable of understanding and complying with the protocol requirements and must have signed the informed consent document.

9. Sexually active fertile subjects and their partners must agree to use highly effective methods of contraception that alone or in combination result in a failure rate of less than 1% per year when used consistently and correctly (defined in Appendix K) during the course of the study and for 5 months after the last dose of study treatment. An additional contraceptive method, such as a barrier method (eg, condom), is recommended.

10. Female subjects of childbearing potential must not be pregnant at screening. Female subjects are considered to be of childbearing potential unless one of the following criteria is met: permanent sterilization (hysterectomy, bilateral salpingectomy, or bilateral oophorectomy) or documented postmenopausal status (defined as 12 months of amenorrhea in a woman over 45 years-of-age in the absence of other biological or physiological causes. In addition, females under 55 years-of-age must have a serum follicle stimulating hormone (FSH) level > 40 mIU/mL to confirm menopause). Note: Documentation may include review of medical records, medical examination, or medical history interview by study site staff.

Exclusion Criteria

1. Prior treatment with cabozantinib or ICIs including anti-CTLA-4, anti-PD-1, anti-PD-L1, anti-PD-L2, anti-OX-40, anti-CD137 therapy [redacted]
2. Receipt of any type of small molecule kinase inhibitor (including investigational kinase inhibitor) within 2 weeks before first dose of study treatment. [redacted]
3. [redacted]
4. HCC subjects who meet any of the following criteria are ineligible:
   a. Received prior local anticancer therapy (including embolization and ablation) within 4 weeks before first dose of study treatment. For prior radiation for bone metastases, refer to Exclusion Criteria 6.
   b. Subjects with fibrolamellar HCC, sarcomatoid HCC, or mixed cholangiocarcinoma.
5. Receipt of any type of anticancer antibody (including investigational antibody) or systemic chemotherapy within 4 weeks before first dose of study treatment [redacted]
6. Radiation therapy for bone metastasis within 2 weeks, any other local radiation therapy within 4 weeks before first dose of study treatment. Subjects who have received systemic treatment with radionuclides within 6 weeks before first dose of study treatment are not eligible. Subjects with clinically relevant ongoing complications from prior radiation therapy are not eligible.
7. Known brain metastases or cranial epidural disease unless adequately treated with radiotherapy and/or surgery (including radiosurgery) and stable for at least 4 weeks before first dose of study treatment. Eligible subjects must be neurologically asymptomatic and without corticosteroid treatment at the time of first dose of study treatment.
8. Concomitant anticoagulation with oral anticoagulants except for those specified below.
   a. Allowed anticoagulants are:
      i. Prophylactic use of low-dose aspirin for cardioprotection (per local applicable guidelines) and low-dose low molecular weight heparins (LMWH)
      ii. Therapeutic doses of LMWH or specified direct factor Xa inhibitors rivaroxaban, edoxaban, or apixaban in subjects (excluding HCC subjects) without known brain metastases who are on a stable dose of the anticoagulant for at least 1 week before first dose of study treatment and without clinically significant hemorrhagic complications from the anticoagulation regimen or the tumor.

      Note: Subjects with HCC may be treated with therapeutic LMWH but must have a screening platelet count > 100,000/µL. Direct inhibitors of thrombin or factor Xa are not permitted in subjects with HCC.
9. Diagnosis of immunodeficiency or is receiving systemic steroid therapy (> 10 mg daily prednisone equivalent) or any other form of immunosuppressive therapy within 2 weeks prior to first dose of study treatment. Inhaled,

intranasal, intraarticular, and topical corticosteroids and mineralocorticoids are allowed.
Note: Adrenal replacement steroid doses > 10 mg daily prednisone equivalent are permitted in the absence of active autoimmune disease. Transient short-term use of systemic corticosteroids for allergic conditions (eg, contrast allergy) is also allowed.

10. Administration of a live, attenuated vaccine within 30 days before first dose of study treatment.
11. The subject has uncontrolled, significant intercurrent or recent illness including, but not limited to, the following conditions:
    a. Cardiovascular disorders:
        i. Congestive heart failure New York Heart Association Class 3 or 4, unstable angina pectoris, serious cardiac arrhythmias.
        ii. Uncontrolled hypertension defined as sustained blood pressure (BP) > 140 mm Hg systolic or > 90 mm Hg diastolic despite optimal antihypertensive treatment.
        iii. Stroke (including transient ischemic attack [TIA]), myocardial infarction (MI), or other ischemic event, or thromboembolic event (eg, deep venous thrombosis [DVT], pulmonary embolism [PE]) within 6 months before first dose. Upon Sponsor approval, subjects with a diagnosis of incidental, subsegmental PE or DVT within 6 months are allowed if stable, asymptomatic, and treated with anticoagulation for at least 1 week before first dose. Iatrogenic arterial embolization procedures such as tumor arterial embolization or splenic artery embolization are allowed.
    b. Gastrointestinal (GI) disorders including those associated with a high risk of perforation or fistula formation:
        i. Tumors invading the GI-tract, active peptic ulcer disease, inflammatory bowel disease, diverticulitis, cholecystitis, symptomatic cholangitis or appendicitis, acute pancreatitis or acute obstruction of the pancreatic or biliary duct, or gastric outlet obstruction. Presence of primary GI tumor is not excluded.
        ii. Abdominal fistula, GI perforation, bowel obstruction, or intra-abdominal abscess within 6 months before first dose. Note: Complete healing of an intra-abdominal abscess must be confirmed before first dose.
        iii. Gastric or esophageal varices that are untreated or incompletely treated with bleeding or high risk for bleeding. Subjects treated with adequate endoscopic therapy (according to institutional standards) without any episodes of recurrent GI bleeding requiring transfusion or hospitalization for at least 6 months prior to study entry are eligible.
    c. Clinically significant hematuria, hematemesis, or hemoptysis of > 0.5 teaspoon (2.5 mL) of red blood, or other history of significant bleeding (eg, pulmonary hemorrhage) within 12 weeks before first dose.
    d. Cavitating pulmonary lesion(s) or known endobronchial disease manifestation.
    e. Lesion invading a major blood vessel including, but not limited to, inferior vena cava, pulmonary artery, or aorta. HCC subjects with lesions invading the hepatic portal vasculature are eligible.
    f. Other clinically significant disorders such as:
        i. Active or history of autoimmune disease or immune deficiency, including, but not limited to, myasthenia gravis, myositis, autoimmune hepatitis, systemic lupus erythematosus, rheumatoid arthritis, psoriatic arthritis, inflammatory bowel disease, antiphospholipid antibody syndrome, Wegener

granulomatosis, Sjögren's syndrome, Guillain-Barré syndrome, or multiple sclerosis (see Appendix E for a more comprehensive list of autoimmune diseases and immune deficiencies). Subjects with the following conditions are eligible for the study:

- A history of autoimmune-related hypothyroidism and on thyroid replacement hormone therapy

  Note: Subjects with prior history of thyroiditis are allowed if they have undergone sub-total, near-total, or total thyroidectomy.

- Controlled Type 1 diabetes mellitus and on an insulin regimen
- Asthma
- Eczema, psoriasis, lichen simplex chronicus, or vitiligo with dermatologic manifestations only provided all of following are true:
  - Rash covers < 10% of body surface area
  - Disease is well controlled at baseline and requires only low-potency topical corticosteroids
  - No occurrence of acute exacerbations of the underlying condition requiring psoralen plus ultraviolet A radiation, methotrexate, retinoids, biologic agents, oral calcineurin inhibitors, or high potency or oral corticosteroids within the previous 12 months

ii. Active infection requiring systemic treatment, infection with human immunodeficiency virus (HIV) or acquired immunodeficiency syndrome (AIDS)-related illness, acute or chronic hepatitis B or C infection in non-HCC tumor cohorts, or a known positive test for tuberculosis infection if supported by clinical or radiographic evidence of disease. Subjects with history of COVID-19 must have recovered from the disease at least 30 days prior to enrollment.

iii. History of idiopathic pulmonary fibrosis, organizing pneumonia (eg, bronchiolitis obliterans), drug-induced pneumonitis, idiopathic pneumonitis, or evidence of active pneumonitis on screening chest computerized tomography (CT) scan. History of radiation pneumonitis in the radiation field (fibrosis) is permitted.

iv. Serious non-healing wound/ulcer/bone fracture.

v. Malabsorption syndrome.

vi. For all subjects [redacted]: Free thyroxine (FT4) outside the laboratory normal reference range. Asymptomatic subjects with FT4 abnormalities can be eligible after sponsor approval.

vii. Moderate to severe hepatic impairment for subjects with chronic liver disease (Child-Pugh B or C; Appendix L).

viii. Requirement for hemodialysis or peritoneal dialysis.

ix. History of solid organ or allogenic stem cell transplant.

12. Major surgery (eg, GI surgery, removal or biopsy of brain metastasis) within 4 weeks or minor surgery (eg, simple excision, tooth extraction) within 10 days before first dose of study treatment. Complete wound healing from surgery must have occurred before first dose. Subjects with clinically relevant ongoing complications from prior surgery are not eligible.

13. Corrected QT interval calculated by the Fridericia formula (QTcF) > 500 ms per electrocardiogram (ECG) within 14 days before first dose of study treatment (see Section 5.6.4 for Fridericia formula).

Note: If a single ECG shows a QTcF with an absolute value > 500 ms, two additional ECGs at intervals of approximately 3 min must be performed within 30 min after the initial ECG, and the average of these three consecutive results for QTcF will be used to determine eligibility (ie, if the average is ≤ 500 ms the subject is eligible).

14. Pregnant or lactating females.
15. Inability to swallow tablets.
16. Previously identified allergy or hypersensitivity to components of the study treatment formulations. *Subjects with a history of infusion-related reaction to prior therapy with atezolizumab may be eligible by sponsor approval if the reaction was considered mild and manageable with appropriate supportive care (eg, use of premedication according to standard of care).*
17. Diagnosis of another malignancy within 2 years before first dose of study treatment, except for superficial skin cancers, or localized, low grade tumors deemed cured and not treated with systemic therapy. Incidentally diagnosed prostate cancer is allowed if assessed as stage ≤ T2N0M0 and Gleason score ≤ 6.

ESTIMATED LENGTH OF SUBJECT PARTICIPATION

It is estimated that subjects with advanced previously treated GC/GEJC/LEC may receive study treatment for an average of 4 months, subjects with [redacted] untreated HCC may receive study treatment for an average of 6 months, [redacted] Subjects will however be followed until death, withdrawal of consent, or Sponsor decision to no longer collect survival data.

ESTIMATED STUDY DURATION

[redacted] It is estimated that approximately 24 months will be required to enroll the 20 Combination-Therapy Expansion Cohorts, [redacted] The true intervals required to meet the milestones above may be longer or shorter due to the impact of the global COVID-19 pandemic on subject enrollment and other aspects of study conduct.

INVESTIGATIONAL REGIMEN DOSE/ROUTE/INTERVAL

Cabozantinib will be supplied as 60-mg and 20-mg tablets (expressed as freebase weight). [redacted]

Atezolizumab will be administered at a standard dosing regimen of 1200 mg as an IV infusion once every 3 weeks (q3w). The initial infusion of atezolizumab will be given over 60 (± 15) minutes without premedication for potential IRRs or CRS. Subsequent IV infusions may be given over 30 (± 10) minutes if the initial infusion is tolerated. Premedication for infusion-reaction is allowed after the initial infusion. No bolus or IV push of atezolizumab is allowed.

[redacted]

[redacted] Prior to Protocol Amendment 2.0 the recommended dose for the Expansion stage had already been identified (combination treatment of 40 mg cabozantinib qd orally plus

1200 mg atezolizumab q3w IV) during the Escalation Stage using the Standard Dosing Schedule. The Cabozantinib Run-In Dosing Schedule will therefore not be implemented in the study.

In the Expansion Stage, all initially enrolled subjects in the Combination-Therapy Cohorts and any additional subjects enrolled per Extended Enrollment Option 1 will receive cabozantinib 40 mg in combination with the standard dose of atezolizumab. Additional subjects enrolled per Extended Enrollment Option 2 will receive cabozantinib 60 mg in combination with the standard dose of atezolizumab. For details on the Extended Enrollment Options, see Section 3.5.2.1. In addition, intra-subject dose escalation of cabozantinib from 40 mg to 60 mg is allowed after Sponsor approval for subjects enrolled at the 40-mg cabozantinib dose level who are tolerating the 40 mg cabozantinib dose level well and have been treated on this dose level for at least 4 weeks.

[redacted]

Refer to Section 6.5.1 for management guidance on dose reductions and/or dose interruptions due to an AE in the Dose-Escalation and Expansion Stages.

SAFETY ASSESSMENTS

Safety evaluations will include assessments of AEs (including irAEs and AESIs), vital signs, ECGs, laboratory tests, and concomitant medications. Adverse event seriousness, severity grade, relationship to study treatment, and relationship to immune effects (ie, irAEs) will be assessed by the investigator. Severity grade will be defined by the NCI CTCAE version 4.

TUMOR ASSESSMENTS

Tumor response will be assessed using RECIST 1.1 (Appendix G). Additional exploratory efficacy evaluation will include the application of irRECIST for immune response (Appendix H). Subjects will be assessed using a magnetic resonance imaging (MRI) or a CT scan from the date of the first dose of study treatment until the later of radiographic disease progression per RECIST 1.1 as determined by the investigator or the date of the decision to permanently discontinue study treatment. Radiographic tumor assessments will continue on the protocol-defined schedule, regardless of whether study treatment is reduced, interrupted, delayed, or discontinued.

<u>Chest / Abdomen / Pelvis/ Neck</u>: Unless otherwise described, CT of Chest/Abdomen/Pelvis (CAP) or CT chest and MRI abdomen/pelvis will be performed in all subjects at screening and every 6 weeks (± 5 days) after initiation of study treatment throughout the first 12 months on study. Upon completion of 12 months on study, these assessments will be performed every 12 weeks (± 7 days). [redacted] Low dose non-contrast CT images from combined positron emission tomography/computed tomography (PET/CT) imaging cannot be used for tumor evaluations in this study.

Brain: MRI (or CT) of the brain will be performed at screening [redacted] for subjects with the other tumor indications who have a history or clinical symptoms of brain metastasis. After study treatment initiation MRI (or CT) scans of the brain are only required in subjects with documented, treated brain metastasis or if clinically indicated by signs and symptoms suggestive of new central nervous system (CNS) metastases. Assessments after the first dose of study treatment will be performed every 12 weeks (± 7 days). MRI is the preferred imaging method for brain. If CT of the brain is performed instead of MRI, ambiguous results must be confirmed by MRI unless contraindicated. Subjects without documented brain metastasis during the screening assessment are not required to undergo brain imaging after initiating study treatment unless clinically indicated. In order to meet the eligibility requirements of the study, brain metastasis must have been treated and stable for at least 4 weeks before first dose of study treatment.

Bone scans: Technetium bone scans (TBS) will be performed at screening [redacted] for subjects with the other tumor indications who have a history or clinical symptoms (ie, bone pain) of bone metastases. After study treatment initiation bone scans are only required in subjects with documented bone lesions or if clinically indicated by signs and symptoms suggestive of new bone metastases. Assessments after the first dose will follow routine clinical practice (approximately every 12 weeks throughout the first 12 months and every 24 weeks thereafter). Lesions identified on bone scan are not to be recorded as target, non-target, or new lesions. Bone scan findings alone cannot be used for the determination of progression or response in this study and need to be corroborated by CT/MRI. Bone lesions corroborated by CT/MRI must be reported as non-target or new lesions. PET scan or plain films are not considered adequate imaging techniques to measure bone lesions.

[redacted]

For the purpose of determining radiographic study endpoints for selected cohorts, central review of radiographic images may be conducted by a BIRC. All protocol-required radiographic tumor assessments for these selected cohorts will be sent to the BIRC, which also will review prior radiation history data and prior local therapy information for the purpose of selection of target lesions. Details are provided in the Imaging Manual.

TUMOR MARKER ASSESSMENTS

For subjects with [redacted] HCC, [redacted] tumor marker samples (ie, [redacted] alpha-feta protein [AFP], [redacted]) will be collected at screening, Day 1 of every third cycle (or every 9 weeks, whichever is earlier) for the first 12 months on study, and then Day 1 of every fifth cycle (or every 15 weeks, whichever is earlier) until the earlier of initiation of subsequent systemic anticancer therapy or permanent loss to radiographic follow-up (including hospice admission). [redacted] The tumor marker assessments will not be used to determine progressive disease or to make study treatment decisions in this study.

OVERALL SURVIVAL FOLLOW-UP ASSESSMENTS

Subjects will be contacted (eg, in person or by telephone) approximately every 12 weeks (± 14 days) after the Post-Treatment Follow-Up Visit to assess survival status and to document receipt of subsequent anticancer therapy unless consent to participate in survival follow-up is withdrawn or the Sponsor deems sufficient efficacy data have been collected for the study.

PHARMACOKINETIC ASSESSMENTS

[redacted]

Expansion Stage:

Combination Therapy Expansion Cohorts:

Blood samples for PK analysis will be obtained for plasma cabozantinib and serum atezolizumab concentration measurement on the date of first dose of study treatment (C1D1; prior to study treatment administration [cabozantinib and atezolizumab], approximately 5 min after completion of the atezolizumab infusion, and 2 h after the first dose of cabozantinib) and prior to study treatment dosing (atezolizumab infusion) on C2D1 and C3D1.

[redacted]

[redacted]

[redacted] for the Combination-Therapy Cohorts in the Expansion Stage, samples will be analyzed for the plasma concentration of cabozantinib and the serum concentrations of atezolizumab. [redacted]

IMMUNOGENICITY ASSESSMENTS

Blood samples will be obtained from all subjects in the combination treatment cohorts [redacted] for immunogenicity assessment predose on C1D1, C3D1, C7D1, and at the Post-Treatment Follow-up Visit.

[redacted]

[redacted]

BIOMARKER ASSESSMENTS

Peripheral blood and tumor tissue will be collected and may be assessed for exploratory biomarker analyses. Peripheral blood samples will be obtained as specified in the Schedule of Assessments. Tumor tissue (archival) will be obtained prior to first dose of study treatment, and optional fresh tumor tissue biopsies may also be performed. Exploratory analyses may include the following:

- MET, AXL, and PD-L1 in tumor specimens for association with clinical outcomes
- Immune cell infiltration and tumor characteristics (ie, mutational load assessment) in tumor specimens and blood for association with clinical outcome
- Circulating immune cells in peripheral blood (ie, lymphocyte subset analyses by flow cytometry)
- Blood biomarkers (ie, cytokines/chemokines, VEGF)
- Evaluation of MMR and MSI status

Collection of biomarker samples may be halted early or sampling frequency may be modified at the discretion of the Sponsor.

[redacted]

STATISTICAL METHODS

Combination Therapy Cohorts:

[redacted]

Expansion Stage:

Combination-Therapy Expansion Cohorts

Objective Response Rate: The objective of the Expansion Stage is to estimate ORR, defined as the proportion of subjects with a confirmed complete response (CR) or partial response (PR) per RECIST 1.1 as determined by the investigator. ORR will be evaluated independently within each of the Combination-Therapy Expansion Cohorts.

The primary purpose of estimating ORR in the Combination-Therapy Cohorts in the Expansion Stage is to assess if the true response rate with this combination regimen is better than that expected with monotherapy. Thus, 2-sided

80% and 60% Blyth-Still-Casella confidence intervals (CIs) will be constructed for ORR, providing 90% and 80% 1-sided confidence, respectively, when interpreting the lower bound. The sample size of 30 subjects for each of the Expansion Cohorts was chosen to ensure the lower bound of the 2-sided 80% CI extended no more than 12 percentage points from the point estimate.

Combination-Therapy Expansion Cohorts may enroll additional subjects beyond the initial subjects per Extended Enrollment Option 1 or 2 (described below). It is anticipated that not all Expansion Cohorts will open for additional enrollment. Note: Up to 10 cohorts [redacted] may be expanded in the Expansion Stage; [redacted]

Extended Enrollment Option 1: Should the SOC deem that a clinically meaningful ORR has been observed in an Expansion Cohort, approximately 100 new subjects may be added to that cohort to further investigate the safety and clinical benefit of the combination in that treatment setting.

Decisions by the SOC regarding the clinical significance of the achieved ORR in Expansion Cohorts will include an evaluation of the lower bound of confidence intervals for ORR in the initially enrolled approximately 30 subjects, and the expansion cohorts will be extended as follows:

Extension-Part I: Approximately 50 additional subjects will be added in this extension part for a total of approximately 80 subjects in an expansion cohort. The observed ORR in the previously enrolled subjects will be considered. A minimum observed ORR of around 20% or more will be used as a target (though not a requirement) for the SOC to consider cohort expansion. This corresponds to 80% confidence that the true ORR is ≥ 13% for n = 30 (≥ 11% for n = 15). The magnitude of ORR deemed clinically meaningful by the SOC may vary by cohort, and the committee may consider other factors of clinical benefit (eg, time to response, duration of response, safety/tolerability) in the decision to extend enrollment. [redacted]

Extension-Part II: Approximately 50 additional subjects will be added in this second extension part for a maximum total of approximately 130 subjects in an expansion cohort. The observed ORR for the previously enrolled subjects (approximately 30 subjects initially enrolled + approximately 50 subjects enrolled in Extension-Part I) will be considered. A minimum observed ORR of around 35% or more will be used as a target for the SOC to consider additional cohort expansion. This corresponds to 90% confidence that the true ORR is ≥ 28% for n = 80. Part II extension will only apply to tumor indications with a high-unmet medical need and very encouraging efficacy and safety data observed in Part I.

A total sample size of 130 subjects was selected to ensure the lower bound of the 95% confidence interval for ORR will extend less than 10% points from the point estimate if Part II is implemented.

Extended Enrollment Option 2: For Expansion Cohorts in which the initially enrolled approximately 30 subjects do not meet the criteria for Extended Enrollment Option 1, the SOC may decide to allow each selected Expansion Cohort to enroll approximately 30 new subjects to receive the highest dose level of cabozantinib explored in the Dose-Escalation Stage (60 mg) in combination with atezolizumab 1200 mg to explore whether the higher cabozantinib dose will lead to improved clinical activity and maintain an acceptable safety profile.

PFS and OS: Median PFS and OS will be estimated using Kaplan-Meier methods.

DOR: DOR medians will be estimated using Kaplan-Meier analysis and will be limited to patients who experienced a confirmed objective response.

Safety: Summaries of AEs, irAEs, AESIs, and SAEs will be tabulated by cohort according to system organ class and preferred term by overall incidence; worst reported severity; and relationship to study treatment. Selected laboratory test results will be summarized by treatment group to evaluate worst post-baseline CTCAE grade and shifts or changes from baseline.

[redacted]

[redacted]

[redacted]

[redacted]

[redacted]

[redacted]

[redacted]

[redacted]

TABLE OF CONTENTS

CLINICAL STUDY PROTOCOL 1

PROTOCOL APPROVAL PAGE 2

PROTOCOL ACCEPTANCE FORM 3

SYNOPSIS 4

TABLE OF CONTENTS 35

LIST OF ABBREVIATIONS 43

1 BACKGROUND AND RATIONALE 48

1.1 Background 48

1.1.1 Atezolizumab 48

[redacted]

1.1.1.8 Clinical Experience in Hepatocellular Carcinoma 55

1.1.1.9 Clinical Experience in Gastric Cancer 56

[redacted]

1.2 Cabozantinib 58

1.2.1 Nonclinical Toxicology 59

[redacted]

1.2.9 Clinical Experience in Hepatocellular Carcinoma 66

1.2.10 Clinical Experience in Gastric Cancer 67

[redacted]
1.2.14 Immunological Effects ... 69
1.3 Rationale ... 70
1.3.1 Rationale for the Study and Study Design ... 70
1.3.2 Rationale for Dosage Selection and Treatment Schedule ... 83
1.4 Overall Risk Benefit Assessment ... 90

2 STUDY OBJECTIVES ... 93

3 STUDY DESIGN ... 95
3.1 Overview ... 95
3.2 Study Sites ... 95
3.3 Blinding and Randomization ... 95
3.4 Pretreatment Period ... 96
3.5 [redacted]
3.5.2 Expansion Stage (Combination-Therapy Cohorts, [redacted] ... 101
3.5.2.1 Combination-Therapy Expansion Cohorts ... 105
[redacted]
3.6 Post-Treatment Period and Survival Follow-up ... 109
3.7 Maintenance Phase ... 109
3.8 Treatment Discontinuation and Withdrawals ... 110
3.9 Subject Replacements ... 112

4 STUDY POPULATION ... 113
4.1 Target Population ... 113
4.2 Inclusion Criteria ... 113
4.3 Exclusion Criteria ... 121

5 STUDY ASSESSMENTS AND PROCEDURES ... 126
5.1 Pretreatment Period ... 127
5.2 Treatment Period ... 128

[redacted]
5.2.2 Expansion Stage 129
5.2.2.1 Combination-Therapy Cohorts 129
[redacted]
5.3 Post-Treatment Period 130
5.4 Maintenance Phase 131
5.5 Unscheduled Visits or Assessments 132
5.6 Procedure Details 132
5.6.1 Demographics, Medical and Cancer History 132
5.6.2 Physical Examination 133
5.6.3 Vital Signs 133
5.6.4 Electrocardiogram Assessments 133
5.6.5 Laboratory Assessments 134
5.6.6 Pharmacokinetic Assessments 137
5.6.6.1 Pharmacokinetic Blood Samples 138
5.6.7 Immunogenicity Assessments 139
5.6.8 Biomarker Assessments 139
5.6.9 Tumor Assessment 140
5.6.9.1 Routine Tumor Assessment 140
5.6.9.2 Confirmation of Tumor Response and Tumor Progression 144
5.6.9.3 Serum Testosterone Assessment 144
5.6.9.4 Tumor Marker Assessment 144
5.6.10 Subject Daily Dosing Diary 145
5.6.11 Overall Survival 145
6 TREATMENTS 146
6.1 Composition, Formulation, and Storage 146
6.1.1 Investigational Treatment: Cabozantinib 146
6.1.2 [redacted] Combination Treatment: Atezolizumab 146
6.2 Schedule of Treatment 147
6.2.1 Administration at the Clinic 148
6.2.2 Cabozantinib Administration outside the Clinic 149
6.3 Compliance 150
6.4 Study Treatment Accountability 150
6.5 Safety Considerations 150
6.5.1 Management of AEs with Dose Reductions and/or Dose Interruptions 150
6.5.2 Warnings, Precautions, Guidelines for Management of Adverse Events 159

6.5.2.1 Cabozantinib 159
6.5.2.2 Atezolizumab 174
6.5.2.3 Management Guidelines for Hepatic Encephalopathy 198

7 CONCOMITANT MEDICATIONS AND THERAPIES 199
7.1 Allowed Therapy 199
7.2 Prohibited or Restricted Therapy 202
7.3 Potential Drug Interactions 204
7.3.1 Potential Drug Interactions with Cabozantinib 204
7.3.2 Potential Drug Interactions with Atezolizumab 205

8 SAFETY 206
8.1 Adverse Events and Laboratory Abnormalities 206
8.1.1 Adverse Events 206
8.1.2 Laboratory Abnormalities 207
8.2 Serious Adverse Events 207
8.2.1 Regulatory Reporting 209
8.3 Adverse Events of Special Interest for Atezolizumab 210
8.3.1 General Information on Immune-Related Adverse Events 212
8.4 Follow-Up of Adverse Events 213
8.5 Other Safety Considerations 213
8.5.1 Pregnancy 213
8.5.2 Medication Errors/Overdose 214

9 STATISTICAL CONSIDERATIONS 215
9.1 Power and Sample Size 215
[redacted]
9.1.2 Expansion Stage 215
[redacted]
9.2 Analysis Populations 222
9.2.1 Safety Population 222
[redacted]
9.2.3 Other Population(s) 222
9.3 Planned Analyses 222
9.3.1 Safety and Tolerability Analyses 222
9.3.1.1 Adverse Events 222
9.3.1.2 Laboratory Test Results 223
9.3.1.3 Study Treatment 223

9.3.2 Analyses of Preliminary Antitumor Activity....223
9.3.3 Interim Analyses....224

10 OTHER ANALYSES....224
10.1 Pharmacokinetic Analyses....224
10.2 Immunogenicity Analyses....224
10.3 Biomarker Analyses....224

11 DATA QUALITY ASSURANCE....225

12 STUDY COMMITTEES....225
12.1 Cohort Review Committee....225
12.2 Study Oversight Committee....225
12.3 Corporate Safety Governance....225
12.4 Blinded Independent Radiology Committee (BIRC)....226

13 ETHICAL ASPECTS....226
13.1 Local Regulations....226
13.2 Informed Consent....226
13.3 Institutional Review Board/ Ethics Committee....227
13.4 Disposition of Subject Samples....227

14 CONDITIONS FOR MODIFYING THE PROTOCOL....227

15 CONDITIONS FOR TERMINATING THE STUDY....228

16 STUDY DOCUMENTATION, CASE REPORT FORMS, AND RECORD KEEPING....229
16.1 Investigator's Files and Retention of Documents....229
16.2 Source Documents and Background Data....230
16.3 Audits and Inspections....230
16.4 Case Report Forms....230

17 MONITORING THE STUDY....231

18 CONFIDENTIALITY OF TRIAL DOCUMENTS AND SUBJECT RECORDS 232

19 PUBLICATION OF DATA AND PROTECTION OF TRADE SECRETS....232

20 COMPLIANCE WITH DATA PROTECTION LAWS....233

21 REFERENCES....234

LIST OF TABLES

Table 1-1: Overall Safety Overview as of 21 May 2019 (Dose-Escalation Stage and Expansion Stage) 87
Table 1-2: Summary of Frequent Adverse Events Regardless of Causality (≥ 10%) as of 21 May 2019 (Dose-Escalation Stage and Expansion Stage) 88
Table 1-3: Summary of Immune-Related Adverse Events (≥ 1%) by Preferred Term and Severity as of 21 May 2019 (Dose-Escalation Stage and Expansion Stage) 89
Table 3-1: Dosing Combinations for Potential Evaluation during the Dose-Escalation Stage 97
Table 3-2: Dose-Escalation Stage Decision Rules 98
Table 3-3: Summary of All Combination-Therapy and Single-Agent Cohorts in the Expansion Stage 103
Table 5-1: Clinical Laboratory Panels 136
Table 5-2: Estimation of the Creatinine Clearance by Cockcroft and Gault 137
Table 5-3: Tumor Assessment Requirements by Indication 142
Table 5-4: Criteria for Discontinuing Radiographic Assessments: 143
Table 6-1: Cabozantinib Tablet Components and Composition 146
Table 6-2: Atezolizumab Infusion Requirements and Guidance 149
Table 6-3: Dose Reductions of Cabozantinib (Oral Dosing) 154
Table 6-4: Dose Modifications for Cabozantinib-Associated AEs 155
Table 6-5: Dose Interruptions of Atezolizumab 156
Table 6-6: Dose Modifications for Atezolizumab-Associated AEs 157
Table 6-7: Management of Diarrhea Associated with Cabozantinib 161
Table 6-8: Management of Hypertension Associated with Cabozantinib 164
Table 6-9: Management of Palmar-plantar Erythrodysesthesia (PPE) Associated with Cabozantinib 166
Table 6-10: Management of Proteinuria Associated with Cabozantinib 167
Table 6-11: Management of Hepatotoxicity Associated with Cabozantinib for Subjects in Non-HCC Cohorts 172
Table 6-12 Management of Hepatotoxicity Associated with Cabozantinib for Subjects in the HCC Cohort 173
Table 6-13: Management Guidelines for Immune-Related Pulmonary Events, Including Pneumonitis 176
Table 6-14: Management Guidelines for Immune-Related Diarrhea or Colitis 177
Table 6-15: Management Guidelines for Endocrine Events 179
Table 6-16: Atezolizumab Management Guidance of Immune-Related Dermatologic Events 182
Table 6-17: Atezolizumab Management Guidance of Immune-Related Ocular Events 183

Table 6-18: Management Guidelines for Immune-Related Meningoencephalitis.....184
Table 6-19: Management Guidelines for Immune-Related Neurologic Disorders....185
Table 6-20: Management Guidelines for Pancreatic Events, Including Pancreatitis.186
Table 6-21: Management Guidelines for Immune-Related Myocarditis..................188
Table 6-22: Management Guidelines for Immune-Related Nephritis......................189
Table 6-23: Management Guidelines for Immune-Related Myositis.......................191
Table 6-24: Management Guidelines for Suspected Hemophagocytic Lymphohistiocytosis or Macrophage Activation Syndrome.................194
Table 6-25: Management Guidelines for Hepatic Events (All Tumor Types Except HCC)196
Table 6-26: Management Guidelines for Hepatic Events (HCC Subjects Only).......197
Table 6-27: Management of Hepatic Encephalopathy Associated with Study Treatment 198
Table 8-1: Adverse Events of Special Interest for Atezolizumab..........................211
Table 9-1: Example Blyth-Still-Casella Confidence Intervals for N=30 for ORR for Expansion Cohorts with 1-Sided Interpretations of the Lower Bound...216
Table 9-2: Example Blyth-Still-Casella Confidence Intervals for N=15 for ORR for the Expansion Cohorts of 15 Subjects with 1-Sided Interpretations of the Lower Bound...217
Table 9-3: Example Blyth-Still-Casella Confidence Intervals for N = 60 for ORR for Expansion Cohorts with 1-Sided Interpretations of the Lower Bound...218
Table 9-4: Example Blyth-Still-Casella Confidence Intervals for N=80 for ORR for Expansion Cohorts with 1-Sided Interpretations of the Lower Bound...219
Table 9-5: Example Blyth-Still-Casella Confidence Intervals for N=130 for ORR for Expansion Cohorts with 1-Sided Interpretations of the Lower Bound...220

LIST OF APPENDICES

Appendix A: Schedule of Assessments for the Dose-Escalation Stage......................250
Appendix B: Schedule of Assessments for the Expansion Stage (Combination-Therapy Cohorts [redacted] 257
[redacted]
Appendix D: Maintenance Phase...277
Appendix E: Preexisting Autoimmune Diseases and Immune Deficiencies...............280
Appendix F: Performance Status Criteria...281
Appendix G: Response Evaluation Criteria in Solid Tumors Version 1.1 (RECIST 1.1) 282
Appendix H: Immune-Related Response Criteria (irRECIST).................................291

Appendix I: Infusion-Related Reaction and Cytokine-Release Syndrome Guidelines 293
Appendix J: Potential Drug Interactions with Cabozantinib 296
Appendix K: Methods of Contraception 297
Appendix L: Child-Pugh Scoring System for Subjects with Chronic Liver Disease...298
Appendix M: COVID-19 Instructions...299

LIST OF ABBREVIATIONS

ACTH	adrenocorticotropic hormone
ADT	androgen deprivation therapy
AE	adverse event
AESI	adverse event of special interest
AIDS	acquired immunodeficiency syndrome
ALK	anaplastic lymphoma kinase
ALP	alkaline phosphatase
ALT	alanine aminotransferase
ANC	absolute neutrophil count
aoC#D#	Add-On Cycle # Day #
AST	aspartate aminotransferase
AUC	area under the plasma drug concentration-vs-time curve
aoW#D#	Add-on Week # Day #
BIRC	Blinded Independent Radiology Committee
BP	blood pressure
BSR	bone scan response
BUN	blood urea nitrogen
C#D# (eg, C1D1)	Cycle # Day #
CAP	chest/abdomen/pelvis
CFR	Code of Federal Regulations
CI	confidence interval
CNS	central nervous system
CR	complete response
CRC	colorectal cancer
CRF	case report form
CRPC	castration-resistant prostate cancer
CRS	cytokine-release syndrome
CT	computerized tomography
CTC	circulating tumor cell
CTCAE	Common Terminology Criteria for Adverse Events
CTEP	Cancer Therapy Evaluation Program
CYP	cytochrome P450
ddMVAC	dose-dense methotrexate, vinblastine, doxorubicin, and cisplatin
DILI	drug-induced liver injury
DLT	dose-limiting toxicity
DOR	duration of response
DVT	deep vein thrombosis
EC	Ethics Committee

ECG	electrocardiogram
ECOG	Eastern Cooperative Oncology Group
EGFR	epidermal growth factor receptor
FACS	fluorescence-activated cell sorting
FDA	Food and Drug Administration
FSH	follicle-stimulating hormone
FXa	Factor Xa
GC/GEJC	gastric cancer and gastroesophageal junction cancer
GCP	Good Clinical Practice
GFR	glomerular filtration rate
GI	gastrointestinal
GLP	Good Laboratory Practice
GnRH	gonadotropin-releasing hormone
GU	genitourinary
HBsAg	Hepatitis B surface antigen
HCV	Hepatitis C virus
HCV Ab	Hepatitis C virus antibody
HIV	human immunodeficiency virus
HLH	hemophagocytic lymphohistiocytosis
HNSCC	head and neck squamous cell carcinoma
HR	hazard ratio
IC+	percentage of tumor-associated immune cells with staining
ICF	informed consent form
ICH	International Conference on Harmonisation
ICI	Immune checkpoint inhibitor
ICP	Immune Cells Present
Ig	immunoglobulin
IHC	immunohistochemical
IMDC	International Metastatic RCC Database Consortium
INR	International Normalized Ratio
irAE	immune-related adverse event
IRB	Institutional Review Board
IRC	independent radiology committee
IRF	independent review facility
IRR	infusion-related reaction
IRT	Interactive Response Technology
irSAE	immune-related serious adverse events
ITT	intent-to-treat
IUD	intrauterine device

IUS	intrauterine hormone-releasing system
IV	intravenous
LEC	lower esophageal cancer
LDH	lactate dehydrogenase
LFT	liver function test
LH	luteinizing hormone
LMWH	low molecular weight heparins
MAS	macrophage activation syndrome
mCRC	metastatic colorectal cancer
mCRPC	metastatic castration-resistant prostate cancer
mCSPC	metastatic castration-sensitive prostate cancer
MDSC	myeloid-derived suppressor cell
MedDRA	Medical Dictionary for Regulatory Activities
MHC	major histocompatibility complex
MI	myocardial infarction
MMR	mismatch repair
MRI	magnetic resonance imaging
MSI	microsatellite instability
MSKCC	Memorial Sloan-Kettering Cancer Center
MTC	medullary thyroid cancer
MTD	maximum tolerated dose
NA	Not applicable
NCI	National Cancer Institute
NE	not estimable
NHT	novel hormonal therapy
NR	Not reported
NSAID	Non-steroid anti-inflammatory drug
NSCLC	non-small cell lung cancer
ONJ	osteonecrosis of the jaw
ORR	objective response rate
OS	overall survival
PARP	poly (ADP-ribose) polymerase
PD	progressive disease
PD-1	programmed death receptor 1
PD-L1	programmed death receptor 1 ligand
PE	pulmonary embolism
PFS	progression-free survival
PK	pharmacokinetic or pharmacokinetics
PO	by mouth

PPE	palmar-plantar erythrodysesthesia
PPI	proton pump inhibitor
PR	partial response
PSA	prostate-specific antigen
PSADT	prostate-specific antigen doubling time
PT	prothrombin time
PTT	partial thromboplastin time
qd	once daily
qod	every other day
QTcF	Corrected QT interval calculated by the Fridericia formula
RCC	renal cell carcinoma
RECIST (1.1)	Response Evaluation Criteria in Solid Tumors (version 1.1)
RPLS	reversible posterior leukoencephalopathy syndrome
RSI	reference safety information
RTK	receptor tyrosine kinase
SAA	Single-Agent Atezolizumab
SAC	Single-Agent Cabozantinib
SAE	serious adverse event
SAP	statistical analysis plan
SD	stable disease
SI	Système Internationale
SLD	Sum of lesion diameter
SNP	single nucleotide polymorphism
SOC	Study Oversight Committee
SoD	Sum of the diameters
T3	triiodothyronine
T4	thyroxine
TAM	tumor-assisted macrophage
TBS	technetium bone scan
TIA	transient ischemic attack
TKI	tyrosine kinase inhibitor
TPR	time point response
T_{reg}	regulatory T-cell
TSH	thyroid-stimulating hormone
UC	urothelial carcinoma
ULN	upper limit of normal
UPCR	urine protein/creatinine ratio
VAD	ventricular assist device
VEGF	vascular endothelial growth factor

VEGFR	vascular endothelial growth factor receptor
W#D#	Week # Day #
WBC	white blood cell

1 BACKGROUND AND RATIONALE

1.1 Background

Multi-targeted tyrosine kinase inhibitors (TKIs) and immune checkpoint inhibitors (ICIs) immunotherapies represent two systemic modalities that have been instrumental in the recent advancements of anticancer treatment over the past several years. Both classes of therapies have demonstrated broad clinical effects leading to new approved treatment options across multiple tumor types. The success of these therapy types as single agents with distinct mechanisms of action has naturally led to interest in evaluating combinations of TKIs with ICIs in search of further, possibly synergistic, anticancer clinical effects.

1.1.1 Atezolizumab

Atezolizumab is a humanized immunoglobulin (Ig) G1 monoclonal antibody which potently and selectively inhibits binding of programmed death receptor 1 ligand (PD-L1) on tumor cells and tumor infiltrating immune cells in the tumor microenvironment (McDermott et al 2016). Through this interaction, atezolizumab interrupts the negative regulatory effects of PD-L1 on T-cell proliferation and function that result from PD-L1 binding to programmed death receptor 1 (PD-1) and B7.1 (CD80) expressed on T lymphocytes and other immune cells. The result is an increase in the susceptibility of tumor cells to T-cell-meditated immune response, an effect that has been demonstrated in clinical activity across several tumor types.

Atezolizumab injection, for intravenous (IV) use (1200 mg once every 3 weeks [q3w]), has been approved in the US and the EU for the treatment of adult patients with advanced urothelial carcinoma (UC) after prior platinum containing chemotherapy or in a subset of patients who are considered cisplatin-ineligible (different patient populations are indicated depending on region; Rosenberg et al 2016, Balar et al 2017). Atezolizumab in combination with bevacizumab, paclitaxel, and carboplatin has been approved in US for the first-line treatment of adult patients with metastatic non-squamous non-small cell lung cancer (NSCLC) with no epidermal growth factor receptor (EGFR) or anaplastic lymphoma kinase (ALK) genomic tumor aberrations. Atezolizumab is also approved for adult patients with locally advanced or metastatic NSCLC after prior chemotherapy (Fehrenbacher et al 2016; Tecentriq™ US prescribing information [US PI] and European Medicines Agency Summary of Product Characteristics [EMA SmPC]). Atezolizumab has also been approved for first-line treatment in combination with carboplatin and etoposide in adult patients with extensive-stage small cell lung cancer (ES-SCLC; Horn et al 2018, Tecentriq US PI). Atezolizumab in combination with bevacizumab has been approved for the treatment of patients with unresectable or metastatic HCC who have not received prior systemic therapy (Tecentriq USPI and EMA SmPC). In addition, atezolizumab in combination

with cobimetinib and vemurafenib has been approved for the treatment of patients with BRAF V600 mutation-positive unresectable or metastatic melanoma (Tecentriq USPI and EMA SmPC). In these tumor indications, atezolizumab has either prolonged overall survival (OS) or induced durable disease responses. Notably, similar to other ICIs, the effects of single-agent atezolizumab on progression-free survival (PFS) were modest, suggesting the possibility of delayed anticancer immune effects contributing to the observed survival benefit (Fehrenbacher et al 2016). Like other ICIs, treatment with atezolizumab is generally well-tolerated but can be associated with immune-related adverse events (irAEs) including pneumonitis, hepatitis, colitis, endocrinopathies including hypophysitis, ocular toxicity, myocarditis, myositis, pancreatitis, and severe cutaneous adverse reactions (Michot et al 2016; Tecentriq US PI and EMA SmPC).

[redacted]

[redacted]

[redacted]

[redacted]

[redacted]

[redacted]

[redacted] [redacted]

[redacted]

[redacted]

[redacted]

[redacted]

[redacted]

[redacted] [redacted]

[redacted]

[redacted]

[redacted]

[redacted]

[redacted]

[redacted]

[redacted] [redacted]

[redacted]

[redacted]

[redacted]

[redacted]

[redacted]

[redacted]

[redacted]

[redacted] [redacted]

[redacted]

[redacted]

[redacted] [redacted]

[redacted]

1.1.1.8 Clinical Experience in Hepatocellular Carcinoma

In a Phase 1b study of atezolizumab (1200 mg q3w) in combination with the anti-VEGF targeting antibody bevacizumab, 103 subjects with advanced HCC naïve to systemic therapy had been enrolled at the data cutoff of 26 July 2018 (NCT02715531; Pishvaian et al 2018). Among 73 efficacy-evaluable subjects, the median survival follow-up was 7.2 months. The ORR by independent radiology facility (IRF) was 27% (with 4 complete responses [CRs]) per RECIST

1.1 and was 34% (with 8 CRs) per modified RECIST (mRECIST); ORR by the Investigator per RECIST 1.1 was 32% with 1 CR. Confirmed responses were reported across the patient population regardless of HCC etiology, geographic region, baseline alpha-fetoprotein (AFP) levels, or extrahepatic spread of tumor. The investigator-assessed median PFS per RECIST 1.1 was 14.9 months, and the IRF-assessed median PFS per RECIST 1.1 was 7.5 months. Median estimates for duration of response (DOR) and OS were not yet reached at the data cutoff of 26 July 2018. Among the 103 safety-evaluable subjects, treatment-related Grade 3 or 4 AEs were reported in 28 subjects (27%), most commonly hypertension (n = 10 [10%]). Five (5) Grade 5 AEs were observed, 2 of which were assessed as treatment related (one sepsis, one pneumonitis). A total of 19 subjects (18%) experienced treatment-related serious adverse events (SAEs). Adverse events of special interest (AESIs) of any grade for atezolizumab were reported for 54% of subjects, and AESIs of any grade for bevacizumab were reported for 47% of subjects. Immune-related AESIs for atezolizumab of ≥ Grade 3 requiring corticosteroid treatment included pneumonitis (2 subjects), autoimmune encephalitis, drug-induced liver injury (DILI), colitis, AST increased, γ-glutamyltranspeptidase (GGT) increased, diabetes mellitus, and pancreatitis (1 subject each). The high response rate observed suggested that the combination of atezolizumab with bevacizumab has synergistic activity in advanced HCC and compared favorably to early single-agent atezolizumab data in treatment-naïve HCC.

These results improved upon the preliminary single-agent data of atezolizumab in subjects with treatment-naïve advanced HCC, in which few objective responses were observed (the following data provided by Roche). Single-agent activity of atezolizumab in previously untreated advanced HCC has been explored in two single-arm Phase 1 studies. Study NCT01375842 enrolled five treatment-naïve HCC subjects; there were no responders per investigator assessment. In Study NCT02825940 (currently ongoing), there were two confirmed responses per Investigator assessment out of seven treatment-naïve subjects.

A Phase 3 study of atezolizumab in combination with bevacizumab compared with sorafenib in patients with untreated locally advanced or metastatic hepatocellular carcinoma is ongoing (NCT03434379).

1.1.1.9 Clinical Experience in Gastric Cancer

In a Phase 1 first-in-human dose escalation study (Taieb et al 2018; NCT01375842), atezolizumab was evaluated in subjects advanced gastric cancer (GC). Six subjects with advanced GC were enrolled following at least 2 prior systemic anticancer treatments. The ORR among the six subjects was 17%.

A randomized Phase 2 study of atezolizumab + FLOT (5-FU, calciumfolinat, oxaliplatin, and docetaxel) vs. FLOT alone in patients with advanced GC/GEJC is currently on-going (NCT03421288).

[redacted]

[redacted]

[redacted]

[redacted]

[redacted]

1.2 Cabozantinib

Cabozantinib (XL184) is a potent inhibitor of multiple receptor tyrosine kinases (RTKs) known to play important roles in tumor cell proliferation and/or tumor neovascularization including MET, vascular endothelial growth factor receptor (VEGFR), AXL, and RET. Increased expression of MET and AXL has been implicated in the development of resistance to VEGFR inhibitors in preclinical models of several cancers (Shojaei et al 2010, Zhou et al 2016, Sennino et al 2012, Ciamporcero et al 2015). In addition, targets of cabozantinib are implicated in promoting tumor-immune suppression including TYRO3, MER, and AXL (tumor-assisted macrophage [TAM] family kinases). Cabozantinib has demonstrated broad preclinical and clinical activity across several tumor types including RCC, UC, CRPC, HCC, and NSCLC. In the US and the EU, cabozantinib capsules (140 mg) have been approved for the treatment of progressive, metastatic medullary thyroid cancer (Elisei et al 2013; Cometriq™ US PI and EMA SmPC). Cabozantinib tablets (60 mg) have been approved in the US for the treatment of patients with advanced RCC and in the EU for the treatment of advanced RCC after prior VEGFR-targeted therapy and for previously untreated advanced RCC of intermediate or poor risk (Choueiri et al 2015, Choueiri et al 2016, Choueiri et al [J Clin Oncol] 2017, Choueiri et al [Ann Oncol] 2017, Cabometyx™ US PI and EMA SmPC). Based on the results from a randomized placebo-controlled Phase 3 study (CELESTIAL) in subjects who had received prior sorafenib, cabozantinib tablets (60 mg) as a single agent have also been approved in the US, EU, and other regions for an HCC indication (Cabometyx US PI and EMA SmPC). Cabozantinib tablets (60 mg) have also been approved for the treatment of patients with radioactive iodine (RAI)-refractory differentiated thyroid cancer (DTC) in the US and are currently under review with EMA and Japan agencies. In addition, cabozantinib tablets (40 mg) have been approved in the

United States, EU, and Japan for patients with advanced RCC, as a first-line treatment in combination with nivolumab.

Summaries of cabozantinib pharmacology, toxicology, PK, and clinical data are contained in the Investigator's Brochure supplied by the Sponsor (or designee), which must be reviewed before initiating the study.

1.2.1 Nonclinical Toxicology

Cabozantinib nonclinical toxicology has been characterized in single- and repeat-dose studies in multiple species. Details can be found in the Investigator's Brochure.

[redacted]

[redacted]

[redacted]

[redacted]

[redacted]

[redacted] [redacted]

[redacted]

[redacted]

[redacted]

[redacted] [redacted]

[redacted]

[redacted]

[redacted]

[redacted]

[redacted] [redacted]

[redacted]

[redacted]

[redacted]

[redacted]

[redacted]

[redacted] [redacted]

[redacted]

[redacted]

[redacted]

[redacted]

[redacted]

[redacted]

[redacted]

1.2.9 Clinical Experience in Hepatocellular Carcinoma

The clinical activity and safety of single-agent cabozantinib (60 mg, tablets) in HCC has been demonstrated in a randomized placebo-controlled Phase 3 study (CELESTIAL) in subjects who had received prior therapy with sorafenib (subjects were required to have progressed during or following prior systemic therapy and up to 2 prior lines of systemic therapy were allowed; Abou-Alfa et al 2018). The primary endpoint of the study was OS. At the second pre-planned interim analysis, the prespecified event-driven primary efficacy endpoint analysis of the 707 subjects enrolled at the data cutoff (470 cabozantinib, 237 placebo) demonstrated a statistically significant improvement in OS for subjects in the cabozantinib arm compared with placebo (Intent-to-Treat [ITT] population): the HR, adjusted for stratification factors, was 0.76 (95% CI: 0.63, 0.92; stratified log-rank p-value = 0.0049; critical p-value to reject the null hypothesis of equal OS = 0.021). The Kaplan-Meier estimates for median duration of OS were 10.2 months in the cabozantinib arm vs 8.0 months in the placebo arm. The secondary endpoint analysis of PFS as determined by the investigator yielded a median duration of PFS of 5.2 months in the cabozantinib arm and 1.9 months in the placebo arm. The HR, adjusted for stratification factors, was 0.44 (95% CI: 0.36, 0.52, stratified log-rank p-value < 0.0001). Investigator-determined objective response rate (ORR) was 4% and 0.4% for subjects in the cabozantinib and placebo arms, respectively (unstratified Fisher exact test p-value = 0.0059); all were partial responses (PRs). In addition, there was a high rate of stable disease (SD) in the cabozantinib arm relative to placebo (60% vs 33%). Adverse events reported for ≥ 20% of subjects in the cabozantinib arm by decreasing frequency were diarrhea, decreased appetite, palmar-plantar erythrodysesthesia (PPE), fatigue, nausea, hypertension, vomiting, aspartate aminotransferase (AST) increased, and asthenia. Grade 3 or 4 adverse events (AEs) regardless of causality were reported for 68% of subjects in the cabozantinib arm and 36% in the placebo arm. Grade 3 or 4 AEs reported for ≥ 5% of subjects in the cabozantinib arm by decreasing frequency were PPE, hypertension, AST increased, fatigue, diarrhea, asthenia, and decreased appetite Based on the results of this study in subjects who had received prior sorafenib, cabozantinib tablets (60 mg) as a single agent have been approved in the US, EU, and other regions for an HCC indication (Cabometyx US PI and EMA SmPC).

In a Phase 2 randomized discontinuation trial (RDT; NCT00940225; Kelley et al 2017), 41 subjects with advanced HCC were enrolled. Key eligibility criteria included up to 1 line of prior systemic therapy, documented progression of disease, and Child-Pugh score of A. Median

age was 60 years, and most subjects were male. Thirty-seven percent of subjects were of Asian ancestry. The most frequent cause of HCC was cirrhosis (44%), unknown (29%), HBV (24%), HCV (24%), and alcohol-related (20%). The majority of subjects (80%) had received prior systemic therapy; 54% had received sorafenib. Extrahepatic spread was present in 73% of subjects. Among 37 evaluable subjects, three had a confirmed PR. Seventy-eight percent had at least one tumor assessment which demonstrated a reduction in measurable disease. Of the 26 subjects with elevated baseline alpha-fetoprotein (AFP) and ≥ 1 post-baseline measurement, 9 (35%) had AFP response (> 50% reduction from baseline). Progression-free survival was 5.2 months, and OS was 11.5 months. The most common Grade 3/4 AEs, regardless of causality, were diarrhea (20%), PPE (15%), and thrombocytopenia (15%).

1.2.10 Clinical Experience in Gastric Cancer

In a Phase 1 study of cabozantinib, four subjects with advanced gastric cancer (GC) or gastroesophageal junction cancer (GEJC) were enrolled (Kurzrock et al 2011; NCT00215605). One subject with GEJC had a decrease in the sum of tumor measurements of approximately 20% from baseline.

In a Phase 2 randomized discontinuation trial (RDT) of cabozantinib, 21 subjects with advanced GC or GEJC were enrolled (Schöffski et al 2017; NCT00940225). Subjects were allowed to have received up to one prior systemic anticancer treatment. The primary endpoint ORR in the GC/GEJC cohort was 4.8% with 1 confirmed PR; there were also 8 subjects with SD as best response. The DCR (PR + SD) at Week 12 for the GC/GEJC cohort was 33% (95% CI: 14.6, 55.1). The safety results in the RDT study with cabozantinib were consistent with those for patients with advanced cancer treated with other VEGFR-TKIs.

[redacted]

[redacted]

[redacted]

[redacted]

[redacted] [redacted]

[redacted]

[redacted] [redacted]

[redacted]

[redacted]

[redacted]

[redacted]

1.2.14 Immunological Effects

Cabozantinib is a potent inhibitor of multiple RTKs known to play important roles in tumor cell proliferation and/or tumor neovascularization including MET, VEGFR, and RET. In addition, targets of cabozantinib are implicated in promoting tumor immune suppression including TYRO3, MER, and AXL (TAM family kinases). Through preclinical and preliminary clinical evaluation, cabozantinib treatment has been shown to affect tumor cells and the tumor microenvironment in a manner that would potentially make them more sensitive to immune-mediated attack. In vitro and in vivo experiments employing a murine colon carcinoma cell line (MC38-CEA) demonstrated that cabozantinib treatment altered immune modulation and immune subset conditioning (Kwilas et al 2014). Specifically, treatment of tumor cells with cabozantinib in vitro led to increased tumor-cell expression of major histocompatibility complex (MHC) class 1 antigen and greater sensitivity of tumor cells to T-cell-mediated killing. In a mouse MC38-CEA tumor model, cabozantinib treatment led to increased peripheral CD8+ T-cell counts, decreased regulatory T-cells (T_{reg}s) and myeloid-derived suppressor cells (MDSCs), and decreased T_{reg} suppressor activity. Further, synergistic effects including increased CD8+ T-cell infiltration and decreased infiltration by MDSCs and TAMs were observed when a poxviral-based cancer vaccine was administered in addition to cabozantinib in the mouse tumor model.

In the clinical setting, reductions in immunosuppressive T_{reg} lymphocytes following treatment with cabozantinib were observed in the Phase 2 study of subjects with advanced refractory UC discussed in Section 1.2.3 (Apolo et al 2014). In a Phase 2 study in metastatic triple-negative

breast cancer, cabozantinib-treated subjects experienced a persistent increase in the fraction of circulating CD3+ T lymphocytes and a persistent decrease in the CD14+ monocytes possibly reflecting activation of systemic antitumor immunity (Tolaney et al 2016).

Together, the preclinical and clinical observations presented above suggest that cabozantinib promotes an immunopermissive environment which might present an opportunity for synergistic effects from combination treatment with PD-1 checkpoint inhibitors.

1.3 Rationale

1.3.1 Rationale for the Study and Study Design

Rationale for Treatment Combination: Through potent inhibition of RTKs including MET, VEGFR, and RET, cabozantinib has demonstrated clinical activity as a single agent across multiple tumor types (see Section 1.2). In addition, targets of cabozantinib are implicated in promoting tumor-immune suppression including TYRO3, MER, AXL (TAM family kinases). Preclinical studies (Kwilas et al 2014, Song et al 2015, Lu et al 2017) and clinical observations on circulating immune suppressive cells and immune effector cells (Apolo et al [J Clin Oncol] 2014) suggest that cabozantinib promotes an immune-permissive environment which might present an opportunity for synergistic effects from combination treatment with ICIs which may be independent of tumor PD-L1 expression. Atezolizumab, a potent PD-L1 inhibitor that has also demonstrated clinical activity in multiple tumor types (see Section 1.1.1) is an appropriate combination therapy for this evaluation.

[redacted]

[redacted]

Rationale for Evaluating Selected Tumor Types: In the Expansion Stage, 20 tumor-specific cohorts in RCC, UC, CRPC, NSCLC, TNBC, OC, EC, HCC, GC/GEJC/LEC, CRC, H&N, and DTC will be enrolled to receive the combination treatment in order to further evaluate the safety and efficacy in these tumor indications on the recommended dose and schedule. The rationale for the planned Expansion Cohorts is based on available clinical activity and safety of both drugs in

these solid tumors as monotherapy or in combination therapies (see Section 1.2 [cabozantinib] and Section 1.1.1 [atezolizumab]).

In addition to exploring cabozantinib in combination with atezolizumab in subjects who had already received standard of care anticancer therapy, Expansion Cohorts [redacted] 14, [redacted] will include subjects with advanced cancer who have not received prior systemic anticancer therapy for inoperable locally advanced or metastatic cancer. This is supported by the observed clinical activity of cabozantinib and/or ICIs including atezolizumab in previously untreated subjects as well as the evolving treatment landscape in advanced solid tumors.

[redacted]

[redacted]

[redacted]

[redacted]

[redacted]

[redacted]

[redacted]

[redacted]

[redacted]

[redacted]

[redacted]

[redacted]

[redacted]

[redacted]

[redacted]

[redacted]

[redacted]

[redacted]

[redacted]

[redacted]

[redacted]

[redacted]

[redacted]

[redacted]

[redacted]

[redacted]

[redacted]

Rationale for Expansion Cohort 14 (HCC): In Expansion Cohort 14, subjects with advanced HCC (Child-Pugh A) who have not received prior systemic anticancer therapy will be enrolled. First-line therapy with single-agent VEGFR-TKIs has improved the outcome of subjects with advanced HCC; however, the survival benefit is modest with a median OS between 10.7 and 13.6 months (Llovet et al 2008; Kudo et al 2017). Both, cabozantinib and ICIs have shown promising single-agent clinical activity in advanced HCC (Abou-Alfa et al 2018; El-Khoueiry et al 2017). Based on the results from a randomized placebo-controlled Phase 3 study (CELESTIAL, NCT01908426) in subjects previously treated with sorafenib, cabozantinib tablets (60 mg) as a single agent have also been approved in the US, EU, and other regions for an HCC indication (Cabometyx US PI and EMA SmPC). Results of an ongoing clinical trial combining atezolizumab with bevacizumab suggest that a combination of ICI therapy with VEGF-targeting agents has synergistic clinical activity (Stein et al. ASCO 2018). Therefore, further evaluation of cabozantinib in combination with atezolizumab in previously untreated subjects with advanced HCC is warranted.

Rationale for Expansion Cohort 15 (GC/GEJC/LEC): In Expansion Cohort 15, subjects with advanced gastric cancer, gastroesophageal junction cancer, or lower esophageal cancer (GC/GEJC/LEC) who have received standard of care first-line therapy systemic anticancer therapy including platinum or fluoropyrimidine-containing chemotherapy and Her-2/neu targeted therapy if indicated will be enrolled. Since lower esophageal cancer is biologically similar to GEJC (both are adenocarcinomas) and is treated similarly, they began to be included in this cohort starting with Protocol Amendment 4.0. Current available therapies for GC/GEJC/LEC

generally do not provide durable responses, hence survival remains relatively short. Therefore, novel therapies with the potential to extend treatment response are needed. Cabozantinib and atezolizumab have shown preliminary single-agent activity in advanced GC/GEJC and may have the potential for synergist effects when used in combination as salvage therapy in subjects with advanced GC/GEJC/LEC (Schöffski et al 2017; Taieb et al 2018).

[redacted]

[redacted]

[redacted]

[redacted]

[redacted]

[redacted]

[redacted]

[redacted]

[redacted]

1.3.2 Rationale for Dosage Selection and Treatment Schedule

In accordance with the US PI, atezolizumab will be administered at the standard dosing regimen of 1200 mg as an IV infusion over 60 min (± 15 min) every 3 weeks (-2 days) on Day 1 of each 21-day cycle [redacted]

In the Dose-Escalation Stage, cabozantinib was to be administered orally at dose levels of 20, 40, or 60 mg in escalation cohorts. Sixty (60) mg is the approved tablet dose level for the single-agent treatment of advanced RCC and was also the dose used for the evaluation of cabozantinib as a single agent in the Phase 3 study in previously treated HCC and multiple Phase 2 studies in refractory metastatic UC, NSCLC, DTC, and CRPC. Dose reductions to

40 mg and 20 mg are utilized to manage AEs. In the Phase 3 METEOR study in RCC, the average daily dose was 41 mg/day, taking into account dose modifications. The Dose-Escalation Stage was initiated at the 40 mg cabozantinib dose level. This dose was also the recommended dose determined for cabozantinib for the clinical evaluation in combination with nivolumab, another antibody inhibitor of the PD-1/PD-L1 pathway (Apolo et al [Ann Oncol] 2016). The Dose-Escalation Stage cohorts used the Standard Dosing schedule where cabozantinib and atezolizumab were started on Day 1 of Cycle 1.

[redacted]

[redacted]

[redacted]

[redacted]

[redacted]

[redacted]

The Expansion Stage has been initiated (first subject enrolled on 26 March 2018) in multiple tumor cohorts with the recommended dose of the Dose-Escalation stage: cabozantinib 40 mg qd + atezolizumab 1200 mg q3w on the standard dosing schedule.

As of 21 May 2019, 268 subjects were enrolled across 18 Expansion Stage cohorts evaluating cabozantinib + atezolizumab in 12 different solid tumor types.

[redacted]

[redacted]

[redacted]

[redacted]

[redacted] [redacted]

[redacted]

[redacted]

Upon evaluation of safety and efficacy data of approximately 30 initially enrolled subjects in each Combination-Therapy Expansion Cohort, the Study Oversight Committee (SOC) may recommend extending enrollment with up to 100 additional subjects to further evaluate the clinical activity and tolerability of the combination therapy in those selected tumor cohort(s) (see Section 3.5.2.1 for further details). Extended enrollment in a cohort may occur at Dose Level 1 (cabozantinib 40 mg + atezolizumab 1200 mg) or Dose Level 2 (cabozantinib 60 mg + atezolizumab 1200 mg) depending on the observed clinical activity and safety of the combination therapy with atezolizumab. Extended enrollment at Dose Level 1 may be recommended by the SOC for tumor cohort(s) with encouraging clinical activity (eg, meaningful ORR). Extended enrollment at Dose Level 2 may be recommended by the SOC for tumor cohort(s) which do not meet the criteria for Extended Enrollment Option 1 to explore whether the higher cabozantinib dose will lead to improved clinical activity and maintain an acceptable safety profile. [redacted]

[redacted]

[redacted]

[redacted]

[redacted]

1.4 Overall Risk Benefit Assessment

The study will evaluate the safety, tolerability, and preliminary clinical activity of cabozantinib in combination with atezolizumab in tumor indications where at least one agent has either received regulatory approval or has demonstrated encouraging clinical activity in early stage trials (refer to Sections 1.1.1 and 1.2). In addition, a scientific rationale for a treatment combination of cabozantinib with an ICI has been established in both the preclinical and clinical settings (Kwilas et al 2014, Apolo et al 2014, Tolaney et al 2016). Further, encouraging

preliminary clinical activity and safety of cabozantinib in combination with a PD-1 targeting checkpoint inhibitor in ICI-naïve and ICI-pretreated subjects have been demonstrated in a Phase 1 study in subjects with GU cancer including metastatic UC and RCC (Nadal et al [Ann Oncol] 2017, Nadal et al 2018). In addition, in recent studies it has been demonstrated that an ICI in combination with cabozantinib or a TKI with a similar target profile as cabozantinib (inhibiting targets which regulate the immune system) was able to provide clinical benefit in cancer patients who progressed on prior ICI therapy (Nadal et al 2018, Leal et al 2017). The above suggests that combining ICI treatment with cabozantinib may result in a tumor microenvironment that is conducive to re-sensitization to ICI treatment after progression which could potentially address an important unmet medical need as the majority of cancer patients develop resistance and some patients are a priori refractory to ICI therapy.

[redacted]

[redacted]

The safety profiles of both cabozantinib and atezolizumab are well described based on multiple clinical evaluations. During an initial standard "3 + 3" Dose-Escalation Stage, a tolerable dose and dosing schedule of cabozantinib that can be administered in combination with the standard dose of atezolizumab in this study population was determined by a Cohort Review Committee (Section 12.1). The recommended dose for the Combination-Therapy Cohorts in the Expansion Stage of the study will be evaluated across 20 tumor cohorts. Throughout the study, all enrolled subjects will have to undergo regular safety visits in order to ensure adequate management and reporting of AEs. An SOC will periodically review safety and efficacy data of the Expansion Stage and recommend further enrollment with up to an additional approximately 100 subjects each in the Combination-Therapy Expansion Cohorts [redacted]

(Sections 9.1.2 and 12.2). The extended enrollment will be limited to up to 10 cohorts in the Expansion Stage [redacted] The Sponsor's Corporate Safety Governance (Section 12.3) will also periodically review safety data from all subjects enrolled in this study.

In order to minimize the safety risks to participating subjects, this protocol has eligibility criteria appropriate to the populations, and includes allowances for dose reductions (cabozantinib) and treatment delays (cabozantinib, atezolizumab). Periodic clinical assessments (physical examination, vital sign, and electrocardiographic assessments) and clinical laboratory tests will monitor for cabozantinib- and atezolizumab-related toxicities. Subjects will also be carefully monitored for AEs potentially related to inhibition of VEGFR by cabozantinib including gastrointestinal (GI) perforation, fistula formation, wound dehiscence, serious bleeding, proteinuria, hypertension, thromboembolic events, osteonecrosis, and reversible posterior leukoencephalopathy syndrome (RPLS) as well as immune-related side effects related to atezolizumab (pneumonitis, hepatitis, colitis, endocrinopathies, skin disorders, ocular events, neurological toxicity, pancreatitis, and infections).

Based on the clinical activity of cabozantinib and atezolizumab as single agents in multiple tumor types and the observed favorable tolerability and encouraging clinical activity of this combination in the Dose-Escalation Stage of this study, the potential benefit from cabozantinib administered alone or in combination with atezolizumab appears to outweigh the potential risks in subjects with advanced RCC, UC, CRPC, NSCLC, TNBC, OC, EC, HCC, GC/GEJC/LEC, CRC, head and neck squamous cell carcinoma (HNSCC), and DTC.

2 STUDY OBJECTIVES

[redacted]

[redacted]

- [redacted]

[redacted]

- [redacted]
- [redacted]

[redacted]

- [redacted]
- [redacted]

Expansion Stage (Combination-Therapy Cohorts):

The primary objective and endpoint is as follows:

- To evaluate preliminary efficacy of the combination therapy by estimating the ORR as assessed by the Investigator per RECIST 1.1

The secondary objective is as follows:

- To assess safety for the combination therapy through the evaluation of incidence and severity of nonserious AEs and SAEs, including irAEs and AESIs.

The exploratory objectives and endpoints are as follows:

- ORR as assessed by the Investigator per irRECIST for immune response
- DOR as assessed by the Investigator per RECIST 1.1
- PFS as assessed by the Investigator per RECIST 1.1
- ORR, DOR, and PFS as assessed by a Blinded Independent Radiology Committee (BIRC) per RECIST 1.1 for selected cohorts
- Overall survival
- Correlation of immune cell, tumor cell, and blood biomarker analyses with clinical outcome
- Changes in tumor infiltration and/or histology or other molecular changes as determined from optional tumor biopsy
- To further evaluate the plasma PK of daily oral administration of cabozantinib in subjects with solid tumors when given in combination with atezolizumab
- Tumor marker changes from baseline in select tumor indications
- Evaluation of mismatch repair (MMR) and microsatellite instability (MSI) status in relevant tumor indications

[redacted]

[redacted]

[redacted]

[redacted]

[redacted]

[redacted]

[redacted]

3 STUDY DESIGN

3.1 Overview

This is a multicenter, open-label Phase 1b study to assess safety, tolerability, preliminary efficacy, and PK of cabozantinib taken alone or in combination with atezolizumab in subjects with advanced RCC, UC, CRPC, NSCLC, TNBC, OC, EC, HCC, GC/GEJC/LEC, CRC, H&N cancer, and DTC. This study consists of two stages for the combination therapy: the Dose-Escalation Stage and the Expansion Stage. [redacted]

3.2 Study Sites

The Dose-Escalation Stage of this study will be conducted at up to 5 clinical sites in the US. Additional US, European, and Australian sites will be added (approximately 130 total sites) for the Combination-Therapy Cohorts, [redacted] in the Expansion Stage.

3.3 Blinding and Randomization

This is an open-label study with treatment arm assignment based upon currently enrolling dose level in the Dose-Escalation Stage and by tumor type and prior anticancer therapy in the Expansion Stage. There will be no blinding in this study.

[redacted] Subjects will be randomized, per an unstratified permuted block design, into the cohorts open to enrollment following the eligibility review process by the Sponsor. Randomized cohort assignment will be implemented as long as two or more cohorts with identical eligibility criteria are open to enrollment. Following confirmation of subject eligibility, Sponsor or CRO personnel will randomize subjects according to a centrally-maintained randomization list and enter the assigned cohort into the Interactive Response Technology (IRT).

Subjects are deemed to be enrolled in the study upon receipt of any study treatment. Subjects randomized who do not receive study treatment will not be considered enrolled into the study and will be classified as screen failures based upon the reason for not receiving treatment. Refer to Section 3.5.2 for cohort enrollment details.

3.4 Pretreatment Period

Potential subjects will be screened to determine if they meet eligibility criteria. Qualifying screening assessments must be performed within 28 days before first dose of study treatment unless otherwise stated (certain lab values must be obtained closer to first dose; see the schedules of assessment for details [redacted] Combination-Therapy Expansion Cohorts, [redacted] Appendix B]).

3.5 Treatment Period

Subjects will receive study treatment as long as they continue to experience clinical benefit in the opinion of the investigator or until there is unacceptable toxicity, the need for subsequent systemic anticancer treatment, or until any other reasons for treatment discontinuation listed in the protocol (Section 3.8). Treatment may continue after radiographic progression as long as the Investigator believes that the subject is still receiving clinical benefit from study treatment and that the potential benefit of continuing study treatment outweighs potential risk. Clinical judgment should be used for allowing treatment beyond radiographic progression. Subjects with clinically significant symptomatic deterioration at the time of radiographic progression may not be suitable for further treatment. The possibility of a delayed anti-tumor immune response should be taken into consideration: mixed responses with decreasing and increasing tumor lesion sizes at the same imaging time point or the appearance of new lesions prior to achieving a radiological response have been reported with ICI. For subjects in combination treatment cohorts, discontinuation of one component of the combination study treatment while continuing to receive the other may be allowed with Sponsor notification. For cohorts where the initial dose is 40 mg cabozantinib, intra-subject dose escalation of cabozantinib from 40 mg to 60 mg is allowed after Sponsor approval for subjects who are tolerating the 40 mg cabozantinib dose level well and have been treated on this dose level for at least 4 weeks.

[redacted]

All enrolled subjects will be treated with best supportive care while on study treatment. This excludes systemic nonprotocol anticancer therapy, which requires study treatment to be discontinued. Permitted study drug modifications to manage AEs will comprise dose reductions (from 60 mg to 40 mg daily, from 40 mg to 20 mg daily, or from 20 mg daily to 20 mg every other day [qod]) or interruptions for cabozantinib and dose delays for atezolizumab.

Special accommodations during the global COVID-19 pandemic are described in Appendix M.

[redacted]

[redacted]

[redacted]

[redacted]

[redacted]

[redacted]

[redacted]

[redacted]

[redacted]

[redacted]

[redacted]

[redacted]

[redacted]

[redacted]

[redacted]

[redacted]

[redacted] [redacted]

[redacted]

[redacted]

[redacted]

[redacted]

[redacted]

- [redacted]
- [redacted]
- [redacted]
- [redacted]

3.5.2 Expansion Stage (Combination-Therapy Cohorts, [redacted]

The Expansion Stage will enroll 20 different tumor cohorts to evaluate the recommended dose of the combination therapy from the Dose-Escalation Stage. [redacted]

After initial enrollment of approximately 30 subjects, cohorts in the Expansion Stage [redacted] may extend enrollment based on the available clinical data per the SOC (Figure 3-1). [redacted] The extended enrollment will be limited to 10 cohorts in the Expansion Stage with a maximum extended enrollment of up to 1000 subjects.

Sections 3.5.2.1, [redacted] describe the study design for the Combination-Therapy Expansion Cohorts, [redacted] An abbreviated description of Expansion Cohorts 1-18 [redacted] is provided in Table 3-3. [redacted] The Sponsor can decide to stop enrollment of any cohort at any time (eg, due to slow subject accrual). [redacted]

Figure 3-1: Expansion Stage Enrollment Overview (Maximum 1720 Subjects)

Expansion-Stage Cohorts 1-24

Dose level: Combination Therapy: Cabozantinib 40 mg + Atezolizumab 1200 mg

[redacted]

Initial enrollment: n ≈ 30 subjects per cohort [redacted] (total up to 720 subjects)

↓

Study Oversight Committee (SOC) Review:

Safety and Efficacy of initially enrolled subjects in each cohort

↓

The SOC Approves Extended Enrollment for up to 10 Cohorts in the Expansion Stage [redacted] (maximum 1000 additional subjects):

Combination-Therapy Cohorts

[redacted]

Option 1-Part I:
Cabozantinib **40 mg** +
Atezolizumab 1200 mg
Enrollment: n ≈ ***5[redacted]*** *subjects per cohort*
Total ≈ 80 subjects

Option 2:
Cabozantinib **60 mg** +
Atezolizumab 1200 mg
Enrollment: n ≈ ***30*** *subjects per cohort*
Total ≈ 60 subjects

[redacted]

Option 1-Part II:
Cabozantinib **40 mg** +
Atezolizumab 1200 mg
Enrollment: n ≈ ***50*** *subjects per cohort*
Total ≈ 130 subjects

[redacted]

Table 3-3: Summary of All Combination-Therapy [redacted]

Cohort	Tumor Type (Histology)	Abbreviated Eligibility Description	Initial Cohort Size (n)
		Combination-Therapy Cohorts	
[redacted]	[redacted]	[redacted]	[redacted]
[redacted]	[redacted]		[redacted]
[redacted]	[redacted]		[redacted]
[redacted]	[redacted]		[redacted]
[redacted]	[redacted]		[redacted]
[redacted]	[redacted]		[redacted]
[redacted]	[redacted]		[redacted]
[redacted]	[redacted]		[redacted]
14	HCC (adeno)	No prior systemic anticancer therapy	30
15	GC/GEJC/LEC (adeno)	Prior platinum- or fluoropyridine-containing therapy	30
[redacted]	[redacted]	[redacted]	[redacted]
[redacted]	[redacted]	[redacted]	[redacted]
[redacted]	[redacted]	[redacted]	[redacted]
[redacted]	[redacted]	[redacted]	[redacted]
[redacted]	[redacted]	[redacted]	[redacted]
		[redacted]	
[redacted]	[redacted]	[redacted]	[redacted]
[redacted]	[redacted]		[redacted]
		[redacted]	
[redacted]	[redacted]	[redacted]	[redacted]
		[redacted]	[redacted]

[redacted]

3.5.2.1 Combination-Therapy Expansion Cohorts

On 22 February 2018, the Cohort Review Committee identified the recommended dose and schedule of cabozantinib in combination with atezolizumab in the Dose-Escalation Stage and opened accrual in the Expansion Stage in March 2018. In this stage, 20 Combination-Therapy Expansion Cohorts in subjects with advanced RCC, UC, CRPC, NSCLC, TNBC, OC, EC, HCC, GC/GEJC/LEC, CRC, HNSCC, and DTC will be enrolled to obtain additional efficacy, safety, PK, and pharmacodynamic data at the recommended dose and schedule (the Standard Dosing Schedule) of cabozantinib in combination with atezolizumab. An abbreviated description of Expansion Cohorts 1-18 and 23-24 is provided in Table 3-3.

All Combination-Therapy Expansion Cohorts will initially enroll approximately 30 subjects (600 subjects in total).

Because of the high unmet medical need of patients with advanced, incurable cancer, the SOC may decide after periodic review of safety and efficacy data of the initially enrolled subjects in each Combination-Therapy Expansion Cohort to allow for additional enrollment to further assess the clinical activity and safety of the combination therapy.

Extended enrollment of a Combination-Therapy Expansion Cohort will be based on only one of the following two Extended Enrollment Options (see Figure 3-1):

- Extended Enrollment Option 1:
    - Part I: Combination-Therapy Expansion Cohorts may enroll approximately 50 additional subjects to receive the same dosing regimen as the initially enrolled approximately 30 subjects (cabozantinib 40 mg + atezolizumab 1200 mg) upon approval by the SOC. Decisions by the SOC regarding additional enrollment will be based on the clinical significance of the achieved ORR in the Expansion Cohorts and will include an evaluation of the lower bound of confidence intervals for ORR in the originally enrolled subjects. A minimum observed ORR of around 20% or more will be used as a target (though not a requirement) for the SOC to consider Part I of Extended Enrollment Option 1 (Section 9.1.2.1). The magnitude of ORR deemed clinically meaningful by the SOC may vary by cohort. The committee may also consider other factors of clinical benefit (eg, time to response, duration of response, safety/tolerability) in the decision to extend enrollment. [redacted]

[redacted]

- Part II: Combination-Therapy Expansion Cohorts may further enroll approximately 50 additional subjects for a maximum total of approximately 130 subjects in an expansion cohort. These subjects will receive the same dosing regimen as the previously enrolled subjects (cabozantinib 40 mg + atezolizumab 1200 mg) upon approval by the SOC. Decisions by the SOC regarding further extended enrollment in Part II of Extended Enrollment Option 1 will be based on the clinical significance of the achieved ORR in the Expansion Cohorts and will include an evaluation of the lower bound of confidence intervals for ORR in the previously enrolled subjects. A minimum observed ORR of around 35% or more will be used as a target (though not a requirement) for the SOC to consider Part II of the Extended Enrollment Option 1 (Section 9.1.2.1). Part II extension will only apply to tumor indications with a high-unmet medical need and very encouraging efficacy and safety data observed in Part I.

- Extended Enrollment Option 2: For Combination-Therapy Expansion Cohorts in which the initially enrolled approximately 30 subjects do not meet the criteria for Extended Enrollment Option 1, the SOC may decide to allow each selected Expansion Cohort to enroll approximately 30 additional subjects to receive the highest dose level of cabozantinib explored in the Dose-Escalation Stage (60 mg) in combination with atezolizumab 1200 mg to explore whether the higher cabozantinib dose will lead to improved clinical activity and maintain an acceptable safety profile.

Details about the composition, role, schedule, and guidance for committee decisions are provided in a separate SOC Charter.

All subjects enrolled in the Expansion Cohorts will be following the same frequency of assessments as described in Appendix B [redacted] For more details regarding the eligibility of subjects in the Expansion Cohorts refer to inclusion and exclusion criteria (Sections 4.2 and 4.3). Rationales for enrollment in each Expansion Cohort are provided in Section 1.3.1.

[redacted] [redacted]

[redacted]

[redacted]

[redacted]

[redacted] [redacted]

[redacted]

[redacted]

[redacted]

[redacted]

[redacted]

[redacted]

- [redacted]

- [redacted]

[redacted]

[redacted]

[redacted]

3.6 Post-Treatment Period and Survival Follow-up

The final safety assessment will occur at the Post-Treatment Follow-Up Visit 30 (+14) days after the date of the decision to discontinue treatment. If a subject is experiencing an ongoing treatment-related AE that led to study treatment discontinuation, SAE, or AESI at the time of that visit, the subject will continue to be followed until the AE has resolved, the AE has improved to Grade 2 or lower, or the Investigator determines that the event has become stable or irreversible. During the Post-Treatment Period, each subject will continue to be followed for survival. The Investigator (or designee) will make contact (eg, in person or by telephone) with the subject at least as frequently as every 12 weeks (± 14 days) after the Post-Treatment Follow-Up Visit, until the subject expires or the Sponsor decides to discontinue collection of these data for the study.

3.7 Maintenance Phase

The purpose of the Maintenance Phase is to continue to provide long-term access to study drug(s) to subjects who are deriving clinical benefit even after evaluation of the study objectives has been completed. When sufficient data have been collected to adequately evaluate all study endpoints, and upon site notification by the Sponsor, subjects remaining on study treatment or who have not completed the Post-Treatment Follow-Up Visit will enter the study Maintenance Phase. In the Maintenance Phase subjects who remain on treatment will continue to receive study treatment until a protocol-defined criterion for discontinuation has been met. With Sponsor notification, subjects in combination treatment cohorts may be allowed to discontinue one component of study treatment but continue to receive the other. After implementation of the Maintenance Phase, the study will be considered complete at sites and in countries that no longer have active subjects.

In the Maintenance Phase, subjects are to undergo periodic safety assessments (including local laboratory tests) and tumor assessments; the nature and frequency of these assessments are to be performed per standard of care if allowed by local regulations. It is the Investigator's responsibility to ensure that subject visits occur frequently enough and adequate assessments are performed to ensure subject safety. In order to continue to collect important safety information on subjects still enrolled in the study, reporting of SAEs; AEs (including irAEs), whether serious or not, leading to dose modifications or treatment discontinuation; AESIs; and other reportable events (pregnancy and medication errors with sequelae) is to continue per protocol requirements specific to the Maintenance Phase (Section 5.4).

Assessments in the Post-Treatment Period (including the Post-Treatment Follow-Up Visit) are not required for subjects who discontinue study treatment in the Maintenance Phase (such subjects are to be followed per standard of care). Further details are available in Appendix D.

3.8 Treatment Discontinuation and Withdrawals

Subjects may discontinue study treatment and assessments or withdraw their consent to participate in the study at any time without prejudice. When subjects withdraw consent, all study treatments will be stopped. The investigator may withdraw a subject from study treatment or from the study if, in his or her clinical judgment, it is in the best interest of the subject or if the subject cannot comply with the protocol. The investigator will also withdraw a subject from study treatment or from the study upon the Sponsor’s request or if the Sponsor chooses to terminate the study.

Any of the following conditions require withdrawal of the subject from study treatment:

- Subject no longer experiences clinical benefit as determined by the investigator (eg, disease progression and/or clinical deterioration attributable to disease progression of which both are unlikely to reverse with continued study treatment and/or supportive care). [redacted]
- Unacceptable side effects the investigator feels may be due to study treatment. However, discontinuation of one component of the combination study treatment while continuing to receive the other may be allowed for subjects in combination treatment cohorts with Sponsor notification in an effort to manage such side effects in subjects experiencing clinical benefit.
- The investigator feels it is not in the best interest of the subject to continue on study.
- Subject participation in another clinical study using an investigational agent, investigational medical device, or other intervention.
- Necessity for treatment with nonprotocol systemic anticancer therapy.
- Necessity for interrupting all study treatment for greater than 12 weeks for study-treatment related AEs unless approved by the Sponsor. (Note: temporary interruptions of study treatment for greater than 12 weeks due to the effects of COVID-19 and unrelated to AEs are described in Appendix M)
- Refusal of sexually active fertile subjects (excluding subjects who have been sterilized) to use highly effective methods of contraception (Appendix K).
- Female subjects who become pregnant.
- Subject request to discontinue study treatment (with or without concurrent withdrawal of informed consent).
- Significant noncompliance with the protocol schedule in the opinion of the investigator or the Sponsor.

The Sponsor should be notified of all subject study treatment discontinuations and study withdrawals as soon as possible. The reason for discontinuation or withdrawal will be documented.

For subjects who discontinue study treatment, every effort must be made to undertake protocol-specified follow-up procedures including end-of-treatment assessments, survival follow-up, and subsequent anticancer treatment unless consent to participate in the study is also withdrawn.

If a subject fails to return for the protocol-defined visits, an effort must be made to determine the reason. If the subject cannot be reached by telephone, at the minimum a registered letter should be sent to the subject (or the subject's legal guardian) requesting contact with the clinic.

If a subject is discontinued from study treatment because of an AE (including irAE) considered to be related to study treatment and the event is ongoing at the time of the Post-Treatment Follow-Up Visit 30 (+14) days after the date of the decision to discontinue treatment (see Section 5.3 for further details), the event must be followed until resolution or determination by the investigator that the event has become stable or irreversible.

If a subject withdraws consent to participate in the study, no further study procedures or assessments will be performed and no further study data will be collected for this subject other than the determination of survival status for subjects enrolled in the Expansion Stage. This information may be obtained from public records such as government vital statistics or obituaries, as permitted by local law.

3.9 Subject Replacements

Only subjects who sign the informed consent and receive any study treatment will be considered enrolled.

[redacted]

Subjects enrolled in the Expansion Stage will not be replaced.

4 STUDY POPULATION

4.1 Target Population

This study will enroll subjects with advanced solid tumors. The precise populations with these tumor types will vary between/within the Dose-Escalation Stage and cohorts in the Expansion Stage (Combination-Therapy Expansion Cohorts, [redacted] Eligibility criteria for this study have been carefully considered to ensure the safety of the study subjects and to safeguard the integrity of the study results. It is imperative that subjects fully meet all of the inclusion criteria and none of the exclusion criteria. The Sponsor will not grant waivers to study eligibility criteria.

Of note, in the eligibility criteria described below, maintenance anticancer therapy after the initial anticancer therapy does not count towards the limit of prior systemic therapies, provided there is no tumor progression between the initial anticancer therapy and the start of maintenance anticancer therapy. In addition, radiosensitization chemotherapy and retreatment with the same anticancer agent do not count towards the limit of prior systemic therapies.

[redacted]

4.2 Inclusion Criteria

A subject must meet all of the following criteria to be eligible for the study:

1. Cytologically or histologically and radiologically confirmed solid tumor that is inoperable locally advanced, metastatic, or recurrent:

   [redacted]

   <u>Expansion Stage:</u>

   [redacted]

[redacted]

- [redacted]

  [redacted]

- [redacted]

[redacted]

- [redacted]

[redacted]

- [redacted]
- [redacted]

[redacted]

[redacted]

- [redacted]
- [redacted]

[redacted]

- [redacted]
- [redacted]
- [redacted]

[redacted]

- [redacted]
  - [redacted]
  - [redacted]
- [redacted]

[redacted]

[redacted]

- [redacted]
- [redacted]

[redacted]

- [redacted]
- [redacted]

[redacted]

[redacted]

- [redacted]
- [redacted]
- [redacted]
    - [redacted]
    - [redacted]
- [redacted]

[redacted]

- [redacted]
- [redacted]
- [redacted]

[redacted]

- [redacted]
- [redacted]

p. Expansion Cohort 14: Subjects with advanced HCC who have a Child-Pugh score of A (Appendix L) and have not received prior systemic anticancer therapy for inoperable locally advanced, recurrent, or metastatic disease.

- *Subjects with active hepatitis B virus (HBV) infection (defined by HBsAg positive) must be on standard of care antiviral therapy and have HBV DNA < 500 IU/mL.*

- *Prior local-regional treatment (eg, radiofrequency ablation, transcatheter arterial chemoembolization [TACE]) is allowed.*

q. Expansion Cohort 15: Subjects with gastric cancer, gastroesophageal junction adenocarcinoma, or lower one-third esophageal adenocarcinoma who have radiographically progressed during or following platinum-containing or fluoropyrimidine-containing chemotherapy for inoperable locally advanced, recurrent, or metastatic disease.

- *Allowed are up to 2 lines of prior systemic anticancer therapy for inoperable locally advanced, recurrent, or metastatic disease.*
- *Prior HER-2/neu directed therapy is allowed.*

[redacted]

[redacted]

- [redacted]
- [redacted]

[redacted]

- [redacted]
- [redacted]
- [redacted]

[redacted]

- [redacted]

  [redacted]

- [redacted]
- [redacted]

  [redacted]

[redacted]

[redacted]

- [redacted]

[redacted]

[redacted]

[redacted]

- [redacted]
- [redacted]
- [redacted]
- [redacted]

[redacted]

- [redacted]

[redacted]

[redacted]

2. Measurable disease per RECIST 1.1 as determined by the Investigator. Measurable disease must be outside the radiation field if prior radiation therapy was administered.
3. Tumor tissue material available (archival or recent tumor biopsy).

[redacted]

4. Recovery to baseline or ≤ Grade 1 CTCAE v4 from toxicities related to any prior treatments, unless AE(s) are clinically nonsignificant and/or stable on supportive therapy.
5. Age eighteen years or older on the day of consent.
6. Eastern Cooperative Oncology Group (ECOG) Performance Status of 0 or 1.
7. Adequate organ and marrow function, based upon meeting all of the following laboratory criteria within 14 days before first dose of study treatment:
   a. Absolute neutrophil count (ANC) ≥ 1500/µL (≥ 1.5 × 10^9/L) without granulocyte colony-stimulating factor support within 2 weeks before screening laboratory sample collection.
   b. White blood cell count ≥ 2500/µL (≥ 2.5 × 10^9/L).
   c. Platelets ≥ 100,000/µL (≥ 100 × 10^9/L) without transfusion within 2 weeks before screening laboratory sample collection. For subjects with HCC ≥ 75,000/µL (≥ 75 × 10^9/L).
   d. Hemoglobin ≥ 9 g/dL (≥ 90 g/L) without transfusion within 2 weeks before screening laboratory sample collection.
   e. Alanine aminotransferase (ALT), aspartate aminotransferase (AST), and alkaline phosphatase (ALP) ≤ 3 × upper limit of normal (ULN). ALP ≤ 5 × ULN with documented bone metastases. For subjects with HCC: ALT, AST, and ALP ≤ 5 × ULN. [redacted]
   f. Total bilirubin ≤ 1.5 × ULN (for subjects with Gilbert's disease ≤ 3 × ULN). For subjects with HCC ≤ 2 mg/dL (≤ 34.2 µmol/L).
   g. Serum creatinine ≤ 1.5 × ULN or calculated creatinine clearance ≥ 40 mL/min (≥ 0.67 mL/sec) using the Cockcroft-Gault equation (see Table 5-2 for Cockcroft-Gault formula).
   h. Urine protein/creatinine ratio (UPCR) ≤ 1 mg/mg (≤ 113.2 mg/mmol). [redacted]
8. Capable of understanding and complying with the protocol requirements and must have signed the informed consent document.
9. Sexually active fertile subjects and their partners must agree to use highly effective methods of contraception that alone or in combination result in a failure rate of less than 1% per year when used consistently and correctly (defined in Appendix K) during the course of the study

and for 5 months after the last dose of study treatment. An additional contraceptive method, such as a barrier method (eg, condom), is recommended.

10. Female subjects of childbearing potential must not be pregnant at screening. Female subjects are considered to be of childbearing potential unless one of the following criteria is met: permanent sterilization (hysterectomy, bilateral salpingectomy, or bilateral oophorectomy) or documented postmenopausal status (defined as 12 months of amenorrhea in a woman over 45 years-of-age in the absence of other biological or physiological causes. In addition, females under 55 years-of-age must have a serum follicle stimulating hormone (FSH) level > 40 mIU/mL to confirm menopause). Note: Documentation may include review of medical records, medical examination, or medical history interview by study site staff.

4.3 Exclusion Criteria

A subject who meets any of the following criteria is ineligible for the study:

1. Prior treatment with cabozantinib or ICIs including anti-CTLA-4, anti-PD-1, anti-PD-L1, anti-PD-L2, anti-OX-40, anti-CD137 therapy [redacted]
2. Receipt of any type of small molecule kinase inhibitor (including investigational kinase inhibitor) within 2 weeks before first dose of study treatment. [redacted]
3. [redacted]
4. HCC subjects who meet any of the following criteria are ineligible:
    a. Received prior local anticancer therapy (including embolization and ablation) within 4 weeks before first dose of study treatment. For prior radiation for bone metastases, refer to Exclusion Criteria 6.
    b. Subjects with fibrolamellar HCC, sarcomatoid HCC, or mixed cholangiocarcinoma.
5. Receipt of any type of anticancer antibody (including investigational antibody) or systemic chemotherapy within 4 weeks before first dose of study treatment [redacted]
6. Radiation therapy for bone metastasis within 2 weeks, any other local radiation therapy within 4 weeks before first dose of study treatment. Subjects who have received systemic treatment with radionuclides within 6 weeks before first dose of study treatment are not eligible. Subjects with clinically relevant ongoing complications from prior radiation therapy are not eligible.

7. Known brain metastases or cranial epidural disease unless adequately treated with radiotherapy and/or surgery (including radiosurgery) and stable for at least 4 weeks before first dose of study treatment. Eligible subjects must be neurologically asymptomatic and without corticosteroid treatment at the time of first dose of study treatment.
8. Concomitant anticoagulation with oral anticoagulants except for those specified below.
    a. Allowed anticoagulants are:
        i. Prophylactic use of low-dose aspirin for cardioprotection (per local applicable guidelines) and low-dose low molecular weight heparins (LMWH)
        ii. Therapeutic doses of LMWH or specified direct factor Xa inhibitors rivaroxaban, edoxaban, or apixaban in subjects (excluding HCC subjects) without known brain metastases who are on a stable dose of the anticoagulant for at least 1 week before first dose of study treatment and without clinically significant hemorrhagic complications from the anticoagulation regimen or the tumor.

        Note: Subjects with HCC may be treated with therapeutic LMWH but must have a screening platelet count > 100,000/µL. Direct inhibitors of thrombin or factor Xa are not permitted in subjects with HCC.
9. Diagnosis of immunodeficiency or is receiving systemic steroid therapy (> 10 mg daily prednisone equivalent) or any other form of immunosuppressive therapy within 2 weeks prior to first dose of study treatment. Inhaled, intranasal, intraarticular, and topical corticosteroids and mineralocorticoids are allowed.
    Note: Adrenal replacement steroid doses > 10 mg daily prednisone equivalent are permitted in the absence of active autoimmune disease. Transient short-term use of systemic corticosteroids for allergic conditions (eg, contrast allergy) is also allowed.
10. Administration of a live, attenuated vaccine within 30 days before first dose of study treatment.
11. The subject has uncontrolled, significant intercurrent or recent illness including, but not limited to, the following conditions:
    a. Cardiovascular disorders:
        i. Congestive heart failure New York Heart Association Class 3 or 4, unstable angina pectoris, serious cardiac arrhythmias.
        ii. Uncontrolled hypertension defined as sustained blood pressure (BP) > 140 mm Hg systolic or > 90 mm Hg diastolic despite optimal antihypertensive treatment.
        iii. Stroke (including transient ischemic attack [TIA]), myocardial infarction (MI), or other ischemic event, or thromboembolic event (eg, deep venous thrombosis [DVT], PE) within 6 months before first dose. Upon Sponsor approval, subjects with a diagnosis of incidental, subsegmental PE or DVT within 6 months are allowed if stable, asymptomatic, and treated with anticoagulation for at least 1 week before first dose. Iatrogenic arterial embolization procedures such as tumor arterial embolization or splenic artery embolization are allowed.

b. Gastrointestinal (GI) disorders including those associated with a high risk of perforation or fistula formation:

   i. Tumors invading the GI-tract, active peptic ulcer disease, inflammatory bowel disease, diverticulitis, cholecystitis, symptomatic cholangitis or appendicitis, acute pancreatitis or acute obstruction of the pancreatic or biliary duct, or gastric outlet obstruction. Presence of primary GI tumor is not excluded.

   ii. Abdominal fistula, GI perforation, bowel obstruction, or intra-abdominal abscess within 6 months before first dose. Note: Complete healing of an intra-abdominal abscess must be confirmed before first dose.

   iii. Gastric or esophageal varices that are untreated or incompletely treated with bleeding or high risk for bleeding. Subjects treated with adequate endoscopic therapy (according to institutional standards) without any episodes of recurrent GI bleeding requiring transfusion or hospitalization for at least 6 months prior to study entry are eligible.

c. Clinically significant hematuria, hematemesis, or hemoptysis of > 0.5 teaspoon (2.5 mL) of red blood, or other history of significant bleeding (eg, pulmonary hemorrhage) within 12 weeks before first dose.

d. Cavitating pulmonary lesion(s) or known endobronchial disease manifestation.

e. Lesion invading a major blood vessel including, but not limited to, inferior vena cava, pulmonary artery, or aorta. HCC subjects with lesions invading the hepatic portal vasculature are eligible.

f. Other clinically significant disorders such as:

   i. Active or history of autoimmune disease or immune deficiency, including, but not limited to, myasthenia gravis, myositis, autoimmune hepatitis, systemic lupus erythematosus, rheumatoid arthritis, psoriatic arthritis, inflammatory bowel disease, antiphospholipid antibody syndrome, Wegener granulomatosis, Sjögren's syndrome, Guillain-Barré syndrome, or multiple sclerosis (see Appendix E for a more comprehensive list of autoimmune diseases and immune deficiencies). Subjects with the following conditions are eligible for the study:

      - A history of autoimmune-related hypothyroidism and on thyroid replacement hormone therapy

        Note: Subjects with prior history of thyroiditis are allowed if they have undergone sub-total, near-total, or total thyroidectomy.

      - Controlled Type 1 diabetes mellitus and on an insulin regimen
      - Asthma
      - Eczema, psoriasis, lichen simplex chronicus, or vitiligo with dermatologic manifestations only provided all of following are true:
        - Rash covers < 10% of body surface area

- Disease is well controlled at baseline and requires only low-potency topical corticosteroids
- No occurrence of acute exacerbations of the underlying condition requiring psoralen plus ultraviolet A radiation, methotrexate, retinoids, biologic agents, oral calcineurin inhibitors, or high potency or oral corticosteroids within the previous 12 months

ii. Active infection requiring systemic treatment, infection with human immunodeficiency virus (HIV) or acquired immunodeficiency syndrome (AIDS)-related illness, acute or chronic hepatitis B or C infection in non-HCC tumor cohorts, or a known positive test for tuberculosis infection if supported by clinical or radiographic evidence of disease. Subjects with history of COVID-19 must have recovered from the disease at least 30 days prior to enrollment.

iii. History of idiopathic pulmonary fibrosis, organizing pneumonia (eg, bronchiolitis obliterans), drug-induced pneumonitis, idiopathic pneumonitis, or evidence of active pneumonitis on screening chest computerized tomography (CT) scan. History of radiation pneumonitis in the radiation field (fibrosis) is permitted.

iv. Serious non-healing wound/ulcer/bone fracture.

v. Malabsorption syndrome.

vi. For all subjects [redacted]: Free thyroxine (FT4) outside the laboratory normal reference range. Asymptomatic subjects with FT4 abnormalities can be eligible after sponsor approval.

vii. Moderate to severe hepatic impairment for subjects with chronic liver disease (Child-Pugh B or C; Appendix L).

viii. Requirement for hemodialysis or peritoneal dialysis.

ix. History of solid organ or allogenic stem cell transplant.

12. Major surgery (eg, GI surgery, removal or biopsy of brain metastasis) within 4 weeks or minor surgery (eg, simple excision, tooth extraction) within 10 days before first dose of study treatment. Complete wound healing from surgery must have occurred before first dose. Subjects with clinically relevant ongoing complications from prior surgery are not eligible.

13. Corrected QT interval calculated by the Fridericia formula (QTcF) > 500 ms per electrocardiogram (ECG) within 14 days before first dose of study treatment (see Section 5.6.4 for Fridericia formula).

    Note: If a single ECG shows a QTcF with an absolute value > 500 ms, two additional ECGs at intervals of approximately 3 min must be performed within 30 min after the initial ECG, and the average of these three consecutive results for QTcF will be used to determine eligibility (ie, if the average is ≤ 500 ms the subject is eligible).

14. Pregnant or lactating females.

15. Inability to swallow tablets.

16. Previously identified allergy or hypersensitivity to components of the study treatment formulations. *Subjects with a history of infusion-related reaction to prior therapy with atezolizumab may be eligible by sponsor approval if the reaction was considered mild and manageable with appropriate supportive care (eg, use of premedication according to standard of care).*
17. Diagnosis of another malignancy within 2 years before first dose of study treatment, except for superficial skin cancers, or localized, low grade tumors deemed cured and not treated with systemic therapy. Incidentally diagnosed prostate cancer is allowed if assessed as stage ≤ T2N0M0 and Gleason score ≤ 6.

5 STUDY ASSESSMENTS AND PROCEDURES

The study assessment schedules are presented in [redacted] Appendix B for the Combination-Therapy Cohorts, [redacted]

Most study assessments and procedures (including treatment administration) will be performed in cycles. Cycle 1 Day 1 (C1D1) is defined as the date of first dose of any study treatment.

Cycles for the [redacted] Combination-Therapy Cohorts in the Expansion Stage: A cycle is generally the 21-day interval starting with the date of an atezolizumab infusion and ending with the day before the next atezolizumab infusion. However, under some circumstances no atezolizumab may be dosed during a cycle:

- If atezolizumab treatment is discontinued but cabozantinib treatment is allowed to continue with the notification of the Sponsor, each consecutive 21-day interval starting with the date of the decision to discontinue atezolizumab will be defined as a cycle. If the decision to discontinue atezolizumab occurs less than 21 days after the last infusion, then the next cycle will begin on the 22nd day after the last infusion.

Cycles may extend beyond 21 days if atezolizumab dosing is delayed. During an atezolizumab dose delay, subjects should return to the site for scheduled safety visits every three weeks from the last dose of atezolizumab. Further, the study site should perform unscheduled visits or telephone calls weekly (or more frequently) as clinically indicated to monitor subject safety and appropriateness for re-treatment with study treatment.

[redacted]

[redacted]

[redacted]

[redacted]

Imaging Schedule: Imaging assessments (CT, magnetic resonance imaging [MRI], bone scan) are to be performed at protocol-defined intervals based on the first dose of study treatment (defined as Week 1 Day 1 [W1D1]); all subsequent time points for these assessments will apply the same nomenclature, which will not be modified as a result of modifications or discontinuations of treatment administration. The frequencies of imaging assessments are provided [redacted] in Appendix B for the Combination-Therapy Cohorts, [redacted] in the Expansion Stage, [redacted]

Considerations for Maintaining Assessment Schedules:

Unless otherwise indicated, in the absence of toxicity all scheduled visits will occur within windows for the protocol-specified visit schedule. If the subject experiences toxicity, study treatment can be modified or delayed as described in Section 6.5. If the subject is unable to have a study assessment taken within the defined time window due to an event outside of his or her control (eg, clinic closure, personal emergency, inclement weather, vacation), the assessment should be performed as close as possible to the required schedule. Special accommodations during the global COVID-19 pandemic are described in Appendix M. Laboratory panels for serum chemistry, hematology, and urinalysis are defined in Section 5.6.5.

5.1 Pretreatment Period

Informed consent must be obtained prior to initiation of any clinical screening procedure that is performed solely for the purpose of determining eligibility for research; however, evaluations performed as part of routine care prior to informed consent can be utilized as screening evaluations if permitted by the site's Institutional Review Board (IRB)/ Ethics Committee (EC) policies. Informed consent may be obtained greater than 28 days before first dose of study treatment. At informed consent, subjects will be assigned a subject identifier; subject identifiers are not to be re-assigned if a subject is determined to be ineligible, and subjects are to maintain their original identifier if re-screening is required or if the subject experiences a change in study site or investigator.

To determine subject eligibility as stipulated in Section 4, subjects will undergo required screening evaluations as outlined in [redacted] Appendix B (Combination-Therapy Expansion Cohorts, [redacted] and as described in Section 5.6. Qualifying screening assessments must be performed within 28 days before the first dose of study treatment unless otherwise stated (certain lab values must be obtained closer to first dose of study treatment). Eligibility criteria based on laboratory values will use the central laboratory result (except serum pregnancy test and 24-hour urine protein; see Section 5.6.5). Local laboratory assessments may be obtained and used if the results are required by the investigator in a rapid timeframe to confirm eligibility. Local laboratory results used for confirmation of eligibility must be forwarded to the local laboratory management vendor. Study eligibility is based on a subject meeting all of the study inclusion criteria and none of the exclusion criteria at screening.

5.2 Treatment Period

While the subject is receiving study treatment, the subject's clinical status is to be evaluated by an Investigator at each clinic visit to confirm that the subject is suitable for continuing study treatment and to make timely decisions regarding the interruption or restarting of study treatment. Clinical laboratory results from samples obtained during clinic visits and tumor assessments from imaging visits are to be reviewed by an Investigator. Also refer to Section 5.6.5 for handling of samples for laboratory assessments.

Subjects will receive study treatment as long as they continue to experience clinical benefit in the opinion of the investigator or until unacceptable toxicity, the need for subsequent systemic anticancer treatment, or until any other reasons for treatment discontinuation listed in the protocol (Section 3.8). Administration of study treatment may continue after radiographic progression per RECIST 1.1 as long as subjects meet all of the following criteria:

- Evidence of clinical benefit, as determined by the investigator following a review of all available data
- Absence of symptoms and signs indicating unequivocal progression of disease (eg, laboratory values, such as clinically significant hypercalcemia for subjects with RCC) that cannot be managed by optimizing supportive therapy
- Absence of decline in ECOG performance status that can be attributed to disease progression
- Absence of tumor progression at critical anatomical sites (eg, leptomeningeal disease) that cannot be managed by protocol-allowed medical interventions.

[redacted]

The investigator should take into consideration the possibility of a delayed anti-tumor immune response with the possibility of regressing and enlarging tumor lesions at the same imaging time point (mixed response) or the appearance of new lesions prior to achieving a radiological response.

Clinic visits for safety evaluations will occur at minimum every 3 weeks (± 3 days) after treatment is initiated independent of any dose delays or interruptions. The final assessment will occur at the Post-Treatment Follow-Up Visit unless an AE is determined to be ongoing (see Section 5.3).

If study treatment is interrupted or delayed due to AEs, investigators should perform additional safety assessments weekly (or more frequently as clinically indicated).

Radiographic tumor assessments will be performed as described in Section 5.6.9. The schedule of assessments should be followed regardless of whether study treatment is reduced, interrupted, delayed, or discontinued.

[redacted]

5.2.2 Expansion Stage

Subjects in the Expansion Stage will be assigned to a treatment cohort based on tumor type and prior cancer history as described in Section 3.5.2.

5.2.2.1 Combination-Therapy Cohorts

The cabozantinib starting dose level defined by the Cohort Review Committee in combination with atezolizumab will be administered to the initially enrolled subjects in the Expansion Cohorts and any additional subjects enrolled per Extended Enrollment Option 1. Additional

subjects enrolled per Extended Enrollment Option 2 will receive cabozantinib 60 mg in combination with the standard dose of atezolizumab. For details on the Extended Enrollment Options, see Section 3.5.2.1.

Study treatment will be administered according to the Cohort Review Committee-recommended dosing schedule. Expansion Cohort subjects will be evaluated according to the scheduled of assessments provided in Appendix B.

[redacted]

[redacted]

[redacted]

5.3 Post-Treatment Period

Subjects who discontinue from study treatment will return to the site 30 days (+ 14 days) after the date of the decision to discontinue study treatment for a Post-Treatment Follow-Up Visit. During the Post-Treatment Follow-Up Visit, safety assessments will be performed. Refer to [redacted] Appendix B (Combination-Therapy Expansion Cohorts, [redacted] for a description of all assessments for the Post Treatment Follow-Up Visit.

The date of the decision to discontinue study treatment is defined for each subject as the later of (a) the date of the decision of the Investigator to permanently discontinue study treatment or (b) the date of the last dose of study treatment taken by the subject.

Adverse events (including irAEs and AESIs) are to be documented and/or followed as described in Section 8.3.

Subjects will be followed for OS as described in Section 5.6.11. Receipt of nonprotocol anticancer therapy will be collected during survival follow-up. If a subject is lost to follow-up, multiple attempts to contact the study subject or designee must be documented in the subject records.

Radiographic tumor assessments may need to be collected until radiographic progression as described in Section 5.6.9.

These assessments in the Post-Treatment Period (including the Post-Treatment Follow-Up Visit) are not required for subjects who discontinue study treatment in the Maintenance Phase (such subjects are to be followed per standard of care).

5.4 Maintenance Phase

The purpose of the Maintenance Phase is to continue to provide long-term access to study drug(s) to subjects who are deriving clinical benefit even after evaluation of the study objectives has been completed. When sufficient data have been collected to adequately evaluate all study endpoints, and upon site notification by the Sponsor, subjects who continue study treatment or who have not completed the Post-Treatment Follow-Up Visit will enter the study Maintenance Phase. Upon initiation of the Maintenance Phase, the Sponsor considers the safety and efficacy profile of the drug within this study to have been sufficiently established for regulatory purposes. After implementation of the Maintenance Phase, the study will be considered complete at sites and in countries that no longer have active subjects.

In the Maintenance Phase, subjects who remain on treatment will continue to receive study treatment until a criterion for protocol-defined discontinuation has been met (Section 3.8). Subjects are to undergo periodic safety assessments (including local laboratory tests) and tumor assessments (Appendix D). The nature and frequency of these assessments are to be performed per standard of care if allowed per local regulations. It is the Investigator’s responsibility to ensure that subject visits occur frequently enough and adequate assessments are performed to ensure subject safety.

Subjects who enter the Maintenance Phase after discontinuing study treatment, but prior to their Post-Treatment Follow Up Visit, are to be followed in the Maintenance Phase until their Post-Treatment Follow Up Visit.

In order to continue to collect important safety information on subjects still enrolled in the study, reporting of SAEs, AESIs, and other reportable events (pregnancy and medication errors with sequelae) is to continue per protocol (Section 8).

Further, the following AEs (including irAEs), whether serious or not, are to be reported using the same process as for reporting SAEs described in the protocol Section 8.2 (though SAE reporting timeline requirements do not apply to non-serious events reported in these categories):

- Adverse events (including irAEs), whether serious or not, leading to study treatment discontinuation
- Adverse events (including irAEs), whether serious or not, leading to study treatment dose modification (ie, causing study treatment to be interrupted, delayed, or reduced)

Study drug accountability is to continue as described in Section 6.4.

Only data collected prior to implementation of Maintenance Phase will be reported in a clinical study report.

5.5 Unscheduled Visits or Assessments

If the investigator determines that a subject should be monitored more frequently or with additional laboratory parameters assessments than indicated by the protocol-defined visit schedule, unscheduled visits or assessments are permitted. The laboratory assessments will be done by the central lab; however, if the results are needed immediately (eg, for AE management), they may be done by the local lab and the results forwarded to the management vendor for handling of local laboratory data. Whenever possible a sample for central lab analysis will also be collected. During a dose interruption due to AE (ie, the time between the last dose and the time drug is restarted), the study site should perform unscheduled visits or telephone calls weekly (or more frequently) as clinically indicated to monitor subject safety and appropriateness for re-treatment with study treatment.

5.6 Procedure Details

5.6.1 Demographics, Medical and Cancer History

Demographics at screening will include age at informed consent, medical and cancer history [redacted] surgical history, radiation therapy

history, and systemic anticancer treatment history including names of agents and administration dates. Refer to [redacted] Appendix B for the Combination-Therapy Expansion Cohorts, [redacted]

5.6.2 Physical Examination

Physical examinations at screening will include height, weight, performance status, and an assessment of the following systems: skin, head, eyes, ears, nose, throat, respiratory system, cardiovascular system, GI system, neurological condition, blood and lymphatic systems, and the musculoskeletal system. Symptom-directed physical examination will be conducted on C1D1 before the first dose of study treatment and at subsequent safety assessment visits. Any ongoing / intercurrent condition(s) prior to first dose will be recorded in source documents and on case report forms (CRFs).

The Karnofsky performance status will be assessed during screening for subjects with RCC to determine the prognostic risk score according to the MSKCC prognostic criteria (Motzer et al 2004). For all subjects the ECOG performance status will be assessed at screening and at subsequent visits. A table for both performance status scores is included in Appendix F for reference.

Refer [redacted] for the schedule of physical examination and performance status assessments [redacted] Appendix B for the Combination-Therapy Expansion Cohorts, [redacted]

5.6.3 Vital Signs

Vital signs including approximately 5-minute sitting BP, pulse, respiratory rate, and temperature will be assessed at the time points indicated in [redacted] Appendix B (Combination-Therapy Expansion Cohorts, [redacted] in the Expansion Stage), [redacted] On atezolizumab infusion days, vital signs should be assessed within 60 min prior to initiation of the infusion, and further vital sign assessment should be performed during and after the infusion as clinically indicated.

5.6.4 Electrocardiogram Assessments

At screening and during the study, single ECG assessments will be performed with standard 12-lead ECG equipment according to standard procedures to determine the corrected QT interval

calculated by the Fridericia formula (QTcF). If at any time the single ECG shows a QTcF with an absolute value > 500 ms, two additional ECGs at intervals of approximately 3 min must be performed within 30 min after the initial ECG, and the average of these three consecutive results for QTcF will be used (see Section 6.5.2.1.15).

ECGs will be performed at the time points indicated in [redacted] Appendix B (Combination-Therapy Expansion Cohorts, [redacted]

Abnormalities in the ECG that lead to a change in subject management (eg, dose reduced or interrupted, treatment discontinued; requirement for additional medication or monitoring) or result in clinical signs and symptoms are considered clinically significant for the purposes of this study and will be deemed AEs. If values meet criteria defining them as serious, they must be reported as SAEs (Section 8.2).

The Fridericia formula is depicted below for calculation of QTcF.

$$QTcF = \frac{QT}{RR^{1/3}}$$

QT = measured QT interval in milliseconds; RR = measured R to R interval (which can be derived from the heart rate as 60/heart rate)

5.6.5 Laboratory Assessments

Laboratory analytes that will be measured for this study are listed in Table 5-1. The schedule for laboratory assessments is provided in [redacted] Appendix B for the Combination-Therapy Expansion Cohorts, [redacted] Laboratory tests to establish eligibility must be done within 14 days prior to first dose of study treatment unless otherwise stated.

Hematology, serum chemistry, coagulation, UPCR including components, and thyroid function tests are to be performed by a central laboratory for samples collected at scheduled safety visits and at unscheduled visits whenever possible. All central laboratory results will be provided to the investigator. Local laboratory assessments for these panels may be obtained and used if the results are required by the investigator in a rapid timeframe. All local laboratory results must be forwarded to the study local laboratory management vendor if performed in lieu of the central laboratory assessment at any scheduled or unscheduled visit.

Routine (dipstick) urinalysis, microscopic urine examination, and serum and urine pregnancy tests are to be performed by a local laboratory. Results or status from these tests will be recorded on CRFs and will not be submitted to the study local laboratory management vendor.

If performed to determine eligibility or at any scheduled or unscheduled visit, 24-hour urine protein tests are to be performed by a local laboratory and the lab results are to be forwarded to the study local laboratory management vendor.

Serum chemistry, hematology, and urinalysis laboratory samples must be collected and the results must be reviewed within 72 h before any atezolizumab infusion administered on study.

Throughout the study, glucose is to be monitored.

A serum pregnancy test must be repeated before dosing on C1D1 unless a pregnancy evaluation was done during screening within 7 days prior to C1D1. Pregnancy tests (serum or urine) will be performed for post-baseline pregnancy evaluations.

Follicle stimulating hormone will be assessed during screening for women under the age of 55 years to confirm menopause (see Inclusion Criterion #10 in Section 4.2).

For all tumor types except HCC: Hepatitis B surface antigen and Hepatitis C antibody (with reflex testing of Hepatitis C virus RNA if antibody test is positive) will be assessed at screening.

For HCC subjects: Hepatitis B surface antigen, Hepatitis B core antibody, Hepatitis B e-antigen/e-antibody, Hepatitis B DNA, Hepatitis D testing for Hepatitis B positive, Hepatitis C antibody, and Hepatitis C virus RNA will be assessed at screening.

[redacted]

Additional tumor markers will be assessed for selected tumor indications: [redacted], AFP for HCC, [redacted] Subjects with these tumor types will have the corresponding tumor markers assessed at screening and after first dose of study treatment as described in Section 5.6.9.4 and the schedule of assessments for the Combination-Therapy Expansion Cohorts, [redacted] (Appendix B), [redacted]

Table 5-1: Clinical Laboratory Panels

Central Laboratory<br>*If performed by local laboratory in lieu of central lab assessment, submit results to study local laboratory management vendor*		
**Laboratory Assessments for All Cohorts**		**Cohort-Specific Assessments**
**Hematology:**<br>• White blood cell (WBC) count with differential (ANC, basophils, eosinophils, lymphocytes, monocytes)<br>• hematocrit<br>• platelet count<br>• red blood cell count<br>• hemoglobin<br><br>**Coagulation:**<br>• prothrombin time (PT)/International Normalized Ratio (INR)<br>• partial thromboplastin time (PTT)<br><br>**Thyroid function:**<br>• thyroid-stimulating hormone (TSH)<br>• Free thyroxine (T4; required at screening; after screening only if TSH is outside normal range)<br><br>**Urine Chemistry:**<br>• Protein (spot urine; fully quantitative)<br>• Creatinine (spot urine; fully quantitative)<br>• Urine protein/creatinine ratio (UPCR; spot urine)	**Serum Chemistry**<br>• albumin<br>• total alkaline phosphatase (ALP)<br>• amylase<br>• alanine amino transferase (ALT)<br>• aspartate amino transferase (AST)<br>• blood urea nitrogen (BUN)<br>• corrected calcium<br>• bicarbonate<br>• chloride<br>• creatinine<br>• γ-glutamyltranspeptidase (GGT)<br>• glucose<br>• lactate dehydrogenase (LDH)<br>• lipase<br>• magnesium<br>• phosphorus<br>• potassium<br>• sodium<br>• total bilirubin (conjugated and unconjugated if total bilirubin elevated)<br>• total protein<br><br>**Hepatitis screening (All Non-HCC Cohorts):**<br>• Hepatitis B surface antigen (HBsAg; screening)<br>• Hepatitis C virus antibody (HCV Ab; HCV RNA reflex testing if antibody positive [screening])<br><br>**Other Parameters**<br>• Follicle Stimulating Hormone (FSH)[a]	[redacted]<br><br>[redacted]<br><br>**HCC Cohort 14:**<br>• Alpha-fetoprotein (AFP)<br>• Screening assessments:<br>• Hepatitis Viral assessments:<br>○ Hepatitis B surface antigen (HBsAg)<br>○ Hep B core antibody<br>○ Hep B e-antigen/e-antibody<br>○ HBV DNA<br>○ Hepatitis C virus antibody (HCV Ab)<br>○ Hep C viral load (PCR)HDV testing for HBV positive<br><br>[redacted]<br><br>[redacted]

[redacted] HCC, hepatocellular cancer.

Local Laboratory (All Cohorts)<br>*Submit only 24-hour urine protein test results to study local laboratory management vendor*		
**Urinalysis**<br>(Dipstick or Routine as per institutional standard)<br>• pH<br>• specific gravity<br>• ketones<br>• protein<br>• glucose<br>• nitrite<br>• urobilinogen<br>• leukocyte esterase<br>• blood	**Microscopic Urine Examination**<br>• Perform at the discretion of the investigator based on results or routine urinalysis or as clinically indicated<br>**Urine Chemistry**<br>• 24-hour urine protein: perform at the discretion of the investigator based on increases in UPCR from routine assessments	**Pregnancy Blood Test (prior to first dose)**<br>• β-human chorionic gonadotropin (β-HCG)<br>**Pregnancy Urine or Blood Test (after first dose of study treatment)**<br>• β-human chorionic gonadotropin (β-HCG)

[a] For women under the age of 55 years to confirm menopause as needed. Local laboratory tests can be used, if necessary.

Table 5-2: Estimation of the Creatinine Clearance by Cockcroft and Gault

Based on Serum creatinine in conventional units (mg/dL)<br>• Males: (140 – age) x weight (kg)/(serum creatinine × 72)<br>• Females: [(140 – age) x weight (kg)/(serum creatinine × 72)] × 0.85
Based on Serum creatinine in SI units (µmol/L)<br>• Males: [(140 – age) x weight (kg)/(serum creatinine)] × 1.23<br>• Females: [(140 – age) x weight (kg)/(serum creatinine)] × 1.04

Abnormalities in any clinical laboratory test (including tests not required per protocol) that lead to a change in subject management (eg, dose interrupted, delayed, or reduced, treatment discontinued; requirement for additional medication or monitoring) are considered clinically significant for the purposes of this study and will be reported as AEs. If laboratory values constitute part of an event that meets criteria defining it as serious, the event (and associated laboratory values) needs to be reported as an SAE (see Section 8.2).

5.6.6 Pharmacokinetic Assessments

Unless otherwise approved by the Sponsor, PK blood samples will be obtained from all enrolled subjects as described in Section 5.6.6.1 as well as [redacted] in Appendix B for the Combination-Therapy Expansion Cohorts, [redacted].

5.6.6.1 Pharmacokinetic Blood Samples

Samples will be collected for the evaluation of cabozantinib and atezolizumab PK. The plasma concentrations of cabozantinib and serum concentration of atezolizumab will be measured, and the results will be used to confirm exposure to cabozantinib and atezolizumab, to identify possible drug-drug interactions between cabozantinib and atezolizumab, and to further characterize the PK for cabozantinib in these populations. Collection of PK samples may be halted early or sampling frequency may be modified at the discretion of the Sponsor.

[redacted]

[redacted]

Expansion Stage

Combination-Therapy Expansion Cohorts:

Blood samples for PK analysis will be obtained on the date of first dose of study treatment (C1D1; prior to study treatment administration [cabozantinib and atezolizumab], approximately 5 min after completion of the atezolizumab infusion, and 2 h after the first dose of cabozantinib) and prior to study treatment dosing on C2D1 and C3D1.

[redacted]

[redacted]

[redacted]

[redacted]

[redacted]

[redacted]

[redacted]

5.6.7 Immunogenicity Assessments

Blood samples will be obtained from all subjects in the combination treatment cohorts [redacted] in the Expansion Stage for immunogenicity assessment predose on C1D1, C3D1, C7D1, and at the Post-Treatment Follow-up Visit.

[redacted]

5.6.8 Biomarker Assessments

Blood and tissue samples will be obtained from consented subjects for analysis of established and/or exploratory biomarkers. Refer to [redacted] for the schedule for these assessments [redacted] Appendix B for the Combination-Therapy Expansion Cohorts, [redacted] in the Expansion Stage, [redacted]

The required and optional blood samples will be used to study plasma, serum, and cellular biomarkers. Archival and optional fresh tumor tissue samples will be used to evaluate changes in biomarker expression and genetic/genomic alterations. The analyses will help identify biomarkers that are predictive of response to the study drug, and may help improve understanding of tumor development, tumor microenvironment and effects on peripheral immune activity for the study indications. If tumor biopsies are to be performed prior to first dose of study treatment, cabozantinib treatment will not be given until complete wound healing has occurred; if optional tumor biopsies are to be performed after first dose, cabozantinib treatment must be interrupted for

at least 5 days before optional tumor biopsies are performed and may not be reinitiated until adequate wound healing has occurred.

Analyses may include, but may not be limited to, sequencing of DNA and/or RNA from tissue and/or blood (plasma) to look for genetic/genomic changes (eg, mutations, copy number variation, mutational burden), immunohistochemical (IHC) assessment of biomarker levels in tissue (eg, MET, AXL, PD-L1), and immune cell profiling by fluorescence-activated cell sorting (FACS) analyses. These studies may use conventional as well as novel technology or methodology. The goal is to correlate modulation of these putative biomarkers to clinical outcome as a consequence of cabozantinib and atezolizumab treatment. The determination of PD-L1 levels is for research/exploratory purposes in this study and will not be shared with investigators as these results will not impact therapeutic decisions. [redacted]. Immune cell profiling by FACS may be conducted at selected sites.

In addition, single nucleotide polymorphism (SNP) genotyping may be performed in order to correlate variations in subject genotype with the safety/ tolerability, PK, and/ or pharmacodynamics of cabozantinib and atezolizumab.

The biomarker assessment samples may also be used for diagnostic assay development related to study drug and for the discovery of biomarkers that may prove to be valuable surrogates for clinical response as well as to understand the underlying mechanisms of the disease.

[redacted]

Please refer to Exelixis Pharmacokinetic and Pharmacodynamic/Biomarker Laboratory Manual for specific instructions on sample collection, processing, storage, and shipment.

5.6.9 Tumor Assessment

5.6.9.1 Routine Tumor Assessment

Determination of the study endpoints of ORR, DOR, and PFS will be based on tumor assessment by the investigator per RECIST 1.1 (Appendix G). Additional exploratory efficacy evaluation of immune-related response will include the application of irRECIST (Appendix H). Independent review of tumor assessments may be requested at the discretion of the study sponsor; this would

potentially include submission of all radiographic images from the study (eg, CT/MRI, technetium bone scans) to an independent radiology core laboratory.

Radiographic tumor assessments will include the following (see Table 5-3 for a summary of required tumor assessments by indication):

1. **Chest / Abdomen / Pelvis / Neck:** Unless otherwise described, CT of Chest/Abdomen/Pelvis (CAP) or CT chest and MRI of abdomen/pelvis will be performed in all subjects at screening and every 6 weeks (± 5 days) after initiation of study treatment throughout the first 12 months on study. Upon completion of 12 months on study, these assessments will be performed every 12 weeks (± 7 days). [redacted]

2. **Brain:** MRI (or CT) of the brain will be performed at screening [redacted] for subjects with other tumor indications who have a history or clinical symptoms of brain metastasis. After study treatment initiation MRI (or CT) scans of the brain are only required in subjects with documented, treated brain metastasis or if clinically indicated by signs and symptoms suggestive of new central nervous system (CNS) metastases. Assessments after the first dose of study treatment will be performed every 12 weeks (± 7 days). MRI is the preferred imagining method for brain. If CT of the brain is performed instead of MRI, ambiguous results must be confirmed by MRI unless contraindicated. Subjects without documented brain metastasis during the screening assessment are not required to undergo brain imaging after initiating study treatment unless clinically indicated. In order to meet the eligibility requirements of the study, brain metastasis must have been treated and stable for at least 4 weeks before the first dose of study treatment.

3. **Bone scans**: Technetium bone scans (TBS) will be performed at screening [redacted] for subjects with other tumor indications who have a history or clinical symptoms (ie, bone pain) of bone metastases. After study treatment initiation bone scans are only required in subjects with documented bone lesions or if clinically indicated by signs and symptoms suggestive of new bone metastases. Assessments after the first dose will follow

routine clinical practice (approximately every 12 weeks throughout the first 12 months and every 24 weeks thereafter). Lesions identified on bone scan are not to be recorded as target, non-target, or new lesions. Bone scan findings alone cannot be used for the determination of progression or response in this study and need to be corroborated by CT/MRI. Bone lesions corroborated by CT/MRI must be reported as non-target or new lesions. PET scan or plain films are not considered adequate imaging techniques to measure bone lesions.

Table 5-3: Tumor Assessment Requirements by Indication

Tumor Assessment	Screening	Post-Baseline
Chest/Abdomen/Pelvis CT[a]	All subjects	All Subjects
Neck CT/MRI	[redacted]	[redacted]
Brain MRI (or CT)	[redacted] For other cohorts: subjects with history or clinical symptoms of brain metastases	Subjects with documented brain metastases or clinical symptoms of new brain metastases
Bone Scans	[redacted] For other cohorts: subjects with history or clinical symptoms of bone metastases	Subjects with documented bone lesions or clinical symptoms of new bone metastases

[redacted] CT, computed tomography; [redacted]; MRI, magnetic resonance imaging; [redacted]

[a] CT of chest and MRI of abdomen/pelvis is also permitted.

If there is clinical concern regarding the administration of any contrast, then a non-contrast CAP imaging study may be acceptable as a screening assessment if it clearly demonstrates measurable disease per RECIST 1.1 that can be followed without the need for contrast. In these subjects a post-contrast MRI of the brain must still be performed if required per protocol for the corresponding tumor indication to exclude new metastasis during screening. If at a follow up imaging time point the use of contrast is prohibited (eg, due to acquired impaired renal function) then the same modality should be used without contrast. Low dose non-contrast CT images from combined positron emission tomography/computed tomography (PET/CT) imaging cannot be used for tumor evaluations in this study.

Investigators are encouraged, if any doubt or ambiguities exist about radiographic progression, to continue study treatment if the subject is tolerating it acceptably, repeat radiographic tumor imaging at the next scheduled time point, and delay determination of progression until the findings indicating radiographic progression are unequivocal. Radiographic progression determined by the investigator does not necessarily warrant discontinuation of tumor assessments or study treatment (see Section 3.8). Treatment may continue after radiographic

progression as long as the investigator believes that the subject is still receiving clinical benefit from study treatment and that the potential benefit of continuing study treatment outweighs potential risk. Clinical judgment should be used for allowing treatment beyond radiographic progression. Subjects with clinically significant symptomatic deterioration at the time of radiographic progression may not be suitable for further treatment. [redacted]

Guidance on study treatment continuation or termination of tumor assessment based on subject status is provided in Table 5-4.

Table 5-4: Criteria for Discontinuing Radiographic Assessments:

Subject Status		Action with Radiographic Assessments
**Study Treatment Permanently Discontinued?**	**Investigator-Assessed rPD (per RECIST 1.1) Reached or Initiation of Systemic NPACT?**	
No	No	Continue assessments
No	Yes[a]	Continue assessments (ie, Investigator deems the clinical benefit of continued study drug treatment outweighs the potential risks)
Yes	No	Continue assessments
Yes	Yes	Discontinue assessments

NPACT, non-protocol anticancer therapy; rPD, radiographic progressive disease; RECIST 1.1, Response Evaluation Criteria for Solid Tumors version 1.1.

[a] Investigator-assessed rPD only. Receipt of NPACT is a requirement for study treatment discontinuation (see Section 3.8).

[b] Bone scan evaluations will end on the date of last CT/MRI scan. If the bone scan schedule does not coincide with the last CT/MRI scan, no additional bone scan is needed after the last CT/MRI scan has been performed.

For the purpose of determining radiographic study endpoints for selected cohorts, central review of radiographic images may be conducted by a BIRC. All protocol-required radiographic tumor assessments for these selected cohorts will be sent to the BIRC, which also will review prior radiation history data and prior local therapy information for the purpose of selection of target lesions.

Refer [redacted] for the schedule for these assessments [redacted] Appendix B for the Combination-Therapy Expansion Cohorts, [redacted]

5.6.9.2 Confirmation of Tumor Response and Tumor Progression

For subjects with an overall response of PR or CR per RECIST 1.1 at a given time point, changes in tumor measurements must be confirmed by repeat assessments to be performed no fewer than 4 weeks after the criteria for response are first met. This may be performed at the next scheduled assessment.

In order to identify potential delayed immune-mediated tumor response, subjects with an overall response of PD per RECIST 1.1 who continue with study treatment because of evidence of clinical benefit as assessed by the investigator should have tumor measurement outcomes confirmed ≥ 4 weeks after the initial PD criteria were met. Continuation of study treatment after confirmatory tumor imaging is at the discretion of the investigator. For subjects who continue treatment after the confirmatory tumor imaging, regularly scheduled imaging will continue.

[redacted]

5.6.9.4 Tumor Marker Assessment

For subjects with [redacted] HCC, [redacted], tumor marker samples (ie, [redacted], AFP, [redacted]) will be collected at screening, Day 1 of every third cycle (or every 9 weeks, whichever is earlier) for the first 12 months, and then Day 1 of every fifth cycle (or every 15 weeks, whichever is earlier) until the earlier of initiation of subsequent systemic anticancer therapy or permanent loss to radiographic follow-up (including hospice admission).

[redacted]

The tumor marker assessments will not be used to determine progressive disease or to make study treatment decisions in this study.

[redacted]

5.6.11 Overall Survival

Following study treatment discontinuation each subject will continue to be followed for survival and subsequent anticancer treatment. The investigator (or designee) will make contact (eg, in person or by telephone) with the subject at least as frequently as every 12 weeks (± 14 days) after the Post-Treatment Follow-Up Visit until the subject expires or the Sponsor decides to discontinue collection of these data for the study.

At each contact, the investigator (or designee) will determine if the subject is alive and collect information on nonprotocol anticancer treatments the subject has received. If the subject has died the investigator will record the date and cause of death as best can be determined. All efforts must be undertaken by the study sites to determine the date of death (or date subject last known alive at the time of a data cut-off). This may include, but not necessarily be limited to telephone contacts, communication at study visits, registered letters, and reviews of local obituaries and government death records (if allowed by local laws and regulations).

Refer [redacted] for the schedule for these assessments [redacted] Appendix B for the Combination-Therapy Expansion Cohorts, [redacted]

These assessments are not required for subjects who discontinue study treatment in the Maintenance Phase (such subjects are to be followed per standard of care).

6 TREATMENTS

6.1 Composition, Formulation, and Storage

At study sites, all study medication will be stored as described in the appropriate prescribing information for that country (if applicable) or the pharmacy manual and inventoried in accordance with applicable state and federal regulations.

6.1.1 Investigational Treatment: Cabozantinib

The Sponsor will provide each investigator with adequate supplies of cabozantinib, which will be supplied as 60-mg and 20-mg yellow film-coated tablets. The 60-mg tablets are oval and the 20-mg tablets are round. Doses of 40 mg will comprise two 20-mg tablets. The components of the tablets are listed in Table 6-1.

Table 6-1: Cabozantinib Tablet Components and Composition

Ingredient	Function	% w/w[a]
Cabozantinib Drug Substance (25% drug load as free base)	Active Ingredient	31.68
Microcrystalline Cellulose (Avicel® PH-102)	Filler	38.85
Lactose Anhydrous (60M)	Filler	19.42
Hydroxypropyl Cellulose (EXF)	Binder	3.00
Croscarmellose Sodium (Ac-Di-Sol®)	Disintegrant	6.00
Colloidal Silicon Dioxide	Glidant	0.30
Magnesium Stearate	Lubricant	0.75
Opadry® yellow film coating which includes HPMC 2910/hypromellose 6 cp, titanium dioxide, triacetin, and iron oxide yellow	Film Coating	4.00

[a] weight fraction, expressed in percentage; HPMC, Hydroxypropyl methylcellulose

Refer to the Pharmacy Manual for details on storage and handling of cabozantinib.

6.1.2 [redacted] Combination Treatment: Atezolizumab

Atezolizumab is an Fc-engineered, humanized, monoclonal antibody (non-glycosylated IgG1 kappa immunoglobulin) produced in Chinese hamster ovary cells with a calculated molecular mass of 145 kDa.

The Sponsor will provide each investigator with adequate supplies of atezolizumab, which will be supplied as a 1200 mg/20 mL (60 mg/mL) colorless to slightly yellow solution in single-dose vials. Atezolizumab solution contains the following inactive ingredients: glacial acetic acid, L-histidine, sucrose, and polysorbate 20. Refer to the package insert (or the local label) and the pharmacy manual for additional information and instructions for preparing atezolizumab for

infusion. Solution used as diluent (0.9% NaCl) should be sourced by investigative sites if available and permitted by local regulations.

6.2 Schedule of Treatment

Cabozantinib will be administered orally at assigned daily dose levels of 20, 40, or 60 mg. Atezolizumab will be administered at a standard dosing regimen of 1200 mg as an IV infusion once every 3 weeks.

[redacted]

In the Combination-Therapy Expansion Cohorts, all initially enrolled subjects and any additional subjects enrolled per Extended Enrollment Option 1 will receive cabozantinib 40 mg in combination with the standard dose of atezolizumab 1200 mg. Additional subjects enrolled per Extended Enrollment Option 2 will receive cabozantinib 60 mg in combination with the standard dose of atezolizumab 1200 mg. For details on the Extended Enrollment Options, see Section 3.5.2.1.

[redacted]

[redacted]

[redacted]

- [redacted]

- [redacted]

Further instructions for treatment administration are provided in Sections 6.2.1 and 6.2.2. Special accommodations during the global COVID-19 pandemic are described in Appendix M.

Subjects will receive study treatment as long as they continue to experience clinical benefit as assessed by the investigator or until unacceptable toxicity, the need for subsequent systemic anticancer treatment, or until any other reasons for treatment discontinuation listed in the protocol (Section 3.8). For subjects in combination treatment cohorts, discontinuation of one component of the combination study treatment while continuing to receive the other is allowed but requires Sponsor notification.

For guidance on dose modifications, interruptions, delays, or discontinuations due to AEs, refer to Section 6.5.1.

6.2.1 Administration at the Clinic

Cabozantinib:

The first doses of cabozantinib and atezolizumab will be administered at the clinic; for subjects receiving combination treatment, atezolizumab is to be administered first.

The subject will be fasted (with the exception of water) for at least 2 hours before receiving cabozantinib. Upon completion of the 2-hour fast, the subject will receive the oral dose of cabozantinib with a minimum of 8 oz (240 mL) of water in the clinic and then the subject will continue to fast for 1 hour while under observation to monitor for potential AEs. For cabozantinib dosing on subsequent dosing days refer to Section 6.2.2.

Atezolizumab:

Doses of atezolizumab will always be administered intravenously at the clinic by infusion on Day 1 of each 21-day cycle (-2 days). Cycles may be longer than 3 weeks if atezolizumab treatment is delayed due to toxicity or other reasons.

The infusion of atezolizumab (1200 mg fixed dose) will be prepared according to local prescribing information or the pharmacy manual. The IV administration of atezolizumab can only occur in a clinical setting with staff experienced in managing of IRRs and with access to emergency services. The initial intravenous (IV) infusion of atezolizumab will be given over 60 min (± 15 min) without premedication for potential IRRs or CRS. Subsequent IV infusions may be given over 30 min (± 10 min) if the initial infusion is tolerated. Premedication for infusion-reaction or CRS is allowed after the initial infusion. No bolus or IV push of atezolizumab is allowed. Dose delays will be allowed for toxicities suspected to be due to atezolizumab administration. Atezolizumab infusion requirements and guidance are summarized in Table 6-2.

Table 6-2: Atezolizumab Infusion Requirements and Guidance

First Infusion	Subsequent Infusions
• No premedication is permitted.<br>• Vital signs (blood pressure, pulse, respiratory rate, and temperature) should be recorded within 60 min prior to the infusion.<br>• Atezolizumab should be infused over 60 (± 15) min.<br>• If clinically indicated, vital signs should be recorded during the infusion at 15, 30, 45, and 60 min (± 5 min for all time points) during the infusion and at 30 (± 10) min after the infusion.<br>• Subjects should be informed about the possibility of delayed post-infusion symptoms and instructed to contact their study physician if they develop such symptoms.	• If the subject experienced an infusion-related reaction or cytokine-release syndrome with any previous infusion, premedication with antihistamines, antipyretics, and/or analgesics may be administered for subsequent doses at the discretion of the investigator.<br>• Vital signs should be recorded within 60 min prior to the infusion.<br>• Atezolizumab should be infused over 30 (± 10) min if the previous infusion was tolerated without an infusion-related reaction or cytokine-release syndrome, or 60 (± 15) min if the subject experienced an infusion-related reaction or cytokine release syndrome with the previous infusion.<br>• If the subject experienced an infusion-related reaction or cytokine release syndrome with the previous infusion or if clinically indicated, vital signs should be recorded during the infusion and at 30 (± 5) min after the infusion.

After the completion of IV administration of the first dose of atezolizumab in the clinic, the subject will wait for at least 1 hour before taking cabozantinib. If the subject develops an infusion reaction or CRS, the oral administration of cabozantinib will be delayed or interrupted until the subject has recovered and the investigator believes that it is safe to administer cabozantinib. For management of IRRs and CRS refer to Appendix I.

If the first dose of atezolizumab cannot be given for any reason, no oral treatment with cabozantinib is to be initiated.

6.2.2 Cabozantinib Administration outside the Clinic

Following the first dose of cabozantinib, the subject should take subsequent cabozantinib doses outside the clinic at approximately the same time every day, preferentially before going to bed, and should adhere to the fasting requirements described in this section.

Subjects should fast (with the exception of water) for at least 2 hours after eating the evening meal before taking their dose. After the 2-hour fast and before going to bed, subjects are to take cabozantinib with a full glass of water (minimum of 8 oz or 240 mL) with no more food intake for one hour post-dose. If the subject's schedule requires taking cabozantinib during the day, the subject is to be instructed to follow the same fasting recommendations.

Cabozantinib tablets should not be crushed or chewed. Grapefruit and Seville oranges (and products made from them) should be avoided while being treated with cabozantinib.

Subjects are to be instructed to not make up vomited doses and to maintain the planned dosing schedule. Subjects are not to make up for missed doses if more than 12 hours have elapsed after the time the subject would usually take cabozantinib. In the event of missed doses, subjects are not to take 2 doses to make up for the one the subject missed.

[redacted]

Any unused study treatment must be returned to the study site for drug accountability and disposal.

6.3 Compliance

Subject compliance with outpatient study treatment will be assessed by the site using drug dispensing and return records, progress notes about dose reductions/interruptions, subject interview, [redacted] These data will not be directly recorded in the CRF; rather, the CRF will capture intervals of constant dose and reasons for changes in dose level (eg, a new record completed each time dose level changes, including periods where no dose was taken, and the reason for a dose level change).

6.4 Study Treatment Accountability

The investigator or designee will maintain accurate records of receipt of all study treatment including dates of receipt. In addition, accurate records will be kept regarding when and how much study treatment is dispensed and used by each subject in the study. Reasons for deviation from the expected dispensing regimen must also be recorded. At completion of the study, to satisfy regulatory requirements regarding drug accountability, all unused study treatment will be reconciled and destroyed according to applicable state, federal, and local regulations.

6.5 Safety Considerations

6.5.1 Management of AEs with Dose Reductions and/or Dose Interruptions

Subjects will be monitored for AEs from the time of signing informed consent through 30 days (90 days for AESIs) after the date of the decision to permanently discontinue all study treatment. Subjects will be instructed to notify their physician immediately for any occurring AE. Causality assessment of AEs should include at minimum confounding factors such as disease and

concomitant medications. Adverse event severity will be graded by the investigator according to CTCAE v.4.0.

The following should be taken into consideration in decisions regarding dose modifications (reductions and/or interruptions) for treatment-related side effects:

- Cabozantinib and atezolizumab have class-specific safety profiles based on their mechanism of action but may also cause AEs that overlap. For management of AEs which can be clearly attributed to cabozantinib or atezolizumab in the combination treatment cohorts, independent dose modification for either agent is allowed.
    - Examples of VEGFR TKI associated AEs caused by cabozantinib are hypertension and hand-foot syndrome.
    - Examples of irAEs caused by atezolizumab are pneumonitis and endocrinopathies.

  For AEs without clear attribution to either study treatment, management of toxicity should include dose modifications of both agents per the discretion of the investigator. Examples of overlapping AEs are diarrhea and transaminase increases.

- As a general approach all AEs should be managed with supportive care including both pharmacological and non-pharmacological treatments according to consensus management guidelines at the earliest signs of toxicity considered related to study treatment.
- Study treatment may be continued for mild AEs if appropriate supportive care has been initiated to ameliorate symptoms. Should this be ineffective and toxicities become unacceptable, dose modifications of study treatment should be considered to prevent worsening of toxicity. Moderate to severe AEs usually require dose modifications including dose reductions and/or interruptions.
- Dose interruptions of cabozantinib or atezolizumab for AEs may occur at any time and independently at the discretion of the investigator. If either or both study treatments are interrupted for more than 12 weeks, the sponsor should be contacted to discuss potential treatment continuation.

<u>Cabozantinib:</u>

- The assigned dose for cabozantinib in Cohort 1 of the Dose-Escalation Stage was 40 mg qd. Following review of safety data of the Dose-Escalation Stage by the Cohort Review

Committee, the assigned dose for the Combination-Therapy Expansion Cohorts in the Expansion Stage was determined as 40 mg. The maximum protocol-allowed dose for cabozantinib is 60 mg qd.

- Three dose reduction levels of cabozantinib (40 mg daily, 20 mg daily, and 20 mg qod) are permitted (see Table 6-3).
- [redacted]
- Dose modification criteria for treatment-related AEs of cabozantinib are shown in Table 6-4.
- Dose reinstitution and re-escalation of cabozantinib after dose interruptions and/or reductions:
    - If the subject recovers from his or her toxicities to CTCAE v.4.0 ≤ Grade 1 or to the baseline value (or lower) and the AE was unrelated to cabozantinib, then cabozantinib may be restarted with no change in dose.
    - If the subject recovers from his or her toxicities to ≤ Grade 1 or to the baseline value (or lower) the AE was deemed possibly related to cabozantinib, then cabozantinib may be restarted at a reduced dose (see Table 6-3). Subjects who initiated treatment with cabozantinib at 40 mg and experience a possibly related AE of Grade 1 or 2 severity may be restarted with no dose change after recovery of the toxicities to ≤ Grade 1 or to the baseline value (or lower) if appropriate supportive care can prevent or minimize the risk of the AE.
    - Subjects receiving a dose of 20 mg qod may be restarted at the same dose if deemed safe at the discretion of the investigator. Subjects unable to tolerate a dose of 20 mg qod should discontinue cabozantinib.
    - For subjects in the Combination-Therapy Expansion Cohorts [redacted] in the Expansion Stage, [redacted], re-escalation of cabozantinib to the previous dose after a dose reduction may be allowed at the discretion of the investigator for AEs which have resolved or recovered to Grade 1 (or baseline value) and are deemed tolerable and easily managed by optimized supportive treatment. A minimum two-week interval is needed between resuming with study treatment and the escalation to the next higher dose level. Dose

re-escalation is not allowed during the Dose-Escalation Stage or following a cabozantinib-related dose reduction for Grade 4 AEs affecting major organs (eg, CNS, cardiac, hepatic, renal). Since the primary objective of the Dose-Escalation stage has been met, the remaining active subjects enrolled in the Dose-Escalation Stage will be allowed to re-escalate cabozantinib to the previous dose level after a dose reduction per the above-mentioned protocol guidelines.

- Intra-subject dose escalation of cabozantinib:
  - During the Expansion Stage [redacted] escalation of cabozantinib from 40 mg qd to 60 mg qd is allowed after Sponsor approval for subjects who are tolerating the 40 mg cabozantinib dose level well and have been treated on this dose level for at least 4 weeks. In general, subjects who develop clinically relevant AEs (eg, Grade 3 or 4 AEs) are not allowed to escalate cabozantinib from 40 mg qd to 60 mg qd.
  - For the remaining active subjects enrolled in the Dose-Escalation Stage at 40 mg of cabozantinib, escalation of cabozantinib to 60 mg is allowed per the above-mentioned protocol guidelines.
- Guidelines for the management of specific AEs of cabozantinib such as GI disorders, non-GI fistula formation, hemorrhage, thromboembolic events, hypertension, stomatitis and mucositis, skin disorders, osteonecrosis, proteinuria, nervous system disorders, hepatocellular toxicity, infections and infestations, blood system disorders, fatigue, weight loss, QTc prolongation, electrolyte disorders, endocrine disorders, and respiratory disorders are provided in Section 6.5.2.1.

Table 6-3: Dose Reductions of Cabozantinib (Oral Dosing)

Assigned Starting Dose	First Dose Level Reduction	Second Dose Level Reduction	Third Dose Level Reduction
60 mg daily (qd)	40 mg daily (qd)	20 mg daily (qd)	20 mg every other day (qod)
40 mg daily (qd)	20 mg daily (qd)	20 mg every other day (qod)	No dose reduction permitted
20 mg daily (qd)	20 mg every other day (qod)	No dose reduction permitted	—

Though a dose level of 20 mg every other day (qod) is permitted resulting from dose reductions, that dose level will not be evaluated as an assigned starting dose in either stage of this study. Cabozantinib will be discontinued if a dose of 20-mg cabozantinib every other day (minimum dose) is not tolerated.

Table 6-4: Dose Modifications for Cabozantinib-Associated AEs

CTCAE v.4.0 Grade	Recommended Guidelines for Management[a]
Grade 1 AEs	Add supportive care as indicated. Continue cabozantinib at the current dose level if AE is manageable and tolerable.
Grade 2 AEs which are tolerable and are easily managed	Continue cabozantinib at the current dose level with supportive care.
Grade 2 AEs which are **intolerable** and **cannot be adequately managed**	Cabozantinib should be dose reduced or interrupted.<br>Note: It is recommended that dose interruptions be as brief as possible.
Grade 3 AEs (except clinically non-relevant laboratory abnormalities)	Cabozantinib should be interrupted unless the toxicity can be easily managed with a dose reduction of cabozantinib and optimal medical care.<br>Note: It is recommended that dose interruptions be as brief as possible.
Grade 4 AEs (except clinically non-relevant laboratory abnormalities)	Cabozantinib must be interrupted immediately.<br>In general, cabozantinib should be discontinued unless the following criteria are met:<br>• Subject is deriving clear clinical benefit as determined by the investigator and agreed by the Sponsor<br>• Toxicity can be managed with a dose reduction of cabozantinib following recovery to Grade 1 (or baseline) and optimal medical care<br>Sponsor must be contacted to discuss treatment continuation upon resolution of adverse events.

AE, adverse event.

Note: Cabozantinib dose modification criteria for specific medical conditions are provided in Section 6.5.2.1.

[a] Study treatment dose adjustment is only needed if the toxicity was deemed related to treatment or had an unclear relationship to study treatment.

Atezolizumab:

- The assigned dose for atezolizumab is 1200 mg IV every 3 weeks. Infusion will occur every three weeks (-2 days) on Day 1 of each Cycle.
- Dose interruptions are allowed for atezolizumab (see Table 6-5) but dose reductions are not allowed.
- Dose modification criteria for irAEs and for guidance on reinstituting atezolizumab are shown in Table 6-6.
- If corticosteroids are initiated for treatment of irAEs, they must be tapered over ≥ 1 month to ≤ 10 mg/day oral prednisone or equivalent before atezolizumab can be resumed.
- Guidelines for the management of IRRs and CRS and irAEs of atezolizumab (ie, pneumonitis, hepatitis, diarrhea/colitis, myocarditis, endocrinopathies including hypophysitis, and infection) are provided in Section 6.5.2.2.

Table 6-5: Dose Interruptions of Atezolizumab

Assigned dose	Dose Interruptions
1200-mg atezolizumab IV q3w	At any time to manage unacceptable irAEs

q3w, every 3 weeks; irAE, immune-related adverse events

Table 6-6: Dose Modifications for Atezolizumab-Associated AEs

CTCAE v.4.0 Grade	Recommended Management
Grade 1* or 2 pneumonitis<br>Grade 2 nephritis<br>Hepatic events:<br>**Non-HCC Cohorts:**<br>• Asymptomatic with ALT/AST to > 3.0 to ≤ 5.0 x ULN for > 5 days duration, **or**<br>• ALT/AST increases to > 3 to ≤ 5 x ULN with the appearance of worsening of fatigue, nausea, vomiting, right upper quadrant pain or tenderness, fever, rash, or eosinophilia, **or**<br>• Total bilirubin increases to > 1.5 to ≤ 3.0 x ULN for > 5 days duration<br>**HCC Cohort:**<br>• If AST/ALT is within normal limits at baseline and increases to > 3 × ULN to ≤ 10 × ULN, **or**<br>• If AST/ALT is > ULN to ≤ 3 × ULN at baseline and increases to > 5 × ULN to ≤ 10 × ULN, **or**<br>• If AST/ALT is > 3 × ULN to ≤ 5 × ULN at baseline and increases to > 8 × ULN to ≤ 10 × ULN<br>Grade 2 or 3 diarrhea or colitis<br>Grade 2 or 3 myositis<br>Symptomatic adrenal insufficiency, hypothyroidism, or hyperthyroidism; Grade 2 or 3 hypophysitis; or Grade 3 or 4 hyperglycemia<br>Grade 2 ocular inflammatory toxicity<br>Grade 2 or 3 pancreatitis or increases in amylase and/or lipase levels to > 2.0 - 5.0 x ULN regardless of signs or symptoms or to > 5.0 x ULN<br>Grade 3 or 4 infection<br>Grade 2 infusion-related reactions or cytokine release syndrome<br>Grade 3 rash<br>Suspected Stevens-Johnson syndrome or toxic epidermal necrolysis (any grade)<br>Other Grade 2 or 3 atezolizumab-associated AEs	Delay treatment with atezolizumab<br>Treatment may be resumed in subjects following recovery to Grade 0-1.<br>*(Note: The guidance above applies to all events listed on the left column)*

* For Grade 1 pneumonitis, consider withholding atezolizumab.

<table>
<tr><th>CTCAE v.4.0 Grade</th><th>Recommended Management</th></tr>
<tr><td>Grade 2-4 myocarditis</td><td rowspan="15">Permanently discontinue atezolizumab
(Note: This guidance applies to all events listed on the left column)</td></tr>
<tr><td>Grade 4 myositis and/or recurrent Grade 3 myositis</td></tr>
<tr><td>Grade 3 or 4 pneumonitis</td></tr>
<tr><td>Grade 3 or 4 nephritis</td></tr>
<tr><td>Hepatic events:
Non-HCC Cohorts:
• Symptomatic AST/ALT increases to > 5.0 x ULN, **or**
• Asymptomatic AST/ALT increases to > 5 x ULN for > 2 weeks, **or**
• AST/ALT increases to > 20.0 x ULN, **or**
• Total bilirubin increase to > 3.0 x ULN, **or**
• The following hepatic events that do not resolve to Grade 1 or better within 12 weeks
o Asymptomatic with ALT/AST to > 3.0 to ≤ 5.0 x ULN, **or**
o ALT/AST increases to > 3 to ≤ 5 x ULN with the appearance of worsening of fatigue, nausea, vomiting, right upper quadrant pain or tenderness, fever, rash, or eosinophilia, **or**
o Total bilirubin increases to > 1.5 to ≤ 3.0 x ULN.
HCC Cohort:
AST or ALT > 10 × ULN or total bilirubin > 3 × ULN</td></tr>
<tr><td>Grade 4 diarrhea or colitis</td></tr>
<tr><td>Grade 4 hypophysitis and/or recurrent hypophysitis</td></tr>
<tr><td>Myasthenic syndrome/myasthenia gravis, Guillain-Barré or meningoencephalitis (all grades)</td></tr>
<tr><td>Grade 3 or 4 ocular inflammatory toxicity</td></tr>
<tr><td>Grade 4 or any grade of recurrent pancreatitis</td></tr>
<tr><td>Grade 3 or 4 infusion-related reactions or cytokine release syndrome</td></tr>
<tr><td>Grade 4 rash</td></tr>
<tr><td>Confirmed Stevens-Johnson syndrome or toxic epidermal necrolysis (any grade)</td></tr>
<tr><td>Grade 4 pancreatitis</td></tr>
<tr><td>Other Grade 4 or recurrent Grade 3 atezolizumab-associated AEs</td></tr>
</table>

ALT, alanine aminotransferase; AST, aspartate aminotransferase; irAE, immune-related adverse event; ULN, upper limit of normal.

Note: Additional information for atezolizumab dose modification criteria and treatment recommendations for irAEs and infusion reactions are provided in Section 6.5.2.2.

6.5.2 Warnings, Precautions, Guidelines for Management of Adverse Events

Subjects will be monitored for AEs from the time of signing informed consent through 30 days (90 days for AESIs) after the date of the decision to permanently discontinue treatment. Subjects will be instructed to notify their physician immediately for any occurring AE. Causality assessment of AEs should include at minimum confounding factors such as disease and concomitant medications. Adverse event severity will be graded by the investigator according to CTCAE v.4.0

Management of severe or intolerable adverse reactions may require temporary dose reduction and/or interruption for cabozantinib and/or dose delays of atezolizumab therapy.

6.5.2.1 Cabozantinib

The most frequent AEs experienced by ≥ 20% of subjects treated with cabozantinib in descending order of frequency were diarrhea, fatigue, nausea, decreased appetite, vomiting, weight decreased, PPE, constipation, hypertension, dysgeusia, dysphonia, and asthenia. For a full description of the safety profile of cabozantinib, refer to the Cabozantinib Investigator's Brochure.

Other medically important but less frequent AEs including arterial thrombotic AEs (eg, TIA, and MI) and venous thrombotic AEs (eg, DVT and PE), severe hemorrhagic events, proteinuria, wound healing complications, GI perforation, abscesses including intra-abdominal and pelvic abscess, GI and non-GI fistula formation, osteonecrosis, and RPLS.

Adverse events associated with laboratory abnormalities experienced by ≥ 5% of subjects treated with cabozantinib in descending order of frequency were anemia, AST increased, ALT increased, hypothyroidism, hypokalemia, hypomagnesemia, thrombocytopenia, hypocalcemia, hypophosphatemia, lactate dehydrogenase (LDH) increased, lipase increased, neutropenia, hyponatremia, ALP increased, leukopenia, and hyperglycemia.

Adverse events may occur within the first few weeks in the course of treatment with cabozantinib, as cabozantinib is expected to reach steady state exposure at approximately 2 weeks following first dose. Events that generally have an early onset include hypocalcemia, hypokalemia, thrombocytopenia, hypertension, PPE, abdominal pain, mucosal inflammation, constipation, diarrhea, and vomiting. Adverse events should be managed with supportive care at the earliest signs of toxicity. Dose reductions and treatment interruptions should be considered. Dose reductions are recommended for events that, if persistent, could become serious or intolerable (Table 6-3).

Cabozantinib should be discontinued for the following AEs: visceral perforation or fistula formation, severe hemorrhage, serious arterial thromboembolic events, nephrotic syndrome, hypertensive emergency, persistent uncontrolled hypertension despite optimal medical management, and RPLS.

6.5.2.1.1 Gastrointestinal Disorders

Gastrointestinal perforation, GI fistula, and intra-abdominal and pelvic abscess: After starting treatment with cabozantinib, subjects should be monitored for early signs of GI perforation such as abdominal pain, nausea, emesis, constipation, and fever especially if known risk factors for developing GI perforation or fistula (Turnage and Badgwell 2016) are present. Discontinue cabozantinib and initiate appropriate management in subjects who have been diagnosed with GI perforation or fistula.

Diarrhea: Subjects should be instructed to notify their physician immediately at the first signs of poorly formed or loose stool or an increased frequency of bowel movements. Guidelines for the evaluation and management of diarrhea are shown in Table 6-7. Administration of antidiarrheal/antimotility agents is recommended at the first sign of diarrhea as initial management. Some subjects may require concomitant treatment with more than one antidiarrheal agent. When therapy with antidiarrheal agents does not control the diarrhea to tolerable levels, cabozantinib should be temporarily interrupted or dose reduced. When the diarrhea is controlled, retreatment with cabozantinib may be acceptable per investigator decision. In addition, general supportive measures should be implemented such as continuous oral isotonic hydration, correction of fluid and electrolyte abnormalities, small frequent meals, and stopping lactose-containing products, high-fat meals, and alcohol.

Recurrent or prolonged diarrhea can be associated with anal or perianal skin erosions which increase the risk for anal abscesses, fistulas, or proctitis. Good personal hygiene should be emphasized. Regular examinations of the perianal region should be performed whenever diarrhea has occurred during treatment with cabozantinib. Infections of the perianal region should be treated per local guidelines.

Table 6-7: Management of Diarrhea Associated with Cabozantinib

Status	Management
Tolerable Grade 1-2 (duration < 48 h)	• Continue with study treatment and consider dose reduction<br>• Initiate treatment with an antidiarrheal agent (eg, loperamide 4 mg followed by 2 mg after each episode of diarrhea [maximum: 16 mg loperamide per day])<br>• Dietary modifications (eg, small lactose-free meals, bananas and rice)<br>• Intake of isotonic fluids (1-1.5 L/day)<br>• Re-assess after 24 hours:<br>o Diarrhea resolving to baseline bowel habits: gradually add solid foods and discontinue or decrease antidiarrheal treatment after 12 h diarrhea-free interval<br>o Diarrhea not resolving: Continue/resume antidiarrheal treatment
Intolerable Grade 2, Grade 2 > 48 h, or ≥ Grade 3	• Interrupt study treatment<br>• Ask subject to attend clinic<br>• Rule out infection (eg, stool sample for culture)<br>o Administer antibiotics as needed (eg, if fever or Grade 3-4 neutropenia persists > 24 h)<br>• Administer fluids (1-1.5 L/day orally or IV, as appropriate) for hydration or to correct electrolyte abnormalities<br>• For Grade 3-4 or complicated lower grade diarrhea consider hospitalization and IV hydration<br>• Re-assess after 24 h<br>o Diarrhea resolving to baseline bowel habits or Grade ≤ 1: consider restarting study treatment at reduced dose<br>o Diarrhea not resolving: Start and or continue antidiarrheal treatment (eg, loperamide 4 mg followed by 2 mg after each episode of diarrhea [maximum: 16 mg loperamide per day]). Consider starting second line antidiarrheal or referral to gastroenterologist

Nausea and vomiting: Antiemetic agents are recommended as clinically appropriate for treatment or prophylaxis of nausea and vomiting, along with supportive care. Dehydration and electrolyte abnormalities may be associated with vomiting and monitoring for and correction of fluid and electrolyte disturbances should be implemented. Antiemetic medications should be assessed for potential drug interactions (refer to Section 7.3 for further details).

6.5.2.1.2 Non-Gastrointestinal Fistula

Complications from radiation therapy especially of the thoracic cavity including mediastinum have been identified as a possible predisposing risk factor for non-GI fistula formation in subjects undergoing treatment with VEGF pathway inhibitors.

Discontinue cabozantinib and initiate appropriate management in subjects who have been diagnosed with a non-GI fistula.

6.5.2.1.3 Hemorrhage

Hemorrhagic events, including serious and sometimes fatal events, have been reported with cabozantinib. Subjects should be monitored for bleeding events with serial complete blood counts and physical examination while on study. The risk of hemorrhage in cabozantinib-treated subjects with brain metastases has not been thoroughly analyzed. Subjects enrolled with treated and stable brain metastases should be monitored with a high index of suspicion if symptoms that could be due to a CNS hemorrhage occur.

Cabozantinib should be discontinued in subjects with serious and life-threatening bleeding events or recent hemoptysis (≥ 2.5 mL of red blood).

6.5.2.1.4 Thromboembolic events

Thromboembolic events are frequent in cancer subjects due to procoagulant changes induced by the malignancy or anticancer therapy. DVT and PE have been observed in clinical studies with cabozantinib, including fatal events. Subjects who develop a PE and/or DVT should have study treatment interrupted until therapeutic anticoagulation is established. Treatment with cabozantinib may be resumed in subjects with PE or DVT if it is determined that the event is uncomplicated and that the subject is deriving clinical benefit from cabozantinib treatment and that anticoagulation does not place them at a significant risk that outweighs the benefit of resuming treatment per discretion of the investigator and according to individual protocols. Therapeutic doses of LMWH or specified direct factor Xa oral inhibitors rivaroxaban, edoxaban, or apixaban are allowed for management of thrombotic events in subjects (excluding HCC subjects). Other oral anticoagulants including, but not limited to, coumarin agents (eg, warfarin), platelet inhibitors (eg, clopidogrel), and chronic use of aspirin above low dose levels for cardioprotection per local applicable guidelines are not allowed until 4-weeks after cabozantinib has been permanently discontinued. Subjects with HCC are not allowed to be treated with direct inhibitors of thrombin or factor Xa. See Section 7.2 for additional restrictions on anticoagulation therapy.

Arterial thrombotic events (eg, TIA, MI) have been observed in studies with cabozantinib. Further treatment with cabozantinib should be discontinued in subjects who develop an acute MI, cerebral infarction, or any other clinically significant arterial thromboembolic complication.

6.5.2.1.5 Hypertension

Table 6-8 provides treatment guidelines for hypertension deemed related to cabozantinib. Blood pressure should be monitored in a constant position visit to visit, either sitting or supine in a relaxed setting. Decisions to reduce or interrupt the dose of study treatment must be based on BP readings taken by a medical professional and must be confirmed with a second measurement at least 5 minutes following the first measurement.

Cabozantinib should be discontinued in subjects with hypertension with life threatening consequences or when urgent intervention is indicated, and appropriate medical management should be initiated.

Table 6-8: Management of Hypertension Associated with Cabozantinib

Criteria for Dose Modifications	Treatment/Cabozantinib Dose Modification
> 150 mm Hg (systolic)[a] and < 160 mm Hg<br>OR<br>> 100 mm Hg (diastolic) and < 110 mm Hg	• Optimize antihypertensive medications by adding new or additional antihypertensive medications and/or increase dose of existing medications.<br>• Reduce cabozantinib treatment by one dose level if optimal antihypertensive therapy (usually to include 3 agents) does not result in BP < 150 mm Hg systolic and < 100 mm Hg diastolic<br>• If subject is symptomatic, interrupt cabozantinib treatment and restart only if symptoms have resolved and BP is < 150 mm Hg systolic and < 100 mg Hg diastolic
≥ 160 mm Hg (systolic)<br>OR<br>≥ 110 mm Hg (diastolic)	• Reduce cabozantinib by one dose level or interrupt cabozantinib treatment per investigator discretion. Treatment should be interrupted if upper limits of systolic BP (≥ 160 mm Hg) are sustained and not adequately manageable or if systolic BP is > 180 mm Hg or sustained diastolic BP > 110 mm Hg, or if subject is symptomatic<br>• Add new or additional anti-hypertensive medications and/or increase dose of existing medications and monitor subject closely for hypotension. If optimized antihypertensive therapy (usually to include 3 agents) does not result in BP < 150 mm Hg systolic and < 100 mm Hg diastolic, cabozantinib treatment should be dose reduced further or interrupted<br>• Re-start cabozantinib treatment at reduced dose and re-escalate only if BP falls to and is sustained at < 150 mm Hg systolic and < 100 mm Hg diastolic
Hypertension with life threatening consequences (eg, malignant hypertension, transient or permanent neurologic deficit, hypertensive crisis); urgent intervention indicated	• Discontinue cabozantinib treatment<br>• Initiate appropriate medical management

BP, blood pressure.
[a] The investigator may decide to initiate or adjust antihypertensive treatment at a lower threshold than systolic BP > 150 mm Hg or diastolic BP > 100 mm Hg based on their clinical judgment and assessment of the individual subject.

6.5.2.1.6 Stomatitis and Mucositis

Preventive measures may include a comprehensive oral examination to identify and treat any potential risk for complications before study treatment is initiated. Appropriate correction of local factors should be instituted as indicated, such as modification of ill-fitting dentures and appropriate care of gingivitis. During treatment with cabozantinib, good oral hygiene and

standard local treatments such as non-traumatic and non-irritating cleansing, and oral rinses (eg, with a weak solution of salt and baking soda) should be maintained. Lips should be kept moisturized with lip balm. The use of lipstick, lip-gloss, and Vaseline should be avoided.

Local treatment should be instituted at the earliest onset of symptoms. Obtain bacterial/viral culture if oral infection is suspected and treat infection as clinically indicated.

6.5.2.1.7 Skin and Subcutaneous Tissue Disorders

Wound healing and surgery: Cabozantinib has the potential to cause wound healing complications and wound dehiscence which may even occur long after a wound has been considered healed. Therefore, surgical and traumatic wounds must not only be completely healed prior to starting cabozantinib treatment but must also be monitored for wound dehiscence, wound infection and other signs of impaired wound healing while the subject is being treated with cabozantinib. If dehiscence occurs, cabozantinib treatment should not be restarted until complete healing has taken place.

Treatment with cabozantinib should be stopped at least 28 days prior to scheduled surgery and at least 5 days before an optional tumor biopsy. The decision to resume treatment with cabozantinib after surgery should be based on clinical judgment of adequate wound healing.

Palmar-plantar erythrodysesthesia (PPE; also known as hand-foot syndrome), skin rash (including blister, erythematous rash, macular rash, skin exfoliation, dermatitis acneiform, and papular rash), pruritus, dry skin, erythema, pigmentary changes, and alopecia have been reported with cabozantinib. All subjects on study should be advised on prophylactic measures including the use of emollients, removal of calluses, avoidance of exposure of hands and feet to hot water leading to vasodilatation, protection of pressure-sensitive areas of hands and feet, and use of cotton gloves and socks to prevent injury and keep the palms and soles dry.

Early manifestations include tingling, numbness, mild hyperkeratosis, and symmetrical red and swollen areas on the palms and soles. The lateral sides of the fingers or periungual zones may also be affected. Adequate interventions are required to prevent worsening of skin symptoms such as blisters, desquamations, ulcerations, or necrosis of affected areas. Analgesics may be required for pain control.

Aggressive management of symptoms is recommended, including early dermatology referral. Treatment recommendations in response to PPE are summarized in Table 6-9.

Table 6-9: Management of Palmar-plantar Erythrodysesthesia (PPE) Associated with Cabozantinib

CTCAE v.4.0 Grade	Action To Be Taken
Grade 1	Cabozantinib treatment may be continued at the current dose if PPE is clinically insignificant and tolerable. Otherwise, cabozantinib should be reduced to the next lower dose level[a]. Start urea 20% cream twice daily AND clobetasol 0.05% cream once daily. Reassess at least weekly; if PPE worsens at any time or does not improve after 2 weeks, proceed to the intervention guidelines for Grade 2.
Grade 2	Cabozantinib treatment may be continued if PPE is tolerated. Cabozantinib should be dose reduced or interrupted if PPE is intolerable. Continue urea 20% cream twice daily AND high potency steroid cream (eg, clobetasol 0.05%) once daily and add analgesics (eg, NSAIDs/gamma-aminobutyric acid agonists) for pain control if needed. Reassess at least weekly; if PPE worsens or affects self-care, proceed to the intervention guidelines for Grade 3.
Grade 3	Interrupt cabozantinib treatment until severity decreases to Grade 1 or 0. Continue treatment of skin reaction with high potency steroid cream (eg, clobetasol 0.05%) twice daily AND analgesics. Resume study drug at a reduced dose if PPE recovers to Grade ≤ 1. Discontinue subject from study treatment if PPE does not improve within 6 weeks.

CTCAE, Common Terminology Criteria for Adverse Events; NSAID, non-steroidal anti-inflammatory drug; PPE, palmar plantar erythrodysesthesia.

[a] Permitted dose levels are defined by individual protocols.

6.5.2.1.8 Osteonecrosis

Osteonecrosis has been reported in subjects treated with cabozantinib. Additional risk factors include use of bisphosphonates and denosumab, chemotherapy and anti-angiogenic drugs, use of corticosteroids, local radiotherapy, and dental or orofacial surgery procedures.

Osteonecrosis of the jaw (ONJ) can manifest as jaw pain, osteomyelitis, osteitis, bone erosion, tooth or periodontal infection, toothache, gingival ulceration, or gingival erosion. Persistent pain or slow healing of the mouth or jaw after dental surgery may also be manifestations of osteonecrosis.

Advise subjects regarding oral hygiene practice and to quickly report symptoms to investigator. Caution should be used in subjects receiving bisphosphonates and/or denosumab.

Invasive dental procedures should be avoided. In cases where dental procedures are unavoidable, treatment with cabozantinib should be interrupted for at least 4 weeks prior to the procedure and resumed after complete wound healing has occurred. Bone healing may often require a protracted time.

6.5.2.1.9 Proteinuria

Proteinuria has been reported with cabozantinib. Proteinuria should be monitored by measuring UPCR. Table 6-10 provides treatment guidelines for proteinuria deemed related to cabozantinib.

Cabozantinib should be discontinued in subjects who develop nephrotic syndrome (proteinuria > 3.5 grams per day in combination with low blood protein levels, high cholesterol levels, high triglyceride levels, and edema).

Table 6-10: Management of Proteinuria Associated with Cabozantinib

Severity of Proteinuria (UPCR)	Management of Proteinuria
Non-UC:<br>≤ 1 mg/mg<br>(≤ 113.1 mg/mmol)<br>[redacted]	• No change in cabozantinib treatment or monitoring
Non-UC:<br>> 1 and < 3.5 mg/mg<br>(> 113.1 and < 395.9 mg/mmol)<br>[redacted]	• Consider confirming with a 24-h protein assessment within 7 days<br>• No change in cabozantinib treatment required if UPCR ≤ 2 mg/mg or urine protein ≤ 2 g/24 h on 24-h urine collection.<br>• Dose reduce or interrupt cabozantinib treatment if UPCR > 2 mg/mg on repeat UPCR testing or urine protein > 2 g/24 h on 24-h urine collection. Continue cabozantinib on a reduced dose if UPCR decreases to < 2 mg/mg. Consider interrupting cabozantinib treatment if UPCR remains > 2 mg/mg despite a dose reduction until UPCR decreases to < 2 mg/mg. Restart cabozantinib treatment at a reduced dose after a dose interruption unless otherwise approved by sponsor.<br>• If UPCR > 2 mg/mg, repeat UPCR monitoring within 7 days and once per week. If UPCR < 2 mg/mg on 2 consecutive readings, UPCR monitoring can revert to protocol-specific times. (Second reading is confirmatory and can be done within 1 week of first reading.)
All Tumor Types:<br>≥ 3.5 mg/mg<br>(≥ 395.9 mg/mmol)	• Interrupt cabozantinib treatment pending repeat UPCR monitoring within 7 days and/or 24-h urine protein.<br>• If ≥ 3.5 mg/mg on repeat UPCR monitoring, continue to interrupt cabozantinib treatment and check UPCR every 7 days. If UPCR decreases to < 2 mg/mg, restart cabozantinib treatment at a reduced dose and monitoring of UPCR until it remains < 2 mg/mg on two consecutive measurements. If UPCR monitoring is determined to be stable (< 20% change) for 1 month then continue with UPCR monitoring per protocol or as clinically indicated.
Nephrotic syndrome	• Discontinue cabozantinib treatment

UC, urothelial carcinoma; UPCR, urine protein/creatinine ratio.

6.5.2.1.10 Nervous System Disorders

Cabozantinib appears to represent minimal risk of adverse neurological effects based on nonclinical Good Laboratory Practice (GLP)-compliant toxicology studies. Dysphonia, dysgeusia, headache, dizziness, confusional state, convulsion, depression, memory impairment, hypoesthesia, peripheral neuropathy, insomnia, ataxia, and encephalopathy have been observed in clinical studies with cabozantinib. The development of any new or progressive, unexplained neurological symptoms should be assessed for underlying causes.

RPLS has been reported. RPLS should be considered in any subject presenting with seizures, headache, visual disturbances, confusion or altered mental function. Cabozantinib treatment should be discontinued in subjects with RPLS.

6.5.2.1.11 Infections and Infestations

Infections are commonly observed in cancer subjects. Predisposing risk factor include a decreased immune status (eg, after myelosuppressive anticancer therapies, splenectomy), destructive growth of the underlying malignancy including bone marrow infiltration with suppression of normal hematopoiesis, as well as the presence of IV devices.

Infections and abscesses should be treated with appropriate local care and systemic therapy. Cabozantinib should be interrupted until adequate healing has taken place.

6.5.2.1.12 Blood and Lymphatic System Disorders

Hematological toxicities (ie, neutropenia and thrombocytopenia) and associated complications have been observed after administration of cabozantinib and may be managed with dose interruptions and/or dose reductions. Subjects with hematologic toxicities may require additional or more frequent laboratory tests according to institutional guidelines.

Dose reductions or dose interruptions for hematological toxicities are not mandated but can be applied as clinically indicated. Supportive care for thrombocytopenia or anemia, such as transfusions, may be managed according to institutional guidelines. The use of colony-stimulating growth factors should be considered. Febrile neutropenia or evidence of infection associated with neutropenia must be assessed immediately and treated appropriately and in a timely manner according to institutional guidelines.

6.5.2.1.13 Fatigue

Common causes of fatigue, such as anemia, deconditioning, emotional distress (depression and/or anxiety), poor nutrition, dehydration, sleep disturbance, and hypothyroidism should be

ruled out and treated according to standard of care. Pharmacological management should be considered after disease specific morbidities have been excluded when not prohibited.

6.5.2.1.14 Weight Loss

Anorexia and weight loss should be managed according to local standard of care including nutritional support. Pharmacologic therapy should be considered for appetite enhancement when not prohibited by a particular protocol.

6.5.2.1.15 Corrected QT Prolongation

The effect of orally administered cabozantinib 140 mg qd on QTc interval was evaluated in a placebo-controlled study in subjects with medullary thyroid cancer (MTC). A mean increase in QTcF of 10-15 ms was observed after 4 weeks after initiating cabozantinib treatment. A concentration-QTc relationship could not be definitively established. Changes in cardiac wave form morphology or new rhythms were not observed. No cabozantinib-treated subjects in this study had a QTcF > 500 ms. Review of the larger safety database (approximately 5000 subjects exposed to cabozantinib in clinical trials and in post-marketing experience) confirmed the absence of safety concerns associated with QT prolongation. There were no events of torsades de pointes reported.

Concomitant treatment with strong cytochrome P450 (CYP) 3A4 inhibitors, which may increase cabozantinib plasma concentrations, should be avoided.

If at any time on study there is an increase in QTcF to an absolute value > 500 ms, two additional ECGs must be performed with intervals not less than 3 min apart within 30 min after the initial ECG.

If the average QTcF from the three ECGs is > 500 ms, the following actions must be taken:

- Interrupt cabozantinib treatment
- Immediately notify the Sponsor
- Hospitalize symptomatic subjects (eg, with palpitations, dizziness, syncope, orthostatic hypotension, a significant ventricular arrhythmia on ECG) for a thorough cardiology evaluation and management
- Consider cardiology consultation for asymptomatic subjects for evaluation and management
- Check electrolytes, especially magnesium, potassium and calcium; correct abnormalities as clinically indicated
- Check concomitant medications for any medication that may have contributed to QT prolongation, and if possible, discontinue these medications (http://www.qtdrugs.org)
- Repeat ECG triplicates hourly until the average QTcF is ≤ 500 msec, or otherwise determined by consultation with a cardiologist or appropriate expert.
- Send copies of ECGs to central ECG laboratory for independent read

Subjects with QTc prolongation and symptoms must be monitored closely until the QTc elevation and symptoms have resolved. Cabozantinib treatment may be restarted at a reduced dose level if all of the following conditions are met:

- Symptoms are determined to be unrelated to the QT interval prolongation
- The QTcF value > 500 ms is not confirmed
- Cabozantinib treatment has been interrupted through a minimum of 1 week following the return of the QTcF to ≤ 500 ms.
- QT prolongation can be unequivocally associated with an event other than cabozantinib administration and is treatable/has been resolved
- Sponsor has reviewed all available information and has agreed to the continuation of study treatment

Following reinitiation of study treatment, ECGs must be repeated weekly for 2 weeks, then every 2 weeks for 1 month, then according to the protocol-defined time points.

Cabozantinib treatment must be permanently discontinued if either of the following applies:

- Cardiac evaluation confirms that symptoms are the consequence of QT interval prolongation
- Recurrence of QTcF prolongation after reinitiation of study treatment at a reduced dose

6.5.2.1.16 Electrolyte Disorders

Serum electrolyte disorders including hyponatremia, hypokalemia, hypomagnesemia, and hypophosphatemia have been reported during treatment with cabozantinib, and serum electrolyte levels should be monitored frequently while receiving cabozantinib. Clinically relevant

electrolyte disorders should be managed according to the dose modification guidelines as outlined in Table 6-4 or as clinically indicated. Standard clinical practice guidelines should be used for management of electrolyte disorders and may include oral or IV replacement.

6.5.2.1.17 Endocrine Disorders

Treatment-emergent elevation of thyroid-stimulating hormone (TSH) has been observed with cabozantinib treatment. Currently available data are insufficient to determine the mechanism of thyroid function test alterations and its clinical relevance. Management of thyroid dysfunction (eg, symptomatic hypothyroidism) should follow accepted clinical practice guidelines.

6.5.2.1.18 Hepatocellular Toxicity

Elevations of aminotransferases (ALT and AST) and bilirubin have been observed during treatment with cabozantinib. It is recommended that subjects with elevation of ALT, AST, and/or bilirubin have more frequent laboratory monitoring of these parameters. If possible, hepatotoxic concomitant medications should be discontinued in subjects who develop increased values of ALT, AST, or bilirubin, and other causes (eg, cancer related, infection) should be evaluated.

Management guidelines for hepatotoxicity related to cabozantinib treatment for subjects in **non-HCC** cohorts are provided in Table 6-11.

Table 6-11: Management of Hepatotoxicity Associated with Cabozantinib for Subjects in Non-HCC Cohorts

Severity of Transaminase (ALT or AST) and total bilirubin Elevations	Management
If ALT or AST is within normal limits at baseline and increases to > ULN - 3.0 x ULN<br>OR<br>Total bilirubin increases to > ULN - 1.5 x ULN	• Dose adjustment is usually not required.<br>• Consider discontinuing concomitant hepatotoxic medications and adding supportive care as indicated.
If elevation of ALT or AST to > 3.0 - 5.0 x ULN (total bilirubin ≤ 2.0 x ULN)<br>OR<br>Total bilirubin increases to > 1.5 - 3.0 x ULN (ALT or AST ≤ 3.0 x ULN)	• Interrupt cabozantinib if lasting longer than 1 week.<br>• Restart cabozantinib after lab abnormalities have resolved to CTCAE Grade ≤ 1 or baseline grade at the same dose level prior to dose interruption or one dose level lower at the discretion of the Sponsor.
If ALT or AST increases to > 5.0 to ≤ 8.0 x ULN, (total bilirubin ≤ 2.0 x ULN)<br>OR<br>Total bilirubin increases to > 3.0 x ULN (ALT or AST ≤ 3.0 x ULN)	• Interrupt cabozantinib and consider more frequent monitoring of ALT, AST, and bilirubin.<br>• Restart cabozantinib at a reduced dose after lab abnormalities have resolved to CTCAE Grade ≤ 1 or baseline grade.<br>• Discontinue if lab abnormalities cannot be reversed despite interruption of cabozantinib.
ALT or AST > 8 × ULN<br>OR<br>ALT or AST > 3 × ULN in combination with total bilirubin > 2 × ULN without reasonable other explanation, consistent with DILI	• Discontinue cabozantinib unless these laboratory abnormalities have recovered to Grade 1 or baseline level after an interruption and the Sponsor has approved reinstitution of cabozantinib.

ALT, alanine aminotransferase; AST, aspartate aminotransferase; CTCAE, Common Terminology Criteria for Adverse Events; DILI, drug-induced liver injury.
Note: The guidance for dose modifications for bilirubin abnormalities applies only to subjects without Gilbert's Disease.

Guidelines for management of hepatotoxicity related to cabozantinib treatment for subjects in the HCC cohort is provided in Table 6-12. Since the HCC cohort allows subjects with ALT/AST up to 5 x ULN at study entry, hepatotoxicity AEs are expected to occur relatively frequently due to their disease. These guidelines are to be followed for cabozantinib-associated liver function abnormalities in these subjects.

Table 6-12 Management of Hepatotoxicity Associated with Cabozantinib for Subjects in the HCC Cohort

Severity of Event (Transaminase [ALT or AST] and total bilirubin Elevations)	Management
If ALT or AST is ≤ 3.0 × ULN at baseline and increases to ≥ 5.0 × ULN (total bilirubin ≤ 2.0 x ULN)	• Interrupt cabozantinib<br>• Monitor LFTs more frequently until return to baseline values<br>• If event resolves to baseline, or values stabilize at clinically acceptable levels, cabozantinib may be resumed at a reduced dose
If ALT or AST is > 3.0 × ULN to ≤ 5.0 × ULN at baseline and doubles compared with the baseline values (total bilirubin ≤ 2.0 x ULN)<br>**OR**<br>If ALT or AST is > 3.0 × ULN to ≤ 5.0 × ULN at baseline and increases are less than double but are accompanied by progressive elevations of total bilirubin and/or elevations of coagulation tests (eg, INR) (total bilirubin ≤ 2.0 x ULN)	• Interrupt cabozantinib<br>• Monitor LFTs more frequently until return to baseline values.<br>• If event resolves to baseline, or values stabilize at clinically acceptable levels, cabozantinib may be resumed at a reduced dose
Drug-related ALT or AST > 10 × ULN for > 2 weeks<br>OR<br>Drug-related ALT or AST > 15 × ULN irrespective of duration	• Discontinue cabozantinib unless these laboratory abnormalities have recovered to Grade 1 or baseline level after an interruption and the Sponsor has approved reinstitution of cabozantinib
If hepatic dysfunction is not reversible despite temporary interruption of cabozantinib or drug-related ALT or AST > 3 × ULN in combination with total bilirubin > 2 × ULN without reasonable other explanation, consistent with DILI	• Discontinue cabozantinib

ALT, alanine aminotransferase; AST, aspartate aminotransferase; DILI, drug-induced liver injury; INR, international normalized ratio; LFT, liver function test; ULN, upper limit of normal.

More frequent monitoring of transaminases should be considered and study treatment should be held until the etiology of the abnormalities is determined and these abnormalities are corrected or stabilize to clinically acceptable levels (eg, baseline grade or lower). If hepatic toxicity resolved during a temporary hold and was deemed related to study treatment, then study treatment may be restarted at a reduced dose. Study treatment should be discontinued if hepatic dysfunction is not reversible despite temporary interruption of study treatment.

6.5.2.2 Atezolizumab

The most common AEs reported in ≥ 20% of subjects treated with atezolizumab include fatigue, decreased appetite, nausea, urinary tract infection, pyrexia, and constipation (Tecentriq USPI).

Subjects treated with atezolizumab may also develop IRRs and CRS as well as irAEs such as myocarditis, pneumonitis, hepatitis, colitis, nephritis, endocrinopathies (hypophysitis, thyroid disorders, adrenal insufficiency, Type 1 diabetes), skin disorders, severe cutaneous adverse reactions, ocular events, neurological toxicity (myasthenic syndrome/myasthenia gravis, Guillain-Barré syndrome or meningoencephalitis), pancreatitis, myositis, and embryo-fetal toxicity. Management guidance for atezolizumab-associated AEs is provided in Sections 6.5.2.2.1 to 6.5.2.2.16.

For details on warnings & precautions, possible AEs and management guidance of AEs, and use in special patient populations refer to the local prescribing information of atezolizumab and the atezolizumab Investigator's Brochure.

6.5.2.2.1 Infusion-Related Reactions and Cytokine-Release Syndrome

Infusion-related reactions (IRRs) are known to occur with the administration of monoclonal antibodies and have been reported with atezolizumab. These reactions, which are thought to be due to release of cytokines and/or other chemical mediators, occur within 24 hours of atezolizumab administration and are generally mild to moderate in severity.

Cytokine-release syndrome (CRS) is defined as a supraphysiologic response following administration of any immune therapy that results in activation or engagement of endogenous or infused T cells and/or other immune effector cells. Symptoms can be progressive, always include fever at the onset, and may include hypotension, capillary leak (hypoxia), and end-organ dysfunction (Lee et al 2019). CRS has been well documented with chimeric antigen receptor T-cell therapies and bispecific T-cell engager antibody therapies but has also been reported with immunotherapies that target PD-1 or PD-L1 (Rotz et al 2017; Adashek and Feldman 2019), including atezolizumab.

No premedication is indicated for the administration of Cycle 1 of atezolizumab. However, subjects who experience an IRR or CRS with atezolizumab may receive premedication with antihistamines, antipyretics, and/or analgesics (eg, acetaminophen) for subsequent infusions. Metamizole (dipyrone) is prohibited in treating atezolizumab-associated IRRs because of its potential for causing agranulocytosis.

There may be significant overlap in signs and symptoms of IRRs and CRS, and in recognition of the challenges in clinically distinguishing between the two, consolidated guidelines for the medical management of IRRs and CRS are provided in Appendix I.

Severe COVID-19 appears to be associated with a CRS involving the inflammatory cytokines interleukin (IL)-6, IL-10, IL-2, and IFN-γ (Merad and Martin 2020). If a subject develops suspected CRS during the study, a differential diagnosis should include COVID-19, which should be confirmed or refuted through assessment of exposure history, appropriate laboratory testing, and clinical or radiologic evaluations per Investigator judgment. If a diagnosis of COVID-19 is confirmed, the disease should be managed as per local or institutional guidelines.

For subjects who develop COVID-19 while on study, the Investigator is to evaluate the overall risk-benefit ratio for the subject to determine whether holding study treatment(s) is in the best interest of the subject.

6.5.2.2.2 Immune-Related Pulmonary Events

Dyspnea, cough, fatigue, hypoxia, pneumonitis, and pulmonary infiltrates have been associated with the administration of atezolizumab. Subjects will be assessed for pulmonary signs and symptoms throughout the study and will also have CT scans of the chest performed at every tumor assessment.

All pulmonary events should be thoroughly evaluated for other commonly reported etiologies such as pneumonia or other infections, lymphangitic carcinomatosis, PE, heart failure, chronic obstructive pulmonary disease, or pulmonary hypertension. Management guidelines for pulmonary events are provided in Table 6-13.

Table 6-13: Management Guidelines for Immune-Related Pulmonary Events, Including Pneumonitis

Severity of Event	Management
Grade 1	• Continue atezolizumab and monitor closely<br>• Re-evaluate on serial imaging<br>• Consider subject referral to pulmonary specialist<br>• For Grade 1 pneumonitis, consider withholding atezolizumab.
Grade 2	• Withhold atezolizumab for up to 12 weeks after event onset [a].<br>• Refer subject to pulmonary and infectious disease specialists and consider bronchoscopy or BAL<br>• Initiate treatment with 1–2 mg/kg/day oral prednisone or equivalent.<br>• Resume atezolizumab if event resolves to Grade 1 or better within 12 weeks[a,b]<br>• Permanently discontinue atezolizumab and contact the Sponsor if event does not resolve to Grade 1 or better within 12 weeks[a,b,c]<br>• For recurrent events or events with no improvement after 48-72 hours of corticosteroids, treat as a Grade 3 or 4 event
Grade 3 or 4	• Permanently discontinue atezolizumab and contact the Sponsor[c]<br>• Bronchoscopy or BAL is recommended.<br>• Initiate treatment with 1–2 mg/kg/day IV methylprednisolone or equivalent.<br>• If event does not improve within 48 hours after initiating corticosteroids, consider adding an immunosuppressive agent.<br>• If event resolves to Grade 1 or better, taper corticosteroids over ≥ 1 month.

BAL, bronchoscopic alveolar lavage.

[a] Atezolizumab may be withheld for a period of time beyond 12 weeks after event onset to allow for corticosteroids (if initiated) to be reduced to ≤ 10 mg/day oral prednisone or equivalent. The acceptable length of the extended period of time must be based on an assessment of benefit-risk by the Investigator and in alignment with the protocol requirements for duration of treatment and documented by the Investigator. The Sponsor is available to advise as needed.

[b] If corticosteroids have been initiated, they must be tapered over ≥ 1 month to ≤ 10 mg/day oral prednisone or equivalent before atezolizumab can be resumed.

[c] Resumption of atezolizumab may be considered in subjects who are deriving benefit and have fully recovered from the immune-related event. The decision to rechallenge subjects with atezolizumab should be based on Investigator's assessment of benefit-risk and documented by the Investigator (or an appropriate delegate). The Sponsor is available to advise as needed.

6.5.2.2.3 Immune-Related Colitis or Diarrhea

Immune-related colitis has been associated with the administration of atezolizumab. Management guidelines for diarrhea or colitis are provided in Table 6-14.

All events of diarrhea or colitis should be thoroughly evaluated for other more common etiologies. For events of significant duration or magnitude or associated with signs of systemic inflammation or acute-phase reactants (eg, increased c-reactive protein, platelet count, or bandemia): perform sigmoidoscopy (or colonoscopy, if appropriate) with colonic biopsy, with

three to five specimens for standard paraffin block to check for inflammation and lymphocytic infiltrates to confirm colitis diagnosis.

Table 6-14: Management Guidelines for Immune-Related Diarrhea or Colitis

Severity of Event	Management
Grade 1	• Continue atezolizumab<br>• Initiate symptomatic treatment<br>• Endoscopy is recommended if symptoms persist for > 7 days<br>• Monitor closely
Grade 2	• Withhold atezolizumab for up to 12 weeks after event onset [a].<br>• Initiate symptomatic treatment<br>• Subject referral to GI specialist is recommended<br>• For recurrent events or events that persist > 5 days, initiate treatment with 1–2 mg/kg/day oral prednisone or equivalent<br>• Resume atezolizumab if event resolves to Grade 1 or better within 12 weeks[a,b]<br>• Permanently discontinue atezolizumab and contact the Sponsor if event does not resolve to Grade 1 or better within 12 weeks[a,b,c]
Grade 3	• Withhold atezolizumab for up to 12 weeks after event onset [a].<br>• Refer subject to GI specialist for evaluation and confirmatory biopsy<br>• Initiate treatment with 1–2 mg/kg/day IV methylprednisolone or equivalent and convert to 1–2 mg/kg/day oral prednisone or equivalent upon improvement<br>• Resume atezolizumab if event resolves to Grade 1 or better within 12 weeks[a,b]<br>• Permanently discontinue atezolizumab and contact the Sponsor if event does not resolve to Grade 1 or better within 12 weeks[a,b,c]
Grade 4	• Permanently discontinue atezolizumab and contact the Sponsor[c]<br>• Refer subject to GI specialist for evaluation and confirmatory biopsy.<br>• Initiate treatment with 1–2 mg/kg/day IV methylprednisolone or equivalent and convert to 1–2 mg/kg/day oral prednisone or equivalent upon improvement.<br>• If event does not improve within 48 hours after initiating corticosteroids, consider adding an immunosuppressive agent.<br>• If event resolves to Grade 1 or better, taper corticosteroids over ≥ 1 month.

GI, gastrointestinal; IV, intravenous

[a] Atezolizumab may be withheld for a period of time beyond 12 weeks after event onset to allow for corticosteroids (if initiated) to be reduced to ≤ 10 mg/day oral prednisone or equivalent. The acceptable length of the extended period of time must be based on an assessment of benefit-risk by the Investigator and in alignment with the protocol requirement for duration of treatment and documented by the Investigator. The Sponsor is available to advise as needed.

[b] If corticosteroids have been initiated, they must be tapered over ≥ 1 month to ≤ 10 mg/day oral prednisone or equivalent before atezolizumab can be resumed.

[c] Resumption of atezolizumab may be considered in subjects who are deriving benefit and have fully recovered from the immune-related event. The decision to rechallenge subjects with atezolizumab should be based on Investigator's assessment of benefit-risk and documented by the Investigator (or an appropriate delegate). The Sponsor is available to advise as needed.

6.5.2.2.4 Immune-related Endocrinopathies

Thyroid disorders, adrenal insufficiency, diabetes mellitus, and pituitary disorders have been associated with the administration of atezolizumab. Management guidelines for endocrine events are provided in Table 6-15.

Monitor for signs and symptoms of hypophysitis. Subjects with unexplained symptoms such as headache, fatigue, myalgias, impotence, mental status changes, or constipation should be investigated for the presence of thyroid, pituitary, or adrenal endocrinopathies. The subject should be referred to an endocrinologist if an endocrinopathy is suspected. Thyroid-stimulating hormone (TSH) and free triiodothyronine (T3) and thyroxine (T4) levels should be measured to determine whether thyroid abnormalities are present. Pituitary hormone levels and function tests (eg, TSH, growth hormone, luteinizing hormone, follicle-stimulating hormone, testosterone, prolactin, adrenocorticotropic hormone [ACTH] levels, and ACTH stimulation test) and MRI of the brain (with detailed pituitary sections) may help to differentiate primary pituitary insufficiency from primary adrenal insufficiency.

Table 6-15: Management Guidelines for Endocrine Events

Event	Management
Hypophysitis (pan-hypopituitarism) Grade 2-3	• Withhold atezolizumab for up to 12 weeks after event onset[a]<br>• Refer subject to endocrinologist.<br>• Perform brain MRI (pituitary protocol).<br>• Initiate treatment with 1-2 mg/kg/day IV methylprednisolone or equivalent and convert to 1-2 mg/kg/day oral prednisone or equivalent upon improvement.<br>• Initiate hormone replacement therapy if clinically indicated.<br>• If event resolves to Grade 1 or better, resume atezolizumab.[a,c]<br>• If event does not resolve to Grade 1 or better while withholding atezolizumab, permanently discontinue atezolizumab and contact the Sponsor.[b]<br>• For recurrent hypophysitis, treat as a Grade 4 event.
Hypophysitis (pan-hypopituitarism) Grade 4	• Permanently discontinue atezolizumab and contact the Sponsor.[b]<br>• Refer subject to endocrinologist.<br>• Perform brain MRI (pituitary protocol).<br>• Initiate treatment with 1-2 mg/kg/day IV methylprednisolone or equivalent and convert to 1-2 mg/kg/day oral prednisone or equivalent upon improvement.[c]<br>• Initiate hormone replacement therapy if clinically indicated.
Asymptomatic hypothyroidism	• Continue atezolizumab<br>• Initiate treatment with thyroid replacement hormone<br>• Monitor TSH closely
Symptomatic hypothyroidism	• Withhold atezolizumab<br>• Initiate treatment with thyroid replacement hormone<br>• Monitor TSH closely<br>• Consider subject referral to endocrinologist.<br>• Resume atezolizumab when symptoms are controlled, and thyroid function is improving
Asymptomatic hyperthyroidism	**TSH ≥ 0.1 mU/L and < 0.5 mU/L**:<br>• Continue atezolizumab<br>• Monitor TSH every 4 weeks<br>• Consider subject referral to endocrinologist<br>**TSH < 0.1 mU/L**:<br>• Follow guidelines for symptomatic hyperthyroidism<br>• Consider subject referral to endocrinologist

Event	Management
Symptomatic hyperthyroidism	• Withhold atezolizumab<br>• Initiate treatment with anti-thyroid drug such as methimazole or carbimazole as needed<br>• Consider subject referral to endocrinologist<br>• Resume atezolizumab when symptoms are controlled, and thyroid function is improving<br>• Permanently discontinue atezolizumab and contact the Sponsor for life-threatening immune-related hyperthyroidism[b]
Symptomatic adrenal insufficiency Grade 2–4	• Withhold atezolizumab for up to 12 weeks after event onset[a]<br>• Refer subject to endocrinologist<br>• Perform appropriate imaging<br>• Initiate treatment with 1–2 mg/kg/day IV methylprednisolone or equivalent and convert to 1–2 mg/kg/day oral prednisone or equivalent upon improvement.<br>• Resume atezolizumab if event resolves to Grade 1 or better and subject is stable on replacement therapy (if required) within 12 weeks[a,c]<br>• Permanently discontinue atezolizumab and contact the Sponsor if event does not resolve to Grade 1 or better or subject is not stable on replacement therapy within 12 weeks[a,b,c]
Hyperglycemia Grade 1 or 2	• Continue atezolizumab<br>• Investigate for diabetes. If subject has Type 1 diabetes, treat as a Grade 3 event. If subject does not have Type 1 diabetes, treat as per institutional guidelines.<br>• Monitor for glucose control
Hyperglycemia Grade 3 or 4	• Withhold atezolizumab.<br>• Initiate treatment with insulin.<br>• Evaluate for diabetic ketoacidosis and manage as per institutional guidelines.<br>• Monitor for glucose control.<br>• Resume atezolizumab when symptoms resolve and glucose levels are stable.

IV, intravenous; MRI, magnetic resonance imaging; TSH, thyroid-stimulating hormone.

[a] Atezolizumab may be withheld for a period of time beyond 12 weeks after event onset to allow for corticosteroids (if initiated) to be reduced to ≤ 10 mg/day oral prednisone or equivalent. The acceptable length of the extended period of time must be based on an assessment of benefit-risk by the Investigator and in alignment with the protocol requirement for duration of treatment and documented by the Investigator. The Sponsor is available to advise as needed.

[b] Resumption of atezolizumab may be considered in subjects who are deriving benefit and have fully recovered from the immune-related event. The decision to rechallenge subjects with atezolizumab should be based on Investigator's assessment of benefit-risk and documented by the Investigator (or an appropriate delegate). The Sponsor is available to advise as needed.

[c] If corticosteroids have been initiated, they must be tapered over ≥ 1 month to ≤ 10 mg/day oral prednisone or equivalent before atezolizumab can be resumed.

6.5.2.2.5 Immune-Related Dermatologic Events

Treatment-emergent rash has been associated with atezolizumab. The majority of cases of rash were mild in severity and self-limiting, with or without pruritus. Although uncommon, cases of severe cutaneous adverse reactions such as Stevens-Johnson Syndrome and toxic epidermal necrolysis have been reported with atezolizumab. A dermatologist should evaluate persistent and/or severe rash or pruritus. A biopsy should be considered unless contraindicated. Management guidelines for dermatologic events are provided in Table 6-16.

Table 6-16: Atezolizumab Management Guidance of Immune-Related Dermatologic Events

Severity of Event	Management of Skin Disorder
Grade 1	• Continue atezolizumab.<br>• Consider treatment with topical corticosteroids and/or other symptomatic therapy (eg, antihistamines).
Grade 2	• Continue atezolizumab.<br>• Consider subject referral to dermatologist for evaluation and, if indicated, biopsy.<br>• Initiate treatment with topical corticosteroids.<br>• Consider treatment with higher-potency topical corticosteroids if event does not improve<br>• If unresponsive to topical corticosteroids, consider oral prednisone 0.5 mg/kg/day
Grade 3	• Delay atezolizumab for up to 12 weeks after event onset[a].<br>• Refer subject to dermatologist for evaluation and, if indicated, biopsy.<br>• Initiate treatment with 10 mg/day oral prednisone or equivalent, increasing dose to 1−2 mg/kg/day if event does not improve within 48−72 hours.<br>• Resume atezolizumab if event resolves to Grade 1 or better within 12 weeks.[b,c]<br>• Permanently discontinue atezolizumab and contact Sponsor if event does not resolve to Grade 1 or better within 12 weeks.[a,b,c]
Grade 4	• Permanently discontinue atezolizumab and contact Sponsor.[c]
**Stevens-Johnson syndrome or toxic epidermal necrolysis (any grade)**	**Additional guidance for Stevens-Johnson syndrome or toxic epidermal necrolysis:**<br>• Withhold atezolizumab for suspected Stevens-Johnson syndrome or toxic epidermal necrolysis.<br>• Confirm diagnosis by referring subject to a specialist (dermatologist, ophthalmologist, or urologist as relevant) for evaluation and, if indicated, biopsy.<br>• Follow the applicable treatment and management guidelines above.<br>• If Stevens-Johnson syndrome or toxic epidermal necrolysis is confirmed, permanently discontinue atezolizumab.

[a] Atezolizumab may be withheld for a period of time beyond 12 weeks after event onset to allow for corticosteroids (if initiated) to be reduced to ≤ 10 mg/day oral prednisone or equivalent. The acceptable length of the extended period of time must be based on an assessment of benefit-risk by the Investigator and in alignment with the protocol requirement for duration of treatment and documented by the Investigator. The Sponsor is available to advise as needed.

[b] If corticosteroids have been initiated, they must be tapered over ≥ 1 month to ≤ 10 mg/day oral prednisone or equivalent before atezolizumab can be resumed.

[c] Resumption of atezolizumab may be considered in subjects who are deriving benefit and have fully recovered from the immune-related event. The decision to rechallenge subjects with atezolizumab should be based on Investigator's assessment of benefit-risk and documented by the Investigator (or an appropriate delegate). The Sponsor is available to advise as needed.

6.5.2.2.6 Immune-Related Ocular Events

Treatment-emergent ocular events have been associated with atezolizumab. An ophthalmologist should evaluate visual complaints (eg, uveitis, retinal events). Management guidelines for ocular events are provided in Table 6-17.

Table 6-17: Atezolizumab Management Guidance of Immune-Related Ocular Events

Severity of Event	Management of Ocular Event
Grade 1	• Continue atezolizumab.<br>• Subject referral to ophthalmologist is strongly recommended.<br>• Initiate treatment with topical corticosteroid eye drops and topical immunosuppressive therapy.<br>• If symptoms persist, treat as a Grade 2 event.
Grade 2	• Delay atezolizumab for up to 12 weeks after event onset[a].<br>• Subject referral to ophthalmologist is strongly recommended.<br>• Initiate treatment with topical corticosteroid eye drops and topical immunosuppressive therapy.<br>• Resume atezolizumab if event resolves to Grade 1 or better within 12 weeks.[a,b]<br>• Permanently discontinue atezolizumab and contact the Sponsor if event does not resolve to Grade 1 or better within 12 weeks.[a,b,c]
Grade 3 or 4	• Permanently discontinue atezolizumab and contact the Sponsor.[c]<br>• Refer subject to ophthalmologist.<br>• Initiate treatment with 1−2 mg/kg/day oral prednisone or equivalent.<br>• If event resolves to Grade 1 or better, taper corticosteroids over ≥ 1 month.

[a] Atezolizumab may be withheld for a period of time beyond 12 weeks after event onset to allow for corticosteroids (if initiated) to be reduced to ≤ 10 mg/day oral prednisone or equivalent. The acceptable length of the extended period of time must be based on an assessment of benefit-risk by the Investigator and in alignment with the protocol requirement for duration of treatment and documented by the Investigator. The Sponsor is available to advise as needed.

[b] If corticosteroids have been initiated, they must be tapered over ≥ 1 month to ≤ 10 mg/day oral prednisone or equivalent before atezolizumab can be resumed.

[c] Resumption of atezolizumab may be considered in subjects who are deriving benefit and have fully recovered from the immune-related event. The decision to rechallenge subjects with atezolizumab should be based on Investigator's assessment of benefit-risk and documented by the Investigator (or an appropriate designee). The Sponsor is available to advise as needed.

6.5.2.2.7 Immune-Related Meningoencephalitis

Immune-related meningoencephalitis is an identified risk associated with the administration of atezolizumab. Immune-related meningoencephalitis should be suspected in any subject presenting with signs or symptoms suggestive of meningitis or encephalitis, including, but not limited to, headache, neck pain, confusion, seizure, motor or sensory dysfunction, and altered or depressed level of consciousness. Encephalopathy from metabolic or electrolyte imbalances

needs to be distinguished from potential meningoencephalitis resulting from infection (bacterial, viral, or fungal) or progression of malignancy, or secondary to a paraneoplastic process.

All subjects being considered for meningoencephalitis should be urgently evaluated with a CT scan and/or MRI scan of the brain to evaluate for metastasis, inflammation, or edema. If deemed safe by the treating physician, a lumbar puncture should be performed and a neurologist should be consulted.

Subjects with signs and symptoms of meningoencephalitis, in the absence of an identified alternate etiology, should be treated according to the guidelines in Table 6-18.

Table 6-18: Management Guidelines for Immune-Related Meningoencephalitis

Severity of Event	Management
All grades	• Permanently discontinue atezolizumab and contact the Sponsor[a]<br>• Refer subject to neurologist<br>• Initiate treatment with 1–2 mg/kg/day IV methylprednisolone or equivalent and convert to 1–2 mg/kg/day oral prednisone or equivalent upon improvement<br>• If event does not improve within 48 hours after initiating corticosteroids, consider adding an immunosuppressive agent<br>• If event resolves to Grade 1 or better, taper corticosteroids over ≥ 1 month

IV, intravenous.

[a] Resumption of atezolizumab may be considered in subjects who are deriving benefit and have fully recovered from the immune-related event. The decision to rechallenge subjects with atezolizumab should be based on Investigator's assessment of benefit-risk and documented by the Investigator (or an appropriate delegate). The Sponsor is available to advise as needed.

6.5.2.2.8 Immune-Related Motor and Sensory Neuropathy

Myasthenia gravis and Guillain-Barré syndrome have been observed with single-agent atezolizumab. Patients may present with signs and symptoms of sensory and/or motor neuropathy. Diagnostic work-up is essential for an accurate characterization to differentiate between alternative etiologies. Management guidelines for neurologic disorders are provided in Table 6-19.

Table 6-19: Management Guidelines for Immune-Related Neurologic Disorders

Event	Management
Immune-related neuropathy Grade 1	• Continue atezolizumab<br>• Investigate etiology
Immune-related neuropathy Grade 2	• Withhold atezolizumab for up to 12 weeks after event onset [a]<br>• Investigate etiology and refer subject to neurologist<br>• Initiate treatment as per institutional guidelines<br>• Resume atezolizumab if event resolves to Grade 1 or better within 12 weeks[a,b]<br>• Permanently discontinue atezolizumab and contact the Sponsor if event does not resolve to Grade 1 or better within 12 weeks[a,b,c]
Immune-related neuropathy Grade 3 or 4	• Permanently discontinue atezolizumab and contact the Sponsor[c]<br>• Refer subject to neurologist<br>• Initiate treatment as per institutional guidelines.
Myasthenia gravis and Guillain-Barré syndrome, any grade	• Permanently discontinue atezolizumab and contact the Sponsor[c]<br>• Refer subject to neurologist.<br>• Initiate treatment as per institutional guidelines.<br>• Consider initiation of 1–2 mg/kg/day oral or IV prednisone or equivalent.

IV, intravenous.

[a] Atezolizumab may be withheld for a period of time beyond 12 weeks after event onset to allow for corticosteroids (if initiated) to be reduced to ≤ 10 mg/day oral prednisone or equivalent. The acceptable length of the extended period of time must be based on an assessment of benefit-risk by the Investigator and in alignment with the protocol requirement for duration of treatment and documented by the Investigator. The Sponsor is available to advise as needed.

[b] If corticosteroids have been initiated, they must be tapered over ≥ 1 month to ≤ 10 mg/day oral prednisone or equivalent before atezolizumab can be resumed.

[c] Resumption of atezolizumab may be considered in subjects who are deriving benefit and have fully recovered from the immune-related event. The decision to rechallenge subjects with atezolizumab should be based on Investigator's assessment of benefit-risk and documented by the Investigator (or an appropriate delegate). The Sponsor is available to advise as needed.

6.5.2.2.9 Immune-Related Pancreatitis

Symptoms of abdominal pain associated with elevations of amylase and lipase, suggestive of pancreatitis, have been associated with the administration of atezolizumab. The differential diagnosis of acute abdominal pain should include pancreatitis. Appropriate work-up should include an evaluation for ductal obstruction, as well as serum amylase and lipase tests. Management guidelines for pancreatic events, including pancreatitis, are provided in Table 6-20.

Table 6-20: Management Guidelines for Pancreatic Events, Including Pancreatitis

Event	Management
Amylase and/or lipase > ULN - 1.5 x ULN	• Continue atezolizumab<br>• Monitor amylase and lipase prior to dosing
Amylase and/or lipase > 1.5 - 2.0 x ULN, or > 2.0 - 5.0 x ULN and asymptomatic	**Amylase and/or lipase > 1.5-2.0 x ULN**<br>• Continue atezolizumab<br>• Monitor amylase and lipase weekly<br>• For prolonged elevation (eg, > 3 weeks), consider treatment with 10 mg/day oral prednisone or equivalent<br>**Asymptomatic with amylase and/or lipase > 2.0-5.0 x ULN**<br>• Treat as a Grade 3 event
Amylase and/or lipase > 2.0 - 5.0 x ULN with signs or symptoms, or > 5.0 x ULN	• Withhold atezolizumab for up to 12 weeks after event onset [a].<br>• Refer subject to GI specialist<br>• Monitor amylase and lipase every other day<br>• If no improvement, consider treatment with 1–2 mg/kg/day oral prednisone or equivalent<br>• Resume atezolizumab if event resolves to Grade 1 or better within 12 weeks[a,b]<br>• Permanently discontinue atezolizumab and contact the Sponsor if event does not resolve to Grade 1 or better within 12 weeks[a,b,c]<br>• For recurrent events, permanently discontinue atezolizumab and contact the Sponsor[c]
Immune-related pancreatitis, Grade 2 or 3	• Withhold atezolizumab for up to 12 weeks after event onset [a]<br>• Refer subject to GI specialist<br>• Initiate treatment with 1–2 mg/kg/day IV methylprednisolone or equivalent and convert to 1–2 mg/kg/day oral prednisone or equivalent upon improvement<br>• Resume atezolizumab if event resolves to Grade 1 or better within 12 weeks[a,b]<br>• Permanently discontinue atezolizumab and contact the Sponsor if event does not resolve to Grade 1 or better within 12 weeks[a,b,c]<br>• For recurrent events, permanently discontinue atezolizumab and contact the Sponsor[c]
Immune-related pancreatitis, Grade 4	• Permanently discontinue atezolizumab and contact the Sponsor[c]<br>• Refer subject to GI specialist.<br>• Initiate treatment with 1–2 mg/kg/day IV methylprednisolone or equivalent and convert to 1–2 mg/kg/day oral prednisone or equivalent upon improvement.<br>• If event does not improve within 48 hours after initiating corticosteroids, consider adding an immunosuppressive agent.<br>• If event resolves to Grade 1 or better, taper corticosteroids over ≥ 1 month.

GI, gastrointestinal; IV, intravenous; ULN, upper limit of normal.

[a] Atezolizumab may be withheld for a period of time beyond 12 weeks after event onset to allow for corticosteroids (if initiated) to be reduced to ≤ 10 mg/day oral prednisone or equivalent. The acceptable length of the extended period of time must be based on an assessment of benefit-risk by the Investigator and in alignment with the protocol requirement for duration of treatment and documented by the Investigator. The Sponsor is available to advise as needed.

[b] If corticosteroids have been initiated, they must be tapered over ≥ 1 month to ≤ 10 mg/day oral prednisone or equivalent before atezolizumab can be resumed.

[c] Resumption of atezolizumab may be considered in subjects who are deriving benefit and have fully recovered from the immune-related event. The decision to rechallenge subjects with atezolizumab should be based on Investigator's assessment of benefit-risk and documented by the Investigator (or an appropriate delegate). The Sponsor is available to advise as needed.

6.5.2.2.10 Immune-Related Myocarditis

Immune-related myocarditis has been associated with the administration of atezolizumab. Immune-related myocarditis should be suspected in any subject presenting with signs or symptoms suggestive of myocarditis, including, but not limited to, laboratory (eg, B-type natriuretic peptide) or cardiac imaging abnormalities, dyspnea, chest pain, palpitations, fatigue, decreased exercise tolerance, or syncope. Myocarditis may also be a clinical manifestation of myositis and should be managed accordingly. Immune-related myocarditis needs to be distinguished from myocarditis resulting from infection (commonly viral, eg, in a subject who reports a recent history of gastrointestinal illness), ischemic events, underlying arrhythmias, exacerbation of pre-existing cardiac conditions, or progression of malignancy.

All subjects with possible myocarditis should be urgently evaluated by performing cardiac enzyme assessment, an ECG, a chest X-ray, an echocardiogram, and a cardiac MRI as appropriate per institutional guidelines. A cardiologist should be consulted. An endomyocardial biopsy may be considered to enable a definitive diagnosis and appropriate treatment, if clinically indicated.

Subjects with signs and symptoms of myocarditis, in the absence of an identified alternate etiology, should be treated according to the guidelines in Table 6-21.

Table 6-21: Management Guidelines for Immune-Related Myocarditis

Event	Management
Immune-related myocarditis, Grade 1	• Refer patient to cardiologist<br>• Initiate treatment as per institutional guidelines.
Immune-related myocarditis, Grade 2-4	• Permanently discontinue atezolizumab and contact the Sponsor.[a]<br>• Refer subject to cardiologist<br>• Initiate treatment as per institutional guidelines and consider antiarrhythmic drugs, temporary pacemaker, ECMO, or VAD as appropriate.<br>• Initiate treatment with 1-2 mg/kg/day IV methylprednisolone or equivalent and convert to 1-2 mg/kg/day oral prednisone or equivalent upon improvement.<br>• If event does not improve within 48 hours after initiating corticosteroids, consider adding an immunosuppressive agent.<br>• If event resolves to Grade 1 or better, taper corticosteroids over ≥ 1 month.

ECMO, extracorporeal membrane oxygenation; IV, intravenous; VAD, ventricular assist device.

[a] Resumption of atezolizumab may be considered in subjects who are deriving benefit and have fully recovered from the immune-related event. The decision to rechallenge subjects with atezolizumab should be based on Investigator's assessment of benefit-risk and documented by the Investigator (or an appropriate delegate). The Sponsor is available to advise as needed.

6.5.2.2.11 Immune-Related Nephritis

Immune-related nephritis has been associated with the administration of atezolizumab. Eligible subjects must have adequate renal function. Renal function, including serum creatinine, should be monitored throughout study treatment. Subjects with abnormal renal function should be evaluated and treated for other more common etiologies (including prerenal and postrenal causes, and concomitant medications such as non-steroidal anti-inflammatory drugs). Refer the subject to a renal specialist if clinically indicated. A renal biopsy may be required to enable a definitive diagnosis and appropriate treatment.

Atezolizumab should be withheld for moderate (Grade 2) immune-related nephritis and permanently discontinued for severe nephritis (Grade 3 or 4). Refer subjects to a renal specialist and consider renal biopsy and supportive measures as indicated. Corticosteroids and/or additional immunosuppressive agents should be administered as clinically indicated. Refer to the current atezolizumab Investigator's Brochure for further guidance on the management of immune-related nephritis.

Subjects with signs and symptoms of nephritis, in the absence of an identified alternate etiology, should be treated according to the guidelines in Table 6-22.

Table 6-22: Management Guidelines for Immune-Related Nephritis

Event	Management
Renal event, Grade 1	• Continue atezolizumab.<br>• Monitor kidney function closely, including creatinine and urine protein, until values resolve to within normal limits or to baseline values.
Renal event, Grade 2	• Withhold atezolizumab for up to 12 weeks after event onset. [a]<br>• Refer subject to renal specialist.<br>• Initiate treatment with corticosteroids equivalent to 1−2 mg/kg/day oral prednisone.<br>• If event resolves to Grade 1 or better, resume atezolizumab. [b]<br>• If event does not resolve to Grade 1 or better while withholding atezolizumab, permanently discontinue atezolizumab and contact the Sponsor. [c]
Renal event, Grade 3 or 4	• Permanently discontinue atezolizumab and contact the Sponsor.<br>• Refer subject to renal specialist and consider renal biopsy.<br>• Initiate treatment with corticosteroids equivalent to 1−2 mg/kg/day oral prednisone.<br>• If event does not improve within 48 hours after initiating corticosteroids, consider adding an immunosuppressive agent.<br>• If event resolves to Grade 1 or better, taper corticosteroids over ≥ 1 month.

[a] Atezolizumab may be withheld for a longer period of time (ie, > 12 weeks after event onset) to allow for corticosteroids (if initiated) to be reduced to the equivalent of ≤ 10 mg/day oral prednisone. The acceptable length of the extended period of time must be based on an assessment of benefit-risk by the Investigator and in alignment with the protocol requirement for duration of treatment and documented by the Investigator. The Sponsor is available to advise as needed.

[b] If corticosteroids have been initiated, they must be tapered over ≥ 1 month to the equivalent of ≤ 10 mg/day oral prednisone before atezolizumab can be resumed.

[c] Resumption of atezolizumab may be considered in subjects who are deriving benefit and have fully recovered from the immune-related event. The decision to rechallenge subjects with atezolizumab should be based on Investigator's assessment of benefit-risk and documented by the Investigator (or an appropriate delegate). The Sponsor is available to advise as needed.

6.5.2.2.12 Immune-Related Myositis

Immune-related myositis has been associated with the administration of atezolizumab. Myositis or inflammatory myopathies are a group of disorders sharing the common feature of inflammatory muscle injury; dermatomyositis and polymyositis are among the most common disorders. Initial diagnosis is based on clinical (muscle weakness, muscle pain, skin rash in dermatomyositis), biochemical (serum creatinine-kinase increase), and imaging (electromyography/MRI) features and is confirmed with a muscle biopsy.

Atezolizumab should be withheld for moderate or severe (Grade 2 or 3) immune-related myositis and permanently discontinued for recurrent severe or life-threatening myositis (recurrent Grade 3 or Grade 4). Please refer the subject to rheumatologist and/or neurologist and consider muscle biopsy and supportive measures as clinically indicated. Corticosteroids treatment with 1-2 mg/kg/day IV methylprednisolone or higher-dose bolus if severely compromised (weakness severely limiting mobility, cardiac function, respiratory function, dysphagia) and/or additional immunosuppressive agents should be administered for ≥ Grade 2 events or if the event does not improve after initial corticosteroids. Subjects with possible myositis should be monitored for signs of myocarditis.

Subjects with signs and symptoms of myositis, in the absence of an identified alternate etiology, should be treated according to the guidelines in Table 6-23.

Table 6-23: Management Guidelines for Immune-Related Myositis

Event	Management
Immune-related myositis, Grade 1	• Continue atezolizumab<br>• Refer subject to rheumatologist or neurologist<br>• Initiate treatment as per institutional guidelines
Immune-related myositis, Grade 2	• Withhold atezolizumab for up to 12 weeks after event onset[a] and contact Sponsor.<br>• Refer subject to rheumatologist or neurologist<br>• Initiate treatment as per institutional guidelines<br>• Consider treatment with corticosteroid equivalent to 1-2 mg/kg/day IV methylprednisolone and convert to 1-2 mg/kg/day oral prednisone or equivalent upon improvement.<br>• If corticosteroids are initiated and event does not improve within 48 hours after initiating corticosteroids, consider adding an immunosuppressive agent<br>• If event resolves to Grade 1 or better, resume atezolizumab.[b]<br>• If event does not resolve to Grade 1 or better while withholding atezolizumab, permanently discontinue atezolizumab and contact the Sponsor. [c]
Immune-related myositis, Grade 3	• Withhold atezolizumab for up to 12 weeks after event onset[a] and contact the Sponsor<br>• Refer subject to rheumatologist or neurologist<br>• Initiate treatment as per institutional guidelines<br>• Respiratory support may be required in more severe cases<br>• Initiate treatment with corticosteroid equivalent to 1-2 mg/kg/day IV methylprednisolone or higher dose bolus if subject is severely compromised (eg, cardiac or respiratory symptoms, dysphagia, or weakness that severely limits mobility); convert to 1-2 mg/kg/day oral prednisone or equivalent upon improvement.<br>• If event does not improve within 48 hours after initiating corticosteroids, consider adding an immunosuppressive agent.<br>• If event resolves to Grade 1 or better, resume atezolizumab.[b]<br>• If event does not resolve to Grade 1 or better while withholding atezolizumab, permanently discontinue atezolizumab and contact the Sponsor. [c]<br>• For recurrent events, treat as a Grade 4 event. Permanently discontinue atezolizumab and contact the Sponsor[c]

Event	Management
Immune-related myositis, Grade 4	• Permanently discontinue atezolizumab and contact the Sponsor.[c]<br>• Refer subject to rheumatologist or neurologist<br>• Initiate treatment as per institutional guidelines.<br>• Respiratory support may be required in more severe cases<br>• Initiate treatment with corticosteroid equivalent to 1-2 mg/kg/day IV methylprednisolone or higher dose bolus if subject is severely compromised (eg, cardiac or respiratory symptoms, dysphagia, or weakness that severely limits mobility); convert to 1-2 mg/kg/day oral prednisone or equivalent upon improvement.<br>• If event does not improve within 48 hours after initiating corticosteroids, consider adding an immunosuppressive agent.<br>• If event resolves to Grade 1 or better, taper corticosteroids over ≥ 1 month.

IV, intravenous

[a] Atezolizumab may be withheld for a period of time (ie, > 12 weeks after event onset) to allow for corticosteroids (if initiated) to be reduced to ≤ 10 mg/day oral prednisone or equivalent. The acceptable length of the extended period of time must be based on an assessment of benefit–risk by the Investigator and in alignment with the protocol requirement for duration of treatment and documented by the Investigator. The Sponsor is available to advise as needed..

[b] If corticosteroids have been initiated, they must be tapered over ≥ 1 month to ≤ 10 mg/day oral prednisone or equivalent before atezolizumab can be resumed.

[c] Resumption of atezolizumab may be considered in subjects who are deriving benefit and have fully recovered from the immune-related event. The decision to rechallenge subjects with atezolizumab should be based on Investigator's assessment of benefit–risk and documented by the Investigator (or an appropriate delegate). The Sponsor is available to advise as needed.

6.5.2.2.13 Hemophagocytic Lymphohistiocytosis and Macrophage Activation Syndrome

Immune-related reactions may involve any organ system and may lead to hemophagocytic lymphohistiocytosis (HLH) and macrophage activation syndrome (MAS), which are considered to be potential risks for atezolizumab.

Clinical and laboratory features of severe CRS overlap with HLH, and HLH should be considered when CRS presentation is atypical or prolonged.

Subjects with suspected HLH should be diagnosed according to published criteria by McClain and Eckstein (2014). A subject should be classified as having HLH if five of the following eight criteria are met:

- Fever ≥ 38.5°C
- Splenomegaly
- Peripheral blood cytopenia consisting of at least two of the following:
  - Hemoglobin < 90 g/L (9 g/dL) (< 100 g/L [10 g/dL] for infants < 4 weeks old)
  - Platelet count < 100 × 10^9/L (100,000/μL)
  - ANC < 1.0 × 10^9/L (1000/μL)
- Fasting triglycerides > 2.992 mmol/L (265 mg/dL) and/or fibrinogen < 1.5 g/L (150 mg/dL)
- Hemophagocytosis in bone marrow, spleen, lymph node, or liver
- Low or absent natural killer cell activity
- Ferritin > 500 mg/L (500 ng/mL)
- Soluble interleukin 2 (IL-2) receptor (soluble CD25) elevated ≥ 2 standard deviations above age-adjusted laboratory-specific norms

Subjects with suspected MAS should be diagnosed according to published criteria for systemic juvenile idiopathic arthritis by Ravelli et al (2016). A febrile subject should be classified as having MAS if the following criteria are met:

- Ferritin > 684 mg/L (684 ng/mL)
- At least two of the following:
  - Platelet count ≤ 181 × 10^9/L (181,000/μL)
  - AST ≥ 48 U/L
  - Triglycerides > 1.761 mmol/L (156 mg/dL)
  - Fibrinogen ≤ 3.6 g/L (360 mg/dL)

Subjects with suspected HLH or MAS should be treated according to the guidelines in Table 6-24.

Table 6-24: Management Guidelines for Suspected Hemophagocytic Lymphohistiocytosis or Macrophage Activation Syndrome

Event	Management
Suspected HLH or MAS	• Permanently discontinue atezolizumab and contact the Sponsor.<br>• Consider subject referral to hematologist.<br>• Initiate supportive care, including intensive care monitoring if indicated per institutional guidelines.<br>• Consider initiation of IV corticosteroids, an immunosuppressive agent, and/or anti-cytokine therapy.<br>• If event does not respond to treatment within 24 hours, contact the Sponsor and initiate treatment as appropriate according to published guidelines (La Rosée 2015; Schram and Berliner 2015; La Rosée et al 2019).<br>• If event does not improve within 48 hours after initiating corticosteroids, consider adding an immunosuppressive agent<br>• If event resolves to Grade 1 or better, taper corticosteroids over ≥ 1 month.

HLH, hemophagocytic lymphohistiocytosis; IV, intravenous; MAS, macrophage activation syndrome

6.5.2.2.14 Other Immune-Related Adverse Events

For management of other irAEs not included in Sections 6.5.2.2.1 through 6.5.2.2.16, the following general management guidance should be applied:

- Grade 2 or 3: delay atezolizumab dosing up to 12 weeks until irAE recovers to Grade 0-1 and corticosteroids have been reduced to ≤ 10 mg prednisone or equivalent per day
- Grade 4 or recurrent Grade 3: permanently discontinue atezolizumab

6.5.2.2.15 Embryo-Fetal Toxicity

Based on its mechanism of action, atezolizumab can cause fetal harm when administered to a pregnant woman. Animal studies have demonstrated that inhibition of the PD-L1/PD-1 pathway can lead to increased risk of immune-related rejection of the developing fetus resulting in fetal death. If atezolizumab is used during pregnancy, or if the subject becomes pregnant while taking atezolizumab, advise the subject of the potential risk to a fetus. Advise females of reproductive potential to use highly effective contraception as defined in Appendix K during treatment with atezolizumab and for at least 5 months after the last dose.

6.5.2.2.16 Immune-Related Hepatic Events

Immune-related hepatitis has been associated with the administration of atezolizumab. Eligible subjects must have adequate liver function, as manifested by measurements of total bilirubin and hepatic transaminases, and liver function will be monitored throughout study treatment. Management guidelines for hepatic events in all tumor types except HCC are provided in Table 6-25; management guidelines for hepatic events in HCC subjects (who may enter the study with elevations of AST/ALT up to 5 × ULN at baseline) are provided in Table 6-26.

Subjects with right upper-quadrant abdominal pain and/or unexplained nausea or vomiting should have liver function tests (LFTs) performed immediately and reviewed before administration of the next dose of study drug.

For subjects with elevated LFTs, concurrent medication, viral hepatitis, and toxic or neoplastic etiologies should be considered and addressed, as appropriate.

Table 6-25: Management Guidelines for Hepatic Events (All Tumor Types Except HCC)

Severity of Event	Management
If AST or ALT is within normal limits at baseline and increases to > ULN - 3.0 x ULN<br>OR<br>Total bilirubin increases to > ULN - 1.5 x ULN	• Continue atezolizumab<br>• Monitor LFTs until values resolve to within normal limits or to baseline values
If asymptomatic with elevation of ALT or AST to > 3.0 - 5.0 x ULN<br>OR<br>Total bilirubin increases to > 1.5 - 3.0 x ULN	**All events:**<br>• Monitor LFTs more frequently until return to baseline values<br>**Events of > 5 days' duration:**<br>• Withhold atezolizumab for up to 12 weeks after event onset. [a]<br>• Initiate treatment with 1–2 mg/kg/day oral prednisone or equivalent<br>• Resume atezolizumab if event resolves to Grade 1 or better within 12 weeks[a,b]<br>• Permanently discontinue atezolizumab and contact the Sponsor if event does not resolve to Grade 1 or better within 12 weeks[a,b,c]
ALT or AST increases to > 3 to ≤ 5 x ULN with the appearance of worsening of fatigue, nausea, vomiting, right upper quadrant pain or tenderness, fever, rash, or eosinophilia	• Monitor LFTs more frequently until return to baseline values<br>• Withhold atezolizumab for up to 12 weeks after event onset. [a]<br>• Initiate treatment with 1–2 mg/kg/day oral prednisone or equivalent<br>• Resume atezolizumab if event resolves to Grade 1 or better within 12 weeks[a,b]<br>• Permanently discontinue atezolizumab and contact the Sponsor if event does not resolve to Grade 1 or better within 12 weeks[a,b,c]
If AST or ALT increases to > 5 x ULN<br>OR<br>Total bilirubin increase to > 3.0 x ULN	• Permanently discontinue atezolizumab and contact the Sponsor[c]<br>• Consider subject referral to GI specialist for evaluation and liver biopsy to establish etiology of hepatic injury.<br>• Initiate treatment with 1–2 mg/kg/day oral prednisone or equivalent.<br>• If event does not improve within 48 hours after initiating corticosteroids, consider adding an immunosuppressive agent.<br>• If event resolves to Grade 1 or better, taper corticosteroids over ≥ 1 month.

GI, gastrointestinal; LFT, liver function test.

[a] Atezolizumab may be withheld for a period of time beyond 12 weeks after event onset to allow for corticosteroids (if initiated) to be reduced to ≤ 10 mg/day oral prednisone or equivalent. The acceptable length of the extended period of time must be based on an assessment of benefit-risk by the Investigator and in alignment with the protocol requirements for duration of treatment and documented by the Investigator. The Sponsor is available to advise as needed.

[b] If corticosteroids have been initiated, they must be tapered over ≥ 1 month to ≤ 10 mg/day oral prednisone or equivalent before atezolizumab can be resumed.

[c] Resumption of atezolizumab may be considered in subjects who are deriving benefit and have fully recovered from the immune-related event. The decision to rechallenge subjects with atezolizumab should be based on Investigator's assessment of benefit-risk and documented by the Investigator (or an appropriate delegate). The Sponsor is available to advise as needed.

Table 6-26: Management Guidelines for Hepatic Events (HCC Subjects Only)

Event	Management
If AST or ALT is within normal limits at baseline and increases to > 3 × ULN to ≤ 10 × ULN<br>OR<br>If AST or ALT is > ULN to ≤ 3 × ULN at baseline and increases to > 5 × ULN to ≤ 10 × ULN<br>OR<br>If AST or ALT is > 3 × ULN to ≤ 5 × ULN at baseline and increases to > 8 × ULN to ≤ 10 × ULN	**All events:**<br>• Monitor LFTs more frequently until return to baseline values<br>• Withhold atezolizumab for up to 12 weeks after event onset[a]<br>**Events of > 5 days' duration:**<br>• Consider initiating treatment with 1-2 mg/ kg/day oral prednisone or equivalent<br>• If event resolves to baseline or to Grade 1 or better, resume atezolizumab[b]<br>• If event does not resolve to baseline or to Grade 1 or better while withholding atezolizumab, permanently discontinue atezolizumab and contact the Sponsor[c]
If AST or ALT increases to > 10 × ULN<br>OR<br>Total bilirubin increases to > 3 × ULN	• Permanently discontinue atezolizumab and contact the Sponsor[c]<br>• Consider subject referral to GI specialist for evaluation and liver biopsy to establish etiology of hepatic injury<br>• Initiate treatment with 1–2 mg/kg/day oral prednisone or equivalent<br>• If event does not improve within 48 hours after initiating corticosteroids, consider adding an immunosuppressive agent<br>• If event resolves to baseline, taper corticosteroids over ≥ 1 month

ALT, alanine aminotransferase; AST, aspartate aminotransferase; GI, gastrointestinal; LFT, liver function test; ULN, upper limit of normal.

[a] Atezolizumab may be withheld for a period of time beyond 12 weeks after event onset to allow for corticosteroids (if initiated) to be reduced to ≤ 10 mg/day oral prednisone or equivalent. The acceptable length of the extended period of time must be based on assessment of benefit-risk by the Investigator and in alignment with the protocol requirement for duration of treatment and documented by the Investigator. The Sponsor is available to advise as needed.

[b] If corticosteroids have been initiated, they must be tapered over ≥ 1 month to ≤ 10 mg/day oral prednisone or equivalent before atezolizumab can be resumed.

[c] Resumption of atezolizumab may be considered in subjects who are deriving benefit and have fully recovered from the immune-related event. The decision to rechallenge subjects with atezolizumab should be based on Investigator's assessment of benefit-risk and documented by the Investigator (or an appropriate delegate). The Sponsor is available to advise as needed.

6.5.2.3 Management Guidelines for Hepatic Encephalopathy

Hepatic encephalopathy is a brain dysfunction caused by liver insufficiency. Hepatic encephalopathy is not uncommon in HCC patients and can be due to acute liver failure, portal systemic shunting and cirrhosis (Wong et al 2011, Willson et al 2013).Guidelines for management of hepatic encephalopathy are presented in Table 6-27.

Table 6-27: Management of Hepatic Encephalopathy Associated with Study Treatment

Hepatic Encephalopathy CTCAE Grade	Recommended Guidelines for Management and Dose Modification
Grade 1	Identify and treat any precipitating factor.<br>If symptomatic, consider treatment for AEs.<br>Continue study treatment if asymptomatic and an AE is manageable and tolerable.
Grade 2	Consider treatment for AEs.<br>Continue study treatment if an AE is manageable and tolerable or interrupt study treatment for grade 2 AEs that are intolerable or cannot be adequately managed.<br>If an irAE is suspected, consider initiating steroid treatment.
Grade 3	Consider hospitalization.<br>If an irAE is suspected, consider initiating steroid treatment.<br>Interrupt study treatment; Sponsor must be contacted to discuss treatment continuation upon resolution of AEs.<br>Study treatment may resume if the toxicity can be easily managed with a dose reduction and optimal medical care.
Grade 4	Discontinue study treatment.<br>Consider hospitalization.<br>If an irAE is suspected, consider initiating steroid treatment.

AE, adverse event; CTCAE, Common Terminology Criteria for Adverse Events; HE, hepatic encephalopathy; irAE, immune-related adverse event.

7 CONCOMITANT MEDICATIONS AND THERAPIES

7.1 Allowed Therapy

- Antiemetics and antidiarrheal medications are allowed prophylactically according to standard clinical practice if clinically indicated.

- Granulocyte colony-stimulating factors (G-CSF or GM-CSF) are allowed if used per clinical guidelines (eg, ASCO or ESMO guidelines).

- Bisphosphonates and/or denosumab can be used to control bone loss or hypercalcemia if the benefit outweighs the risk per the investigator's discretion (Section 6.5.2.1.8).

  Note: osteonecrosis of the jaw has been reported in subjects using bisphosphonates and/or denosumab. Oral examinations are recommended at screening to determine eligibility and periodically during the study. In addition, subjects should be advised regarding oral hygiene practice and to quickly report symptoms to the investigator. Frequent monitoring for potentially overlapping toxicities with study treatment is recommended.

- Transfusions and hormone replacement should be utilized as indicated by standard clinical practice.

- Topical, inhaled, intranasal, and/or intraarticular corticosteroids are allowed. Prophylactic systemic corticosteroids are allowed for control of infusion reactions and must be tapered to a dose level ≤ 10 mg/day of prednisone equivalent before next atezolizumab administration. If corticosteroids are initiated for the treatment of irAEs (except transfusion reactions), they must be tapered over ≥ 1 month to ≤ 10 mg/day oral prednisone or equivalent before atezolizumab can be resumed unless otherwise specified in the AE management guidance (Section 6.5) or the atezolizumab Investigator's Brochure. Prophylactic steroid treatment for subjects with contrast allergies prior to tumor imaging is allowed. A short course of systemic steroids for acute medical conditions (such as worsening of COPD and gout flare) is allowed but steroids must be tapered to ≤ 10 mg/day oral prednisone or equivalent before the subsequent dose of atezolizumab. Local injections of steroids for local medical conditions may be allowed upon Sponsor's approval. Adrenal replacement steroid doses > 10 mg daily prednisone equivalent are permitted in the absence of active autoimmune disease.

- Individualized anticoagulation therapy with heparin or specified direct factor Xa inhibitors rivaroxaban, edoxaban, or apixaban is allowed if it can be provided safely and effectively under the following circumstances:

  At the time of first dose of study treatment:

  - *Low dose low molecular weight heparins (LMWH) for prophylactic use* are allowed if clinically indicated and the benefit outweighs the risk per the investigator's discretion.
  - *Therapeutic doses of LMWH or specified direct factor Xa inhibitors rivaroxaban, edoxaban, or apixaban* are allowed in subjects (excluding HCC subjects) if the subject has no evidence of brain metastasis, has been on a stable dose of the anticoagulant for at least 1 week, and has had no clinically significant hemorrhagic complications from the anticoagulation regimen or the tumor. See Section 7.2 for prohibited anticoagulants.
  - *Subjects with HCC may be treated with therapeutic LMWH but must have a screening platelet count of > 100,000/µL* (no oral anticoagulants are allowed in subjects with HCC).

  After first dose of study treatment:

  - *Low dose low molecular weight heparins (LMWH) for prophylactic use* are allowed if clinically indicated and the benefit outweighs the risk per the investigator's discretion.
  - *Therapeutic doses of LMWH or specified direct factor Xa oral inhibitors rivaroxaban, edoxaban, or apixaban* are allowed in subjects (excluding HCC subjects) if clinically indicated (eg, for the treatment of DVT), and the benefit outweighs the risk per the investigator's discretion. For management of thromboembolic complications while on study, refer to Section 6.5.2.1.4. See Section 7.2 for prohibited anticoagulants.
  - *Subjects with HCC may be treated with therapeutic LMWH* (no oral anticoagulants are allowed in subjects with HCC).

  Considerations for Use of Anticoagulation Therapy:

  Accepted clinical guidelines regarding appropriate management while receiving any kind of anticoagulation therapy must be followed. This includes, but is not limited to, subject education regarding the potential adverse drug reactions, monitoring laboratory parameters,

and dose adjustments (eg, due to kidney dysfunction). Caution is warranted in settings associated with an increased risk for bleeding such as gastrointestinal cancers, urothelial cancers, gastrointestinal mucosal abnormality (eg, mucositis), renal or hepatic impairment, thrombocytopenia, arterial hypertension, or prior history of gastrointestinal bleed. For direct factor Xa inhibitors, the potential for drug-drug interaction with other concomitant medications, as well as gastrointestinal absorption, should be considered. Aspirin and other nonsteroidal anti-inflammatory drugs (NSAIDs) should not be used concomitantly with heparin or factor Xa inhibitors due to the increased risk for bleeding complications. The risks and benefits of the use of anticoagulants should be reassessed on a regular basis. For more information regarding the use of anticoagulants, refer to the prescribing information of the anticoagulants and accepted clinical practice guidelines.

- Considerations for use of COVID-19 vaccine: At this time, there are limited data available for the use of COVID-19 vaccines in specific subsets of individuals such as the immunocompromised and cancer patients, including those receiving anticancer therapy such as cabozantinib and atezolizumab. The decision to proceed with vaccination for subjects enrolled in this clinical study rests with the treating physician and the subject and should be taken after consideration of all safety precautions provided by the manufacturers of the vaccines and local health authorities. Please note, immunizations with live, attenuated COVID-19 vaccines or any experimental vaccines are not allowed. Exelixis will monitor all available information and provide additional guidance as appropriate.

Potential drug interactions with cabozantinib are summarized in Section 7.3.1. The drug interaction potential of atezolizumab is unknown. Refer to the local prescribing information and the atezolizumab Investigator's Brochure.

7.2 Prohibited or Restricted Therapy

The following therapies are <u>prohibited</u> until study treatment has been permanently discontinued:

- Any investigational agent or investigational medical device.
- Oral anticoagulants with coumarin agents (eg, warfarin), direct thrombin and factor Xa inhibitors (unless otherwise specified in Section 7.1), platelet inhibitors (eg, clopidogrel), and chronic use of aspirin above low dose levels for cardioprotection per local applicable guidelines, until 4 weeks after cabozantinib has been permanently discontinued.
    - No oral anticoagulants are allowed in subjects with HCC.
- Any nonprotocol systemic anticancer treatment (eg, chemotherapy, hormonal therapy, immunotherapy, radionuclides, drugs or herbal products used specifically for the treatment of the cancer under investigation) [redacted]
- Immunosuppressive agents including immunosuppressive doses of systemic corticosteroids with exceptions as stated in Section 7.1.
- Live vaccines are prohibited while on study and until 5 months after last atezolizumab dose (eg, intranasal influenza, measles, mumps, rubella, oral polio, Bacillus Calmette-Guérin, yellow fever, varicella, and TY21a typhoid vaccines) in the Combination-Therapy Expansion Cohorts and exploratory SAA Cohort. The use of inactivated (killed) vaccines for the prevention of infectious disease is allowed.
- Metamizole (dipyrone) because of its potential for causing agranulocytosis.

The following therapies should be <u>avoided</u> until study treatment has been permanently discontinued or until otherwise specified:

- Local anticancer treatment including palliative radiation, ablation, embolization, or surgery with impact on tumor lesions should not be performed until radiographic progression per RECIST 1.1 has been established. If clinically unavoidable the investigator should consult the Sponsor prior to the procedure for safety guidance.

- Erythropoietic stimulating agents (eg, epoetin alfa and darbepoetin alfa) should not be used based on a report of increased risk of tumor recurrence/progression associated with erythropoietin (Wright et al 2007).

- Concomitant medications that are known to prolong the QTc interval should be avoided in subjects who receive cabozantinib until they have permanently discontinued cabozantinib treatment (refer to http://www.qtdrugs.org for a list of drugs which have the potential to prolong the QTc interval).

- Chronic co-administration of cabozantinib with strong inducers of the CYP3A4 family (eg, phenytoin, carbamazepine, rifampin, rifabutin, rifapentine, phenobarbital, and St. John's Wort) may significantly decrease cabozantinib concentrations and should be avoided. Selection of alternate concomitant medications with no or minimal CYP3A4 enzyme induction potential is recommended for subjects receiving cabozantinib. See Appendix J for further details.

- Caution must be used when discontinuing treatment with a strong CYP3A4 inducer in a subject who has been concurrently receiving a stable dose of cabozantinib, as this could significantly increase the exposure to cabozantinib.

- Co-administration of cabozantinib with strong inhibitors of the CYP3A4 family (eg, boceprevir, conivaptan, posaconazole, ketoconazole, itraconazole, clarithromycin, atazanavir, indinavir, nefazodone, nelfinavir, saquinavir, ritonavir, lopinavir, telaprevir, telithromycin, and voriconazole) may increase cabozantinib concentrations and should be avoided. Grapefruit, star fruit, and Seville oranges may also increase plasma concentrations of cabozantinib and should be avoided in subjects receiving cabozantinib. See Appendix J for further details.

Additional information on potential drug interactions with cabozantinib is provided in Section 7.3.1.

Refer to the local prescribing information and the atezolizumab Investigator's Brochure for drugs to be avoided when taking atezolizumab.

7.3 Potential Drug Interactions

7.3.1 Potential Drug Interactions with Cabozantinib

Cytochrome P450: Data from a clinical drug interaction study (Study XL184-008) show that clinically relevant steady-state concentrations of cabozantinib appear to have no marked effect on the area under the plasma concentration-vs-time curve (AUC) of co-administered rosiglitazone, a CYP2C8 substrate. Therefore, cabozantinib is not anticipated to markedly inhibit CYP2C8 in the clinic, and by inference, is not anticipated to markedly inhibit other CYP450 isozymes that have lower [I]/Ki values compared with CYP2C8 (ie, CYP2C9, CYP2C19, CYP2D6, CYP1A2, and CYP3A4). In vitro data indicate that cabozantinib is unlikely to induce cytochrome P450 enzymes, except for possible induction of CYP1A1 at high cabozantinib concentrations (30 μM).

Cabozantinib is a CYP3A4 substrate and a weak substrate for CYP2C9 (but not a CYP2D6, CYP2C8, CYP2C19, CYP2B6, or CYP1A2 substrate), based on data from in vitro studies. Results from a clinical pharmacology study, XL184-006, showed that concurrent administration of cabozantinib with the strong CYP3A4 inducer, rifampin, resulted in an approximately 77% reduction in cabozantinib exposure (AUC values) after a single dose of cabozantinib in healthy volunteers. Chronic co-administration of cabozantinib with strong inducers of the CYP3A4 family (eg, phenytoin, carbamazepine, rifampin, rifabutin, rifapentine, phenobarbital, and St. John’s Wort) may significantly decrease cabozantinib concentrations. The chronic use of strong CYP3A4 inducers should be avoided. Other drugs that induce CYP3A4 should be used with caution because these drugs have the potential to decrease exposure (AUC) to cabozantinib. Selection of alternate concomitant medications with no or minimal CYP3A4 enzyme induction potential is recommended.

Results from a clinical pharmacology study, XL184-007, showed that concurrent administration of cabozantinib with the strong CYP3A4 inhibitor, ketoconazole, resulted in a 38% increase in the cabozantinib exposure (AUC values) after a single dose of cabozantinib in healthy volunteers. Co-administration of cabozantinib with strong inhibitors of the CYP3A4 family (eg, boceprevir, conivaptan, posaconazole, ketoconazole, itraconazole, clarithromycin, atazanavir, indinavir, nefazodone, nelfinavir, saquinavir, ritonavir, lopinavir, telaprevir, telithromycin, and voriconazole) may increase cabozantinib concentrations. Grapefruit, star fruit and Seville oranges may also increase plasma concentrations of cabozantinib and should be avoided. Strong CYP3A4 inhibitors should be avoided and other drugs that inhibit CYP3A4 should be used with caution because these drugs have the potential to increase exposure (AUC)

to cabozantinib. Selection of alternate concomitant medications with no or minimal CYP3A4 enzyme inhibition potential is recommended.

For lists of substrates, inducers, and inhibitors of selected CYP450 isozyme pathways, refer to Appendix J.

Protein Binding: Cabozantinib is highly bound (≥ 99.7%) to human plasma proteins. Therefore, highly protein bound drugs should be used with caution with cabozantinib because there is a potential displacement interaction that could increase free concentrations of cabozantinib and/or a co-administered highly protein-bound drug (and a corresponding increase in pharmacologic effect).

Other Interactions: Food may increase exposure levels of cabozantinib by 57%, fasting recommendations should be followed. In vitro data suggest that cabozantinib is unlikely to be a substrate for P-glycoprotein, but it does appear to have the potential to inhibit the P-glycoprotein transport activity. Therefore, cabozantinib may have the potential to increase plasma concentrations of co-administered substrates of P-glycoprotein. Additional details related to these overall conclusions can be found in the investigator brochure.

Administration of the proton pump inhibitor (PPI) esomeprazole resulted in no clinically-relevant effect on cabozantinib plasma PK in healthy volunteers. Therefore, concomitant use of gastric pH modifying agents (ie, PPIs, H_2 receptor antagonists, and antacids) is not contraindicated in subjects administered cabozantinib.

Additional details regarding potential drug interactions with cabozantinib can be found in the investigator brochure.

7.3.2 Potential Drug Interactions with Atezolizumab

Cytochrome P450 enzymes, as well as conjugation/glucuronidation reactions, are not involved in the metabolism of atezolizumab. No drug interaction studies for atezolizumab have been conducted. There are no known interactions with other medicinal products or other form of interactions. For additional details refer to the local prescribing information and the atezolizumab Investigator's Brochure.

8 SAFETY

8.1 Adverse Events and Laboratory Abnormalities

8.1.1 Adverse Events

An AE is any untoward medical occurrence in a patient or clinical investigation subject participating in a clinical study who may have been administered an investigational product, regardless of whether or not the event is assessed as related to the study treatment. An AE can therefore be any unfavorable and unintended sign (including an abnormal laboratory finding, ECG findings, or vital signs), symptom, or disease temporally associated with the use of an investigational product, regardless of whether or not the event is assessed as related to the investigational product. This requirement includes specific events or symptoms associated with cancer progression or general clinical deterioration to ensure potential toxicities are not overlooked. Radiographic progression without associated clinical sequelae is not considered an AE: terms such as "disease progression" should be avoided. Pre-existing medical conditions that worsen during a study will be recorded as AEs. Abnormal laboratory values, ECG findings, or vital signs are to be recorded as SAEs if they meet the criteria described in Section 8.2.

All untoward events that occur after informed consent through 30 days (90 days for AESIs) after the date of the decision to permanently discontinue study treatment are to be recorded in source documents by the investigational site. See the CRF Completion Guidelines for instructions on entering these data on Medical History and/or AE CRFs and Section 8.2 for SAE reporting requirements. The date of the decision to discontinue study treatment is defined for each subject as the later of (a) the date of the decision of the investigator to permanently discontinue study treatment or (b) the date of the last dose of study treatment taken by the subject.

At each scheduled and unscheduled visit, AEs are to be identified and assessed based upon study procedures, routine and symptom-directed clinical investigations, and subject query/report.

Assessment of the relationship of the AEs to study treatment by the investigator will be based on the following two definitions:

- **Not Related**: An event is assessed as not related to study treatment if it is attributable to another cause and/or there is no evidence to support a causal relationship.
- **Related**: An event is assessed as related to study treatment when there is a reasonable possibility that study treatment caused the event. Reasonable possibility means there is evidence to suggest a causal relationship between study treatment and the event. This event is called a suspected adverse reaction. A suspected adverse reaction implies a lesser degree of certainty about causality than adverse reaction, which means any AE caused by a drug.

8.1.2 Laboratory Abnormalities

All laboratory data required by this protocol and any other clinical investigations will be reviewed. Any abnormal value that leads to a change in subject management (eg, dose reduction or delay or requirement for additional medication or monitoring) or that is considered to be of clinical significance by the investigator will be reported as an AE or SAE as appropriate, unless this value is consistent with the subject's present disease state or is consistent with values obtained prior to entry into the study.

8.2 Serious Adverse Events

The SAE definition and reporting requirements are in accordance with the International Conference on Harmonisation (ICH) Guideline for Clinical Safety Data Management: Definitions and Standards for Expedited Reporting, Topic E2A.

An SAE is defined as any untoward medical occurrence that at any dose

- Results in death.
- Is immediately life-threatening (ie, in the opinion of the investigator, the AE places the subject at immediate risk of death; it does not include a reaction that, had it occurred in a more severe form, might have caused death).
- Requires inpatient hospitalization or results in prolongation of an existing hospitalization.
- Results in significant incapacity or substantial disruption of the ability to conduct normal life functions.
- Is a congenital anomaly or birth defect.
- Is an important medical event that may not be immediately life-threatening, result in death, or require hospitalization, but may be considered an SAE when, based upon appropriate medical judgment, it jeopardizes the subject or may require medical or surgical intervention to prevent one of the outcomes listed above.

As soon as an investigator becomes aware of an AE that meets the criteria for an SAE, the investigator will document the SAE to the extent that information is available.

SAEs, regardless of causal relationship, must be reported to the Sponsor or designee within 24 hours of the investigator's knowledge of the event by submitting the completed SAE report form and any other pertinent SAE information as indicated on the SAE Reporting form (or in the

SAE Reporting form Completion Guidelines) and confirming the report was received. Forms for reporting SAEs and contact information will be provided to the study sites.

SAEs that must be recorded on an SAE Reporting form include the following:

- All SAEs that occur after informed consent and through 30 days (90 days for AESIs) after the date of the decision to permanently discontinue study treatment (or the date the subject is deemed to be a screen failure).
- Any SAEs assessed as related to study treatment or study procedures, even if the SAE occurs more than 30 days after the date of the decision to permanently discontinue study treatment.

Note: If the subject does not meet the eligibility criteria during screening, then SAEs only need to be reported from the time the subject signs the informed consent until the day when the subject has been determined to not be eligible for study participation.

SAEs that occur after informed consent through 30 days (90 days for AESIs) after the date of the decision to permanently discontinue of study treatment must also be recorded on the CRF page.

The minimum information required for SAE reporting includes identity of investigator, site number, subject number, and an event description. Other important information requiring timely reporting are the SAE term(s), the reason why the event is considered to be serious (ie, the seriousness criteria), and the investigator's assessment of the relationship of the event to study treatment. Additional SAE information including medications or other therapeutic measures used to treat the event, action taken with the study treatment because of the event, and the outcome/resolution of the event will be recorded on the SAE form.

In all cases, the investigator should continue to monitor the clinical situation and report all material facts relating to the progression or outcome of the SAE. Furthermore, the investigator may be required to provide supplementary information as requested by the Sponsor's Drug Safety personnel or designee.

When reporting SAEs, the following additional points will be noted:

- When the diagnosis of an SAE is known or suspected, the investigator will report the diagnosis or syndrome as the primary SAE term, rather than as signs or symptoms. Signs and symptoms may then be described in the event description.

- Death will not be reported as an SAE, but as an outcome of a specific SAE, unless the event preceding the death is unknown. Terms of "Unexplained Death" or "Death from unknown origin" may be used when the cause is unknown. In these circumstances the cause of death must be investigated and the diagnosis amended when the etiology has been identified. If an autopsy was performed, the autopsy report should be provided.

- While most hospitalizations necessitate reporting of an SAE, some hospitalizations do not require SAE reporting, as follows:

    - Elective or previously scheduled surgeries or procedures for preexisting conditions that have not worsened after initiation of treatment (eg, a previously scheduled ventral hernia repair). SAEs must, however, be reported for any surgical or procedural complication resulting in prolongation of the hospitalization.

    - Prespecified study hospitalizations for observation.

    - Events that result in hospital stays of fewer than 24 hours and that do not require admission (eg, an emergency room visit for hematuria that results in a diagnosis of cystitis and discharge to home on oral antibiotics).

8.2.1 Regulatory Reporting

The Sponsor's Drug Safety group (or designee) will process and evaluate all SAEs and AESIs as the reports are received. For each SAE received, the Sponsor will make a determination as to whether the criteria for expedited reporting to relevant regulatory authorities have been met.

The Sponsor's Drug Safety group (or designee) will assess the expectedness of each SAE to the study treatment using the current reference safety information (RSI) for each study drug.

The Sponsor or its designee is responsible for reporting relevant SAEs to the relevant regulatory authorities, and participating investigators, in accordance with FDA regulations (21 Code of Federal Regulations [CFR] 312.32), ICH guidelines, European Clinical Trials Directive (Directive 2001/20/EC), and/or local regulatory requirements.

Reporting of SAEs by the investigator to his or her IRB/ECs will be done in accordance with the standard operating procedures and policies of the IRB/EC. Adequate documentation must be maintained showing that the IRB/EC was properly notified.

8.3 Adverse Events of Special Interest for Atezolizumab

Adverse events of special interest (AESIs) for atezolizumab consist of immune-mediated adverse events associated with ICIs, cases of potential drug-induced liver injury, and suspected transmission of an infectious agent by the study treatment (Table 8-1).

AESIs will be reported to the Sponsor or designee using the SAE reporting form irrespective of whether the event is serious or nonserious; all AESIs must be reported within 24 hours using the SAE process as described in Section 8.2.

Guidance for management of immune-mediated adverse events associated with atezolizumab is provided in the protocol (Section 6.5.2.2) and can also be found in the local prescribing information and atezolizumab Investigator's Brochure.

Table 8-1: Adverse Events of Special Interest for Atezolizumab

- Cases of potential DILI that include an elevated ALT or AST in combination with either an elevated bilirubin or clinical jaundice as based on the following observations:
  - **<u>For non-HCC subjects (Hy's Law)</u>**:
    - Treatment-emergent ALT or AST > 3 × ULN in combination with total bilirubin > 2 × ULN
    - Treatment-emergent ALT or AST > 3 × ULN in combination with clinical jaundice
  - **<u>For HCC subjects</u>**:
    - Treatment-emergent ALT or AST > 3 × baseline value in combination with total bilirubin > 2 × ULN (of which ≥ 35% is direct bilirubin)
    - Treatment-emergent ALT or AST > 3 × baseline value in combination with clinical jaundice
- Suspected transmission of an infectious agent by the study treatment, as defined below
  - Any organism, virus, or infectious particle (eg, prion protein transmitting transmissible spongiform encephalopathy), pathogenic or non-pathogenic, is considered an infectious agent. A transmission of an infectious agent may be suspected from clinical symptoms or laboratory findings that indicate an infection in a patient exposed to a medicinal product. This term applies only when a contamination of study treatment is suspected.
- Pneumonitis
- Colitis
- Endocrinopathies: diabetes mellitus, pancreatitis, adrenal insufficiency, hyperthyroidism, and hypophysitis
- Hepatitis, including AST or ALT > 10 × ULN
- Systemic lupus erythematosus
- Neurological disorders: Guillain-Barré syndrome, myasthenic syndrome or myasthenia gravis, and meningoencephalitis
- Events suggestive of hypersensitivity, infusion-related reactions, cytokine release syndrome, influenza-like illness, hemophagocytic lymphohistiocytosis, and macrophage activation syndrome
- Nephritis
- Ocular toxicities (eg, uveitis, retinitis)
- Myositis
- Myopathies, including rhabdomyolysis
- ≥ Grade 2 cardiac disorders (eg, atrial fibrillation, myocarditis, pericarditis)
- Vasculitis
- Severe cutaneous reactions (eg, Stevens-Johnson syndrome, bullous dermatitis, toxic epidermal necrolysis)

ALT, alanine aminotransferase; AST, aspartate aminotransferase; DILI, drug-induced liver injury; ULN, upper limit of normal.

8.3.1 General Information on Immune-Related Adverse Events

The immune-modulating properties of checkpoint-inhibitors, such as the anti-PD-L1 antibody atezolizumab, are able to unbalance the immunologic tolerance and generate a subset of AEs (called irAEs) with an autoimmune inflammatory pathomechanism. IrAEs may involve every organ or tissue (Michot et al 2016). Most irAEs occur within the first 12 weeks of exposure to ICIs but some of them may appear with a delayed onset. Diagnosis of irAEs should be based on exposure to an ICI and a reasonable immune-based mechanism of the observed AE. Whenever possible, histologic examination or other immune-based diagnostic evaluations should be used to support the diagnosis. Other etiologic causes including AEs from tumor progression should be ruled out.

The spectrum of irAEs is wide and can be general or organ-specific. Examples of general irAEs in subjects treated with ICIs are fatigue, fever, and chills. Organ-specific irAEs consist of dermatitis (rash, pruritus, vitiligo, oral mucositis, and gingivitis), enterocolitis (diarrhea with abdominal pain and clinical or radiological evidence of colonic inflammation), and endocrinopathies (pituitary, thyroid, adrenal, testes). Diagnosis of endocrine dysfunction is challenging with relatively unspecific symptoms. Additional laboratory testing of the endocrine axes may be helpful: prolactin (pituitary-hypothalamic function), T4 and TSH (pituitary-thyroid function), luteinizing hormone (LH) and follicle-stimulating hormone (FSH) (pituitary-gonadal function), adrenocorticotropic hormone (ACTH) and cortisol (pituitary-adrenal function).

Additional organ-specific irAEs include hepatitis (AST/ALT increases, hepatomegaly, periportal edema, periportal lymphadenopathy, lymphocyte infiltrates periportal and surrounding primary biliary ducts) and pneumonitis (acute interstitial pneumonia). Less frequent irAEs include neurologic syndromes (myasthenia gravis, Guillain-Barré syndrome, aseptic meningitis), ocular AEs (uveitis), renal AEs (interstitial nephritis), cardiac AEs (myocarditis), muscular AEs (myositis), skin-related AEs (Stevens-Johnson syndrome, toxic epidermal necrolysis), and pancreatic AEs (lipase increase).

Medical management of irAEs focuses on suppressing the immune response with non-steroidal and steroidal anti-inflammatory medication. Treatment algorithms for high grade irAEs have been developed and should be followed for subjects with suspected irAEs because of ICI exposure (Naidoo et al 2015).

8.4 Follow-Up of Adverse Events

If a subject is experiencing an ongoing treatment-related AE that led to study treatment discontinuation, SAE, or AESI at the time of the Post-Treatment Follow-Up Visit 30 (+14) days after the date of the decision to discontinue treatment (see Section 5.3 for further details), the subject will continue to be followed until either:

- the AE has resolved
- the AE has improved to Grade 2 or lower
- The investigator determines that the event has become stable or irreversible.

This follow-up requirement also applies to related SAEs that occur > 30 days after the date of the decision to discontinue study treatment.

In addition, AESIs are to be recorded in the CRF until 90 days after the decision to discontinue study treatment.

The status of all other AEs that are ongoing 30 days after the date of the decision to discontinue study treatment will be documented as of the Post-Treatment Follow-Up Visit.

8.5 Other Safety Considerations

8.5.1 Pregnancy

Use of highly effective methods of contraception as defined in Appendix K is very important during the study and for 5 months after the last dose of study treatment. If a subject becomes pregnant during the study, she will be taken off study treatment. She will be followed through the end of her pregnancy and the infant should have follow up for at least 6 months after birth. Furthermore, male subjects must refrain from donating sperm in order to avoid transmission of study treatment in semen for the duration of study treatment and through 5 months after their last dose of study treatment. If a female partner of a male subject becomes pregnant during the study, the Sponsor will ask the pregnant female partner to be followed through the end of her pregnancy and for the infant to be followed for at least 6 months after birth.

The investigator must inform the Sponsor of the pregnancy. Forms for reporting pregnancies will be provided to the study sites upon request. The outcome of a pregnancy (for a subject or for the partner of a subject) and the medical condition of any resultant offspring must be reported to the Sponsor or designee. Any birth defect or congenital anomaly must be reported as an SAE and

any other untoward events occurring during the pregnancy must be reported as AEs or SAEs, as appropriate.

Females should not breastfeed while receiving study treatment and for the following periods after discontinuing study treatment:

- **Cabozantinib + atezolizumab**: at least 5 months from the last dose of atezolizumab or 4 months from the last dose of cabozantinib, whichever is later
- **Single-agent cabozantinib:** 4 months from the last dose of cabozantinib
- **Single-agent atezolizumab:** at least 5 months from the last dose of atezolizumab

8.5.2 Medication Errors/Overdose

Medication error is defined as the administration of study drug medication outside or above the established dosing regimens per the specific protocol.

Any study medication overdose, misuse, abuse, or study medication error (excluding missed doses) that results in an AE or SAE requires reporting to the Sponsor or designee according to the guidance for AE and SAE reporting (Sections 8.1 and 8.2, respectively).

In case of overdose, the Sponsor medical monitor or designee should be contacted promptly to discuss how to proceed. A Medication Error Notification Form should be completed and sent to the Sponsor following any suspected medication error. Any AEs that occur as a result of an overdose have to be treated according to clinical standard practice.

Please refer to the Investigator's Brochure for additional management recommendations for an overdose of cabozantinib.

9 STATISTICAL CONSIDERATIONS

Details of the planned analyses, including strategies if needed to assess and address consequences of the COVID-19 pandemic on trial conduct and study data, will be documented in a separate Statistical Analysis Plan (SAP). Summaries will generally be presented by cohort/dose group and overall (total subjects). No formal statistical tests are planned for this study. Confidence intervals will be calculated for selected endpoints.

9.1 Power and Sample Size

[redacted]

9.1.2 Expansion Stage

9.1.2.1 Combination-Therapy Expansion Cohorts

The objective for the Combination-Therapy Expansion Cohorts is to estimate ORR to assess if the true response rate with this combination regimen is better than that expected with monotherapy. Thus, 2-sided 80% and 60% Blyth-Still-Casella CIs will be constructed for ORR, providing 90% and 80%, respectively, 1-sided confidence when interpreting the lower bound. The sample size of 30 subjects for each of the Expansion Cohorts was chosen to ensure the lower bound of the 2-sided 80% CI extended no more than 12 percentage points from the point estimate. Example 80% and 60% 2-sided CIs, with the 1-sided interpretations of the lower bound, are shown in Table 9-1 for a range of potential values for observed ORR.

Table 9-1: Example Blyth-Still-Casella Confidence Intervals for N=30 for ORR for Expansion Cohorts with 1-Sided Interpretations of the Lower Bound

Observed Responses (Total N=30)	Observed ORR (%)	80% 2-Sided CI			60% 2-Sided CI		
		LCL (%)	UCL (%)	True ORR[a] (90% Confidence) (%)	LCL (%)	UCL (%)	True ORR[a] (80% Confidence) (%)
17	57	44	69	≥ 44	47	66	≥ 47
15	50	38	62	≥ 38	41	59	≥ 41
12	40	28	53	≥ 28	31	47	≥ 31
11	37	25	50	≥ 25	28	44	≥ 28
10	33	23	46	≥ 23	25	41	≥ 25
9	30	19	42	≥ 19	24	38	≥ 24
8	27	16	38	≥ 16	19	34	≥ 19
7	23	15	34	≥ 15	16	31	≥ 16
6	20	11	31	≥ 11	13	28	≥ 13
5	17	9	28	≥ 9	12	24	≥ 12
4	13	6	25	≥ 6	8	19	≥ 8

CI, confidence interval; LCL, lower confidence limit; ORR objective response rate; UCL, upper confidence limit.

[a] Per 1-sided interpretation of the lower bound.

Table 9-2: Example Blyth-Still-Casella Confidence Intervals for N=15 for ORR for the Expansion Cohorts of 15 Subjects with 1-Sided Interpretations of the Lower Bound

Observed Responses (Total N=15)	Observed ORR (%)	80% 2-Sided CI			60% 2-Sided CI		
		LCL (%)	UCL (%)	True ORR[a] (90% Confidence) (%)	LCL (%)	UCL (%)	True ORR[a] (80% Confidence) (%)
9	60	42	77	≥ 43	46	70	≥ 46
7	47	28	64	≥ 28	33	61	≥ 33
6	40	23	57	≥ 23	30	54	≥ 30
5	33	20	51	≥ 20	23	46	≥ 23
4	27	12	44	≥ 12	16	39	≥ 16
3	20	10	36	≥ 10	11	33	≥ 11
2	13	6	28	≥ 6	8	23	≥ 8
1	7	1	23	≥ 1	3	16	≥ 3

CI, confidence interval; LCL, lower confidence limit; ORR objective response rate; UCL, upper confidence limit.
[a] Per 1-sided interpretation of the lower bound.

Combination-Therapy Expansion Cohorts may enroll additional subjects beyond the initial subjects per Extended Enrollment Option 1 or 2 (described below). It is anticipated that not all Expansion Cohorts will open for additional enrollment. Note: Up to 10 tumor-specific cohorts [redacted] may be expanded in the Expansion Stage[redacted]

Extended Enrollment Option 1: Should the SOC deem that a clinically meaningful ORR has been observed in an Expansion Cohort, approximately 100 new subjects may be added to that cohort to further investigate the safety and clinical benefit of the combination in that treatment setting.

Decisions by the SOC regarding the clinical significance of the achieved ORR in Expansion Cohorts will include an evaluation of the lower bound of confidence intervals for ORR in the initially enrolled approximately 30 subjects, and the expansion cohorts will be extended as follows:

Extension-Part I: Approximately 50 additional subjects will be added in this extension part for a total of approximately 80 subjects in an expansion cohort. The observed ORR in the previously enrolled subjects will be considered. A minimum observed ORR of around 20% or more will be used as a target (though not a requirement) for the SOC to consider cohort expansion. This corresponds to 80% confidence that the true ORR is ≥ 13% for n = 30 (see Table 9-1) (≥ 11% for n = 15; see Table 9-2). The magnitude of ORR deemed clinically meaningful by the SOC may vary by cohort, and the committee may consider other factors of clinical benefit (eg, time to response, duration of response, safety/tolerability) in the decision to extend enrollment. [redacted]

Table 9-3: Example Blyth-Still-Casella Confidence Intervals for N = 60 for ORR for Expansion Cohorts with 1-Sided Interpretations of the Lower Bound

Observed Responses (Total N=60)	Observed ORR (%)	80% 2-Sided CI		
		LCL (%)	UCL (%)	True ORR[a] (90% Confidence) (%)
15	25	18	33	≥ 18
16	27	20	35	≥ 20
17	28	21	36	≥ 21
18	30	22	39	≥ 22
19	32	24	39	≥ 24
20	33	25	41	≥ 25
21	35	27	43	≥ 27

CI, confidence interval; LCL, lower confidence limit; ORR objective response rate; UCL, upper confidence limit.
[a] Per 1-sided interpretation of the lower bound.

Table 9-4: Example Blyth-Still-Casella Confidence Intervals for N=80 for ORR for Expansion Cohorts with 1-Sided Interpretations of the Lower Bound

Observed Responses (Total N=80)	Observed ORR (%)	80% 2-Sided CI		
		LCL (%)	UCL (%)	True ORR[a] (90% Confidence) (%)
32	40	33	47	≥ 33
31	39	31	46	≥ 31
30	38	30	45	≥ 30
29	36	29	44	≥ 29
28	35	28	42	≥ 28
27	34	27	41	≥ 27
26	33	26	40	≥ 26
25	31	25	39	≥ 25
24	30	23	37	≥ 23

CI, confidence interval; LCL, lower confidence limit; ORR objective response rate; UCL, upper confidence limit.

[a] Per 1-sided interpretation of the lower bound.

Extension-Part II: Approximately 50 additional subjects will be added in this second extension part for a maximum total of approximately 130 subjects in an expansion cohort. The observed ORR for the previously enrolled subjects (N= ~80, ~30 subjects initially enrolled + ~50 subjects enrolled in Extension-Part I) will be considered. A minimum observed ORR of around 35% or more will be used as a target for the SOC to consider additional cohort expansion. This corresponds to 90% confidence that the true ORR is ≥ 28% for n = 80 (see Table 9-4). Part II extension will only apply to tumor indications with a high-unmet medical need and very encouraging efficacy and safety data observed in Part I.

A total sample size of 130 subjects was selected to ensure the lower bound of the 95% confidence interval for ORR will extend less than 10% points from the point estimate if Part II is implemented. See Table 9-5 for confidence intervals for a range of potentially observed response rates.

Table 9-5: Example Blyth-Still-Casella Confidence Intervals for N=130 for ORR for Expansion Cohorts with 1-Sided Interpretations of the Lower Bound

Observed Responses (Total N=130)	Observed ORR (%)	95% 2-Sided CI		
		LCL (%)	UCL (%)	True ORR[a] (97.5% Confidence) (%)
65	50	41	59	≥ 41
58	45	36	54	≥ 36
52	40	32	49	≥ 32
45	35	27	43	≥ 27

CI, confidence interval; LCL, lower confidence limit; ORR objective response rate; UCL, upper confidence limit.
[a] Per 1-sided interpretation of the lower bound.

Extended Enrollment Option 2: For Combination-Therapy Expansion Cohorts in which the initially enrolled approximately 30 subjects do not meet the criteria for Extended Enrollment Option 1, the SOC may decide to allow each selected Expansion Cohort to enroll approximately 30 new subjects to receive the highest dose level of cabozantinib explored in the Dose-Escalation Stage (60 mg) in combination with atezolizumab 1200 mg to explore whether the higher cabozantinib dose will lead to improved clinical activity and maintain an acceptable safety profile.

Details about the composition, role, schedule, and guidance for committee decisions are provided in a separate SOC Charter.

[redacted]

[redacted]

[redacted]

9.2 Analysis Populations

9.2.1 Safety Population

The Safety population will consist of all subjects who received any study treatment. As enrollment is defined by receipt of study treatment, an Enrolled population is not defined to be distinct from the Safety population.

[redacted]

9.2.3 Other Population(s)

Additional analysis populations may be defined in the SAP.

9.3 Planned Analyses

9.3.1 Safety and Tolerability Analyses

Safety will primarily be assessed by the evaluation of AEs and laboratory tests. Tolerability will be assessed by evaluation of study treatment modification and discontinuation.

9.3.1.1 Adverse Events

Adverse event terms recorded on the CRFs will be mapped to preferred terms using the Medical Dictionary for Regulatory Activities (MedDRA). The investigator will classify the severity of AEs using the CTCAE v4 and will judge each event to be "not related" or "related" to study treatment. Adverse events leading to study treatment discontinuation will also be judged by the investigator to be causally associated, or not, with the disease under study.

Summaries of AEs, irAEs, AESIs, and SAEs will be tabulated by cohort according to system organ class and preferred term by overall incidence; worst reported severity; and relationship to study treatment.

At each level of summarization, a subject will be counted only once for each AE preferred term he or she experiences within that level (ie, multiple episodes of events with the same preferred terms will be counted only once).

All reported subject deaths will be summarized by treatment group, cause of death, and relationship to study treatment.

A narrative will also be prepared to describe the accrual and expansion of Dose-Escalation Stage cohorts, subject replacement, the DLTs observed, Cohort Review Committee decisions and the final rationale for the recommended Expansion Stage dose.

9.3.1.2 Laboratory Test Results

Selected laboratory test results will be summarized by treatment group to evaluate worst post-baseline CTCAE grade and shifts or changes from baseline.

9.3.1.3 Study Treatment

Study treatment parameters will be presented separately for each agent, cabozantinib and atezolizumab. The number of subjects experiencing dose reduction, delay, interruption, modification and/or discontinuation due to adverse event will be provided as appropriate for each agent. Duration and intensity of study treatment will also be tabulated. [redacted]

9.3.2 Analyses of Preliminary Antitumor Activity

The objective of the combination therapy in the Expansion Stage is to estimate ORR, defined as the proportion of subjects with a confirmed CR or PR per RECIST 1.1 as determined by the investigator. Similarly, ORR will be determined per irRECIST for immune response (Appendix H) as determined by the investigator as an exploratory endpoint. ORR will be evaluated independently within each of the Expansion Cohorts and within the dose-escalation cohorts. For selected Expansion Stage cohorts, ORR per RECIST 1.1 will also be evaluated per BIRC.

Best overall tumor response, based upon the evaluation of target, non-target, and new lesions, will be presented as the proportion of subjects in each of the following categories: CR, PR, SD, PD, and not evaluable, and include the ORR. In the Expansion Cohorts, 2-sided 80% and 60% Blyth-Still-Casella CIs will be presented for ORR, providing 90% and 80% 1-sided confidence when interpreting the lower bound for the purpose of evaluating preliminary efficacy of combination therapy vs. expected efficacy with single-agent treatment from historical studies. Confidence intervals at the 95% level will also be presented for consistency with standard presentation conventions.

For exploratory purposes, the ORR as assessed by investigator per RECIST 1.1 will be presented descriptively for each dose-escalation cohort.

Median PFS and OS with associated 2-sided 95% CIs will be estimated using Kaplan-Meier methods.

Duration of response is defined as the time from first documented objective response (CR or PR) as is assessed by the investigator that is subsequently confirmed until the earlier of radiographic

progression or death, or censoring due to lack of these events or start of nonprotocol anticancer therapy. Medians and confidence intervals will be estimated using Kaplan-Meier analysis, limited to patients who experienced a confirmed objective response.

9.3.3 Interim Analyses

Prior to Protocol Amendment 2.0, the Cohort Review Committee reviewed accumulating data from the Dose-Escalation cohorts as described in Section 12.1.

No formal interim analyses are planned for the cohorts in the Expansion Stage. However, safety and anti-tumor findings will be reviewed on an ongoing basis.

10 OTHER ANALYSES

10.1 Pharmacokinetic Analyses

The plasma concentration of cabozantinib will be analyzed by the Sponsor or designee using a validated bioanalytical method. Descriptive statistics (eg, number, mean and/or median, standard deviation, and coefficient of variation) will be used to describe the concentration-time data. Where appropriate, these data may be analyzed using population PK models and/or combined with data from other studies as part of a meta-analysis. The influence of exposure on biomarkers, clinical safety parameters (eg, selected AEs) or clinical response may also be explored.

Serum concentrations of atezolizumab will be analyzed by Sponsor designated lab using validated enzyme-linked immunosorbent assay (ELISA). Descriptive statistics (eg, number, mean and/or median, standard deviation, and coefficient of variation) will be used to summarize the concentration-time data per visit.

10.2 Immunogenicity Analyses

Results of anti-drug antibody (ADA) testing (ie, immunogenicity testing) will be summarized overall as the number of subjects with ADA at any time point. The association between human ADA incidence, PK, and efficacy and/or safety outcomes may be explored.

10.3 Biomarker Analyses

Analyses that may include MET and PD-L1 expression levels and potential correlation with clinical response and other analyses (eg, tumor mutational burden) will be summarized. Exploratory evaluation of relevant biomarkers for on-target effects of therapy and tumor and/or peripheral changes in immune response may be summarized separately.

11 DATA QUALITY ASSURANCE

Accurate and reliable data collection will be ensured by verification and cross-check of the CRFs against the investigator's records by the study monitor (source document verification) and by the maintenance of a drug–dispensing log by the investigator. Data collected on paper CRFs, if any, will be entered into a computer database. If electronic CRFs are employed, authorized study site personnel will enter data directly into a computer database. Study databases will be subject to electronic and manual quality assurance procedures.

12 STUDY COMMITTEES

12.1 Cohort Review Committee

The Cohort Review Committee included the Sponsor medical monitor and/ or the Sponsor Drug Safety physician, the Sponsor's chief medical officer (or Vice President of Clinical Development), and participating principal investigators. The Cohort Review Committee reviewed all safety and available PK data from all subjects from each cohort. All available safety and PK data was considered in decisions to dose escalate or de-escalate the next cohort or to expand the current cohort in the Dose-Escalation Stage. Once all subjects in the Dose-Escalation Stage completed the DLT Evaluation Period, the Cohort Review Committee determined the MTD/recommended dose and dosing schedule for the Expansion Stage based on review and discussion of the safety and available PK data from all subjects in the Dose-Escalation Stage. Details about this process are provided in Section 3.5.1.

12.2 Study Oversight Committee

The SOC consists of Sponsor medical, safety, and biostatistical personnel and selected investigators which are experts in the treatment of the enrolled tumor types. The SOC will periodically monitor safety and efficacy data of cohorts in the Expansion Stage and decide upon further enrollment extension of up to 10 cohorts [redacted] as described in Section 9.1.2.

Details about the composition, role, schedule, and guidance for committee decisions are provided in a separate SOC Charter.

12.3 Corporate Safety Governance

The Sponsor has an established internal safety governance structure which oversees the monitoring of the safety and benefit-risk profile for all investigational products on an ongoing basis across all ongoing company-sponsored clinical studies.

12.4 Blinded Independent Radiology Committee (BIRC)

A BIRC will be established to evaluate tumor scans and prior radiation/prior local tumor history data of trial subjects for selected Expansion Stage cohorts in a central, blinded, and independent fashion. The BIRC will comprise board-certified radiologists who will determine radiographic response and progression following randomization. Additional imaging results may be requested by the Sponsor for BIRC review.

Additional details regarding BIRC member qualification, training, methods, procedures, and other issues relevant to committee operations will be described in the BIRC Charter.

13 ETHICAL ASPECTS

13.1 Local Regulations

The study must fully adhere to the principles outlined in "Guideline for Good Clinical Practice" (GCP) ICH E6 Tripartite Guideline (January 1997) and remain consistent with the most recent version of the Declaration of Helsinki. The investigator will ensure that the conduct of the study complies with the basic principles of GCP as outlined in the current version of 21 CFR, subpart D, Part 312, "Responsibilities of Sponsors and Investigators" Part 50, "Protection of Human Subjects" and Part 56, "Institutional Review Boards."

13.2 Informed Consent

Sample informed consent forms (ICFs) will be supplied to each site. The Sponsor or its designee must review any proposed deviations from the sample ICF. The final IRB/EC-approved document must be provided to the Sponsor for regulatory purposes.

It is the responsibility of the investigator, or a person designated by the investigator, to obtain written informed consent from each subject participating in this study after adequate explanation of the aims, methods, anticipated benefits, and potential hazards of the study. In the case where the subject is unable to read, an impartial witness must be present during the entire informed consent discussion. After the subject has orally consented to participation in the trial, the witness' signature on the form will attest that the information in the consent form was accurately explained and understood. A copy of the ICF must be provided to the subject. If applicable, the ICF will be provided in a certified translation of the subject's language.

The CRF for this study contains a section for documenting informed subject consent, and this must be completed appropriately. Signed ICFs must remain in each subject's study file and must be available for verification by study monitors at any time. If new safety information results in significant changes in the risk/benefit assessment, the consent form will be reviewed and updated

as necessary. All subjects (including those already being treated) will be informed of the new information, will be given a copy of the revised form, and must give their consent to continue in the study.

13.3 Institutional Review Board/ Ethics Committee

This study is being conducted under a United States Investigational New Drug application or other Clinical Trial Application, as appropriate. This protocol (and any modifications) and appropriate consent procedures must be reviewed and approved by an IRB/EC. This board must operate in accordance with current local, regional, and federal regulations. The investigator will send a letter or certificate of IRB/ EC approval to the Sponsor (or designee) before subject enrollment and whenever subsequent modifications to the protocol are made.

13.4 Disposition of Subject Samples

Protocol-defined analyses are anticipated to result in depletion of all or almost all research samples. If a subject requests destruction of their tissue and blood samples, the Sponsor will make every attempt to destroy the samples. The Sponsor will notify the investigator in writing that samples have been destroyed.

14 CONDITIONS FOR MODIFYING THE PROTOCOL

Protocol modifications may be made and will be prepared, reviewed, and approved by the Sponsor representatives.

All protocol modifications must be submitted to the IRB/EC for information and approval in accordance with local requirements, and to regulatory agencies if required. Approval must be obtained before any changes can be implemented, except for changes necessary to eliminate an immediate hazard to study subjects or those that involve only logistical or administrative aspects of the trial (eg, change in monitor or change of telephone number).

15 CONDITIONS FOR TERMINATING THE STUDY

The Sponsor reserves the right to terminate the study, and investigators reserve the right to terminate their participation in the study, at any time. Should this be necessary, the Sponsor and the investigator will arrange the procedures on an individual study basis after review and consultation. In terminating the study, the Sponsor and the investigator will ensure that adequate consideration is given to the protection of the subjects' interests.

Study Completion by Country or by Site: After sufficient data have been collected to adequately evaluate all study endpoints and upon site notification by the Sponsor, the study will be considered complete at sites and in countries that no longer have active subjects.

16 STUDY DOCUMENTATION, CASE REPORT FORMS, AND RECORD KEEPING

16.1 Investigator's Files and Retention of Documents

The investigator must maintain adequate and accurate records to enable the conduct of the study to be fully documented and the study data to be subsequently verified. These documents should be classified into two separate categories as follows: (1) the investigator's study file and (2) subjects' clinical source documents.

The investigator's study file will contain the protocol and protocol amendments, CRFs (site contents will be converted to digital storage format [eg, compact disc] for archiving), query forms, IRB/EC and governmental approvals with correspondence, sample informed consent, drug records, staff curriculum vitae and authorization forms, and other appropriate documents and correspondence.

Subjects' clinical source documents (usually predefined by the project to record key efficacy and safety parameters independent of the CRFs) include the subjects' hospital/ clinic records; physician's and nurse's notes; the appointment book; original laboratory, ECG, electroencephalogram, X-ray, pathology and special assessment reports; signed ICFs; consultant letters; and subject screening and enrollment logs.

The Investigator must retain all study records and source documents for the maximum period required by applicable regulations and guidelines, or institution procedures, or for the period specified by the Sponsor or designee, whichever is longer. The investigator must contact the Sponsor prior to destroying any records associated with the study. The Sponsor or designee will notify the investigator when the study records are no longer needed. If the investigator withdraws from the study (eg, relocation, retirement), the records shall be transferred to a mutually agreed upon designee (eg, another investigator, study site, IRB). Notice of such transfer will be given in writing to the Sponsor or designee.

If the investigator cannot guarantee the archiving requirement at the study site for any or all of the documents, special arrangements must be made between the investigator and the Sponsor to store these in a sealed container outside of the study site so that they can be returned sealed to the investigator in case of a regulatory audit. When source documents are required for the continued care of the subject, appropriate copies should be made for storing outside of the study site.

16.2 Source Documents and Background Data

Upon request, the investigator will supply the sponsor with any required background data from the study documentation or clinic records. This is particularly important when CRFs (if paper) are illegible or when errors in data transcription are suspected. In case of special problems or governmental queries or requests for audit inspections, it is also necessary to have access to the complete study records, provided that subject confidentiality is protected.

16.3 Audits and Inspections

The investigator should understand that source documents for this study must be made available, after appropriate notification, to qualified personnel from the Sponsor's Quality Assurance Unit (or designee) or to health authority inspectors. The verification of the CRF data must be by direct inspection of source documents.

16.4 Case Report Forms

The term "case report form" includes as applicable paper forms and/or electronic data capture screens or forms for studies that utilize electronic data capture. For enrolled subjects, all and only data for the procedures and assessments specified in this protocol and required by the CRFs are to be submitted on the appropriate CRF (unless source data are transmitted to the Sponsor or a designee electronically, eg, central laboratory data). Data from some procedures required by the protocol, such as physical examinations, will be recorded only on the source documents and will not be transcribed to CRFs. Additional procedures and assessments may be performed as part of the investigator's institution or medical practice standard of care. Data from assessments associated with the follow-up of AEs are to be recorded on unscheduled CRF pages. Otherwise, data for unscheduled or additional assessments are to remain in the subject's medical record and are not to be recorded on CRFs unless specifically requested.

The CRF (paper or electronic) casebook must be completed and signed by the investigator or authorized delegate from the study staff. This also applies to records for those subjects who fail to complete the study. If a subject stops dosing or terminates from the study, the dates and reasons must be noted on the CRF.

All paper forms are to be typed or filled out using indelible ink and must be legible. Errors are to be crossed out but not obliterated, the correction inserted, and the change initialed and dated by the investigator or his or her authorized delegate. The investigator should ensure the accuracy, completeness, legibility, and timeliness of the data reported to the Sponsor in the CRF and in all required reports.

The Sponsor's data management personnel (or designees) may, in specific circumstances, modify study data – without changing the meaning of the data – to ensure the dataset complies with conventions required for successful data extract, thesaurus coding, or uniform reporting and does not cause these processes to fail. Examples of these administrative changes include:

- Substitution of non-standard ASCII characters (codes 128-255) or deletion of carriage returns (code 13) that are incompatible with the SAS XPT file format (eg, accented letters replaced with non-accented ones; e for é)
- Splitting multiple verbatim AE terms into multiple records (eg, "nausea and vomiting" to separate records for "nausea" and "vomiting")
- Reformatting failed eligibility criteria numbers for uniformity or specificity (eg, changing "2 a" to "2A"; or "2" to "2A" based on corroborating evidence from the clinical database)
- Changing cause of death from "unknown" to "unknown cause of death" to facilitate coding in the MedDRA thesaurus

Such changes follow a pre-defined documented process and can be clearly identified in the database audit trial. By participating in this study, investigators agree that such administrative changes are permissible without their specific prior approval. A list of all specific changes made can be provided to investigators upon request at any time.

17 MONITORING THE STUDY

The responsible Sponsor monitor (or designee) will contact and visit the investigator regularly and will be allowed on request to inspect the various records of the trial (CRFs and other pertinent data), provided that subject confidentiality is maintained in accordance with local requirements.

It will be the monitor's responsibility to inspect the CRFs at regular intervals throughout the study to verify both adherence to the protocol and the completeness, consistency, and accuracy of the data being entered on them. The monitor is to have access to laboratory test reports and other subject records needed to verify the entries on the CRF. The investigator (or designee) must agree to cooperate with the monitor to ensure that any problems detected in the course of these monitoring visits are resolved.

18 CONFIDENTIALITY OF TRIAL DOCUMENTS AND SUBJECT RECORDS

The investigator must assure that subjects' anonymity will be maintained and that their identities are protected from unauthorized parties. On CRFs or other documents submitted to the Sponsor or designees, subjects are to be identified by identification codes and not by their names. The investigator should keep a subject enrollment log showing codes, names, and addresses. The investigator must maintain documents not for submission to the Sponsor or designees (eg, subjects' written consent forms) in strict confidence.

All tumor scans, research samples, photographs, and results from examinations, tests, and procedures may be sent to the Sponsor and its partners or designees for review.

19 PUBLICATION OF DATA AND PROTECTION OF TRADE SECRETS

The results of this study may be published or presented at scientific meetings. The investigator agrees to submit all manuscripts or abstracts to the Sponsor for review at least 30 days before submission. This allows the Sponsor to protect proprietary information and to provide comments based on information from other studies that may not yet be available to the investigator.

In the event that the Sponsor coordinates a publication or presentation of study results from all study sites, the participation of the investigator(s) or other representatives of the study site(s) as named author(s) shall be determined in accordance with Sponsor policy. Authorship will be assigned in accordance with contribution to design, execution, and interpretation and analysis of the study.

The Sponsor may, at its sole option, provide funding to support the development, submission, and/or presentation of publications for scientific/medical journals or conferences. For publications coordinated by the Sponsor, the Sponsor may also provide funding to support travel and conference registration for the presenting author to attend the conference for the sole purpose of presenting the publication.

20 COMPLIANCE WITH DATA PROTECTION LAWS

The conduct of this study and the processing of any personal data collected from each subject (or from a subject's healthcare professional or other relevant third-party sources) by the Sponsor, the site, and the Investigator for use in the study will fully adhere to the requirements set out in applicable data protection and medical privacy laws or regulations, including, without limitation, the General Data Protection Regulation ([EU] 2016/679) and any national implementing laws, regulations, and secondary legislation, as amended or updated from time to time. The Sponsor shall ensure that at all times it has an appropriate legal basis for processing personal data under applicable data protection law (which may include consent from the subject or another lawful basis).

21 REFERENCES

Abou-Alfa GK, Meyer T, Cheng A-L, El-Khoueiry AB, Rimassa L, Ryoo B-Y, et al. Cabozantinib (C) versus placebo (P) in patients (pts) with advanced hepatocellular carcinoma (HCC) who have received prior sorafenib: Results from the randomized phase III CELESTIAL trial. J Clin Oncol. 2018;36 (suppl 4S; abstr 208).

Adashek ML, Feldman M. Cytokine release syndrome resulting from anti-programmed death-1 antibody: raising awareness among community oncologist. J Oncol Practice 2019;15:502-4.

Ahn S, Kim TH, Kim SW, Ki CS, Jang HW, Kim JS, et al. Comprehensive screening for PD-L1 expression in thyroid cancer. Endocr Relat Cancer. 2017;24(2):97-106.

Apolo AB, Mortazavi A, Stein M, Pal SK, Davarpanah N, Parnes HL, et al. A phase I study of cabozantinib plus nivolumab (CaboNivo) in patients (pts) refractory metastatic urothelial carcinoma (mUC) and other genitourinary tumors. Ann Oncol 27, 2016 (suppl 6).

Apolo AB, Parnes HL, Francis DC, Cordes LM, Berninger M, Lamping E, et al. A phase II study of cabozantinib in patients (pts) with relapsed or refractory metastatic urothelial carcinoma (mUC) [poster]. J Clin Oncol. 34, 2016 (suppl; abstr 4534).

Apolo AB, Tomita Y, Lee M-J, Lee S, Frosch A, Steinberg SM, et al. Effect of cabozantinib on immunosuppressive subsets in metastatic urothelial carcinoma. J Clin Oncol. 32:5s, 2014 (suppl; abstr 4501).

Atkins MB, McDermott DF, Powles T, Motzer RJ, Rini BI, Fong L, et al. IMmotion150: A phase II trial in untreated metastatic renal cell carcinoma (mRCC) patients (pts) of atezolizumab (atezo) and bevacizumab (bev) vs and following atezo or sunitinib (sun).J Clin Oncol. 2017; 35(15):suppl 4505-4505.

Bahleda R, Braiteh FS, Balmanoukian AS, Braña I, Hodi FS, Garbo L, et al. Long-Term Safety and Clinical Outcomes of Atezolizumab in Head and Neck Cancer: Phase Ia Trial Results. Ann Oncol. 2017;28:Issue suppl_5 1044O.

Balar A, Galsky M, Rosenberg J, Powles T, Petrylak D, Bellmunt J,et al. Atezolizumab as first-line treatment in cisplatin-ineligible patients with locally advanced and metastatic

urothelial carcinoma: a single-arm, multicentre, phase 2 trial. Lancet 2017; 389:67-76.

Basch E, Autio KA, Smith MR, Bennett AV, Weitzman AL, Scheffold C, et al. Effects of cabozantinib on pain and narcotic use in patients with castration-resistant prostate cancer: Results From a Phase 2 Nonrandomized Expansion Cohort. Eur Urol. 2015;67(2):310-8.

Bellmunt J, Balar AV, Galsky MD, Loriot Y, Théodore C, Grande E, et al. Updated Analyses of First-Line Atezolizumab in Cisplatin-Ineligible Locally Advanced/Metastatic Urothelial Carcinoma. Ann Oncol 27, 2016 (suppl 6).

Bendell JC, Powderly JD, Lieu CH, Eckhardt SG, Hurwitz H, Hochster HS, et al. Safety and efficacy of MPDL3280A (anti-PDL1) in combination with bevacizumab (bev) and/or FOLFOX in patients (pts) with metastatic colorectal cancer (mCRC). J Clin Oncol. 2015;33:(3_suppl 704).

Bishop JL, Sio A, Angeles A, Roberts ME, Azad AA, Chi KN, et al. PD-L1 is highly expressed in Enzalutamide resistant prostate cancer. Oncotarget. 2015;6:234-42.

Brandao Moreira R, McKay RR, Xie W, Heng DYC, de Velasco G, Castellano DE, et al. Clinical activity of PD1/PDL1 inhibitors in metastatic non-clear cell renal cell carcinoma (nccRCC). J Clin Oncol 2017;35:6_suppl, 482-482.

Brose MS, Shenoy S, Bhat N, Harlacker AK, Yurtal RK, Posey ZA, et al. A Phase II Trial of Cabozantinib for the Treatment of Radioiodine (RAI)-refractory Differentiated Thyroid Carcinoma (DTC) in the First-line Setting. Abstract 8. Presented at Multidisciplinary Head and Neck Cancers Symposium; Feb. 15-17, 2018; Scottsdale, Ariz.

Brusa D, Simone M, Gontero P, Spadi R, Racca P, Micari J, et al. Circulating immunosuppressive cells of prostate cancer patients before and after radical prostatectomy: profile comparison. Int J Urol. 2013;20(10):971-8.

Cabanillas ME, de Souza JA, Geyer S, Wirth LJ, Menefee ME, Liu SV, et al. Cabozantinib As Salvage Therapy for Patients With Tyrosine Kinase Inhibitor–Refractory Differentiated Thyroid Cancer: Results of a Multicenter Phase II International Thyroid Oncology Group Trial. J Clin Oncol. 2017; 35(29):3315-3321.

Cabanillas ME, Brose MS, Holland J, Ferguson KC, Sherman SI. A Phase I Study of Cabozantinib (XL184) in Patients with Differentiated Thyroid Cancer. Thyroid. 2014;24(10):1508-1514.

Choueiri TK, Hessel C, Halabi S, Sanford B, Hahn O, Michaelson MD, et al. Progression-free survival by independent review and updated overall survival results from Alliance A031203 trial (CABOSUN): cabozantinib versus sunitinib as initial targeted therapy for patients with metastatic renal cell carcinoma. Ann Oncol. 2017;28(suppl_5):v605-v649.

Choueiri TK, Halabi S, Sanford BL, Hahn O, Michaelson MD, Walsh MK, et al. Cabozantinib versus Sunitinib as initial targeted therapy for patients with metastatic renal cell carcinoma (mRCC) of poor or intermediate risk groups: The Alliance A031203 (CABOSUN) trial. J Clin Oncol. 2017 Feb 20;35(6):591-597.

Choueiri TK, Escudier B, Powles T, Tannir NM, Mainwaring PN, Rini BI, et al. Cabozantinib versus everolimus in advanced renal cell carcinoma (METEOR): final results from a randomised, open-label, phase 3 trial. Lancet Oncol. 2016;17(7):917-27.

Choueiri TK, Escudier B, Powles T, Mainwaring PN, Rini BI, Donskov F, et al. Cabozantinib versus Everolimus in Advanced Renal-Cell Carcinoma. N Engl J Med. 2015;373(19):1814-23.

Choueiri TK, Fay AP, Gray KP, Callea M, Ho TH, Albiges L, et al. PD-L1 expression in nonclear-cell renal cell carcinoma. Ann Oncol. 2014;25(11):2178-84.

Ciamporcero E, Miles KM, Adelaiye R, Ramakrishnan S, Shen L, Ku S, Pizzimenti S, et al. Combination strategy targeting VEGF and HGF/c-met in human renal cell carcinoma models. Mol Cancer Ther. 2015;14(1):101-10.

Davarpanah NN, Yuno A, Trepel JB, Apolo AB. Immunotherapy: a new treatment paradigm in bladder cancer. Curr Opin Oncol. 2017,29(3):184–195

Dhani NC, Hirte HW, Burnier JV, Jain A, Butler MO, Lheureux S, et al. Phase II study of cabozantinib (cabo) in patients (pts) with recurrent/metastatic endometrial cancer (EC): A study of the Princess Margaret, Chicago, and California phase II consortia. J Clin Oncol. 2017;35:15_suppl, 5524.

Drilon A, Rekhtman N, Arcila M, Wang L, Ni A, Albano M, et al. Cabozantinib in patients with advanced RET-rearranged non-small-cell lung cancer: an open-label, single-centre, phase 2, single-arm trial. Lancet Oncol. 2016;17(12):1653-1660.

Eisenhauer EA, Therasse P, Bogaerts J, et al. New response evaluation criteria in solid tumors: revised RECIST guideline (version 1.1). Eur J Cancer 2009; 45(2): 228–247.

Elisei R, Schlumberger MJ, Müller SP, Schöffski P, Brose MS, Shah MH, et al. Cabozantinib in progressive medullary thyroid cancer. J Clin Oncol. 2013;31:3639-46.

El-Khoueiry AB, Sangro B, Yau T, Crocenzi TS, Kudo M, Hsu C, et al. Nivolumab in patients with advanced hepatocellular carcinoma (CheckMate 040): an open-label, non-comparative, phase 1/2 dose escalation and expansion trial. Lancet. 2017;389(10088):2492-2502.

Engelman JA, Zejnullahu K, Mitsudomi T, Song Y, Hyland C, Park JO, et al. MET amplification leads to gefitinib resistance in lung cancer by activating ERBB3 signaling. Science. 2007;316(5827):1039-43.

Escudier B, Tannir NM, McDermott DF, Frontera OA, Melichar B, Plimack ER, et al. CheckMate 214: Efficacy and safety of nivolumab + ipilimumab (N+I) v sunitinib (S) for treatment-naïve advanced or metastatic renal cell carcinoma (mRCC), including IMDC risk and PD-L1 expression subgroups. Ann Oncol. 2017;28(suppl_5): mdx440.029.

Escudier B, Porta C, Schmidinger M, Rioux-Leclercq N, Bex A, Khoo V, et al. Renal cell carcinoma: ESMO Clinical Practice Guidelines for diagnosis, treatment and follow-up. Ann Oncol. 2016;27(suppl 5):v58-v68.

Fehrenbacher L, Spira A, Ballinger M, Kowanetz M, Vansteenkiste J, Mazieres J, et al. Atezolizumab versus docetaxel for patients with previously treated non-small-cell lung cancer (POPLAR): a multicentre, open-label, phase 2 randomised controlled trial. Lancet. 2016;387(10030):1837-46.

Fleming GF, Emens LA, Eder JP, Hamilton EP, Liu JF, Liu B, et al. Clinical activity, safety and biomarker results from a phase Ia study of atezolizumab (atezo) in advanced/recurrent endometrial cancer (rEC). J CLin Oncol. 2017;35:15_suppl, 5585.

Gabrilovich DI, Nagaraj S. Myeloid-derived suppressor cells as regulators of the immune system. Nat Rev Immunol. 2009;9(3):162-74.

Glodde N, Bald T, van den Boorn-Konijnenberg D, Nakamura K, O'Donnell JS, Szczepanski S, et al. Reactive Neutrophil Responses Dependent on the Receptor Tyrosine Kinase c-MET Limit Cancer Immunotherapy. Immunity. 2017;47(4):789-802.

Gonzalez-Angulo AM, Chen H, Karuturi MS, Chavez-MacGregor M, Tsavachidis S, Meric-Bernstam F, et al. Frequency of mesenchymal-epithelial transition factor gene (MET) and the catalytic subunit of phosphoinositide-3-kinase (PIK3CA) copy number elevationand correlation with outcome in patients with early stage breast cancer. Cancer. 2013;119:7–15.

Graff JN, Alumkal JJ, Drake CG, Thomas GV, Redmond WL, Farhad M, et al. Early evidence of anti-PD-1 activity in enzalutamide-resistant prostate cancer. Oncotarget. 2016;7(33):52810-52817.

Grothey A, Van Cutsem E, Sobrero A, Siena S, Falcone A, Ychou M, et al. Regorafenib monotherapy for previously treated metastatic colorectal cancer (CORRECT): an international, multicentre, randomised, placebo-controlled, phase 3 trial. Lancet. 2013;381(9863):303-312.

Hamanishi J, Mandai M, Iwasaki M, Okazaki T, Tanaka Y, Yamaguchi K, et al. Programmed cell death 1 ligand 1 and tumor-infiltrating CD8+ T lymphocytes are prognostic factors of human ovarian cancer. Proc Natl Acad Sci USA. 2007;104(9):3360–3365.

Hanna N, Johnson D, Temin S, Baker S Jr, Brahmer J, Ellis PM, et al. Systemic Therapy for Stage IV Non-Small-Cell Lung Cancer: American Society of Clinical Oncology Clinical Practice Guideline Update. J Clin Oncol. 2017;35(30):3484-3515.

Heller G, McCormack R, Kheoh T, Molina A, Smith MR, Dreicer R, et al. Circulating Tumor Cell Number as a Response Measure of Prolonged Survival for Metastatic Castration-Resistant Prostate Cancer: A Comparison With Prostate-Specific Antigen Across Five Randomized Phase III Clinical Trials. J Clin Oncol. 2018;36(6):572-580.

Hochster HS, Bendell JC, Cleary JM, Foster P, Zhang W, He X, et al. Efficacy and safety of atezolizumab (atezo) and bevacizumab (bev) in a phase Ib study of microsatellite

instability (MSI)-high metastatic colorectal cancer (mCRC). J Clin Oncol 2017;35: (4_suppl, 673).

Hossain DM, Pal SK, Moreira D, Duttagupta P, Zhang Q, Won H, et al. TLR9-Targeted STAT3 Silencing Abrogates Immunosuppressive Activity of Myeloid-Derived Suppressor Cells from Prostate Cancer Patients. Clin Cancer Res. 2015;21(16):3771-82.

Idorn M, Kollgaard T, Kongsted P, Sengelov L, Thor Straten P. Correlation between frequencies of blood monocytic myeloid-derived suppressor cells, regulatory T cells and negative prognostic markers in patients with castration-resistant metastatic prostate cancer. Cancer Immunol Immunother. 2014;63:1177–1187.

Infante JR, Braiteh F, Emens LA, Balmanoukian AS, Oaknin A, Wang Y, et al. Safety, clinical activity and biomarkers of atezolizumab (atezo) in advanced ovarian cancer (OC), Ann Oncol. 2016;27 (suppl_6):871P.

Kantoff PW, Higano CS, Shore ND, Berger ER, Small EJ, Penson DF, et al. Sipuleucel-T immunotherapy for castration-resistant prostate cancer. N Engl J Med. 2010;363:411-22.

Kelley RK, Verslype C, Cohn AL, Yang T-S, Su W-C, Burris, H et al. Cabozantinib in hepatocellular carcinoma: results of a phase 2 placebo-controlled randomized discontinuation study. Ann Oncol. 2017;28:528–534

Kim J, Shaffer D, Massard C, Powles T, Harshman L, Braiteh F, et al. A phase Ia study of safety and clinical activity of atezolizumab (atezo) in patients (pts) with metastatic castration-resistant prostate cancer (mCRPC). J Clin Oncol 2018;36 (no. 6_suppl):187.

Kudo M. Lenvatinib in Advanced Hepatocellular Carcinoma. Liver Cancer. 2017;6(4):253-263.

Kurzrock R, Sherman SI, Ball DW, Forastiere AA, Cohen RB, Mehra A, et al. Activity of XL184 (cabozantinib), an oral tyrosine kinase inhibitor, in patients with medullary thyroid cancer. J Clin Oncol. 2011;29:2660-6.

Krumbach R, Schuler J, Hofmann M, Giesemann T, Fiebig HH, Beckers T. Primary resistance to cetuximab in a panel of patient-derived tumour xenograft models: Activation of Met as one mechanism for drug resistance. Eur J Cancer. 2011;47:1231–1243.

Kwilas AR, Ardiani A, Donahue RN, Aftab DT, Hodge JW. Dual effects of a targeted small-molecule inhibitor (cabozantinib) on immune-mediated killing of tumor cells and immune tumor microenvironment permissiveness when combined with a cancer vaccine. J Transl Med. 2014;12:294.

La Rosée P. Treatment of hemophagocytic lymphohistiocytosis in adults. Hematology Am Soc Hematol Educ Program 2015;1:190-6.

La Rosée P, Horne A, Hines M, et al. Recommendations for the management of hemophagocytic lymphohistiocytosis in adults. Blood 2019;133:2465-77.

Leal TA, Campbell T, Mapes A, Schneider K, Staab MJ, Velastegui K, et al. MA 02.01 - Evidence of Clinical Activity of Sitravatinib in Combination with Nivolumab in NSCLC Patients Progressing on Prior Checkpoint Inhibitors. Poster presented at IASLC 2017 Chicago Multidisciplinary Symposium in Thoracic Oncology. 16 September 2017.

Lee DW, Santomasso BD, Locke FL, Ghobadi A, Turtle CJ, Brudno JN et al. ASTCT Consensus Grading for Cytokine Release Syndrome and Neurologic Toxicity Associated with Immune Effector Cells. Biol Blood Marrow Transplant. 2019;25(4):625-638.

Lemke E, Shah AY, Msaouel P, Bilen MA, Jonasch E, Venkatesan AM, et al. Comparing cabozantinib (C) treatment for a cohort of patients with metastatic clear cell (ccRCC) and variant histology renal cell carcinoma (vhRCC): A retrospective study. J Clin Oncol. 2018;36: 6_suppl, 702.

Llovet JM, Ricci S, Mazzaferro V, Hilgard P, Gane E, Blanc JF, et al. Sorafenib in advanced hepatocellular carcinoma. N Engl J Med 2008;359:378–390.

Lopes G, Wu Y, Kudaba I, Kowalski D, Cho B, Castro G, et al. Pembrolizumab (Pembro) Versus Platinum-Based Chemotherapy (Chemo) As First-Line Therapy For Advanced/Metastatic Nsclc With A Pd-L1 Tumor Proportion Score (Tps) ≥ 1%: Open-Label, Phase 3 Keynote-042 Study. J Clin Oncol 2018;36:(suppl; abstr LBA4).

Loriot Y, Rosenberg JE, Powles T, Necchi A, Hussain SA, Morales R, et al. Atezolizumab in Platinum-Treated Locally Advanced or Metastatic Urothelial Carcinoma (mUC): Updated OS, Safety and Biomarkers From the Phase II IMvigor210 Study. Ann Oncol. 27, 2016 (suppl. 6; 783P).

Lu X, Horner JW, Paul E, Shang X, Troncoso P, Deng P, et al. Effective combinatorial immunotherapy for castration-resistant prostate cancer. Nature. 2017;543(7647):728-732.

Lucey MR, Brown KA, Everson GT, et al. Minimal criteria for placement of adults on the liver transplant waiting list: A report of a national conference organized by the American Society of Transplant Physicians and the American Association for the Study of Liver Diseases. Liver Transpl Surg 1997;3:628–637.

Madoz-Gurpide J, Zazo S, Chamizo C, Casado V, Carames C, Gavin E, et al. Activation of Met pathway predicts poor outcome to cetuximab in patients with recurrent or metastatic head and neck cancer. J. Transl. Med. 2015;13:282.

Mandilaras V, Dhani NC, Tan Q, Jain A, Johnston C, Hirte HW, et al. Exploratory phase II evaluation of cabozantinib in recurrent/metastatic uterine carcinosarcoma (CS): A study of the Princess Margaret, Chicago, and California phase II consortia. J Clin Oncol. 2017 35:15_suppl, 5587.

Matulonis U, Sill M, Thaker P, Carlson J, Darus C, Mannel R, et al. NRG/GOG 186K: A randomized phase II study of NCI-supplied cabozantinib versus weekly paclitaxel in the treatment of persistent or recurrent epithelial ovarian, fallopian tube, or primary peritoneal cancer—Final results. Gynecol Oncol. 2016;141:207.

Merad M, Martin JC. Pathological inflammation in patients with COVID-19: a key role for monocytes and macrophages. Nat Rev Immunol. 2020;20:355-62.

McClain KL, Eckstein O. Clinical features and diagnosis of hemophagocytic lymphohistiocytosis. Up to Date [resource on the Internet]. 2014 [updated 29 October 2018; cited: 17 May 2019]. Available from: https://www.uptodate.com/contents/clinical-features-and-diagnosis-of-hemophagocytic-lymphohistiocytosis.

McDermott DF, Sosman JA, Sznol M, Massard C, Gordon MS, Hamid O, et al. Atezolizumab, an Anti-Programmed Death-Ligand 1 Antibody, in Metastatic Renal Cell Carcinoma: Long-Term Safety, Clinical Activity, and Immune Correlates From a Phase Ia Study. J Clin Oncol. 2016;34(8):833-42.

Michot JM, Bigenwald C, Champiat S, Collins M, Carbonnel F, Postel-Vinay S, et al. Immune-related adverse events with immune checkpoint blockade: a comprehensive review. Eur J Cancer. 2016;54:139-48.

Miller AM, Lundberg K, Ozenci V, Banham AH, Hellström M, Egevad L, et al. CD4+CD25 high T cells are enriched in the tumor and peripheral blood of prostate cancer patients. J Immunol. 2006;177:7398–7405.

Milowsky MI, Rumble RB, Booth CM, Gilligan T, Eapen LJ, Hauke RJ, et al. Guideline on Muscle-Invasive and Metastatic Bladder Cancer (European Association of Urology Guideline): American Society of Clinical Oncology Clinical Practice Guideline Endorsement. J Clin Oncol. 2016;34(16):1945-52.

Motzer RJ, Penkov K, Haanen J, Rini B, Albiges L, Campbell MT, et al. Avelumab plus Axitinib versus Sunitinib for Advanced Renal-Cell Carcinoma. N Engl J Med. 2019; 380 (12):1103-1115.

Motzer RJ, Powles T, Atkins MB, Escudier B, McDermott DF, Suarez C, et al. IMmotion151: A Randomized Phase III Study of Atezolizumab Plus Bevacizumab vs Sunitinib in Untreated Metastatic Renal Cell Carcinoma (mRCC). J Clin Oncol. 2018 36:6_suppl, 578.

Motzer RJ, Bacik J, Schwartz LH, Reuter V, Russo P, Marion, S, et al. Prognostic Factors for Survival in Previously Treated Patients With Metastatic Renal Cell Carcinoma. J Clin Oncol 2004; 22:454-463.

Nadal R, Mortzavi A, Stein M, Pal S, Lee D, Parnes H, et al. Clinical efficacy of cabozantinib plus nivolumab (CaboNivo) and CaboNivo plus ipilimumab (CaboNivoIpi) in patients (pts) with chemotherapy-refractory metastatic urothelial carcinoma (mUC) either naïve (n) or refractory (r) to checkpoint inhibitor (CPI). J Clin Oncol 2018;36:(suppl; abstr 4528).

Nadal R, Mortazavi A, Stein M, Pal SK, Davarpanah N, Parnes H, et al. 8640-Final Results of a Phase I study of Cabozantinib + Nivolumab and Cabozantinib + Nivolumab + Ipilimumab in patients with metastatic urothelial carcinoma and other genitourinary malignancies. Ann Oncol 2017;28:(suppl_5): v295-v329.

National Comprehensive Cancer Network. NCCN Clinical Practice Guidelines in Oncology, Breast Cancer, Version 1.2018

National Comprehensive Cancer Network. NCCN Clinical Practice Guidelines in Oncology, Head and Neck Cancers, Version 1.2018

National Comprehensive Cancer Network. NCCN Clinical Practice Guidelines in Oncology, Kidney Cancer, Version 4. 2019. NCCN.org.

National Comprehensive Cancer Network. NCCN Clinical Practice Guidelines in Oncology, Non-Small Cell Lung Cancer, Version 4.2018. NCCN.org.

National Comprehensive Cancer Network. NCCN Clinical Practice Guidelines in Oncology, Ovarian Cancer, Version 2.2018. NCCN.org.

National Comprehensive Cancer Network. NCCN Clinical Practice Guidelines in Oncology, Thyroid Carcinoma, Version 2.2017. NCCN.org.

National Comprehensive Cancer Network. NCCN Clinical Practice Guidelines in Oncology, Uterine Neoplasm, Version 1.2018. NCCN.org.

Neal JW, Dahlberg SE, Wakelee HA, Aisner SC, Bowden M, Huang Y, et al. Erlotinib, cabozantinib, or erlotinib plus cabozantinib as second-line or third-line treatment of patients with EGFR wild-type advanced non-small-cell lung cancer (ECOG-ACRIN 1512): a randomised, controlled, open-label, multicentre, phase 2 trial. Lancet Oncol 2016;17(12):1661-1671.

Nishino M, Giobbie-Hurder A, Gargano M, Suda M, Ramaiya NH, Hodi FS. Developing a Common Language for Tumor Response to Immunotherapy: Immune-Related Response Criteria Using Unidimensional Measurements. Clin Cancer Res. 2013;19(14):3936-43.

Naidoo J, Page DB, Li BT, Connell LC, Schindler K, Lacouture ME, et al. Toxicities of the anti-PD-1 and anti-PD-L1 immune checkpoint antibodies. Ann Oncol. 2015;26(12):2375-91. Erratum: 2016;27(7):1362.

NCT01984242. A Phase 2 Study of Atezolizumab (an Engineered Anti-PDL1 Antibody) as Monotherapy or in Combination With Avastin (Bevacizumab) Compared to Sunitinib

in Patients With Untreated Advanced Renal Cell Carcinoma [IMmotion150]. https://clinicaltrials.gov/ct2/show/NCT01984242.

NCT02420821, A Study of Atezolizumab in Combination With Bevacizumab Versus Sunitinib in Participants With Untreated Advanced Renal Cell Carcinoma. https://clinicaltrials.gov/ct2/show/NCT02420821.

Patnaik A, Swanson KD, Csizmadia E, Solanki A, Landon-Brace N, Gehring MP, et al. Cabozantinib Eradicates Advanced Murine Prostate Cancer by Activating Antitumor Innate Immunity. Cancer Discov. 2017;7(7):750-765.

Peters S, Gettinger S, Johnson ML, Jänne PA, Garassino MC, Christoph D, et al. Phase II Trial of Atezolizumab As First-Line or Subsequent Therapy for Patients With Programmed Death-Ligand 1-Selected Advanced Non-Small-Cell Lung Cancer (BIRCH). J Clin Oncol. 2017;35(24):2781-2789.

Pishvaian MJ, Lee MS, RyooB-Y, Stein S, Lee K-H, Verret W, et al. Phase 1b Study of Atezolizumab+ Bevacizumab in HCC—Update on Safety and Clinical Activity; Ann Oncol. 2018;29(Suppl8): Abstract LBA26.

Poveda AM, Selle F, Hilpert F, Reuss A, Savarese A, Vergote I, et al. Bevacizumab Combined With Weekly Paclitaxel, Pegylated Liposomal Doxorubicin, or Topotecan in Platinum-Resistant Recurrent Ovarian Cancer: Analysis by Chemotherapy Cohort of the Randomized Phase III AURELIA Trial. J Clin Oncol. 2015 Nov 10;33(32):3836-8.

Powles T, Duran I, van der Heijden M, Loriot Y, Vogelzang N, De Giogi U, et al. Atezolizumab versus chemotherapy in patients with platinum-treated locally advanced or metastatic urothelial carcinoma (IMvigor211): a multicentre, open-label, phase 3 randomised controlled trial. Lancet 2018;391(10122):748-757.

Pugh R, Murray-Lyon I, Dawson J, Pietroni M, Williams R. Transection of the oesophagus for bleeding oesophageal varices. British Journal of Surgery. 1973;60(8):646-9.

Reck M, Rodríguez-Abreu D, Robinson AG, Hui R, Csőszi T, Fülöp A1, et al. Pembrolizumab versus Chemotherapy for PD-L1-Positive Non-Small-Cell Lung Cancer. N Engl J Med. 2016 Nov 10;375(19):1823-1833.

Reck M, Socinski MA, Cappuzzo F, Orlandi F, Stroyakovskii D, Nogami N, et al. Primary PFS and safety analyses of a randomised Phase III study of carboplatin + paclitaxel +/− bevacizumab, with or without atezolizumab in 1L non-squamous metastatic NSCLC (IMpower150). Ann Oncol. 2017;28 (Supplement 11).

Ravelli A, Minoia F, Davi S, Horne A, Bovis F, Pistorio A, et al. 2016 classification criteria for macrophage activation syndrome complicating systemic juvenile idiopathic arthritis: a European League Against Rheumatism/American College of Rheumatology/Paediatric Rheumatology International Trials Organisation Collaborative Initiative. Ann Rheum Dis 2016;75:481-9.

Riegler LL, Jones GP, and Lee DW. Current approaches in the grading and management of cytokine release syndrome after chimeric antigen receptor T-cell therapy. Ther Clin Risk Manag 2019;15:323-35.

Rini BI, Powles T, Atkins MB, Escudier B, McDermott DF, Suarez C, et al. Atezolizumab plus bevacizumab versus sunitinib in patients with previously untreated metastatic renal cell carcinoma (IMmotion151): a multicentre, open-label, phase 3, randomised controlled trial. Lancet. 2019;393(10189):2404-2415.

Rini BI, Plimack ER, Stus V, Gafanov R, Hawkins R, Nosov D, et al. Pembrolizumab plus Axitinib versus Sunitinib for Advanced Renal-Cell Carcinoma. N Engl J Med. 2019 380 (12):1116-1127.

Rittmeyer A, Barlesi F, Waterkamp D, Park K, Ciardiello F, von Pawel J, et al. Atezolizumab versus docetaxel in patients with previously treated non-small-cell lung cancer (OAK): a phase 3, open-label, multicentre randomised controlled trial. Lancet. 2017;389(10066):255-265.

Rosenberg JE, Hoffman-Censits J, Powles T, van der Heijden MS, Balar AV, Necchi A, et al. Atezolizumab in patients with locally advanced and metastatic urothelial carcinoma who have progressed following treatment with platinum-based chemotherapy: a single-arm, multicentre, phase 2 trial. Lancet. 2016;387(10031):1909-20.

Rotz SJ, Leino D, Szabo S, Mangino JL, Turpin BK, and Pressey JG. Severe cytokine release syndrome in a patient receiving PD-1-directed therapy. Pediatr Blood Cancer 2017;64:e26642.

Schmid P, Cruz C, Braiteh FS, Eder JP, Tolaney S, Kuter I, et al. Atezolizumab in metastatic triple-negative breast cancer: long-term clinical outcomes and biomarker analyses [abstract]. In: Proceedings of the American Association for Cancer Research Annual Meeting 2017; 2017 Apr 1–5; Washington, DC. Philadelphia (PA): AACR; Cancer Res 2017;77(13 Suppl):Abstract 2986.

Schmid P, Adams S, Rugo HS, Schneeweiss A, Barrios CH, Iwata H, et al. Atezolizumab and Nab-Paclitaxel in Advanced Triple-Negative Breast Cancer. N Engl J Med. 2018; 379(22):2108-2121.

Schöffski P, Gordon M, Smith DC, Kurzrock R, Daud A, Vogelzang NJ, et al. Eur J Cancer. Phase II randomised discontinuation trial of cabozantinib in patients with advanced solid tumours. 2017;86:296-304.

Schram AM, Berliner N. How I treat hemophagocytic lymphohistiocytosis in the adult patient. Blood. 2015;125:2908-14.

Sennino B, Ishiguro-Oonuma T, Wei Y, Naylor RM, Williamson CW, Bhagwandin V, et al. Suppression of tumor invasion and metastasis by concurrent inhibition of c-Met and VEGF signaling in pancreatic neuroendocrine tumors. Cancer Discov. 2012;2(3):270-87.

Shojaei F, Lee JH, Simmons BH, Wong A, Esparza CO, Plumlee PA, et al. HGF/c-Met acts as an alternative angiogenic pathway in sunitinib-resistant tumors. Cancer Res 2010;70:10090-100.

Smith M, De Bono J, Sternberg C, Le Moulec S, Oudard S, De Giorgi U, et al. Phase III Study of Cabozantinib in Previously Treated Metastatic Castration-Resistant Prostate Cancer: COMET-1. J Clin Oncol. 2016;34(25):3005-13.

Smith MR, Sweeney C, Corn PG, Rathkopf DE, Smith DC, Hussain M, et al. Cabozantinib in chemotherapy-pretreated metastatic castration-resistant prostate cancer: Results of a Phase II non-randomized expansion study. J Clin Oncol. 2014; 32:3391-9.

Smith DC, Smith MR, Sweeney C, Elfiky AA, Logothetis C, Corn PG, et al. Cabozantinib in patients with advanced prostate cancer: Results of a phase II randomized discontinuation trial. J Clin Oncol. 2013;31:412-419.

Socinski MA, Jotte RM, Cappuzzo F, Orlandi F, Stroyakovskiy D, Nogami N, et al. Atezolizumab for First-Line Treatment of Metastatic Nonsquamous NSCLC. N Engl J Med. 2018;378(24):2288-2301.

Socinski MA, Jotte RM, Cappuzzo F, Orlandi FJ, Stroyakovskiy D, Nogami N, et al. Overall survival (OS) analysis of IMpower150, a randomized Ph 3 study of atezolizumab (atezo) + chemotherapy (chemo) ± bevacizumab (bev) vs chemo + bev in 1L nonsquamous (NSQ) NSCLC. J Clin Oncol. 2018;36:(suppl; abstr 9002).

Song E, Tai W, Messersmith W, Baby S, Purkey A, Quackenbush K, et al. Potent antitumor activity of cabozantinib, a c-MET and VEGFR2 inhibitor, in a colorectal cancer patient-derived tumor explant model. Internat J Canc 2015;136:1967-1975.

Stein S, Pishvaian MJ, Lee MS, Lee K-H, Hernandez S, Kwan A, et al. Safety and clinical activity of 1L atezolizumab + bevacizumab in a phase Ib study in hepatocellular carcinoma (HCC). J Clin Oncol 2018;36:(suppl; abstr 4074).

Sternberg CN, de Mulder P, Schornagel JH, Theodore C, Fossa SD, van Oosterom AT, et al. Seven year update of an EORTC phase III trial of high-dose intensity M-VAC chemotherapy and G-CSF versus classic M-VAC in advanced urothelial tract tumours. Eur J Cancer. 2006;42(1):50-4.

Strickler JH, Rushing CN, Uronis HE, Morse M, Blobe GC, Zafar Y, et al. Phase Ib study of cabozantinib plus panitumumab in KRAS wild-type (WT) metastatic colorectal cancer (mCRC). J Clin Oncol. 2016;34:(15_suppl, 3548).

Taieb J, Moehler M, Boku N, Ajani JA, Yañez Ruiz E, Ryu MH, et al. Evolution of checkpoint inhibitors for the treatment of metastatic gastric cancers: Current status and future perspectives. Cancer Treat Rev. 2018;66:104-113.

Tolaney SM, Ziehr DR, Guo H, Ng MR, Barry WT, Higgins MJ, et al. Phase II and Biomarker Study of Cabozantinib in Metastatic Triple-Negative Breast Cancer Patients. Oncologist. 2017;22:25–32.

Tolaney SM, Ziehr DR, Guo H, Ng MR, Barry WT, Higgins MJ, et al. Phase II and Biomarker Study of Cabozantinib in Metastatic Triple-Negative Breast Cancer Patients. Oncologist. 2016;21:1-8.

Turnage RH, Badgwell B. Abdominal wall, umbilicus, peritoenum, mesentery, omentum and

retroperitoneum. In: Townsend CM Jr, Beauchamp RD, Evers BM, Mattox KL, editors. Sabiston Textbook of Surgery. 20th ed. 2016.

Vanderstraeten A, Luyten C, Verbist G, Tuyaerts S, Amant F. Mapping the immunosuppressive environment in uterine tumors: implications for immunotherapy. Cancer Immunol Immunother. 2014;63(6):545-57.

Vergote IB, Smith DC, Berger R, Kurzrock R, Vogelzang NJ, Sella A, et al. A phase 2 randomised discontinuation trial of cabozantinib in patients with ovarian carcinoma. Eur J Cancer. 2017;83:229-236.

von der Maase H, Sengelov L, Roberts JT, Ricci S, Dogliotti L, Oliver T, et al. Long-term survival results of a randomized trial comparing gemcitabine plus cisplatin, with methotrexate, vinblastine, doxorubicin, plus cisplatin in patients with bladder cancer. J Clin Oncol. 2005;23(21):4602-8.

von der Maase H, Hansen SW, Roberts JT, Dogliotti L, Oliver T, Moore MJ, et al. Gemcitabine and cisplatin versus methotrexate, vinblastine, doxorubicin, and cisplatin in advanced or metastatic bladder cancer: results of a large, randomized, multinational, multicenter, phase III study. J Clin Oncol. 2000;18(17):3068-77.

Vuk-Pavlović S, Bulur PA, Lin Y, Qin R, Szumlanski CL, Zhao X, et al. Immunosuppressive CD14+HLA-DRlow/- monocytes in prostate cancer. Prostate. 2010;70(4):443-55.

Wallin J, Pishvaian MJ, Hernandez G, Yadav M, Jhunjhunwala S, Delamarre L, et al. Abstract 2651: Clinical activity and immune correlates from a phase Ib study evaluating atezolizumab (anti-PDL1) in combination with FOLFOX and bevacizumab (anti-VEGF) in metastatic colorectal carcinoma. Proceedings: AACR 107th Annual Meeting 2016; April 16-20, 2016; New Orleans, LA.

Willson KJ, Nott LM, Broadbridge VT, Price T. Hepatic encephalopathy associated with cancer or anticancer therapy. Gastrointest Cancer Res. 2013; 6(1):11-6.

Witjes JA, Lebret T, Compérat EM, Cowan NC, De Santis M, Bruins HM, et al. Updated 2016 EAU Guidelines on Muscle-invasive and Metastatic Bladder Cancer. Eur Urol. 2017;71(3):462-475.

Wong PY, Xia V, Imagawa DK, Hoefs J, Hu KQ. Clinical presentation of hepatocellular carcinoma (HCC) in Asian-Americans versus non-Asian-Americans. J Immigr Minor Health. 2011;13(5):842-8.

Wolchock JD, Hoos A, O'Day S, Weber JS, Hamid O, Lebbé C, et al. Guidelines for the Evaluation of Immune Therapy Activity in Solid Tumors: Immune Therapy Activity in Solid Tumors: Immune-Related Response Criteria. Clin Cancer Res. 2009;15(23):7412-20.

Wright JR, Ung YC, Julian JA, Pritchard KI, Whelan TJ, Smith C, et al. Randomized, double-blind, placebo-controlled trial of erythropoietin in non-small-cell lung cancer with disease-related anemia. J Clin Oncol. 2007;25(9):1027-32.

Zagouri F, Bago-Horvath Z, Rössler F, Brandstetter A, Bartsch R, Papadimitriou CA, et al. High MET expression is an adverse prognostic factor in patients with triple-negative breast cancer. Br J Cancer. 2013;108:1100–1105.

Zhou L, Liu XD, Sun M, Zhang X, German P, Bai S, et al. Targeting MET and AXL overcomes resistance to sunitinib therapy in renal cell carcinoma. Oncogene. 2016;35(21):2687-97.

[redacted]

[redacted]

[redacted]

[redacted]

[redacted]

[redacted]

[redacted]

Appendix B: Schedule of Assessments for the Expansion Stage (Combination-Therapy Cohorts, [redacted]

The schedule of required assessments for the Combination-Therapy Expansion Cohorts, [redacted] is presented in this appendix in the table below.

Most study assessments and procedures (including treatment administration) will be performed in cycles. Cycle 1 Day 1 (C1D1) is defined as the date of first dose of any study treatment.

Cycles for the Expansion Cohorts: Cycles may extend beyond 21 days if atezolizumab dosing is delayed. A cycle is generally the 21-day interval starting with the date of an atezolizumab infusion and ending with the day before the next atezolizumab infusion. However, if atezolizumab treatment is discontinued but cabozantinib treatment is allowed to continue with the notification of the Sponsor, each consecutive 21-day interval starting with the date of the decision to discontinue atezolizumab will be defined as a cycle. If the decision to discontinue atezolizumab occurs less than 21 days after the last infusion, then the next cycle will begin on the 22nd day after the last infusion. The date of the decision to discontinue study treatment is defined for each subject as the later of (a) the date of the decision of the investigator to permanently discontinue study treatment or (b) the date of the last dose of study treatment taken by the subject.

During an atezolizumab dose delay, subjects should return to the site for scheduled safety visits every three weeks from the last dose of atezolizumab. Further, the study site should perform unscheduled visits or telephone calls weekly (or more frequently) as clinically indicated to monitor subject safety and appropriateness for re-treatment with study treatment. Other unscheduled visits are permitted whenever necessary. See Section 5.5 for further details.

[redacted]

[redacted]

[redacted]

Imaging assessments (CT, MRI, bone scan) are to be performed at protocol-defined, fixed intervals based on the first dose of study treatment (defined as Week 1 Day 1 [W1D1]); all subsequent time points for these assessments will apply the same nomenclature, which will not be modified as a result of modifications or discontinuations of treatment administration.

Unless otherwise indicated, in the absence of side effects all scheduled visits will occur within windows for the protocol-specified visit schedule. If the subject experiences side effects, study treatment can be modified or delayed as described in Section 6.5. If the subject is unable to have a study assessment taken within the defined time window due to an event outside of his or her control (eg, clinic closure, personal emergency, inclement weather, vacation), the assessment should be performed as close as possible to the required schedule. Special accommodations during the global COVID-19 pandemic are described in Appendix M. Laboratory panels for serum chemistry, hematology, and urinalysis are defined in Section 5.6.5.

Appendix B: Schedule of Assessments for the Expansion Stage (Combination-Therapy Cohorts, [redacted]

	Pre-enrollment	Post-enrollment				
Assessment:	Screening[a] (Before First Dose)	Cycle 1 (± 3 days)	Cycles 2 through 8 (± 3 days)	Cycles 9 and Above (± 5 days)	30-Day Post-Treatment Follow-Up (+14 days)	Extended Follow Up
Informed consent (Section 5.1)	X[b]					
Demographics, medical and cancer history (Section 5.6.1)	≤ 28 days					
Physical examination[c] + weight (Section 5.6.2)	≤ 28 days (with height)	C1D1 (predose)	Day 1 of every cycle or every three weeks after the last dose of atezolizumab (if infusions are delayed), whichever is earlier.		X	
ECOG Performance status (Section 5.6.2, Appendix F)	≤ 28 days (+ Karnofsky for RCC subjects)	C1D1	Day 1 of every cycle or every three weeks after the last dose of atezolizumab (if infusions are delayed), whichever is earlier.		X	
Vital signs (Section 5.6.3)	≤ 28 days	C1D1[d]	Day 1 of every cycle or every three weeks after the last dose of atezolizumab (if infusions are delayed)[d], whichever is earlier.		X	
12-lead ECG (Section 5.6.4)[e]	≤ 14 days	C1D1[f] predose	Day 1 of every 4th cycle starting with C3D1 (ie, C3D1, C7D1, etc) or every 12 weeks after the last dose of atezolizumab (if infusions are delayed), whichever is earlier.		X	
Hematology and Chemistry by central lab (Section 5.6.5)	≤ 14 days	C1D1[g,h] predose	Day 1 of every cycle or every three weeks after the last dose of atezolizumab (if infusions are delayed)[g,h], whichever is earlier.		X	
Tumor markers: [redacted], AFP (HCC) [redacted] (Section 5.6.9.4)	≤ 28 days		[redacted], AFP (HCC), [redacted] Day 1 of every 3 cycle (or every 9 weeks, whichever is earlier) for first 12 months and Day 1 of every 5th cycle (or every 15 weeks, whichever is earlier) thereafter until the earlier of initiation of subsequent systemic anticancer therapy or permanent loss to radiographic follow-up (including hospice admission).			
Follicle stimulating hormone by central lab (Section 5.6.5)[a, i]	≤ 28 days					
[redacted]	[redacted]					
Hepatitis screening[j] by central lab (Section 5.6.5)	X					

Appendix B: Schedule of Assessments for the Expansion Stage (Combination-Therapy Cohorts, [redacted]

	Pre-enrollment	Post-enrollment				
**Assessment:**	**Screening[a] (Before First Dose)**	**Cycle 1 (± 3 days)**	**Cycles 2 through 8 (± 3 days)**	**Cycles 9 and Above (± 5 days)**	**30-Day Post-Treatment Follow-Up (+14 days)**	**Extended Follow Up**
PT/INR and PTT by central lab (Section 5.6.5)	≤ 14 days	C1D1[f] predose	Day 1 of every 3rd cycle starting with C3D1 (ie, C3D1, C6D1, etc) or every nine weeks after the last dose of atezolizumab (if infusions are delayed), whichever is earlier.		X	
Urinalysis by **local** lab (Section 5.6.5)	≤ 14 days	C1D1[g] predose	Day 1 of every cycle or every three weeks after the last dose of atezolizumab (if infusions are delayed)[g], whichever is earlier.		X	
Urine chemistry incl. UPCR by central lab (Section 5.6.5)	≤ 14 days	C1D1[f] predose	Day 1 of every other cycle starting with C3D1 (ie, C3D1, C5D1, etc) or every six weeks after the last dose of atezolizumab (if infusions are delayed) , whichever is earlier.		X	
Pregnancy test by **local** lab (Section 5.6.5)	≤ 7 days (serum)	C1D1[f] predose (serum)	Day 1 of every cycle or every three weeks after the last dose of atezolizumab (if infusions are delayed) (serum or urine), whichever is earlier.			
Thyroid function test by central lab (Section 5.6.5)	≤ 14 days	C1D1[f] predose	Day 1 of every 3rd cycle starting with C3D1 (ie, C3D1, C6D1, etc) or every nine weeks after the last dose of atezolizumab (if infusions are delayed), whichever is earlier.		X	
Archival tumor tissue sample[k] (Section 5.6.8)	X					
Optional tumor biopsy (Section 5.6.8)	After the first dose of study treatment (6 weeks or later but prior to progressive disease) if archival tissue is evaluable. If archival samples are not evaluable, then tissue may be collected before the first dose of study treatment. If tumor biopsies are to be performed prior to first dose of study treatment, cabozantinib treatment will not be given until complete wound healing has occurred; if optional tumor biopsies are to be performed after first dose, cabozantinib treatment must be interrupted for at least 5 days before optional tumor biopsies are performed and may not be reinitiated until adequate wound healing has occurred.					

Appendix B: Schedule of Assessments for the Expansion Stage (Combination-Therapy Cohorts, [redacted]

	Pre-enrollment	Post-enrollment				
**Assessment:**	**Screening[a] (Before First Dose)**	**Cycle 1 (± 3 days)**	**Cycles 2 through 8 (± 3 days)**	**Cycles 9 and Above (± 5 days)**	**30-Day Post-Treatment Follow-Up (+14 days)**	**Extended Follow Up**
Tumor assessment: CT/MRI **Chest, Abdomen, Pelvis, Neck** (Section 5.6.9)	≤ 28 days	Unless otherwise described, CT of the chest, abdomen, and pelvis or CT of the chest with MRI of the abdomen and pelvis will be performed in all subjects at screening and every 6 weeks (± 5 days) after first dose (at W7D1, W13D1 etc). Upon completion of 12 months on study, these assessments will be performed every 12 weeks (± 7 days). [redacted]<br>CT/MRIs are to be performed per the protocol-defined schedule regardless of whether study treatment is reduced, interrupted, delayed, or discontinued, and the tumor assessment schedule is independent of the atezolizumab dosing schedule. Tumor imaging will continue until radiographic disease progression per RECIST 1.1 as determined by the investigator. For subjects who discontinue study treatment before radiographic disease progression per RECIST 1.1, regularly scheduled imaging assessments should continue if possible until radiographic progression per RECIST 1.1 or initiation of subsequent anticancer therapy.<br>PR or CR per RECIST 1.1 at a given time point must be confirmed by repeat assessments ≥ 4 weeks after the criteria for response are first met. Subjects with PD per RECIST 1.1 who continue with study treatment are to have tumor measurement outcomes confirmed ≥ 4 weeks after the initial PD criteria were met. For subjects who continue treatment after the confirmatory tumor scans, regularly scheduled imaging will continue.				
Tumor assessment: MRI/CT **Brain** (Section 5.6.9)	≤ 28 days	MRI (or CT) of the brain will be performed at screening [redacted] for subjects with other tumor indications who have a history or clinical symptoms of brain metastasis. After first dose, MRI (or CT) scans of the brain are only required in subjects with documented, treated brain metastasis. Assessments will be performed every 12 weeks (± 7 days) after first dose (at W13D1, W26D1 etc). The schedule for these assessments is independent of the atezolizumab dosing schedule. To ensure image consistency, the same imaging modalities and acquisition protocols used at screening are to be used for subsequent tumor assessments. (Note: in order to meet the eligibility requirements of the study, brain metastasis must have been treated and stable for at least 4 weeks before first dose of study treatment. Subjects without documented brain metastasis during the screening assessment are not required to undergo brain imaging after starting treatment unless clinically indicated).<br>CT/MRIs are to be performed per the protocol-defined schedule regardless of whether study treatment is reduced, interrupted, delayed, or discontinued, and the tumor assessment schedule is independent of the atezolizumab dosing schedule. Tumor imaging will continue until radiographic disease progression per RECIST 1.1 as determined by the investigator. For subjects who discontinue study treatment before radiographic disease progression per RECIST 1.1, regularly scheduled imaging assessments should continue if possible until radiographic progression per RECIST 1.1 or initiation of subsequent anticancer therapy.				
Bone scans (Section 5.6.9)	≤ 28 days	Technetium bone scans (TBS) will be performed at screening [redacted] for subjects with other tumor indications who have a history or clinical symptoms (ie, bone pain) of bone metastases. After study treatment initiation bone scans are only required in subjects with documented bone lesions or if clinically indicated by signs and symptoms suggestive of new bone metastases. Assessments after the first dose will follow routine clinical practice (approximately every 12 weeks throughout the first 12 months and every 24 weeks thereafter). Bone scan findings alone cannot be used for the determination of progression or response in this study and need to be corroborated by CT/MRI. Bone lesions corroborated by CT/MRI must be reported as non-target or new lesions. PET scan or plain films are not considered adequate imaging techniques to measure bone lesions. Bone scan evaluations will end on the date of last CT/MRI scan. If the bone scan schedule does not coincide with the last CT/MRI scan, no additional bone scan is needed after the last CT/MRI has been performed.				

Appendix B: Schedule of Assessments for the Expansion Stage (Combination-Therapy Cohorts, [redacted]

	Pre-enrollment	Post-enrollment				
Assessment:	Screening[a] (Before First Dose)	Cycle 1 (± 3 days)	Cycles 2 through 8 (± 3 days)	Cycles 9 and Above (± 5 days)	30-Day Post-Treatment Follow-Up (+14 days)	Extended Follow Up
**Pharmacokinetic and Biomarker Blood Sample Assessment for the *Standard Dosing Schedule***						
PK blood samples[l] (Section 5.6.6.1)		C1D1 (Combination-Therapy Expansion Cohorts: before treatment, approximately 5 min after atezolizumab infusion & 2 h after cabozantinib dose; [redacted]) and predose on C2D1 and C3D1.				
Blood sample–Pharmacogenetic (Section 5.6.7)		X predose				
Blood sample–immune cell profiling by FACS[m] (Section 5.6.8)		Predose on C1D1 and C2D1. (may be performed at selected sites)				
Blood sample—Immunogenicity (Section 5.6.7)		Predose on C1D1, C3D1, and C7D1 predose (for the Expansion Cohorts [redacted]			X (for the Expansion Cohorts [redacted]	
Blood sample–serum/plasma biomarker[m] (Section 5.6.8)		Predose on C1D1, C2D1, and C3D1 An optional sample may be collected at the first sign of progression per the investigator.				
Blood sample–cell and/or plasma pharmacogenomics [m] (Section 5.6.8)		Predose on C1D1, C2D1, and C3D1 An optional sample may be collected at the first sign of progression per the investigator.				
Concomitant medication (Section 7)	Document concomitant medication taken from 28 days before first dose of study treatment through 30 days after the date of the decision to discontinue study treatment					
Adverse events (Sections 8.1, 8.2, and 8.3.1)	Document new or worsening AEs from informed consent through 30 days (90 days for AESIs) after the date of the decision to permanently discontinue study treatment in source documents. AE information will be collected at study visits and may also be collected at any time over the phone or by spontaneous subject report. See the CRF Completion Guidelines for instructions on entering these data on Medical History and/or AE CRFs and Section 8.2 for SAE reporting requirements. At the date of the first dose of study treatment, AEs will be documented pre- and post-dose. Certain AEs and all SAEs that are ongoing at the time of the Post-Treatment Follow-Up Visit 30 (+14) days after the date of the decision to permanently discontinue study treatment are to be followed until resolution or determination by the investigator that the event is stable or irreversible (see Section 8.4).					

Appendix B: Schedule of Assessments for the Expansion Stage (Combination-Therapy Cohorts, Exploratory [redacted]

	Pre-enrollment	Post-enrollment				
**Assessment:**	**Screening[a] (Before First Dose)**	**Cycle 1 (± 3 days)**	**Cycles 2 through 8 (± 3 days)**	**Cycles 9 and Above (± 5 days)**	**30-Day Post-Treatment Follow-Up (+14 days)**	**Extended Follow Up**
Atezolizumab dosing[n] [redacted]		Atezolizumab will be administered by IV infusion at the clinic; first infusion on C1D1; Subsequent atezolizumab infusions will be administered every three weeks (-2 days) on Day 1 of each cycle until study treatment is discontinued.				
Cabozantinib dosing [redacted]		Cabozantinib will administered in clinic C1D1 and then will be taken once daily at home until study treatment is discontinued				
Dispense/return of oral study drug and compliance accounting[o] (Section 6.3)		Cabozantinib is to be dispensed to subjects every 3 weeks				
Additional anticancer treatment and survival status (Sections 5.3 and 5.6.11)						Every 12 weeks (± 14 days) after 30-day post-treatment follow-up visit until death

[a] Results of screening assessments must be reviewed before first dose of study treatment to confirm that the subject meets the eligibility criteria.

[b] Informed consent may be obtained greater than 28 days prior to first dose of study treatment, but must be provided before any study-specific procedures are performed; however evaluations performed as part of routine care prior to informed consent can be utilized as screening evaluations if permitted by the site's IRB/EC policies.

[c] Symptom-directed physical examination will be conducted on C1D1 before first dose of study treatment and at subsequent safety assessment visits.

[d] Vital signs should always be assessed within 60 min prior to initiation of atezolizumab infusions, and further vital sign assessment should be performed during and after the infusion as clinically indicated.

[e] Additional ECGs are to be performed if clinically indicated.

[f] This assessment is intended to confirm suitability for treatment after screening and prior to first dose. If this assessment has been performed during screening within 14 days (7 days for pregnancy test) prior to first dose (C1D1), this assessment does not need to be performed on C1D1 unless the subject's clinical status has changed (eg, onset of new symptoms indicative of clinical deterioration). If the assessment is performed on C1D1, the results must be available to and reviewed by the investigator prior to any treatment being administered.

[g] Serum chemistry, hematology, and urinalysis laboratory samples must be collected and the results must be reviewed within 72 h before any atezolizumab infusion administered on study.

[h] Local laboratory assessments for these panels may be obtained and used if the results are required by the investigator in a rapid timeframe. See Section 5.6.5 and the Laboratory Manual for more detailed information on laboratory assessments.

[i] For women under the age of 55 years to confirm menopause as needed.

[j] For all tumor types except HCC, Hepatitis B surface antigen and Hepatitis C antibody (with reflex testing of HCV RNA if antibody test is positive) are to be assessed at screening. For HCC subjects, Hepatitis B surface antigen, Hepatitis B core antibody, Hepatitis B e-antigen/e-antibody, Hepatitis B DNA, Hepatitis D testing for Hepatitis B positive, Hepatitis C antibody, and Hepatitis C virus RNA are to be assessed at screening.

[k] Tumor tissue (archival) will be obtained prior to first dose of study treatment whenever available. Formalin-fixed paraffin embedded (FFPE) tumor blocks are preferred but in cases where this is not possible, tumor slides should be obtained. See Translational Medicine Laboratory Manual for specific instructions.

[l] Plasma samples will be collected for cabozantinib PK in the Combination-Therapy Expansion Cohorts [redacted] Serum samples will be collected for atezolizumab PK in the Expansion Cohorts [redacted] Pre-dose PK samples for cabozantinib and atezolizumab for C1D1, C2D1, and C3D1 for the Combination-Therapy Expansion [redacted] should be collected within 15 minutes before atezolizumab dose; it is acceptable to draw this sample up to 1 hour prior to atezolizumab dose. [redacted] The investigator will ask the subject for the date and time of the most recent prior dose of cabozantinib, and this information will be recorded on the appropriate CRF page.

[m] An additional blood sample should be collected if an optional tumor tissue sample is obtained and such tissue sample collection does not coincide with scheduled blood collection for biomarker analysis.

[n] Atezolizumab doses are not to be administered less than 19 days apart.

[o] In exceptional circumstances (eg, COVID-19 pandemic), alternative methods of distribution of oral treatment to subjects may be considered in accordance with the study site's local policies and all applicable regulations.

[redacted]

[redacted]

[redacted]

[redacted]

[redacted]

[redacted]

[redacted]

[redacted]

[redacted]

[redacted]

[redacted]

[redacted]

[redacted]

[redacted]

[redacted]

[redacted]

[redacted]

[redacted]

[redacted]

[redacted]

[redacted]

[redacted]

[redacted]

[redacted]

[redacted]

[redacted]

[redacted]

[redacted]

[redacted]

[redacted]

[redacted]

[redacted]

[redacted]

[redacted]

[redacted]

[redacted]

Appendix D: Maintenance Phase

The purpose of the Maintenance Phase is to continue to provide long-term access to study drug(s) to subjects who are deriving clinical benefit even after evaluation of the study objectives has been completed. When sufficient data have been collected to adequately evaluate all study endpoints, and upon site notification by the Sponsor, subjects remaining on study treatment or who have not completed the Post-Treatment Follow-Up Visit will enter the study Maintenance Phase. Upon initiation of the Maintenance Phase, the Sponsor considers the safety and efficacy profile of the drug within this study to have been sufficiently established for regulatory purposes.

In the Maintenance Phase subjects who remain on treatment will continue to receive study treatment until a criterion for protocol-defined discontinuation has been met (protocol Section 3.8). Subjects are to undergo periodic safety assessments (including local laboratory tests) and tumor assessments; the nature and frequency of these assessments are to be performed per standard of care if allowed per local regulations. It is the Investigator's responsibility to ensure that subject visits occur frequently enough and adequate assessments are performed to ensure subject safety.

Subjects who enter the Maintenance Phase after discontinuing study treatment, but prior to their Post-Treatment Follow Up Visit, are to be followed in the Maintenance Phase until their Post-Treatment Follow Up Visit.

In order to continue to collect important safety information on subjects still enrolled in the study, reporting of SAEs, AESIs, and other reportable events (pregnancy and medication errors with sequelae) is to continue per protocol (Sections 8.1-8.5).

Further, the following AEs, whether serious or not, are to be reported using the same process as for reporting SAEs described in protocol Section 8.2 (though SAE reporting timeline requirements do not apply to non-serious events reported in these categories):

- Adverse Events (including irAEs), whether serious or not, leading to study treatment discontinuation
- Adverse Events (including irAEs), whether serious or not, leading to study treatment dose modification (ie, causing study treatment to be interrupted, delayed, or reduced)

Study drug accountability is to continue as described in Section 6.4.

See Maintenance Phase Schedule of Assessments below. To receive study treatment supplies it may be necessary for subjects to visit the study site more frequently than clinic visits for safety and tumor evaluations performed per standard of care.

Site monitoring visits will occur at a reduced frequency to ensure adherence to GCP, protocol compliance, adequate subject safety follow-up, study drug accountability, and reporting of SAEs and other reportable events.

During the Maintenance Phase no data are to be entered into CRFs. Study central laboratory samples are not to be obtained. Do not submit local laboratory results to the study local laboratory management vendor, radiographic images to the study central imaging vendor, or ECGs to the study central ECG vendor.

Schedule of Assessments for the Maintenance Phase

Assessment	Study Period / Visit	
	**While Subject is Receiving Study Treatment (Until Treatment is Permanently Discontinued)**	**Post-Treatment Follow-Up Visit**
Study drug accountability	Every time study drug is dispensed	✓[a]
Study treatment	Atezolizumab: Once every 3 weeks (-2 days); Cabozantinib: Daily<br>Study treatment may continue until a criterion for discontinuation is met (Section 3.6). Subjects in combination treatment cohorts may be allowed to discontinue treatment with one component of the combination and continue on the other with the notification of the Sponsor.	
Safety evaluation: *Clinical examination and local laboratory assessments per SOC*	Frequency per standard of care	
Reporting of SAEs, AESIs, and other reportable events (pregnancy and medication errors with sequelae)	Submit reports to Sponsor per Sections 8.1-8.5	
Reporting of AEs (including irAEs), serious or not:<br>• leading to study treatment discontinuation<br>• leading to study treatment dose modification (ie, causing study treatment to be interrupted, delayed, or reduced)	Submit reports to Sponsor per the same process as for reporting SAEs per Section 8.2<br><br>SAE reporting timeline requirements do not apply to non-serious events reported in these categories	
Tumor assessments: *Imaging methods per SOC*	Frequency per standard of care	

AE, adverse event; irAE, immune-related adverse event; SAE, serious adverse event; SOC, standard of care.
No data will be entered into electronic case report forms. Do not submit local laboratory results to the study local laboratory management vendor.

[a] A post-treatment visit may be required for the purpose of returning all unused study medication still in the subject's possession.

Appendix E: Preexisting Autoimmune Diseases and Immune Deficiencies

Subjects should be carefully questioned regarding their history of acquired or congenital immune deficiencies or autoimmune disease. Subjects with any history of immune deficiencies or autoimmune disease listed in the table below are excluded from participating in the study. Possible exceptions to this exclusion could include:

- Subjects with a medical history of such entities as atopic disease or childhood arthralgias where the clinical suspicion of autoimmune disease is low
- Subjects with a history of autoimmune-related hypothyroidism on a stable dose of thyroid replacement hormone, controlled Type 1 diabetes mellitus and on an insulin regimen, or asthma
- Subjects with transient autoimmune manifestations of an acute infectious disease that resolved upon treatment of the infectious agent (eg, acute Lyme arthritis)

Caution should be used when considering atezolizumab for subjects who have previously experienced a severe or life–threatening skin adverse reaction while receiving another immunostimulatory anti-cancer agent.

The Sponsor is available to advise on any uncertainty over autoimmune exclusions.

Autoimmune Diseases and Immune Deficiencies

Acute disseminated encephalomyelitis	Dermatomyositis	Neuromyotonia
Addison disease	Diabetes mellitus type 1	Opsoclonus myoclonus syndrome
Ankylosing spondylitis	Dysautonomia	Optic neuritis
Antiphospholipid antibody syndrome	Epidermolysis bullosa acquisita	Ord thyroiditis
Aplastic anemia	Gestational pemphigoid	Pemphigus
Autoimmune hemolytic anemia	Giant cell arteritis	Pernicious anemia
Autoimmune hepatitis	Goodpasture syndrome	Polyarteritis nodosa
Autoimmune hypoparathyroidism	Graves disease	Polyarthritis
Autoimmune hypophysitis	Guillain-Barré syndrome	Polyglandular autoimmune syndrome
Autoimmune myocarditis	Hashimoto disease	Primary biliary cholangitis
Autoimmune oophoritis	IgA nephropathy	Psoriasis
Autoimmune orchitis	Inflammatory bowel disease	Reiter syndrome
Autoimmune thrombocytopenic purpura	Interstitial cystitis	Rheumatoid arthritis
Behçet disease	Kawasaki disease	Sarcoidosis
Bullous pemphigoid	Lambert-Eaton myasthenia syndrome	Scleroderma
Chronic fatigue syndrome	Lupus erythematosus	Sjögren's syndrome
Chronic inflammatory demyelinating polyneuropathy	Lyme disease - chronic	Stiff-Person syndrome
Churg-Strauss syndrome	Meniere syndrome	Takayasu arteritis
Crohn disease	Mooren ulcer	Ulcerative colitis
	Morphea	Vitiligo
	Multiple sclerosis	Vogt-Koyanagi-Harada disease
	Myasthenia gravis	Wegener granulomatosis

Appendix F: Performance Status Criteria

ECOG Performance Status Scale		Karnofsky Performance Status Scale	
**Grade**	**Descriptions**	**Percent**	**Description**
0	Normal activity. Fully active, able to carry on all pre-disease performance without restriction.	100	Normal, no complaints, no evidence of disease.
		90	Able to carry on normal activity; minor signs or symptoms of disease.
1	Symptoms, but ambulatory. Restricted in physically strenuous activity, but ambulatory and able to carry out work of a light or sedentary nature (eg, light housework, office work).	80	Normal activity with effort; some signs or symptoms of disease.
		70	Cares for self, unable to carry on normal activity or to do active work.
2	In bed < 50% of the time. Ambulatory and capable of all self-care, but unable to carry out any work activities. Up and about more than 50% of waking hours.	60	Requires occasional assistance, but is able to care for most of his/her needs.
		50	Requires considerable assistance and frequent medical care.
3	In bed > 50% of the time. Capable of only limited self-care, confined to bed or chair more than 50% of waking hours.	40	Disabled, requires special care and assistance.
		30	Severely disabled, hospitalization indicated. Death not imminent.
4	100% bedridden. Completely disabled. Cannot carry on any self-care. Totally confined to bed or chair.	20	Very sick, hospitalization indicated. Death not imminent.
		10	Moribund, fatal processes progressing rapidly.
5	Dead.	0	Dead.

Appendix G: Response Evaluation Criteria in Solid Tumors Version 1.1 (RECIST 1.1)

Adapted from Eisenhauer et al 2009

Definitions

Baseline: Baseline is defined as the most recent assessment performed prior to receiving study treatment. Baseline assessments must be performed within the period defined in the protocol eligibility criteria.

Measurable lesions: Except for lymph nodes as described below, measurable lesions are defined as those that can be accurately measured in at least 1 dimension (longest diameter to be recorded) as ≥ 10 mm with CT scan (if CT scans have slice thickness greater than 5 mm the minimum size for a measurable lesion is twice the slice thickness).

- To be considered pathologically enlarged and measurable, a lymph node must be ≥ 15 mm in short axis when assessed by CT scan (CT scan slice thickness recommended to be no greater than 5 mm). At baseline and in follow-up, only the short axis will be measured and recorded.
- MRI may be substituted for contrast-enhanced CT for lesions at some anatomical sites, but not for lesions in the lungs. The minimum size for measurability is the same as for CT (10 mm) as long as the scans are performed with slice thickness of 5 mm and no gap. If MRI is performed with thicker slices, the size of a measurable lesion at baseline should be twice the slice thickness. In the event there are interslice gaps, this also needs to be considered in determining the size of measurable lesions at baseline.

Nonmeasurable lesions: All other lesions (or sites of disease), including small lesions (longest diameter < 10 mm or pathological lymph nodes with ≥10 to < 15 mm short axis), are considered nonmeasurable. Lymph nodes that have a short axis < 10 mm are considered nonpathological and are not be recorded or followed. Bone lesions, leptomeningeal disease, ascites, pleural/pericardial effusions, lymphangitis cutis/ pulmonitis, and abdominal masses (not followed by CT or MRI), are considered as nonmeasurable.

Target lesions: All measurable lesions up to a maximum of 2 lesions per organ and 5 lesions in total, representative of all involved organs, are to be identified as **target lesions** and measured and recorded at baseline. Target lesions are to be selected on the basis of their size (lesions with the longest diameter), be representative of all involved organs, and be those that lend themselves to reproducible repeated measurements. It may be the case that, on occasion, the largest lesion does not lend itself to reproducible measurement in which circumstance the next largest lesion

which can be measured reproducibly should be selected. Target lesions will be measured at each assessment (longest axis for nonnodal lesions, shortest axis for measurable malignant nodal lesions).

Nontarget lesions: All other lesions (or sites of disease) including all non-measurable lesions (including pathological lymph nodes with ≥ 10 to <15 mm short axis) and all measurable lesions over and above the 5 target lesions are to be identified as **non-target lesions** and recorded at baseline. Measurements of these lesions are not required, but the presence, absence, or in rare cases unequivocal progression of each is to be recorded throughout follow-up. Lymph nodes that have a short axis < 10 mm are considered non-pathological and are not to be recorded or followed.

To be considered progression of non-target lesions in the presence of measurable disease, unequivocal progression is defined as substantial worsening in non-target disease such that, even in the presence of SD or PR in target disease, the overall tumor burden has increased sufficiently to merit discontinuation of the therapy.

Special Consideration

Lesions by clinical examination will not be used for response in this study.

Cystic lesions

- Cystic lesions that meet the criteria for radiographically defined simple cysts should not be considered as malignant lesions (neither measurable nor nonmeasurable) since they are, by definition, simple cysts.
- Cystic lesions thought to represent cystic metastases can be considered as measurable lesions, if they meet the definition of measurability described above. However, if noncystic lesions are present in the same subject, these are preferred for selection as target lesions.

Bone lesions

- Bone scan, PET scan or plain films are not considered adequate imaging techniques to measure bone lesions.
- Lytic bone lesions or mixed lytic-blastic lesions, with identifiable soft tissue components, that can be evaluated by cross-sectional imaging techniques such as CT or MRI can be considered as measurable lesions if the *soft tissue component* meets the definition of measurability described above.
- Blastic bone lesions are non-measurable.

Lesions with prior local treatment

- Lesions situated in a previously irradiated area, or in an area subjected to other loco-regional therapy, are not considered measurable.

Imaging Methods

The same method of assessment and the same technique used to characterize each identified and reported lesions at baseline should be used during each follow-up assessment. All measurements should be taken and recorded in metric notation using a ruler or calipers. Imaging based evaluation is preferred to evaluation by clinical examination unless the lesion(s) being followed cannot be imaged but assessed by clinical examination (referring to biopsy-proven visible lesion(s) on the chest).

Chest x-ray: Chest x-ray will not be used for response assessment in this study.

Conventional CT and MRI: This guideline has defined measurability of lesions on CT scan based on the assumption that CT slice thickness is 5 mm or less. If CT scans have slice thickness greater than 5 mm, the minimum size for a measurable lesion is twice the slice thickness. MRI is also acceptable in certain situations (eg, for body scan) except for lung.

Use of MRI remains a complex issue. MRI has excellent contrast, spatial, and temporal resolution; however, there are many image acquisition variables involved in MRI, which greatly impact image quality, lesion conspicuity, and measurement. Furthermore, the availability of MRI is variable globally. As with CT, if an MRI is performed, the technical specifications of the scanning sequences used should be optimized for the evaluation of the type and site of disease. Furthermore, as with CT, the modality used at follow-up should be the same as was used at baseline and the lesions should be measured/assessed on the same pulse sequence. It is beyond the scope of the RECIST guidelines to prescribe specific MRI pulse sequence parameters for all scanners, body parts, and diseases. Ideally, the same type of scanner should be used and the image acquisition protocol should be followed as closely as possible to prior scans. Body scans should be performed with breath-hold scanning techniques, if possible.

Low dose non-contrast CT images from combined positron emission tomography/computed tomography (PET/CT) imaging cannot be used for tumor evaluations in this study.

Ultrasound: Ultrasound will not be used for response assessment in this study.

Bone scans will be used to assess the presence or disappearance of the bone component of bone lesions. CT or MRI scan will be used to confirm results of bone scans. Preferred method for confirmation is MRI.

Bone scan findings alone cannot be used for the determination of progression or response in this study and need to be corroborated by CT/MRI. Bone lesions corroborated by CT/MRI must be reported as non-target or new lesions. PET scan or plain films are not considered adequate imaging techniques to measure bone lesions.

Tumor Markers: Tumor markers may be evaluated for changes but will not be used to determine progressive disease in this study.

Cytology, Histology: The cytological confirmation of the neoplastic origin of any effusion that appears or worsens during treatment can be considered if the measurable tumor has met criteria for response or stable disease in order to differentiate between response (or stable disease) and progressive disease.

Time Point Assessments

The frequency and schedule of tumor assessments is defined in the protocol. The schedule is to be maintained regardless of whether study treatment is reduced, interrupted, delayed, or discontinued.

At baseline, tumors and lymph nodes are classified and documented as target or nontarget lesions per the definitions provided above. It is possible to record multiple nontarget lesions involving the same organ as a single item (eg, 'multiple liver metastases'). At all post-baseline (follow-up) evaluations the baseline classification (target, nontarget) is to be maintained and lesions are to be documented and described in a consistent fashion over time (eg, recorded in the same order on source documents).

At each assessment, a sum of the diameters (longest for nonnodal lesions, short axis for nodal lesions) for all target lesions will be calculated and included in source documents. The *baseline sum of the diameters* (SoD) will be used as reference to further characterize any objective tumor regression in the measurable dimension of the disease. The lowest SoD (nadir) since (and including) the baseline value will be used as reference for evaluating progression.

After baseline, target lesions should have the actual size documented, if possible, even if the lesions become very small. If in the opinion of the radiologist the lesion has likely disappeared, 0

mm should be recorded. If the lesion is present but too small to measure, an indicator for ‘too small to measure’ should be included in source documents.

For target lesions, measurements should be taken and recorded in metric notation.

Nontarget lesions are to be assessed qualitatively (present, resolved, or unequivocal progression) and new lesions, if any, are to be documented separately.

At each evaluation, progression status is to be determined based upon the time point status for target lesions, nontarget lesions, and new lesions.

Finding of new lesions should not be attributable to differences in scanning technique, change in imaging modality or findings thought to represent something other than tumor. Necrosis of pre-existing lesions as part of a response to treatment should be excluded before defining a ‘new’ cystic lesion. A lesion identified on a follow-up study in an anatomical location that was not scanned at baseline is considered a new lesion. If a new lesion is equivocal because of its small size, repeat scans need to confirm there is definitely a new lesion, and progression should be declared using the date of the initial scan.

Time point progression cannot be based solely on bone scan findings. Bone scans are to be used to direct corroborative imaging with CT/MRI if necessary. These CT/MRI findings will be used for the determination of progression.

TIME POINT RESPONSE CRITERIA

Target Lesion Time Point Response (TPR)	
**Complete Response (CR)**	Disappearance of all target lesions. All pathological lymph nodes (whether target or non-target) must have reduction in short axis to < 10 mm.
**Partial Response (PR)**	At least a 30% decrease in SoD of target lesions, taking as a reference the baseline SoD.
**Stable Disease (SD)**	Neither sufficient shrinkage to qualify for PR nor sufficient increase to qualify for PD.
**Progressive Disease (PD)**	At least a 20% increase in the SoD of target lesions, taking as a reference the smallest (nadir) SoD since (and including) baseline. In addition to the relative increase of 20%, the SoD must also demonstrate an absolute increase of at least 5 mm.
**Not Applicable (NA)**	No target lesion identified at baseline.
**Unable to Evaluate (UE)**	One or more target lesions are not imaged and the remainder of the SoD compared with the nadir SoD does not meet the criterion for PD.

SoD, baseline sum of diameters (longest for non-nodal lesions; short axis for nodal lesions).

If the target lesion for a subject meet the criteria for both PR and PD at a given time point, the target lesion response is PD.

If the nadir of SoD is 0 (ie, the subject had a prior target lesion CR), the reappearance of any prior target lesion to any degree constitutes PD.

Non-Target Lesion Time Point Response (TPR)	
**Complete Response (CR)**	Disappearance of all non-target lesions. All lymph nodes must be non-pathological in size (<10 mm short axis).
**Non-CR / Non-PD**	Persistence of one or more non-target lesion(s).
**Progressive Disease (PD)**	Unequivocal progression of non-target lesions. Unequivocal progression should normally not trump target lesion status. It must be representative of overall disease status change, not a single lesion increase.
**Not Applicable (NA)**	No non-target lesions identified at screening.
**Unable to Evaluate (UE)**	One or more non-target lesions are not imaged and the remaining non-target lesions do not meet the criterion for PD.

New Lesion Time Point Response (TPR)	
**Yes**	Lesion present at follow-up visit either for the very first time or re-appearing (ie, lesion was present at baseline, disappeared at a follow-up visit and re-appeared later). Note: The appearance of one or more new lesions on CT or MRI scan is considered progression if these findings are unequivocally not due to a change in the imaging technique or modality. On bone scan, new lesions are not sufficient to qualify as PD. Confirmation should be obtained by performing CT or MRI of the area of concern to confirm results of bone scan. Preferred method for confirmation is MRI.
**No**	No new lesions present at follow-up.
**Unable to Evaluate (UE)**	Subject not assessed or incompletely assessed for new lesions.

Evaluation of Overall Time Point Response			
**Target Lesion TPR**	**Non-target lesion TPR**	**New lesion TPR**	**Overall TPR**
CR	CR or NA	No	CR*
CR	Non-CR/non-PD	No	PR*
CR	UE	No	PR*
PR	Non-PD or NA or UE	No	PR*
SD	Non-PD or NA or UE	No	SD
UE	Any except PD	No	UE
PD	Any	No or Yes	PD
Any	PD	No or Yes	PD
Any	Any	Yes	PD**
NA	CR	No	CR*
NA	Non-CR/Non-PD	No	Non-CR/non-PD
NA	UE	No	UE

CR, complete response; PR, partial response; SD, stable disease; PD, progressive disease, TPR, time point response; UE, unable to evaluate; NA, not applicable (no such lesions at screening); Any, CR, PR, SD, PD, NA, or UE.

The overall response at a given time point does not depend upon the overall response assigned at any prior or subsequent time point (ie, confirmation requirement are not considered when assigning time point responses).

* Subjects with an overall response of CR or PR must have a repeat tumor assessment performed no less than 4 weeks after the criteria for response are first met. However, the presence or absence of confirmation is not considered when assigning a time point response.

** If a lesion disappears and reappears at a subsequent time point it should continue to be measured. However, the subject's response at the point in time when the lesion reappears will depend upon the status of his/her other lesions. For example, if the subject's tumor had reached a CR status and the lesion reappeared, then the subject would be considered PD at the time of reappearance. In contrast, if the tumor status was a PR or SD and one lesion which had disappeared then reappears, its maximal diameter should be added to the sum of the remaining lesions for a calculated response.

Confirmation

The main goal of confirmation of objective response is to avoid overestimating the response rate observed. For subjects with an overall response of PR or CR at a given time point, changes in tumor measurements must be confirmed by repeat assessments that should be performed no less than 4 weeks after the criteria for response are first met. However, the presence or absence of confirmation is not considered when assigning a time point response. Longer intervals as determined by the study protocol may also be appropriate.

Best Overall Response

Best overall response, incorporating confirmation requirements, will be derived during statistical analysis from the series of time point responses and need not be considered when assigning response at each time point.

Appendix H: Immune-Related Response Criteria (irRECIST)

Immune-related Response Criteria (irRECIST) are adapted from Wolchock et al 2009 and Nishino et al 2013.

Key aspects of irRECIST for immune-related response assessment:

- New lesions:
  - New lesions after baseline do not necessarily define radiographic progression
  - New measurable lesions are added into the total tumor burden and followed at subsequent tumor assessments
  - Unmeasurable new lesions preclude complete response status
- Non-target lesions:
  - Non-target lesion progression does not define radiographic progression
  - Disappearance of all non-target lesions is required for complete response status
- Radiographic progression:
  - Is determined only on the basis of measurable disease
  - Is defined by a ≥ 20% increase of sum of lesion diameter (SLD; including measurable new lesions)
  - Radiographic progression that is not confirmed ≥ 4 weeks from the first date documented is not radiographic progression by immune-response criteria
  - Best response may occur after any number of radiographic progression assessments

Evaluation of Overall Immune-related Time Point Response by irRECIST Criteria

Target Lesion TPR	Non-Target Lesion TPR	New Measurable Lesion	New Non-Measurable Lesion	% Change in irSLD Tumor Burden (Including Measurable New Lesions)	Overall Immune-Related TPR
CR	CR	No	No	-100%	irCR
PR	Any	Any	Any	≤ -30%	irPR
SD	Any	Any	Any	> -30% to < +20%	irSD
PD	Any	Any	Any	≥ +20%	irPD

CR, complete response; PR, partial response; SD, stable disease; PD, progressive disease, ir, immune-related; SLD, sum of lesion diameter; TPR, time-point response.

Time point responses and best overall response per irRECIST, incorporating confirmation requirements, will be derived during statistical analysis from the tumor evaluations performed by the investigator.

Appendix I: Infusion-Related Reaction and Cytokine-Release Syndrome Guidelines

Event	Management
Grade 1 [a]<br>Fever [b] with or without constitutional symptoms	• Immediately interrupt infusion.<br>• Upon symptom resolution, wait for 30 minutes and then restart infusion at half the rate being given at the time of event onset.<br>• If the infusion is tolerated at the reduced rate for 30 minutes, the infusion rate may be increased to the original rate.<br>• If symptoms recur, discontinue infusion of this dose.<br>• Administer symptomatic treatment [c], including maintenance of IV fluids for hydration.<br>• In case of rapid decline or prolonged CRS (> 2 days) or in subjects with significant symptoms and/or comorbidities, consider managing as per Grade 2.<br>• For subsequent infusions, consider administration of oral premedication with antihistamines, antipyretics, and/or analgesics, and monitor closely for IRRs and/or CRS.
Grade 2 [a]<br>Fever [b] with hypotension not requiring vasopressors<br>**and/or**<br>Hypoxia requiring low-flow oxygen [d] by nasal cannula or blow-by	• Immediately interrupt infusion.<br>• Upon symptom resolution, wait for 30 minutes and then restart infusion at half the rate being given at the time of event onset.<br>• If symptoms recur, discontinue infusion of this dose.<br>• Administer symptomatic treatment.[c]<br>• For hypotension, administer IV fluid bolus as needed.<br>• Monitor cardiopulmonary and other organ function closely (in the ICU, if appropriate). Administer IV fluids as clinically indicated, and manage constitutional symptoms and organ toxicities as per institutional practice.<br>• Rule out other inflammatory conditions that can mimic CRS (eg, sepsis). If no improvement within 24 hours, initiate workup and assess for signs and symptoms of HLH or MAS.<br>• Consider IV corticosteroids (eg, methylprednisolone 2 mg/kg/day or dexamethasone 10 mg every 6 hours).<br>• Consider anti-cytokine therapy.[e]<br>• Consider hospitalization until complete resolution of symptoms. If no improvement within 24 hours, manage as per Grade 3, ie, hospitalize subject (monitoring in the ICU is recommended), permanently discontinue atezolizumab, and contact the Sponsor.<br>• If symptoms resolve to Grade 1 or better for 3 consecutive days, the next dose of atezolizumab may be administered. For subsequent infusions, consider administration of oral premedication with antihistamines, antipyretics, and/or analgesics and monitor closely for IRRs and/or CRS.<br>• If symptoms do not resolve to Grade 1 or better for 3 consecutive days, contact the Sponsor.

Event	Management
Grade 3 [a]<br>Fever [b] with hypotension requiring a vasopressor (with or without vasopressin) **and/or**<br>Hypoxia requiring high-flow oxygen [d] by nasal cannula, face mask, non-rebreather mask, or Venturi mask	• Permanently discontinue atezolizumab and contact the Sponsor. [f]<br>• Administer symptomatic treatment. [c]<br>• For hypotension, administer IV fluid bolus and vasopressor as needed.<br>• Monitor cardiopulmonary and other organ function closely; monitoring in the ICU is recommended. Administer IV fluids as clinically indicated, and manage constitutional symptoms and organ toxicities as per institutional practice.<br>• Rule out other inflammatory conditions that can mimic CRS (eg, sepsis). If no improvement within 24 hours, initiate workup and assess for signs and symptoms of HLH or MAS.<br>• Administer IV corticosteroids (eg, methylprednisolone 2 mg/kg/day or dexamethasone 10 mg every 6 hours).<br>• Consider anti-cytokine therapy. [e]<br>• Hospitalize subject until complete resolution of symptoms. If no improvement within 24 hours, manage as per Grade 4, ie, admit subject to ICU and initiate hemodynamic monitoring, mechanical ventilation, and/or IV fluids and vasopressors as needed; for subjects who are refractory to anti-cytokine therapy, experimental treatments may be considered at the discretion of the investigator and in consultation with the Sponsor.
Grade 4 [a]<br>Fever [b] with hypotension requiring multiple vasopressors (excluding vasopressin) **and/or**<br>Hypoxia requiring oxygen by positive pressure (eg, CPAP, BiPAP, intubation and mechanical ventilation)	• Permanently discontinue atezolizumab and contact the Sponsor. [f]<br>• Administer symptomatic treatment. [c]<br>• Admit subject to ICU and initiate hemodynamic monitoring, mechanical ventilation, and/or IV fluids and vasopressors as needed. Monitor other organ function closely. Manage constitutional symptoms and organ toxicities as per institutional practice.<br>• Rule out other inflammatory conditions that can mimic CRS (eg, sepsis). If no improvement within 24 hours, initiate workup and assess for signs and symptoms of HLH or MAS.<br>• Administer IV corticosteroids (eg, methylprednisolone 2 mg/kg/day or dexamethasone 10 mg every 6 hours).<br>• Consider anti-cytokine therapy [e]. For subjects who are refractory to anti-cytokine therapy, experimental treatments [g] may be considered at the discretion of the Investigator and in consultation with the Sponsor.<br>• Hospitalize subject until complete resolution of symptoms.

ASTCT, American Society for Transplantation and Cellular Therapy; BiPAP, bi-level positive airway pressure; CAR, chimeric antigen receptor; CPAP, continuous positive airway pressure; CRS, cytokine-release syndrome; CTCAE, Common Terminology Criteria for Adverse Events; eCRF, electronic Case Report Form; HLH, hemophagocytic lymphohistiocytosis; ICU, intensive care unit; IRR, infusion-related reaction; MAS, macrophage activation syndrome; NCCN, National Cancer Comprehensive Network; NCI, National Cancer Institute.

Note: The management guidelines have been adapted from NCCN guidelines for management of CAR T-cell-related toxicities (Version 2.2019).

[a] Grading system for these management guidelines is based on ASTCT consensus grading for CRS. NCI CTCAE (version as specified in the protocol) should be used when reporting severity of IRRs, CRS, or organ toxicities associated with CRS on the AE eCRF. Organ toxicities associated with CRS should not influence overall CRS grading.

[b] Fever is defined as temperature ≥ 38°C not attributable to any other cause. In subjects who develop CRS and then receive antipyretic, anti-cytokine, or corticosteroid therapy, fever is no longer required when subsequently determining event severity (grade). In this case, the grade is driven by the presence of hypotension and/or hypoxia.

[c] Symptomatic treatment may include oral or IV antihistamines, antipyretics, analgesics, bronchodilators, and/or oxygen. For bronchospasm, urticaria, or dyspnea, additional treatment may be administered as per institutional practice.

[d] Low flow is defined as oxygen delivered at ≤ 6 L/min, and high flow is defined as oxygen delivered at > 6 L/min.

[e] There are case reports where anti-cytokine therapy has been used for treatment of CRS with immune checkpoint inhibitors (Rotz et al 2017; Adashek and Feldman 2019), but data are limited, and the role of such treatment in the setting of antibody-associated CRS has not been established.

[f] Resumption of atezolizumab may be considered in subjects who are deriving benefit and have fully recovered from the event. The decision to re-challenge subjects with atezolizumab should be based on Investigator's assessment of benefit-risk and documented by the Investigator (or an appropriate delegate). The Sponsor is available to advise as needed. For subsequent infusions, administer oral premedication with antihistamines, antipyretics, and/or analgesics, and monitor closely for IRRs and/or CRS. Premedication with corticosteroids and extending the infusion time may also be considered after assessing the benefit-risk ratio.

[g] Refer to Riegler et al (2019) for information on experimental treatments for CRS.

Appendix J: Potential Drug Interactions with Cabozantinib

The Investigator should evaluate concomitant medications prior to initiation for potential drug interactions with cabozantinib through the CYP3A4 pathway. The table below shows examples of potential strong inhibitors and inducers of CYP3A4.

Strong Inhibitors of CYP3A4		Strong Inducers of CYP3A4
Conivaptan	**Anti-Fungals**	Carbamazepine
Diltiazem	Itraconazole	Efavirenz
Grapefruit Juice	Ketoconazole	Enzalutamide
Idelalisib	Posaconazole	Erythromycin
Nefazodone	Voriconazole	Mitotane
**Antivirals**	**Antibiotics**	Modafinil
Boceprevir	Clarithromycin	Nevirapine
Cobicistat	Telithromycin	Oxcarbazepine
Conivaptan	Troleandomycin	Phenytoin
Danoprevir		Rifampin
Dasabuvir		St. John's wort
Elvitegravir		
Indinavir		
Lopinavir		
Nelfinavir		
Ombitasvir		
Paritaprevir		
Ritonavir		
Saquinavir		
Telaprevir		
Tipranavir		

This table is not all-inclusive. Please refer to the FDA website for the most updated lists of substrates, inducers, and inhibitors of selected CYP450 isozyme pathways:

- http://www.fda.gov/Drugs/DevelopmentApprovalProcess/DevelopmentResources/DrugInteractionsLabeling/ucm080499.htm.

Appendix K: Methods of Contraception

In Inclusion Criterion 9 (Study Synopsis and Protocol Section 4.2):

Sexually active fertile subjects and their partners must agree to use highly effective methods of contraception that alone or in combination result in a failure rate of less than 1% per year when used consistently and correctly during the course of the study and for 5 months after the last dose of study treatment. Such methods include:

- Placement of an intrauterine device (IUD)
- Placement of an intrauterine hormone-releasing system (IUS)
- Bilateral tubal occlusion
- Vasectomized partner
- Sexual abstinence (the reliability of sexual abstinence needs to be evaluated in relation to the preferred and usual lifestyle of the subject)
- Combined (estrogen- and progestogen-containing) hormonal contraception*:
    - Oral
    - Intravaginal
    - Dermal
- Progestogen-only hormonal contraception associated with inhibition of ovulation*:
    - Oral
    - Injectable
    - Implantable

* The effect of cabozantinib on the pharmacokinetics of contraceptive steroids has not been investigated. Because oral contraceptives might possibly not be considered as “effective methods of contraception,” they should be used together with another method.

Furthermore, male subjects must refrain from donating sperm in order to avoid transmission of study treatment in semen for the duration of study treatment and through 5 months after their last dose of study treatment.

Appendix L: Child-Pugh Scoring System for Subjects with Chronic Liver Disease

Modified Child-Pugh classification of severity of liver disease (Pugh et al 1973, Lucey 1997) for subjects with chronic liver disease is according to the degree of ascites, total bilirubin and albumin, prothrombin time, and degree of encephalopathy. Each measure is scored 1-3, with 3 indicating greatest severity:

	Points assigned		
**Parameter**	**1**	**2**	**3**
Ascites	none	mild/moderate (diuretic-responsive)	tense (diuretic-refractory)
Total bilirubin, mg/dL	< 2	2–3	> 3
Albumin, g/dL	> 3.5	2.8–3.5	< 2.8
Prothrombin time			
Seconds over control	1–3	4–6	> 6
or			
INR	< 1.7	1.7–2.3	> 2.3
Encephalopathy	none	Grade 1–2 (or precipitant-induced)	Grade 3–4 (chronic)

Child-Pugh score (A, B, or C) based on total score from the above point assignments:

Grade	Points	1-year survival	2-year survival
A: well-compensated disease	5–6	100%	85%
B: significant functional compromise	7–9	80%	60%
C: decompensated disease	10–15	45%	35%

Appendix M: COVID-19 Instructions

This appendix describes contingencies and accommodations for sites and subjects impacted by the COVID-19 pandemic. In response to the evolving circumstances of the pandemic, the Sponsor will provide ongoing guidance to Investigators on study conduct to ensure subject safety and maintain the scientific integrity of the study. Investigators must also maintain awareness of and respond to instructions and guidelines from their local regulatory authorities during the pandemic. These will be temporary measures and are applicable only during the pandemic, and as necessary to abide by local public health requirements. These measures will be repealed back to the measures described in the full study protocol as soon as the situation (governmental rules, benefit/risk assessment for the trial) allows.

Under the exceptional circumstances of the COVID-19 pandemic where enrolled subjects are not able or willing to physically access the site clinic, the following accommodations may be permitted if allowed by local and other applicable regulations (Note: special accommodations are not permitted for screening assessments):

- Safety assessments should still be performed unless the Investigator and Sponsor agree that specific assessments may be missed as long as this occurs in accordance with all applicable local regulations. However, at a minimum, the Investigator or designee must regularly contact the subject (eg, by phone) to ascertain the subject's condition and occurrence of any symptom-based AEs per the relevant protocol -defined visit schedule. If available, results of any remote assessments performed by a non-study local oncologist or primary care physician must be sent to the Investigator for review and documentation. If components of the safety assessment cannot be collected or the timing of safety assessments needs to be adjusted, it may be possible to continue with study treatment but this will have to be discussed on a case-by-case basis with the Medical Monitor. Any remote laboratory assessments must be performed by laboratories accredited by the local jurisdiction.

- Tumor assessments may be performed at another radiology facility rather than at the study site (this option is not available in Germany). Such facilities should perform tumor assessments in accordance with the protocol, but alternative image acquisition protocols (eg, single post-contrast vs triple phase) may be accepted if the preferred modality is not available. The treatment modality (eg, CT scan or MRI) should be the same as that utilized since the start of study entry in order to avoid discrepancies in imaging interpretation. Imaging should be performed within or as close to the study visit window for scheduled imaging time points as possible. The study site must collect tumor images generated off site in a timely fashion for review and documentation by the Investigator and submission to the BIRC if applicable.

- Alternative methods of distribution of oral treatment to subjects may be considered in accordance with the study site's local policies and all applicable regulations. Confirmation of drug receipt will be obtained by sites.
- Intravenous study treatment should generally only be administered at the study site, but circumstances may arise where the subject may receive infusions in another location under the supervision of the Investigator, with the approval of the Sponsor, and in accordance with all applicable regulations.

If logistical challenges in providing study treatment or performing study-related assessments result in temporary interruption of all study treatment for greater than 12 weeks, subjects are required to permanently discontinue study treatment unless permitted to continue by the Sponsor.

If it becomes necessary to employ any of the accommodations described in this appendix of COVID-19 Instructions, Investigators are to document each incident in source records as COVID-related. To comply with emerging regulatory guidance that such accommodations be reported and their impact on the study assessed, these will be collected by the Sponsor (or designee) as protocol-deviations. However, no corrective action will generally be expected if this appendix is followed.

Subjects are to be informed of changes to standard procedures resulting from effects of the COVID-19 pandemic, and if necessary, subject consent is to be acquired. If additional consent is necessary during the course of the study but cannot be immediately obtained from the subject in writing, the Investigator is to describe to the subject the additional information requiring consent, obtain verbal consent from the subject, document such consent in the subject file, and follow up with written consent the next time a subject returns to the site. This does not apply to initial consent to enter the study; in this case, written consent is still required.

For subjects who develop COVID-19 while on study, the Investigator is to evaluate the overall risk-benefit ratio for the subject to determine whether holding study treatment(s) is in the best interest of the subject.

Any cases of confirmed or suspected COVID-19 infections should follow the general AE reporting requirements defined in the protocol. For any confirmed or suspected COVID-19 cases, the Investigator is responsible for assessing if the event should be reported as an SAE using their clinical judgment. The investigator should further consider if the diagnosis meets the criteria of being a significant medical event.

When recording data missing, impacted, or related to COVID-19 in the electronic CRFs, the conventions below are to be employed. Refer to updated CRF Completion Guidelines and site communication memos for additional instructions on how to document data missing or impacted by COVID-19.

**Case Report Form**	**Instructions**
Adverse Event CRF	• Record COVID-19 diagnoses as "COVID-19"<br>• Record suspected cases as "suspected COVID-19"<br>• If death is the outcome of such an event, the CTCAE grade should be assigned as '5'<br>See the CRF instructions on how to enter fatal events that started at a lower grade
End of Study Treatment CRFs	• Investigators are to use their best judgment to identify the primary reason for study treatment discontinuation<br>• If study treatment ended primarily due to a logistical issue associated with the COVID-19 pandemic and was unrelated to cancer progression or any AE:<br>o Indicate "Other" as the reason for treatment discontinuation and describe the reason in the Specify field, including the term "COVID-19"<br>For example – Other, Specify: "Subject unable to travel due to COVID-19 restrictions"<br>• If study treatment ended primarily due to an AE caused by COVID-19 or suspected COVID-19:<br>o Indicate "AE/SAE unrelated to progression of disease under study"<br>o Record the AE on the Adverse Event CRF as described above with Action Taken = "Treatment Discontinued"
End of Radiographic Follow-Up CRF	• If radiographic assessments ended primarily due to a logistical issue or AE caused by COVID-19 or suspected COVID-19:<br>o Indicate "Other" as the reason for discontinuation and describe the reason in the Specify field, including the term "COVID-19"<br>For example – Other, Specify: "Subject unable to travel due to COVID-19 restrictions" or "Subject discontinued due to hospitalization for suspected COVID-19". In the latter example, also record the AE on the Adverse Event CRF as "suspected COVID-19"
Study Treatment CRFs	• If study treatment was held or delayed solely due to a logistical issue associated with the COVID-19 pandemic:<br>o For oral study treatment CRFs: Indicate "Other" as the reason the dosing interval ended and describe the reason in the Specify field, including the term "COVID-19".<br>o For IV dosing CRFs: Enter "Yes" for "Dose delayed from prior infusion" and "Reason for dose delay" should be entered as "Other" and describe the reason in the Specify field, including the term "COVID-19" (if/when 'Specify' field is available)<br>For example – Other, Specify: "Subject unable to travel due to COVID-19 restrictions"
